# Identification of potential genetic causal variants for rheumatoid arthritis by whole-exome sequencing

**DOI:** 10.18632/oncotarget.22630

**Published:** 2017-11-22

**Authors:** Ying Li, Elaine Lai-Han Leung, Hudan Pan, Xiaojun Yao, Qingchun Huang, Min Wu, Ting Xu, Yuwei Wang, Jun Cai, Runze Li, Wei Liu, Liang Liu

**Affiliations:** ^1^ State Key Laboratory of Quality Research in Chinese Medicine/Macau Institute for Applied Research in Medicine and Health, Macau University of Science and Technology, Macau, China; ^2^ Guangdong Provincial Hospital of Traditional Chinese Medicine, Guangzhou, China; ^3^ The Third Affiliated Hospital of Soochow University, Changzhou, China; ^4^ The First Teaching Hospital of Tianjin University of Traditional Chinese Medicine, Tianjin, China

**Keywords:** rheumatoid arthritis, whole-exome sequencing, Chinese population, genetic causal variants, homology modeling

## Abstract

Rheumatoid arthritis (RA) is a highly prevalent chronic autoimmune disease. However, genetic and environmental factors involved in RA pathogenesis are still remained largely unknown. To identify the genetic causal variants underlying pathogenesis and disease progression of RA patients, we undertook the first comprehensive whole-exome sequencing (WES) study in a total of 124 subjects including 58 RA cases and 66 healthy controls in Han Chinese population. We identified 378 novel genes that were enriched with deleterious variants in RA patients using a gene burden test. The further functional effects of associated genetic genes were classified and assessed, including 21 newly identified genes that were involved in the extracellular matrix (ECM)-receptor interaction, protein digestion and absorption, focal adhesion and glycerophospholipid metabolism pathways relevant to RA pathogenesis. Moreover, six pathogenic variants were investigated and structural analysis predicted their potentially functional alteration by homology modeling. Importantly, five novel and rare homozygous variants (*NCR3LG1*, *RAP1GAP*, *CHCHD5*, *HIPK2* and *DIAPH2*) were identified, which may exhibit more functional impact on RA pathogenesis. Notably, 7 genes involved in the olfactory transduction pathway were enriched and associated with RA disease progression. Therefore, we performed an efficient and powerful technique WES in Chinese RA patients and identified novel, rare and common disease causing genes that alter innate immunity pathways and contribute to the risk of RA. Findings in this study may provide potential diagnostic tools and therapeutic strategies for RA patients.

## INTRODUCTION

Rheumatoid arthritis (RA) is the most common form of systemic autoimmune arthritis with unknown etiology, characterized by systemic inflammation and persistent poly-joint synovitis, principally leading to injury of the flexible joints, often with symptoms of joint pain and swelling, stiffness, bone destruction and fatigue, as well as implications of extra articular organs [1, 2]. The prevalence of RA varies largely in different populations, from 0.25% in Eastern Asians to 0.75% in European ancestry, and to as high as 6% in American Indians [3]. It remains largely unknown whether genetics, cultural, or environmental factors contribute to these differences. During the past years, an increasing list of genetic associations with RA has emerged from genome wide association studies (GWAS), which attributes great relevance to immune system contributed by profound sources of genetic variation with a panel of surface and intracellular signaling molecules as well as cytokines [4, 5]. GWAS has also revealed a complex picture of both shared and population-specific genetic susceptibility loci to this autoimmune disease in comparison of Asian and European populations [6, 7]. Generally, GWASs are designed to capture common genetic variation, and to date, a large portion of the heritability of complex traits has not been explained [8], which has prompted us to explore other potential sources of genetic susceptibility to RA, such as rare variants.

Whole-exome sequencing (WES) has become a popular and powerful technique for the identification of rare variants that alter protein functions, which may contribute to disease pathogenesis [9-11]. Protein-coding variants are more straightforward to annotate biological functions and pinpoint causative genes. To our knowledge, there is no comprehensive WES on RA in Han Chinese population in the literatures yet. As nowadays most RA-associated polymorphisms identified by GWASs are in non-coding region (Supplementary Table 6), in our study, we performed WES of 124 subjects in Chinese ancestry to investigate both rare and common variants predicted to be deleterious. In addition, we further characterized their cellular biological function categories of multiple risk variants based on the analysis of ingenuity pathways. Using this strategy, we have for the first time demonstrated potential causal variants resulted from the established RA risk loci as well as from novel candidate genes and pathways associated with RA in Chinese population.

## RESULTS

### Deleterious variants in novel RA candidate genes

**Figure 1:**
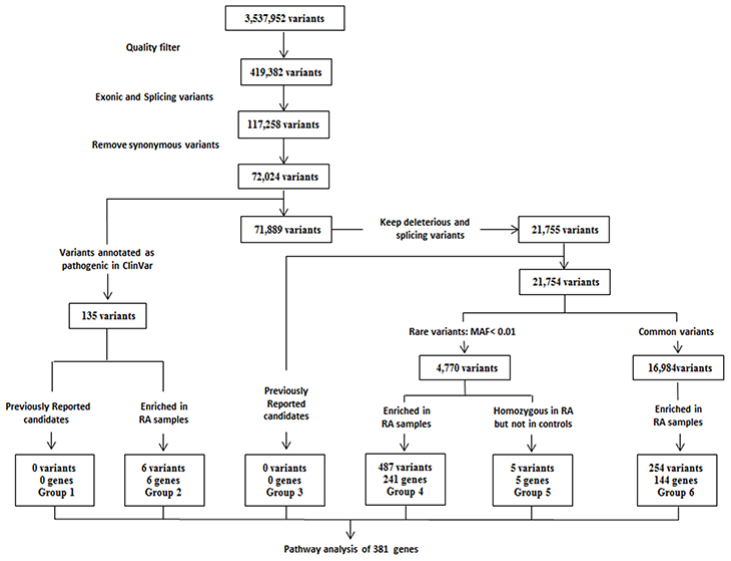
Experimental workflow of whole-exome sequencing was shown to detect and prioritize variants conferring susceptibility to rheumatoid arthritis (RA) using variant filtration and gene burden analysis The variant list for all groups can be found in Supplementary Table 3. MAF = minor allele frequency in the 1000 Genomes Southern Han Chinese (phase III) population. Pathway analysis in candidate genes identified from 58 RA patients was performed using DAVID 6.8 (https://david.ncifcrf.gov/summary.jsp).

WES data were generated from 58 RA patients with a median coverage of 76-fold on targeted exome regions (Supplementary Figure 1A). An average of 96% of all targeted regions was covered by at least 20-fold. The healthy controls had a median coverage of 68-fold on targeted exome regions, and an average of 94% of those regions was covered by at least 20-fold (Supplementary Figure 1B). A total of 3,537,952 variants were identified from 58 RA samples (Figure 1). After applying the quality filters by removing synonymous variants, we found 72,024 exonic and splicing variants, including nonsynonymous substitutions and a small number of stop-gain, stop-loss, frameshift and non-frameshift indels (Supplementary Table 1). Of these, 135 variants were identified as deleterious based on the “pathogenic” annotation in ClinVar, and an additional 21,755 variants predicted to be deleterious were identified using an ensemble logistic regression score.

It was initial surprising that our identified genes were not found in previously reported candidate risk variants GWAS data (group 1 and group 3 in Figure 1; Supplementary Table 3 and 6), such as HLA-associated genes. However, after reviewing the location of the reported RA-associated variants, we found that only 9 of over 200 variants are located in the exome area, including *CTLA4* (rs231775), *FCGR2A* (rs1801274), *IL6R* (rs2228145), *OLIG3* (rs2230926), *PTPN22* (rs2476601), *RTKN2* (rs3125734), *SH2B3* (rs3184504), *TNFAIP3* (rs223092) and *TYK2* (rs34536443). Our data has applied WES technique focusing on exome region, thus explaining the reason why we have identified a new set of RA-associated genes. Interestingly, two novel risk variant loci were identified associated with *TGFβ1* (transforming growth factor β1) and *FOXP3* (forkhead box P3) genes (group 4 and group 6 in Figure 1; Supplementary Table 3) whereas other known variations of these two genes were previously found to be involved in the risk to RA [12-14].

In order to identify novel genes and pathways that could enhance understanding of RA pathogenesis, we performed a gene burden analysis to identify genes for which deleterious variants were enriched in our Han Chinese RA samples compared to healthy control and public control samples. Six such genes were identified (group 2 in Figure 1; Supplementary Table 2 and 3). Of these, a missense variant of *SAA1* (Serum Amyloid A1) was found in 3 RA patients but not present in healthy controls. SAA1 is highly expressed in response to inflammation and tissue injury, and strongly associated with activity of the disease and risk of cardiovascular and renal involvement in RA patients [15-17], suggesting that this novel deleterious variant may potentially contribute to RA disease risk through its interference with proinflammatory effectors. Additional pathogenic variant of *OXCT1* (3-Oxoacid CoA-Transferase 1) was predicted to be damaging (disease-related, D) in our RA patients, encoding Succinyl-CoA:3-ketoacid coenzyme A transferase 1 (SCOT1), which is a key enzyme for synthesis and degradation of ketone bodies involved in cardiovascular disease [18, 19].

Rare variants are more likely to predict a significant impact on protein function and result in clinically relevant consequences than common ones [20]. Thus, we grouped variants that were indicated to be deleterious into rare (minor allele frequency <1%) and common variants, which did not overlap with previously reported candidates (Supplementary Table 6). Performing a gene burden analysis for variants within each of these groups, we identified 241 genes (group 4 in Figure 1; Supplementary Table 3) with rare, deleterious variants specifically enriched in our Chinese RA samples compared to healthy control and public control samples. Notably, since the functional impact of rare and deleterious variants is likely to be greater when present as homozygote, 5 rare and deleterious homozygous variants (*NCR3LG1*, *RAP1GAP*, *CHCHD5*, *HIPK2* and *DIAPH2*) were identified in the Chinese RA samples and absent in the controls (group 5 in Figure 1; Supplementary Table 3). Finally, we also identified 144 genes with common and deleterious variants in RA patients (group 6 in Figure 1; Supplementary Table 3).

### Pathway discovery

Using our methodology, we identified a total of 381 genes as candidates for increased risk of RA (Supplementary Table 3). In order to identify the associated biologic pathways, we performed the functional enrichment analysis using DAVID 6.8 and identified the pathways of the extracellular matrix (ECM)-receptor interaction, protein digestion and absorption, focal adhesion and glycerophospholipid metabolism as significantly overrepresented (Table 1), which were reported to be relevant in pathogenesis of arthritis [21-24].

**Table 1:** Pathway analysis for candidate genes conferring susceptibility to RA

Pathway	P value	Genes
**Based on genes identified in comparison of RA patients and control**s		
**ECM-receptor interaction**	2.1 x 10^-3^	*COL4A4, COL6A5, COL11A1, COL11A2, HSPG2, ITGB5, LAMC1, THBS1*
**Protein digestion and absorption**	2.3 x 10^-3^	*ATP1A1, ATP1A4, COL4A4, COL6A5, COL11A1, COL11A2, MME, PRCP*
**Focal adhesion**	2.8 x 10^-2^	*RASGRF1, COL4A4, COL6A5, COL11A1, COL11A2, FLNB, ITGB5, LAMC1, MYL5, THBS1*
**Glycerophospholipid metabolism**	4.8 x 10^-2^	*CHAT, GPAT4, LPIN3, LPCAT1, MBOAT1, PTDSS2*
**Based on genes only identified in disease duration comparison of RA patients**		
**Olfactory transduction**	1.2 x 10^-2^	*OR14C36, OR4A15, OR52N4, OR6C74, OR6C75, OR7G3, OR9K2*

In order to identify variants that might predispose RA patients to disease duration, we repeated the variant filtration and gene burden analysis on Chinese RA samples with the disease duration ≥3-year compared to the disease duration ≤1-year. A total of 277 genes were identified (Supplementary Table 4) compared to the 381 genes identified in the case–control comparison (Supplementary Table 3). Of these, 87 genes were unique to disease duration with exonic variants (Supplementary Table 5). Pathway analysis performed on the 87 genes identified olfactory transduction pathway as significantly overrepresented (Table 1), including *OR14C36*, *OR4A15*, *OR52N4*, *OR6C74*, *OR6C75*, *OR7G3* and *OR9K2*.

### Structural analysis and function change prediction of mutant proteins

In order to gain structure insights of the protein mutation with pathogenic variants into the clinical conditions of RA patients, we derived a three-dimensionally structure model of SAA1 Gly90Asp (rs79681911) and SCOT1 Thr58Met (rs75134564) by combining homology modeling with point mutation in MOE 2015.09 package. The crystal structures of human SAA1 protein (PDB code: 4IP8.A) and SCOT1 protein (PDB code: 3K6M.C) were selected to be used as templates due to their optimal identity with the target sequences of SAA1 (Protein RefSeq: NP_000322.2) and SCOT1 (Protein RefSeq: NP_000427.1), 83.6% and 83.5%, respectively (Supplementary Figure 3). The SAA1 and SCOT1 models with the best packing quality function and full energy minimization were assessed by Ramachandran plots, indicating that the phi and psi backbone dihedral angles in the models were reasonable (Supplementary Figure 4).

**Figure 2:**
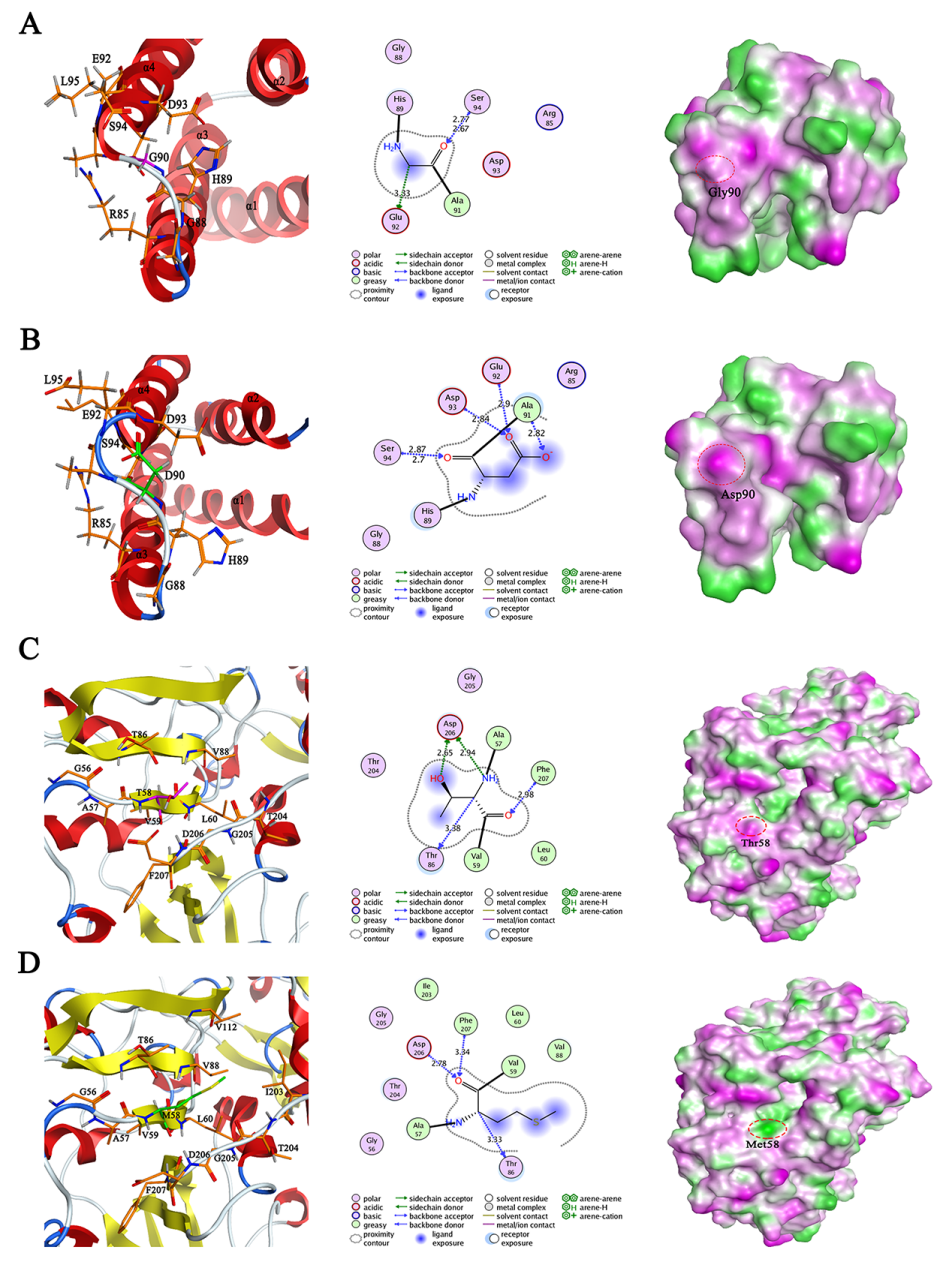
The modeled 3D structure comparison of human wild type SAA1 **(A)** and its mutant G90D **(B)**, as well as wild type SCOT1 **(C)** and its mutant T58M **(D)**. Left panel: the ribbon secondary structure diagram with α helices in red and β sheets in yellow; middle panel: the proposed interactions between mutated residue and its surrounding residues, distances are not represented to scale; right panel: the lipophilic surface representation by showing hydrophilic (magenta), neutral (green) and lipophilic (white).

Structural analysis of SAA1 Gly90Asp (Figure 2A and 2B) revealed that the substitution of glycine with aspartic acid induced the formation of two pairs of hydrogen bonds with two threonine residues (Ala91 and Asp93), exhibiting more stable structure of loop region and promoting the polar interaction. Moreover, this mutation shortened the length of α helix 4, which may affect the stability of SAA1. Structural changes in SAA1 protein caused the surface of Asp90 to be exposed in solvent environment, leading to the increased hydrophilic region. In addition, the construction of 3D models in SCOT1 and its mutant Thr58Met revealed that this substitution resulted in the disappearance of the hydrogen bond between Thr58 and Asp206, the reduction of intramolecular polar interactions and the expansion of hydrophobic region (Figure 2C and 2D), suggesting its potential function alteration in RA pathogenesis.

## DISCUSSION

In this study, we performed perspective WES aiming to identify potentially causal variants in a cohort of Chinese RA patients. We focused on investigating the occurrence frequency of variants in genes previously associated with RA as well as novel genes. We generated a previously reported GWAS candidate risk loci list in Supplementary Table 6, including Caucasians and Black Americans. It was initial surprising that our identified genes were not found in this list of GWAS data (group 1 and group 3 in Figure 1), such as HLA-associated genes. However, after reviewing the location of the reported RA-associated variants, we found that very few variants are located in the exome area, including *CTLA4* (rs231775), *FCGR2A* (rs1801274), *IL6R* (rs2228145), *OLIG3* (rs2230926), *PTPN22* (rs2476601), *RTKN2* (rs3125734), *SH2B3* (rs3184504), *TNFAIP3* (rs223092) and *TYK2* (rs34536443). Notably, most common variants identified by GWAS analysis are located in the non-coding region of the genome, while WES is a powerful technique for the identification of protein-coding variants, which are more straightforward to annotate biological functions and pinpoint causative genes. Our current work may be treated as a pilot study, and it is significantly valuable for our future works by expending samples of RA patients in Han Chinese population, even in comparison with different ethnicities.

Despite known variants of *TGFβ1* and *FOXP3* genes associated with increased RA risk [12-14], two novel risk variant loci in these two genes were for the first time identified to be implicated in the RA risk (group 4 and group 6 in Supplementary Table 3). A novel splicing variant (rs199982059) of *TGFβ1* was found to be significantly enriched in 4 RA patients, but absent in healthy controls. TGFβ1 is a pivotal protein in the pathogenesis of a number of autoimmune disorders and its dysregulation is also increasingly implicated in the risk of developing RA [25-27]. RNA splicing is a focal point on connection between genetic variations and complex disorders [28, 29], and this novel splicing variant of *TGFβ1* might provide new insights into the genetic determinants of RA disease. In addition, a novel missense variant (chrX:49114808) of *FOXP3* was observed in 8 RA patients. FOXP3 is a unique regulatory T cell (T_reg_)-specific marker and important in the development of RA-derived T_reg_ cells as a transcriptional factor [30, 31]. In spite of the other known variants in *TGFβ1* and *FOXP3* genes associated with RA, these two newly-identified variants in our Chinese RA patients may offer the novel genetic contributions to the RA risk.

We have also identified six novel and deleterious genes that are classified as pathogenic in ClinVar database (Supplementary Table 2). Of these, a missense variant (rs79681911) of *SAA1*, initially characterized by serum amyloid a variant (OMIM 104750) and required for the amyloidosis disease process, was identified in our RA patients. SAA1 has been reported to play a pathogenic role in the pro-inflammatory cascades in RA, therefore, this novel deleterious variant may be implicated in RA risk as a sensitive indicator of inflammatory activity [32]. Additional pathogenic variant (rs75134564) of *OXCT1* was predicted to be disease-related in 4 RA patients based on LR score, which previously implicated in Succinyl-CoA acetoacetate transferase deficiency (OMIM 601424) in clinic. *OXCT1* encoding enzyme SCOT1 is essential for ketone body metabolism and involved in cardiovascular disease, which are shown to be strongly associated with the course of RA [33-35], suggesting this enzyme may potentially contribute to RA prognosis. Importantly, the 3D structural analysis of these two mutants revealed that the substitution of mutation points may be involved in the functional alteration of the proteins and further impact on RA disease progression (Figure 2).

We sought to identify novel genes or biological candidate pathways fundamental to the risk of RA disease, including both rare and common variants. To elucidate additive effects of polygenic variants that affect the same gene or pathway, we performed gene burden test and pathway analysis. Notably, the biological impact of rare and deleterious variants is likely to be greater when present as two copies. In our study, 5 homozygous variants (group 5 in Figure 1; Supplementary Table 3) were detected in our RA patients but not in healthy controls. Intriguingly, a frameshift indel variant (rs61406813) of *NCR3LG1* (natural cytotoxicity triggering receptor 3 ligand 1) was identified in our RA patients as homozygote. NCR3LG1 could be detected on monocytes and neutrophils after application of inflammatory stimuli [36], and it was initially described as a tumor cell–expressed ligand of NKp30, which is found to be implicated in RA-associated inflammation [37]. Additionally, a missense variant (rs61014678) of *RAP1GAP* (RAP1 GTPase Activating Protein) was identified as damaging (disease-related, D) by determinant of LR model in our RA patients. RAP1GAP regulates the activity of the ras-related RAP1 protein, which involves in induction of apoptotic pathway in synovial fibroblasts and plays a critical role in oxidative stress and T cell behavior in RA synovial tissues [38, 39]. Thus, these two homozygous variants may perform stronger functions in RA pathogenic mechanisms.

Our WES analysis totally identified 381 genes that may partially contribute to RA pathogenesis and disease progression, including 3 genes (*TGFβ1*, *FOXP3* and *SAA1*) previously implicated in RA and 378 novel candidate genes. Biologic pathway analysis might help us to deeply understand RA pathogenesis, and previously biological pathways have been identified from genes in large-scale association analysis of GWAS data (Supplementary Table 6), such as autoimmune thyroid disease, natural killer cell mediated cytotoxicity and T cell receptor signaling pathways. We deciphered enrichment of our identified deleterious genes within additional pathways of ECM-receptor interaction, protein digestion and absorption, focal adhesion and glycerophospholipid metabolism based on our WES data (Table 1), which have been implicated in the autoimmune conditions or pathogenesis of RA [21-24]. We also sought to identify potential deleterious variants associated with disease duration among RA patients. Our pathway analysis focusing on variants enriched among RA patients with disease duration ≥3-year highlighted seven novel genes in olfactory transduction pathway (Table 1), which has been previously reported to be implicated in regulating inflammatory responses [40].

Pathogenesis of RA is complicated and includes both environmental and genetic factors. Recently, gut microbiota has been evident of being implicated in RA pathogenesis and treatment responses as a critical environmental factor that influences metabolic and immune homeostasis [41], involvement of protein digestion and absorption, glycerophospholipid metabolism and olfactory transduction pathway [42, 43], which were also enriched by novel candidate genes identified in our Chinese RA patients (Table 1). In addition, the homozygous variant *NCR3LG1* (group 5 in Supplementary Table 3) may mediate autoimmune and microbial infection-induced inflammation by associating with the ligand of NKp30 [37]. Therefore, these involved novel deleterious genes might be convincingly considered genetic contributions to microbial alteration in relation to the pathogenesis and development of RA.

Genetic factors on the X chromosome always contribute to the increased risk of developing autoimmune disorders in females compared with males, such as RA [44]. Here, four novel and deleterious variants were investigated to be associated with sex bias in 58 Chinese RA patients, including *OTC* (Ornithine Transcarbamylase) (rs72554348), *DIAPH2* (Diaphanous Related Formin 2) (rs363755), *ARSE* (Arylsulfatase E) (rs56393981) and *FOXP3* (chrX:49114808) (Supplementary Table 7). Notably, *OTC*, *ARSE* and *FOXP3* were previously reported to be implicated in x-linked diseases [45-49], in which these three novel variants identified in our study are also associated with female, supporting that the association of variants on X chromosome and RA may further provide molecular evidence as a risk factor contributing to increased susceptibility in Chinese female RA patients.

In summary, we have performed WES to present support and advance our understanding of associations with genetic variants that may be involved in the development of RA in the Chinese population. The variants highlighted include previously implicated genes as well as novel genes and pathways, involved in regulation of adaptive immune response, transmission of nerve impulse and chromosome organization. This study significantly extends the work of GWAS and provides new insight into fundamental etiologic mechanisms in this common autoimmune disease. While further experiments are required to validate our results and define the underlying biological mechanisms of these novel variants, these findings can serve as a starting point to elucidate the pathogenesis and potential impact on RA through genetics to functional insights.

## MATERIALS AND METHODS

### Patients

58 patients diagnosed as having RA were unrelated individuals of Han Chinese descent recruited from hospitals in Southern and Eastern China (Guangzhou and Changzhou) using 2010 Rheumatoid Arthritis Classification Criteria established by American College of Rheumatology and European League Against Rheumatism Collaborative Initiative (2010 ACR/EULAR) [50]. In addition, 66 healthy and unrelated blood donors of Han Chinese ancestry from Medical Center for Physical Examination and Health Assessment, were included as controls. Detailed descriptions of sequenced individuals and clinical characteristics of the enrolled patients are provided in Table 2 and 3. Written informed consent was obtained from all of the participants, and the study was registered in Chinese Clinical Trial Registry (ChiCTR-ROC-17010351) and approved by the local ethics committees of Macau University of Science and Technology (Macau, China).

**Table 2:** Description of the 124 sequenced individuals

Parameter	Cases	Controls
N	58	66
Sex	43 female, 15 male	41 female, 25 male
Age	48.48±14.08	35.23±10.73

**Table 3:** Demographic and clinical characteristics of the 58 patients with rheumatoid arthritis

Women/men, no. (%)	43(25.9%)/ 15(74.1%)
Age at diagnosis, mean±SD years	
All	45.62±14.19
Women	44.02±12.60
Men	50.20±17.68
Disease duration (years), mean±SD years	3.32±4.23
≤1-Year, no	26
≥3-Year, no	23
Rheumatoid factor +/-, no.(%)	45 (83.3%) / 9 (16.7%); 4 Not Test
No. of tender joints, mean±SD years	6.66±7.37
No. of swollen joints, mean±SD years	3.26±4.60

### Ancestry composition analysis

We verified the population ethnicity information of the RA and healthy control samples by ancestry composition analysis (Supplementary Figure 2) using admixture v1.3.0 [51] (https://www.genetics.ucla.edu/software/admixture) and multidimensional scaling in PLINK v1.07 (http://zzz.bwh.harvard.edu//plink/) [52]. Three ethnic populations were used as reference samples from 1000 Genome Project Phase III data (http://ftp.1000genomes.ebi.ac.uk/vol1/ftp/release/20130502/ALL.chr9.phase3_shapeit2_mvncall_integrated_v5a.20130502.genotypes.vcf.gz), including Utah Residents with Northern and Western European Ancestry (EUR-CEU), Yoruba in Ibadan, Nigeria (AFR-YRI) and Southern Han Chinese (EAS-CHS).

### Generation of candidate gene list

A list of 159 candidate genetic variants reported by previous studies with the P value threshold of P < 1 x 10^-5^ (Supplementary Table 6) was prepared based on Rheumatoid Arthritis associated genes in the NHGRI GWAS Catalog [53] and literatures [6, 7, 54, 55].

### Library preparation and WES

Blood samples were collected according to protocols approved by local institutional review boards. Genomic DNA was extracted from peripheral blood mononuclear cells (PBMCs) using PureLink® Genomic DNA Mini Kit (Invitrogen, USA) according to the manufacturer’s protocol. 500 ng of double-stranded DNA was determined by Qubit (Invitrogen, USA) and randomly fragmented to 150-200bp with Covaris cracker (Covaris, USA). Fragments with specific indexes were hybridized with probes. After PCR amplification and quality control, libraries were sequenced by next-generation sequencing. Agilent liquid phase hybridization was applied to efficiently enrich whole exons which would be sequenced on Illumina platform. Agilent SureSelect Human All ExonV5/V6 (Agilent Technologies, USA) with reagents were used for sequencing libraries and capture, which was recommended by the instruction manual and followed by optimized experimental procedures.

Sequencing was performed on an Illumina HiSeq X sequencer with a paired-end read length of 150bp in the Genomics Core Facility at Novogene (Genome Sequencing Company, Beijing, China). Data generated in this study will be submitted to the National Center for Biotechnology Information (NCBI) BioProject.

### WES data analysis

To analyze the entire cohort of samples for genotype calls, variant analysis and joint genotyping were performed according to the pipeline recommended by the Genome Analysis Toolkit software and the GATK Best Practices procedures on RA patients and healthy controls [56-59]. Briefly, Burrows-Wheeler Aligner (BWA) [56] software is utilized to align the raw sequencing reads in FASTQ formats to the 1000 Genomes (GRCh37 + decoy) human genome reference. The BWA alignment files were converted to BAM files with SAMtools v1.1 [57], which is used for sorting the BAM files. Duplicate reads were marked for BAM files with Picard MarkDuplicates (https://sourceforge.net/projects/picard/). The coverage and depth were computed based on the final BAM file. Local realignment, base quality recalibration, variant calling, joint genotyping, and variant quality score recalibration and filtration were applied using with GATK v3.7 (https://software.broadinstitute.org/gatk/). Default settings were used for BWA, SAMtools, Picard and GATK tools.

Further filtration for the joint genotyped variants was performed using Variant Tools [58]. We applied the following filters to generate a list of preliminary variants by removing false-positive variants through Variant Quality Score Recalibration with tranche truth sensitivity threshold <99.00, as well as variants with low read depth (DP) <10 and poor genotyping quality (GQ) <20, keeping exonic or splicing variants based on University of California, Santa Cruz (UCSC) genome browser build 37 human Reference Sequence Gene annotation, and removing synonymous variants. From the preliminary variant list, variants annotated as “pathogenic” in ClinVar and deleterious variants were identified, respectively, including those candidate genes that overlapped with previous studies or passed the case-control gene burden test threshold. Deleterious variants were predicted to be damaging (disease-related, D) or benign/neutral (tolerated, T) based on LR score determined by logistic regression (LR) model [59]. The novel deleterious variants were divided into the rare and common variant groups, which were distinguished by minor allele frequency (MAF) in Chinese Southern population from the 1000 Genomes Project phase III study.

### Analysis of burden association signal

Case–control gene burden analysis was assessed on both rare and common deleterious variants to investigate causal genes using RA patients with > 80% Chinese ancestry as cases and two types of controls: 105 southern Chinese samples from the 1000 Genomes Project phase III study and 66 healthy controls with >80% Chinese ancestry. Regardless of DP or GQ, all available genotype calls contributed to the number of allele count across the retained deleterious variants in each individual gene. The gene burden ratio was calculated by dividing the allele frequency in cases by the allele frequency in controls. We identified an enrichment of deleterious variants in a gene according to the gene burden ratio >1.5-fold with both types of controls, or the deleterious alleles in the gene with at least 3 RA cases if zero allele frequency in the controls. We further identified genes with rare variants that were homozygous in RA cases but not present in controls, which were considered greater contribution to functional impact.

### Pathway analysis

To discover enriched functional-related gene groups, pathway analysis was performed using DAVID Bioinformatics Resource 6.8 program (DAVID 6.8) (https://david.ncifcrf.gov/summary.jsp) with a Modified Fisher Exact P value less than 0.05 as the significance threshold and strong enrichment in the annotation categories.

### Structural analysis of proteins

Homology modeling is one of the best and reliable ways to construct the three dimensional (3D) structure of protein [60]. Firstly, protein sequence was imported into the Molecular Operating Environment (MOE) 2015.09 software (Chemical Computing Group Inc., Montreal, Canada) to search an optimal template. The top ranked structure based on the Z score towards the target sequence was selected as the template. Target protein sequence and its corresponding crystal structure coordinates of template were separately loaded and aligned. A series of protein models were independently constructed by using a Boltzmann-weighted randomized procedure [61]. Amber force field [62, 63] was applied in the process of construction and energy minimization. Finally, the model with the best packing quality function was selected for further full energy minimization, and the stereochemical qualities of protein model was assessed by means of Ramachandran plots.

To analyze the effect on the point mutation in the 3D structure of the protein, the mutant protein were carried out in Residue Scan module of MOE 2015.09 software based on the 3D structure of homology modeling. In addition, we further analyze the hydrogen bonds, solvent interactions, metal ligation and nonbonded interaction between the target mutant residue and its surrounding key amino acid residues.

## SUPPLEMENTARY MATERIALS FIGURES AND TABLES












