# Supplementary material for: Identification of potential genetic causal variants for rheumatoid arthritis by whole-exome sequencing
Source: Oncotarget. 2017 Nov 22;8(67):111119–29. doi: 10.18632/oncotarget.22630 (PMC5762310; doi:10.18632/oncotarget.22630)
Supplement: Supplementary file 1 [file oncotarget-08-111119-s001.pdf]

Identification of potential genetic causal variants for rheumatoid arthritis by whole-exome sequencing

SUPPLEMENTARY MATERIALS

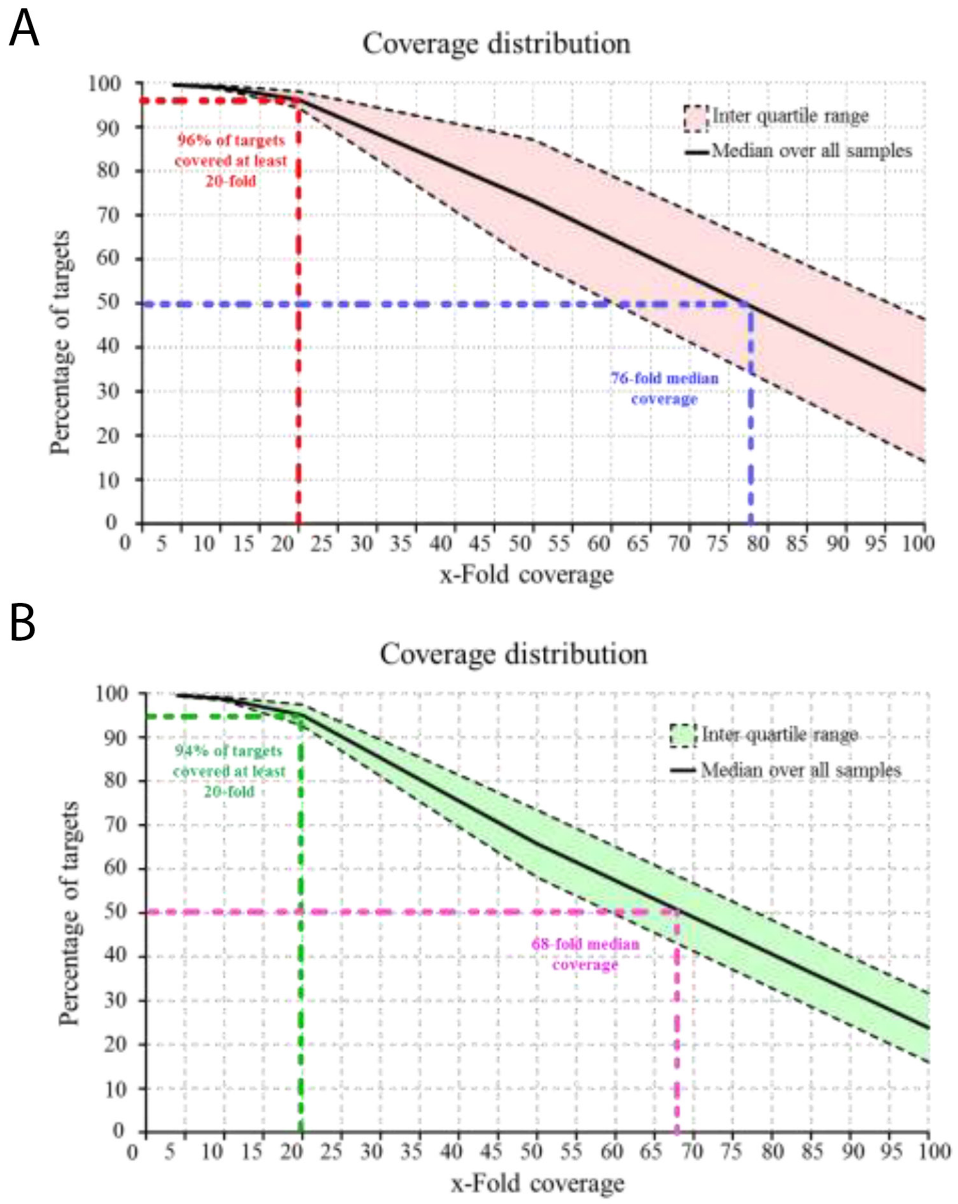

Supplementary Figure 1: Coverage distribution for all exons targeted by enrichment was evaluated by inter-quartile range calculation using SPSS 22.0 software. (A) The median coverage for all RA samples was 76-fold, with on average 96% of all targets covered at least 20-fold. **(B)** The median coverage for all healthy control samples was 68-fold, with on average 94% of all targets covered at least 20-fold.

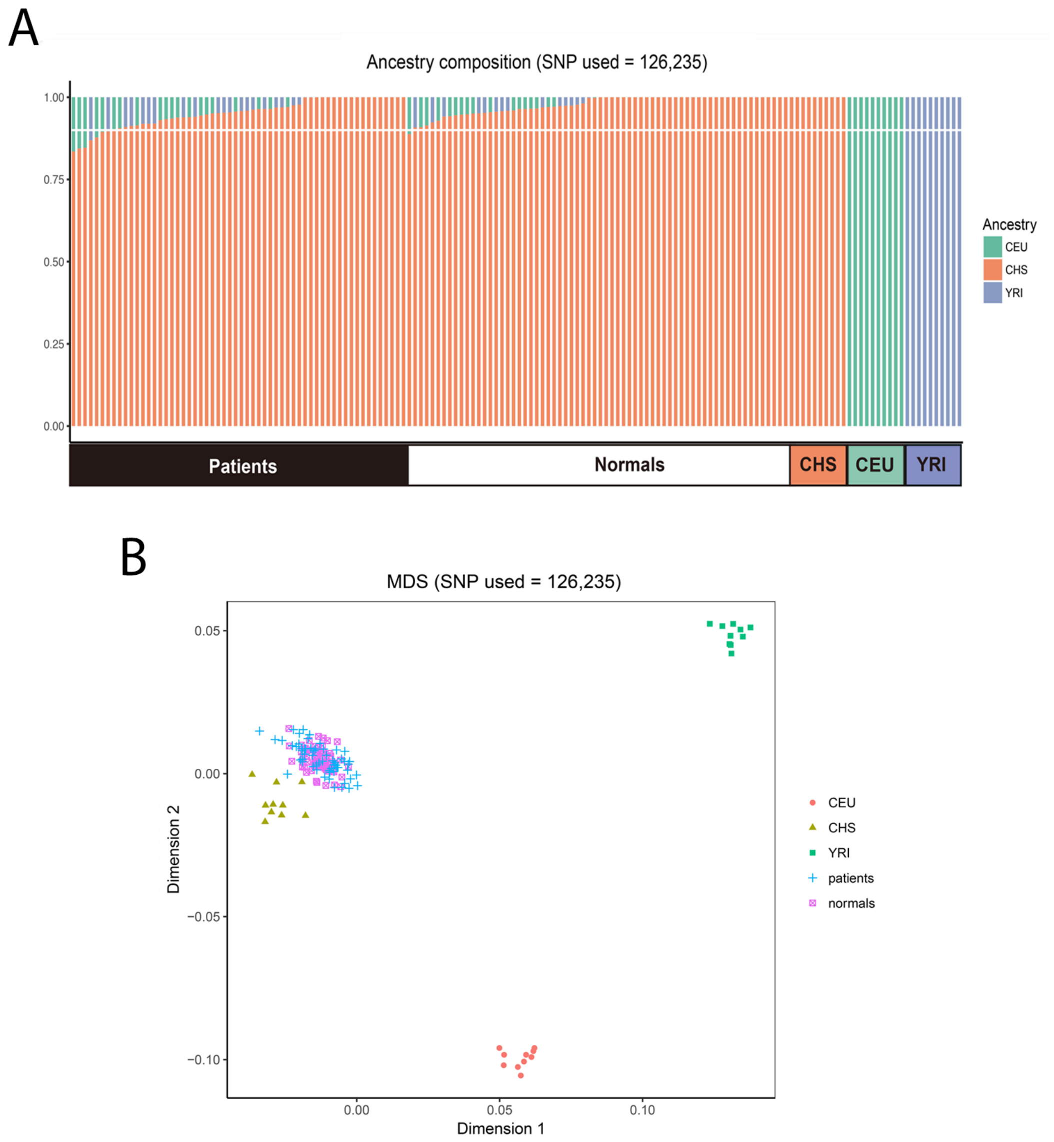

Supplementary Figure 2: Ancestral composition of RA and healthy control samples with Hapmap reference populations. **(A)** All of them had >80% Chinese ancestry (white horizontal line) by admixture analysis. **(B)** Dimension 1 and 2 of multidimensional scaling analysis for the RA and control samples. Patients, fifty-eight RA samples. Normals, sixty-six healthy control samples. Ten HapMap samples were randomly chosen from each of the three reference populations (CEU, CHS and YRI) and are shown in figures. CEU, Utah Residents with Northern and Western European Ancestry; CHS, Southern Han Chinese; YRI, Yoruba in Ibadan, Nigeria.

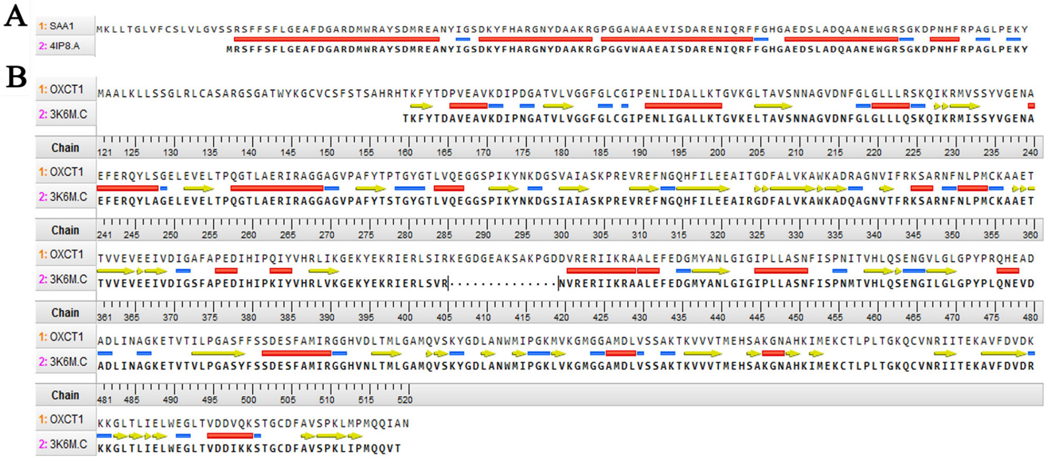

Supplementary Figure 3: Sequence alignment of target proteins **(A)** SAA1 (Protein RefSeq: NP_000322.2) and **(B)** SCOT1 (Protein RefSeq: NP_000427.1) with their template structures used in homolog modeling. Red line represents α helix, yellow arrow represents β sheet, and blue line represents loop region.

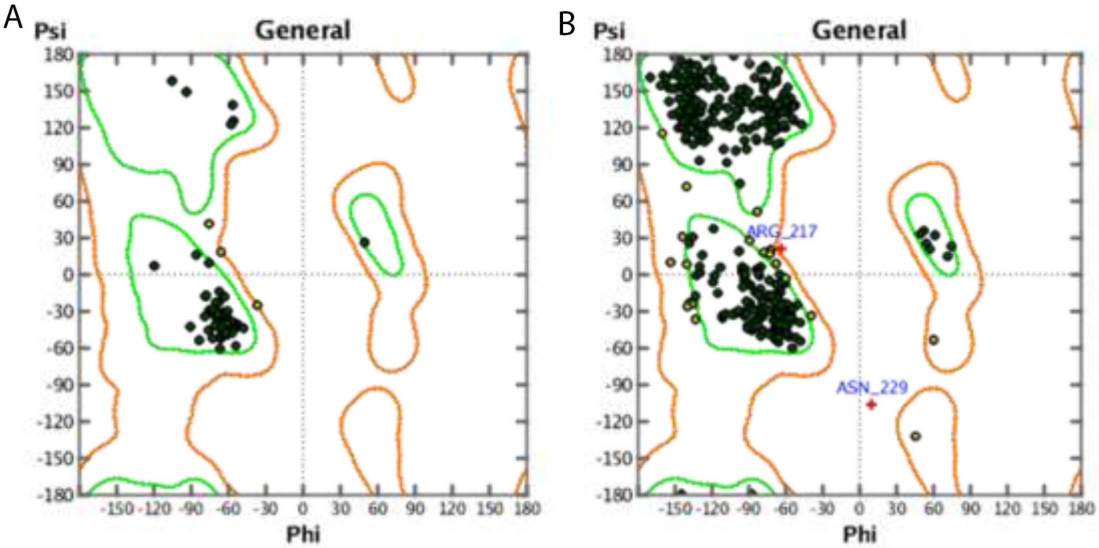

Supplementary Figure 4: Ramachandran plots for evaluation of protein model. **(A)** For SAA1, 99.03% of the residues are in the favored region and 0.97% are in the allowed region. **(B)** For SCOT1, 95.81% of the residues are in the favored region, 3.77% are in the allowed region and only 0.42% are in the disfavored region. Green represents favored region and light-brown represents allowed region.

Supplementary Table 1: Types of exonic, splicing and nonsynonymous variants (total =72,024)

Mutation type	No. of variants
Nonsynonymous SNV	56,466
Stop-gain	828
Stop-loss	49
Frameshift	408
Deletion<br>Insertion	215
Nonframeshift	705
Deletion<br>Insertion	466
Splicing	11,377
Unknown	1510

Supplementary Table 2: Clinical conditions associated with group 2 variants in RA and control comparison.

See Supplementary File 1

Supplementary Table 3: Candidate variant list from RA versus control comparison.

See Supplementary File 2

Supplementary Table 4: Candidate variant list from RA disease duration comparison.

See Supplementary File 3

Supplementary Table 5: Susceptibility genes unique to disease duration with exonic variants in Rheumatoid Arthritis disease

AMPD1	ATXN7	GPR84	OR7G3
PPARGC1B	C10orf142	OR9K2	CST9
ATP6V0A4	CH17-360D5.1	OR6C74	WDR33
HKDC1	NPY4R	OR6C75	DNAJC28
C1orf167	LRRC39	CATSPER4	DDTL
SLCO1B3	GPSM2	NR2C1	UPB1
MYH1	ITGA10	SERPINA10	LCLAT1
SGCA	LCE5A	RORA	CASR
RYR1	OR4A15	TPSG1	CDKN2AIP
KIZ	OR52N4	NEK8	FRYL
TPTE	SLAMF8	HEATR9	CWH43
LRP2	MTA2	PNMT	NMUR2
DYSF	EML3	ABCC3	GABRP
COL6A6	GPR161	NACA2	MDC1
CP	IL18BP	CACNG5	ENPP5
HTR3E	PANO1	AURKB	RAB19
TET2	LHX4	PLEKHG2	EPHB6
THBS2	LAD1	PSG8	GOT1L1
FLNC	SYT14	CBLC	HEMGN
SSPO	FAAP20	CARD8	ALG2
PKHD1L1	HIST4H4	TARM1	FTHL17
IL3RA	OR14C36	PRR36	

Supplementary Table 6: High priority candidate gene list in Rheumatoid Arthritis disease.

See Supplementary File 4

Supplementary Table 7: Comparison of X chromosome associated novel variants distribution between female and male in RA patients.

See Supplementary File 5
