# Supplementary material for: Identification of potential genetic causal variants for rheumatoid arthritis by whole-exome sequencing
Source: Oncotarget. 2017 Nov 22;8(67):111119–29. doi: 10.18632/oncotarget.22630 (PMC5762310; doi:10.18632/oncotarget.22630)
Supplement: Supplementary file 2 [file oncotarget-08-111119-s002.docx]

**Supplementary Table 2: Clinical conditions associated with group 2 variants in RA and control comparison.**

| chr | pos | id | ref | alt | gene | LR | Clinical conditions reported in ClinVar | Function |
| --- | --- | --- | --- | --- | --- | --- | --- | --- |
| 10 | 101829514 | rs61751507 | C | T | CPN1 | T | Anaphylotoxin inactivator deficiency | Peptide hormone metabolism; Protects the body from potent vasoactive and inflammatory peptides.. |
| 11 | 18291302 | rs79681911 | G | A | SAA1 | T | Serum amyloid a variant | Activated TLR4 signaling; Cytokine Signaling in Immune system. |
| 3 | 133476698 | rs41295774 | A | G | TF | T | Atransferrinemia | Vesicle-mediated transport; Iron metabolism in placenta. |
| 5 | 41862758 | rs75134564 | G | A | OXCT1 | D | Ornithine carbamoyltransferase deficiency | Ketone body metabolism; Regulation of lipid metabolism . |
| 7 | 44104839 | rs77938727 | C | T | PGAM2 | D | Gycogen storage disease type X | Glycosaminoglycan metabolism; Immune response in T lymphocytes. |
| X | 38229135 | rs72554348 | G | C | OTC | . | Ornithine carbamoyltransferase deficiency | Carbon metabolism; Viral mRNA Translation. |
