# Supplementary material for: Identification of potential genetic causal variants for rheumatoid arthritis by whole-exome sequencing
Source: Oncotarget. 2017 Nov 22;8(67):111119–29. doi: 10.18632/oncotarget.22630 (PMC5762310; doi:10.18632/oncotarget.22630)
Supplement: Supplementary file 3 [file oncotarget-08-111119-s003.docx]

**Supplementary Table 3: Candidate variant list from RA versus control comparison.**

| group | | chr | | Pos | | id | | ref | alt | | gene | | | | LR | | | **Gene burden ratio** | | | | | | **Total No. of alleles**  **in gene** | | | | | | **No. alt alleles**  **in genes** | | | | | | | | **No. of**  **cases with**  **alt alleles** | | | | | **Variant allele frequency** | | | |
| --- | --- | --- | --- | --- | --- | --- | --- | --- | --- | --- | --- | --- | --- | --- | --- | --- | --- | --- | --- | --- | --- | --- | --- | --- | --- | --- | --- | --- | --- | --- | --- | --- | --- | --- | --- | --- | --- | --- | --- | --- | --- | --- | --- | --- | --- | --- |
|  | |  | |  | |  | |  |  | |  | | | |  | | | KG  East Asia | | | healthy  Ctrl | | | healthy  Ctrl | | | RA | | | healthy Ctrl | | | | | RA | | |  | | | | | KG  East Asia | | | |
| 2 | | 10 | | 101829514 | | rs61751507 | | C | T | | CPN1 | | | | T | | | 1.81 | | | 1.52 | | | 132 | | | 116 | | | 3 | | | | | 4 | | | 4 | | | | | 0.0248 | | | |
| 2 | | 11 | | 18291302 | | rs79681911 | | G | A | | SAA1 | | | | T | | | 1.81 | | | . | | | 132 | | | 116 | | | 0 | | | | | 3 | | | 3 | | | | | 0.0198 | | | |
| 2 | | 3 | | 133476698 | | rs41295774 | | A | G | | TF | | | | T | | | 1.81 | | | 3.41 | | | 132 | | | 116 | | | 2 | | | | | 6 | | | 6 | | | | | 0.0238 | | | |
| 2 | | 5 | | 41862758 | | rs75134564 | | G | A | | OXCT1 | | | | D | | | 3.62 | | | 4.55 | | | 132 | | | 116 | | | 1 | | | | | 4 | | | 4 | | | | | 0.0069 | | | |
| 2 | | 7 | | 44104839 | | rs77938727 | | C | T | | PGAM2 | | | | D | | | 1.81 | | | 2.28 | | | 132 | | | 116 | | | 1 | | | | | 2 | | | 2 | | | | | 0.0129 | | | |
| 2 | | X | | 38229135 | | rs72554348 | | G | C | | OTC | | | | . | | | 2.79 | | | 2.32 | | | 132 | | | 114 | | | 3 | | | | | 6 | | | 5 | | | | | 0.0144 | | | |
| 4 | | 10 | | 102027318 | | rs200164003 | | G | C | | CWF19L1 | | | | . | | | 7.24 | | | . | | | 132 | | | 116 | | | 0 | | | | | 4 | | | 4 | | | | | 0.001 | | | |
| 4 | | 10 | | 114917776 | | rs191206106 | | C | G | | TCF7L2 | | | | . | | | 12.67 | | | 7.97 | | | 264 | | | 232 | | | 1 | | | | | 7 | | | 7 | | | | | 0.001 | | | |
| 4 | | 10 | | 114925441 | | rs138649767 | | G | A | | TCF7L2 | | | | D | | | 12.67 | | | 7.97 | | | 264 | | | 232 | | | 1 | | | | | 7 | | | 7 | | | | | 0.0089 | | | |
| 4 | | 10 | | 135086331 | | rs536126291 | | C | T | | ADAM8 | | | | . | | | 5.43 | | | 3.41 | | | 264 | | | 232 | | | 1 | | | | | 3 | | | 3 | | | | | 0.002 | | | |
| 4 | | 10 | | 135087305 | | rs3810960 | | G | A | | ADAM8 | | | | . | | | 5.43 | | | 3.41 | | | 264 | | | 232 | | | 1 | | | | | 3 | | | 3 | | | | | 0.003 | | | |
| 4 | | 10 | | 25144247 | | rs199794379 | | A | G | | PRTFDC1 | | | | D | | | 5.43 | | | . | | | 264 | | | 232 | | | 0 | | | | | 3 | | | 3 | | | | | 0.004 | | | |
| 4 | | 10 | | 25147326 | | rs199983667 | | C | A | | PRTFDC1 | | | | D | | | 5.43 | | | . | | | 264 | | | 232 | | | 0 | | | | | 3 | | | 3 | | | | | 0.001 | | | |
| 4 | | 10 | | 35894560 | | rs142589386 | | G | A | | GJD4 | | | | . | | | 5.43 | | | 3.41 | | | 396 | | | 348 | | | 1 | | | | | 3 | | | 3 | | | | | 0.003 | | | |
| 4 | | 10 | | 35897091 | | rs547212582 | | T | C | | GJD4 | | | | D | | | 5.43 | | | 3.41 | | | 396 | | | 348 | | | 1 | | | | | 3 | | | 3 | | | | | 0.002 | | | |
| 4 | | 10 | | 35897205 | | rs192362407 | | G | A | | GJD4 | | | | D | | | 5.43 | | | 3.41 | | | 396 | | | 348 | | | 1 | | | | | 3 | | | 3 | | | | | 0.004 | | | |
| 4 | | 10 | | 50854641 | | rs539884711 | | C | A | | CHAT | | | | D | | | 1.81 | | | 2.28 | | | 396 | | | 348 | | | 1 | | | | | 2 | | | 2 | | | | | 0.001 | | | |
| group | | chr | | Pos | | id | | ref | alt | | gene | | | | LR | | | **Gene burden ratio** | | | | | | **Total No. of alleles**  **in gene** | | | | | | **No. alt alleles**  **in genes** | | | | | | | | **No. of**  **cases with**  **alt alleles** | | | | | **Variant allele frequency** | | | |
|  | |  | |  | |  | |  |  | |  | | | |  | | | KG  East Asia | | | healthy  Ctrl | | | healthy  Ctrl | | | RA | | | healthy Ctrl | | | | | RA | | |  | | | | | KG  East Asia | | | |
| 4 | | 10 | | 50857631 | | rs201293521 | | G | T | | CHAT | | | | D | | | 1.81 | | | 2.28 | | | 396 | | | 348 | | | 1 | | | | | 2 | | | 2 | | | | | 0.001 | | | |
| 4 | | 10 | | 50870806 | | rs116628504 | | G | A | | CHAT | | | | D | | | 1.81 | | | 2.28 | | | 396 | | | 348 | | | 1 | | | | | 2 | | | 2 | | | | | 0.006 | | | |
| 4 | | 1 | | 100174455 | | rs192583899 | | T | C | | FRRS1 | | | | . | | | 1.81 | | | . | | | 264 | | | 232 | | | 0 | | | | | 3 | | | 3 | | | | | 0.003 | | | |
| 4 | | 1 | | 100177969 | | rs187278122 | | A | G | | FRRS1 | | | | . | | | 1.81 | | | . | | | 264 | | | 232 | | | 0 | | | | | 3 | | | 3 | | | | | 0.006 | | | |
| 4 | | 1 | | 103444283 | | rs192842970 | | G | A | | COL11A1 | | | | D | | | 10.86 | | | 3.4 | | | 394 | | | 348 | | | 2 | | | | | 6 | | | 6 | | | | | 0.004 | | | |
| 4 | | 1 | | 103468295 | | rs2622875 | | C | T | | COL11A1 | | | | . | | | 10.86 | | | 3.4 | | | 394 | | | 348 | | | 2 | | | | | 6 | | | 6 | | | | | 0.001 | | | |
| 4 | | 1 | | 103544434 | | rs12136865 | | A | G | | COL11A1 | | | | . | | | 10.86 | | | 3.4 | | | 394 | | | 348 | | | 2 | | | | | 6 | | | 6 | | | | | 0.001 | | | |
| 4 | | 11 | | 111857624 | | . | | T | C | | DIXDC1 | | | | . | | | 5.43 | | | 1.71 | | | 264 | | | 232 | | | 2 | | | | | 3 | | | 3 | | | | | 0.002 | | | |
| 4 | | 11 | | 111889694 | | . | | T | C | | DIXDC1 | | | | . | | | 5.43 | | | 1.71 | | | 264 | | | 232 | | | 2 | | | | | 3 | | | 3 | | | | | 0.001 | | | |
| 4 | | 11 | | 121426036 | | rs140499624 | | A | G | | SORL1 | | | | . | | | 7.24 | | | 2.28 | | | 132 | | | 116 | | | 1 | | | | | 2 | | | 2 | | | | | 0.004 | | | |
| 4 | | 11 | | 124006828 | | rs2276055 | | C | T | | VWA5A | | | | . | | | 7.24 | | | . | | | 132 | | | 116 | | | 0 | | | | | 4 | | | 4 | | | | | 0.0079 | | | |
| 4 | | 11 | | 124742777 | | rs117828759 | | C | T | | ROBO3 | | | | . | | | 4.95 | | | 2.01 | | | 128 | | | 106 | | | 3 | | | | | 5 | | | 5 | | | | | 0.0079 | | | |
| 4 | | 11 | | 2432720 | | rs548094761 | | C | T | | TRPM5 | | | | D | | | 3.62 | | | 6.83 | | | 396 | | | 348 | | | 1 | | | | | 6 | | | 6 | | | | | 0.003 | | | |
| 4 | | 11 | | 2434772 | | rs74570003 | | G | T | | TRPM5 | | | | D | | | 3.62 | | | 6.83 | | | 396 | | | 348 | | | 1 | | | | | 6 | | | 6 | | | | | 0.003 | | | |
| 4 | | 11 | | 2441443 | | rs201184691 | | G | A | | TRPM5 | | | | . | | | 3.62 | | | 6.83 | | | 396 | | | 348 | | | 1 | | | | | 6 | | | 6 | | | | | 0.004 | | | |
| 4 | | 1 | | 1267086 | | rs201655269 | | C | T | | TAS1R3 | | | | D | | | 7.24 | | | 4.55 | | | 396 | | | 348 | | | 1 | | | | | 4 | | | 4 | | | | | 0.001 | | | |
| 4 | | 1 | | 1268119 | | rs548456115 | | C | T | | TAS1R3 | | | | D | | | 7.24 | | | 4.55 | | | 396 | | | 348 | | | 1 | | | | | 4 | | | 4 | | | | | 0.001 | | | |
| 4 | | 1 | | 1269146 | | rs576045705 | | G | A | | TAS1R3 | | | | D | | | 7.24 | | | 4.55 | | | 396 | | | 348 | | | 1 | | | | | 4 | | | 4 | | | | | 0.001 | | | |
| group | | chr | | Pos | | id | | ref | alt | | gene | | | | LR | | | **Gene burden ratio** | | | | | | **Total No. of alleles**  **in gene** | | | | | | **No. alt alleles**  **in genes** | | | | | | | | **No. of**  **cases with**  **alt alleles** | | | | | **Variant allele frequency** | | | |
|  | |  | |  | |  | |  |  | |  | | | |  | | | KG  East Asia | | | healthy  Ctrl | | | healthy  Ctrl | | | RA | | | healthy Ctrl | | | | | RA | | |  | | | | | KG  East Asia | | | |
| 4 | | 11 | | 2909699 | | rs80153297 | | C | T | | SLC22A18AS | | | | . | | | 2.41 | | | . | | | 264 | | | 232 | | | 0 | | | | | 4 | | | 4 | | | | | 0.005 | | | |
| 4 | | 11 | | 2920835 | | rs189560463 | | G | C | | SLC22A18AS | | | | . | | | 2.41 | | | . | | | 264 | | | 232 | | | 0 | | | | | 4 | | | 4 | | | | | 0.002 | | | |
| 4 | | 1 | | 146759364 | | . | | C | T | | CHD1L | | | | . | | | 5.62 | | | 3.54 | | | 132 | | | 112 | | | 1 | | | | | 3 | | | 3 | | | | | 0.001 | | | |
| 4 | | 11 | | 479160 | | rs2301168 | | G | T | | PTDSS2 | | | | . | | | 3.62 | | | 4.55 | | | 264 | | | 232 | | | 1 | | | | | 4 | | | 4 | | | | | 0.003 | | | |
| 4 | | 11 | | 490416 | | rs200466000 | | C | T | | PTDSS2 | | | | . | | | 3.62 | | | 4.55 | | | 264 | | | 232 | | | 1 | | | | | 4 | | | 4 | | | | | 0.004 | | | |
| 4 | | 1 | | 151958735 | | rs190916122 | | A | G | | S100A10 | | | | . | | | 5.43 | | | 1.68 | | | 130 | | | 116 | | | 2 | | | | | 3 | | | 3 | | | | | 0.005 | | | |
| 4 | | 11 | | 56756398 | | rs13343184 | | G | T | | OR5AK2 | | | | . | | | 2.72 | | | 3.41 | | | 264 | | | 232 | | | 1 | | | | | 3 | | | 3 | | | | | 0.002 | | | |
| 4 | | 11 | | 56756578 | | rs189588796 | | C | T | | OR5AK2 | | | | . | | | 2.72 | | | 3.41 | | | 264 | | | 232 | | | 1 | | | | | 3 | | | 3 | | | | | 0.002 | | | |
| 4 | | 1 | | 160121885 | | rs146860131 | | C | T | | ATP1A4 | | | | . | | | 10.86 | | | 3.41 | | | 528 | | | 464 | | | 2 | | | | | 6 | | | 6 | | | | | 0.003 | | | |
| 4 | | 1 | | 160143514 | | rs45441496 | | T | G | | ATP1A4 | | | | . | | | 10.86 | | | 3.41 | | | 528 | | | 464 | | | 2 | | | | | 6 | | | 6 | | | | | 0.0089 | | | |
| 4 | | 1 | | 160143945 | | rs199962758 | | T | C | | ATP1A4 | | | | D | | | 10.86 | | | 3.41 | | | 528 | | | 464 | | | 2 | | | | | 6 | | | 6 | | | | | 0.003 | | | |
| 4 | | 1 | | 160144538 | | rs185461260 | | G | T | | ATP1A4 | | | | . | | | 10.86 | | | 3.41 | | | 528 | | | 464 | | | 2 | | | | | 6 | | | 6 | | | | | 0.004 | | | |
| 4 | | 1 | | 161090011 | | rs201971149 | | G | C | | NIT1 | | | | D | | | 3.62 | | | 2.28 | | | 132 | | | 116 | | | 1 | | | | | 2 | | | 2 | | | | | 0.001 | | | |
| 4 | | 11 | | 63400493 | | rs17656941 | | C | T | | ATL3 | | | | . | | | 3.62 | | | 2.28 | | | 264 | | | 232 | | | 1 | | | | | 2 | | | 2 | | | | | 0.003 | | | |
| 4 | | 11 | | 63403670 | | rs3781606 | | C | T | | ATL3 | | | | . | | | 3.62 | | | 2.28 | | | 264 | | | 232 | | | 1 | | | | | 2 | | | 2 | | | | | 0.0079 | | | |
| 4 | | 11 | | 6585361 | | rs113574909 | | A | G | | DNHD1 | | | | . | | | 3.62 | | | 2.28 | | | 132 | | | 116 | | | 1 | | | | | 2 | | | 2 | | | | | 0.0099 | | | |
| 4 | | 11 | | 67786664 | | rs535638253 | | G | A | | ALDH3B1 | | | | . | | | 1.81 | | | 2.84 | | | 528 | | | 464 | | | 2 | | | | | 5 | | | 5 | | | | | 0.001 | | | |
| 4 | | 11 | | 67789111 | | rs371833737 | | C | T | | ALDH3B1 | | | | . | | | 1.81 | | | 2.84 | | | 528 | | | 464 | | | 2 | | | | | 5 | | | 5 | | | | | 0.003 | | | |
| group | | chr | | Pos | | id | | ref | alt | | gene | | | | LR | | | **Gene burden ratio** | | | | | | **Total No. of alleles**  **in gene** | | | | | | **No. alt alleles**  **in genes** | | | | | | | | **No. of**  **cases with**  **alt alleles** | | | | | **Variant allele frequency** | | | |
|  | |  | |  | |  | |  |  | |  | | | |  | | | KG  East Asia | | | healthy  Ctrl | | | healthy  Ctrl | | | RA | | | healthy Ctrl | | | | | RA | | |  | | | | | KG  East Asia | | | |
| 4 | | 11 | | 67789277 | | rs370461081 | | G | A | | ALDH3B1 | | | | . | | | 1.81 | | | 2.84 | | | 528 | | | 464 | | | 2 | | | | | 5 | | | 5 | | | | | 0.002 | | | |
| 4 | | 11 | | 67795344 | | rs374814356 | | G | A | | ALDH3B1 | | | | . | | | 1.81 | | | 2.84 | | | 528 | | | 464 | | | 2 | | | | | 5 | | | 5 | | | | | 0.005 | | | |
| 4 | | 11 | | 74716666 | | rs202090872 | | G | C | | NEU3 | | | | D | | | 5.43 | | | 3.41 | | | 396 | | | 348 | | | 1 | | | | | 3 | | | 3 | | | | | 0.003 | | | |
| 4 | | 11 | | 74716935 | | rs200629627 | | G | A | | NEU3 | | | | D | | | 5.43 | | | 3.41 | | | 396 | | | 348 | | | 1 | | | | | 3 | | | 3 | | | | | 0.003 | | | |
| 4 | | 11 | | 74717001 | | rs539514716 | | C | T | | NEU3 | | | | D | | | 5.43 | | | 3.41 | | | 396 | | | 348 | | | 1 | | | | | 3 | | | 3 | | | | | 0.002 | | | |
| 4 | | 1 | | 176853472 | | rs79630456 | | C | T | | ASTN1 | | | | . | | | 2.72 | | | 1.71 | | | 132 | | | 116 | | | 2 | | | | | 3 | | | 3 | | | | | 0.0089 | | | |
| 4 | | 11 | | 76873966 | | . | | C | T | | MYO7A | | | | D | | | 3.62 | | | 2.28 | | | 264 | | | 232 | | | 1 | | | | | 2 | | | 2 | | | | | 0.001 | | | |
| 4 | | 11 | | 76900487 | | . | | G | C | | MYO7A | | | | D | | | 3.62 | | | 2.28 | | | 264 | | | 232 | | | 1 | | | | | 2 | | | 2 | | | | | 0.004 | | | |
| 4 | | 1 | | 17949680 | | rs75914589 | | C | T | | ARHGEF10L | | | | . | | | 3.62 | | | 2.28 | | | 132 | | | 116 | | | 1 | | | | | 2 | | | 2 | | | | | 0.0089 | | | |
| 4 | | 11 | | 82536079 | | rs202237309 | | C | T | | PRCP | | | | D | | | 3.62 | | | 2.28 | | | 264 | | | 232 | | | 1 | | | | | 2 | | | 2 | | | | | 0.004 | | | |
| 4 | | 11 | | 82549453 | | rs536616254 | | C | T | | PRCP | | | | D | | | 3.62 | | | 2.28 | | | 264 | | | 232 | | | 1 | | | | | 2 | | | 2 | | | | | 0.001 | | | |
| 4 | | 11 | | 82644887 | | rs553247583 | | G | GC | | DDIAS | | | | . | | | 1.81 | | | 2.28 | | | 132 | | | 116 | | | 1 | | | | | 2 | | | 2 | | | | | 0.006 | | | |
| 4 | | 1 | | 183084778 | | rs200671087 | | A | G | | LAMC1 | | | | . | | | 3.62 | | | 2.28 | | | 264 | | | 232 | | | 1 | | | | | 2 | | | 2 | | | | | 0.002 | | | |
| 4 | | 1 | | 183086586 | | rs544527088 | | G | A | | LAMC1 | | | | . | | | 3.62 | | | 2.28 | | | 264 | | | 232 | | | 1 | | | | | 2 | | | 2 | | | | | 0.001 | | | |
| 4 | | 11 | | 8959479 | | rs147479456 | | G | A | | ASCL3 | | | | D | | | 3.62 | | | . | | | 264 | | | 232 | | | 0 | | | | | 4 | | | 4 | | | | | 0.001 | | | |
| 4 | | 11 | | 8959607 | | rs201803232 | | C | T | | ASCL3 | | | | D | | | 3.62 | | | . | | | 264 | | | 232 | | | 0 | | | | | 4 | | | 4 | | | | | 0.003 | | | |
| 4 | | 11 | | 93754643 | | rs192979315 | | T | A | | HEPHL1 | | | | D | | | 5.43 | | | 6.83 | | | 528 | | | 464 | | | 1 | | | | | 6 | | | 6 | | | | | 0.002 | | | |
| 4 | | 11 | | 93754667 | | rs564600266 | | A | G | | HEPHL1 | | | | D | | | 5.43 | | | 6.83 | | | 528 | | | 464 | | | 1 | | | | | 6 | | | 6 | | | | | 0.005 | | | |
| group | | chr | | Pos | | id | | ref | | alt | | gene | | | | LR | | | **Gene burden ratio** | | | | | | **Total No. of alleles**  **in gene** | | | | | | | **No. alt alleles**  **in genes** | | | | | | | | **No. of**  **cases with**  **alt alleles** | | | | **Variant allele frequency** | | |
|  | |  | |  | |  | |  | |  | |  | | | |  | | | KG  East Asia | | | healthy  Ctrl | | | healthy  Ctrl | | | RA | | | healthy Ctrl | | | | | RA | | |  | | | | KG  East Asia | | | |
| 4 | | 11 | | 93779075 | | rs151306000 | | A | | T | | HEPHL1 | | | | D | | | 5.43 | | | 6.83 | | | 528 | | | 464 | | | 1 | | | | | 6 | | | 6 | | | | 0.0069 | | | |
| 4 | | 11 | | 93797581 | | rs146491431 | | G | | C | | HEPHL1 | | | | D | | | 5.43 | | | 6.83 | | | 528 | | | 464 | | | 1 | | | | | 6 | | | 6 | | | | 0.002 | | | |
| 4 | | 1 | | 19566783 | | rs201918168 | | C | | T | | EMC1 | | | | . | | | 1.81 | | | 3.41 | | | 132 | | | 116 | | | 1 | | | | | 3 | | | 3 | | | | 0.0079 | | | |
| 4 | | 1 | | 197070906 | | rs118010078 | | C | | T | | ASPM | | | | D | | | 3.62 | | | 2.28 | | | 264 | | | 232 | | | 2 | | | | | 4 | | | 4 | | | | 0.0069 | | | |
| 4 | | 1 | | 197072871 | | rs144969324 | | C | | T | | ASPM | | | | D | | | 3.62 | | | 2.28 | | | 264 | | | 232 | | | 2 | | | | | 4 | | | 4 | | | | 0.005 | | | |
| 4 | | 1 | | 203195004 | | rs190551025 | | C | | T | | CHIT1 | | | | . | | | 3.62 | | | 4.55 | | | 264 | | | 232 | | | 1 | | | | | 4 | | | 4 | | | | 0.006 | | | |
| 4 | | 1 | | 203198732 | | rs16851144 | | C | | T | | CHIT1 | | | | . | | | 3.62 | | | 4.55 | | | 264 | | | 232 | | | 1 | | | | | 4 | | | 4 | | | | 0.001 | | | |
| 4 | | 12 | | 122247945 | | rs375525174 | | G | | T | | SETD1B | | | | D | | | 7.24 | | | 1.52 | | | 264 | | | 232 | | | 3 | | | | | 4 | | | 4 | | | | 0.002 | | | |
| 4 | | 12 | | 122261544 | | rs553963413 | | G | | A | | SETD1B | | | | D | | | 7.24 | | | 1.52 | | | 264 | | | 232 | | | 3 | | | | | 4 | | | 4 | | | | 0.002 | | | |
| 4 | | 12 | | 12672941 | | rs187382315 | | T | | G | | DUSP16 | | | | . | | | 7.24 | | | 2.24 | | | 130 | | | 116 | | | 2 | | | | | 4 | | | 4 | | | | 0.005 | | | |
| 4 | | 1 | | 21926031 | | rs138045307 | | C | | T | | RAP1GAP | | | | D | | | 7.24 | | | 4.55 | | | 264 | | | 232 | | | 2 | | | | | 8 | | | 7 | | | | 0.001 | | | |
| 4 | | 1 | | 22149838 | | rs554059442 | | C | | T | | HSPG2 | | | | D | | | 2.72 | | | . | | | 396 | | | 348 | | | 0 | | | | | 3 | | | 3 | | | | 0.002 | | | |
| 4 | | 1 | | 22157470 | | rs77527456 | | C | | T | | HSPG2 | | | | . | | | 2.72 | | | . | | | 396 | | | 348 | | | 0 | | | | | 3 | | | 3 | | | | 0.0079 | | | |
| 4 | | 1 | | 22157544 | | rs368497178 | | C | | T | | HSPG2 | | | | D | | | 2.72 | | | . | | | 396 | | | 348 | | | 0 | | | | | 3 | | | 3 | | | | 0.001 | | | |
| 4 | | 1 | | 225599137 | | rs192884088 | | C | | T | | LBR | | | | D | | | 3.64 | | | 4.55 | | | 526 | | | 462 | | | 1 | | | | | 4 | | | 4 | | | | 0.001 | | | |
| 4 | | 1 | | 225600276 | | rs145104817 | | C | | T | | LBR | | | | D | | | 3.64 | | | 4.55 | | | 526 | | | 462 | | | 1 | | | | | 4 | | | 4 | | | | 0.002 | | | |
| 4 | | 1 | | 225607507 | | rs144956313 | | T | | A | | LBR | | | | . | | | 3.64 | | | 4.55 | | | 526 | | | 462 | | | 1 | | | | | 4 | | | 4 | | | | 0.001 | | | |
| 4 | | 1 | | 225611800 | | rs375540112 | | T | | C | | LBR | | | | . | | | 3.64 | | | 4.55 | | | 526 | | | 462 | | | 1 | | | | | 4 | | | 4 | | | | 0.002 | | | |
| group | | chr | | Pos | | id | | ref | | alt | | gene | | | | LR | | | **Gene burden ratio** | | | | | | **Total No. of alleles**  **in gene** | | | | | | | **No. alt alleles**  **in genes** | | | | | | | | **No. of**  **cases with**  **alt alleles** | | | | **Variant allele frequency** | | |
|  | |  | |  | |  | |  | |  | |  | | | |  | | | KG  East Asia | | | healthy  Ctrl | | | healthy  Ctrl | | | RA | | | healthy Ctrl | | | | | RA | | |  | | | | KG  East Asia | | | |
| 4 | | 1 | | 228109706 | | rs146233417 | | C | | A | | WNT9A | | | | . | | | 5.43 | | | 3.41 | | | 132 | | | 116 | | | 1 | | | | | 3 | | | 3 | | | | 0.005 | | | |
| 4 | | 1 | | 23281822 | | rs543523784 | | C | | T | | LACTBL1 | | | | . | | | 3.62 | | | 2.28 | | | 528 | | | 464 | | | 2 | | | | | 4 | | | 4 | | | | 0.001 | | | |
| 4 | | 1 | | 23285238 | | rs374793392 | | C | | T | | LACTBL1 | | | | . | | | 3.62 | | | 2.28 | | | 528 | | | 464 | | | 2 | | | | | 4 | | | 4 | | | | 0.004 | | | |
| 4 | | 1 | | 23285418 | | rs186492394 | | A | | G | | LACTBL1 | | | | . | | | 3.62 | | | 2.28 | | | 528 | | | 464 | | | 2 | | | | | 4 | | | 4 | | | | 0.003 | | | |
| 4 | | 1 | | 23289623 | | rs557724162 | | C | | T | | LACTBL1 | | | | . | | | 3.62 | | | 2.28 | | | 528 | | | 464 | | | 2 | | | | | 4 | | | 4 | | | | 0.001 | | | |
| 4 | | 1 | | 241850753 | | rs146579868 | | T | | C | | WDR64 | | | | . | | | 7.24 | | | 2.28 | | | 264 | | | 232 | | | 2 | | | | | 4 | | | 4 | | | | 0.002 | | | |
| 4 | | 1 | | 241929510 | | rs576824811 | | C | | T | | WDR64 | | | | . | | | 7.24 | | | 2.28 | | | 264 | | | 232 | | | 2 | | | | | 4 | | | 4 | | | | 0.001 | | | |
| 4 | | 12 | | 52822463 | | rs192170292 | | A | | T | | KRT75 | | | | D | | | 1.81 | | | 2.28 | | | 264 | | | 232 | | | 1 | | | | | 2 | | | 2 | | | | 0.002 | | | |
| 4 | | 12 | | 52825806 | | rs548132126 | | A | | G | | KRT75 | | | | D | | | 1.81 | | | 2.28 | | | 264 | | | 232 | | | 1 | | | | | 2 | | | 2 | | | | 0.001 | | | |
| 4 | | 12 | | 52908938 | | rs375140289 | | C | | T | | KRT5 | | | | D | | | 5.43 | | | 1.71 | | | 264 | | | 232 | | | 2 | | | | | 3 | | | 3 | | | | 0.002 | | | |
| 4 | | 12 | | 52913517 | | rs638907 | | G | | A | | KRT5 | | | | . | | | 5.43 | | | 1.71 | | | 264 | | | 232 | | | 2 | | | | | 3 | | | 3 | | | | 0.0079 | | | |
| 4 | | 1 | | 27875517 | | rs111827498 | | AAGG | | A | | AHDC1 | | | | . | | | 7.24 | | | . | | | 132 | | | 116 | | | 0 | | | | | 4 | | | 4 | | | | 0.004 | | | |
| 4 | | 12 | | 94613786 | | rs117324576 | | G | | A | | PLXNC1 | | | | . | | | 9.05 | | | 1.9 | | | 132 | | | 116 | | | 3 | | | | | 5 | | | 5 | | | | 0.0099 | | | |
| 4 | | 12 | | 99478699 | | rs370166322 | | T | | C | | ANKS1B | | | | . | | | 3.85 | | | 2.22 | | | 242 | | | 218 | | | 1 | | | | | 2 | | | 2 | | | | 0.004 | | | |
| 4 | | 12 | | 99640652 | | rs74712860 | | G | | A | | ANKS1B | | | | . | | | 3.85 | | | 2.22 | | | 242 | | | 218 | | | 1 | | | | | 2 | | | 2 | | | | 0.0079 | | | |
| 4 | | 13 | | 103392313 | | rs372526941 | | C | | G | | CCDC168 | | | | . | | | 2.72 | | | 1.71 | | | 396 | | | 348 | | | 2 | | | | | 3 | | | 3 | | | | 0.003 | | | |
| 4 | | 13 | | 103396838 | | rs183822515 | | A | | G | | CCDC168 | | | | . | | | 2.72 | | | 1.71 | | | 396 | | | 348 | | | 2 | | | | | 3 | | | 3 | | | | 0.006 | | | |
| 4 | | 13 | | 103397088 | | rs192109030 | | C | | T | | CCDC168 | | | | . | | | 2.72 | | | 1.71 | | | 396 | | | 348 | | | 2 | | | | | 3 | | | 3 | | | | 0.001 | | | |
| group | | chr | | Pos | | id | | ref | | alt | | gene | | | | LR | | | **Gene burden ratio** | | | | | | **Total No. of alleles**  **in gene** | | | | | | | **No. alt alleles**  **in genes** | | | | | | | | **No. of**  **cases with**  **alt alleles** | | | | **Variant allele frequency** | | |
|  | |  | |  | |  | |  | |  | |  | | | |  | | | KG  East Asia | | | healthy  Ctrl | | | healthy  Ctrl | | | RA | | | healthy Ctrl | | | | | RA | | |  | | | | KG  East Asia | | | |
| 4 | | 13 | | 106142185 | | rs113341591 | | C | | T | | DAOA | | | | . | | | 6.34 | | | 2.66 | | | 264 | | | 232 | | | 3 | | | | | 7 | | | 7 | | | | 0.0069 | | | |
| 4 | | 13 | | 106142236 | | rs111916808 | | G | | A | | DAOA | | | | . | | | 6.34 | | | 2.66 | | | 264 | | | 232 | | | 3 | | | | | 7 | | | 7 | | | | 0.0099 | | | |
| 4 | | 1 | | 35226964 | | rs146378222 | | G | | A | | GJB4 | | | | D | | | 3.02 | | | 1.9 | | | 528 | | | 464 | | | 3 | | | | | 5 | | | 5 | | | | 0.006 | | | |
| 4 | | 1 | | 35227225 | | rs373126632 | | C | | T | | GJB4 | | | | D | | | 3.02 | | | 1.9 | | | 528 | | | 464 | | | 3 | | | | | 5 | | | 5 | | | | 0.001 | | | |
| 4 | | 1 | | 35227231 | | rs146979528 | | G | | A | | GJB4 | | | | D | | | 3.02 | | | 1.9 | | | 528 | | | 464 | | | 3 | | | | | 5 | | | 5 | | | | 0.002 | | | |
| 4 | | 1 | | 35227585 | | rs201000959 | | C | | T | | GJB4 | | | | D | | | 3.02 | | | 1.9 | | | 528 | | | 464 | | | 3 | | | | | 5 | | | 5 | | | | 0.003 | | | |
| 4 | | 1 | | 35350573 | | rs34565935 | | CT | | C | | DLGAP3 | | | | . | | | 1.81 | | | 2.28 | | | 132 | | | 116 | | | 1 | | | | | 2 | | | 2 | | | | 0.006 | | | |
| 4 | | 1 | | 36056256 | | rs114404250 | | G | | A | | TFAP2E | | | | D | | | 2.72 | | | 3.41 | | | 132 | | | 116 | | | 1 | | | | | 3 | | | 3 | | | | 0.0069 | | | |
| 4 | | 13 | | 76378421 | | rs190172966 | | A | | T | | LMO7 | | | | . | | | 3.62 | | | . | | | 396 | | | 348 | | | 0 | | | | | 4 | | | 4 | | | | 0.003 | | | |
| 4 | | 13 | | 76395306 | | rs181473989 | | C | | T | | LMO7 | | | | . | | | 3.62 | | | . | | | 396 | | | 348 | | | 0 | | | | | 4 | | | 4 | | | | 0.005 | | | |
| 4 | | 13 | | 76419523 | | rs566129586 | | C | | T | | LMO7 | | | | . | | | 3.62 | | | . | | | 396 | | | 348 | | | 0 | | | | | 4 | | | 4 | | | | 0.001 | | | |
| 4 | | 1 | | 38025103 | | rs41267309 | | T | | C | | DNALI1 | | | | . | | | 3.62 | | | 2.28 | | | 132 | | | 116 | | | 1 | | | | | 2 | | | 2 | | | | 0.0089 | | | |
| 4 | | 13 | | 95117993 | | rs144365832 | | C | | T | | DCT | | | | D | | | 1.81 | | | 2.28 | | | 264 | | | 232 | | | 1 | | | | | 2 | | | 2 | | | | 0.004 | | | |
| 4 | | 13 | | 95121301 | | rs202004134 | | T | | C | | DCT | | | | . | | | 1.81 | | | 2.28 | | | 264 | | | 232 | | | 1 | | | | | 2 | | | 2 | | | | 0.001 | | | |
| 4 | | 14 | | 21793077 | | rs543867152 | | C | | T | | RPGRIP1 | | | | D | | | 7.24 | | | . | | | 264 | | | 232 | | | 0 | | | | | 4 | | | 4 | | | | 0.001 | | | |
| 4 | | 14 | | 21793236 | | rs7157052 | | G | | A | | RPGRIP1 | | | | . | | | 7.24 | | | . | | | 264 | | | 232 | | | 0 | | | | | 4 | | | 4 | | | | 0.002 | | | |
| 4 | | 1 | | 43664319 | | rs10711519 | | TA | | T | | CFAP57 | | | | . | | | 7.54 | | | 2.3 | | | 384 | | | 334 | | | 2 | | | | | 4 | | | 4 | | | | 0.003 | | | |
| 4 | | 1 | | 43688508 | | rs549638533 | | T | | TCAC | | CFAP57 | | | | . | | | 7.54 | | | 2.3 | | | 384 | | | 334 | | | 2 | | | | | 4 | | | 4 | | | | 0.005 | | | |
| group | | chr | | Pos | | id | | ref | | alt | | gene | | | | LR | | | **Gene burden ratio** | | | | | | **Total No. of alleles**  **in gene** | | | | | | | **No. alt alleles**  **in genes** | | | | | | | | **No. of**  **cases with**  **alt alleles** | | | | **Variant allele frequency** | | |
|  | |  | |  | |  | |  | |  | |  | | | |  | | | KG  East Asia | | | healthy  Ctrl | | | healthy  Ctrl | | | RA | | | healthy Ctrl | | | | | RA | | |  | | | | KG  East Asia | | | |
| 4 | | 1 | | 43689879 | | rs138114943 | | C | | A | | CFAP57 | | | | . | | | 7.54 | | | 2.3 | | | 384 | | | 334 | | | 2 | | | | | 4 | | | 4 | | | | 0.003 | | | |
| 4 | | 14 | | 59954385 | | rs76472382 | | A | | G | | JKAMP | | | | . | | | 2.72 | | | . | | | 130 | | | 116 | | | 0 | | | | | 3 | | | 3 | | | | 0.0069 | | | |
| 4 | | 14 | | 77735581 | | rs117207261 | | C | | G | | NGB | | | | D | | | 2.72 | | | . | | | 264 | | | 232 | | | 0 | | | | | 3 | | | 3 | | | | 0.004 | | | |
| 4 | | 14 | | 77735593 | | rs77722833 | | G | | C | | NGB | | | | D | | | 2.72 | | | . | | | 264 | | | 232 | | | 0 | | | | | 3 | | | 3 | | | | 0.001 | | | |
| 4 | | 14 | | 88406259 | | rs138577661 | | A | | G | | GALC | | | | D | | | 7.24 | | | 2.28 | | | 396 | | | 348 | | | 2 | | | | | 4 | | | 4 | | | | 0.0079 | | | |
| 4 | | 14 | | 88411994 | | rs146286491 | | C | | T | | GALC | | | | D | | | 7.24 | | | 2.28 | | | 396 | | | 348 | | | 2 | | | | | 4 | | | 4 | | | | 0.006 | | | |
| 4 | | 14 | | 88450836 | | rs534598133 | | C | | T | | GALC | | | | D | | | 7.24 | | | 2.28 | | | 396 | | | 348 | | | 2 | | | | | 4 | | | 4 | | | | 0.002 | | | |
| 4 | | 15 | | 39876498 | | rs200938835 | | T | | C | | THBS1 | | | | . | | | 3.62 | | | 4.55 | | | 396 | | | 348 | | | 1 | | | | | 4 | | | 4 | | | | 0.002 | | | |
| 4 | | 15 | | 39881204 | | rs200366954 | | A | | G | | THBS1 | | | | D | | | 3.62 | | | 4.55 | | | 396 | | | 348 | | | 1 | | | | | 4 | | | 4 | | | | 0.001 | | | |
| 4 | | 15 | | 39886402 | | rs185847032 | | G | | A | | THBS1 | | | | . | | | 3.62 | | | 4.55 | | | 396 | | | 348 | | | 1 | | | | | 4 | | | 4 | | | | 0.002 | | | |
| 4 | | 15 | | 45388079 | | rs147945181 | | G | | A | | DUOX2 | | | | D | | | 4.07 | | | 1.71 | | | 660 | | | 580 | | | 6 | | | | | 9 | | | 9 | | | | 0.005 | | | |
| 4 | | 15 | | 45388106 | | rs200541410 | | G | | A | | DUOX2 | | | | D | | | 4.07 | | | 1.71 | | | 660 | | | 580 | | | 6 | | | | | 9 | | | 9 | | | | 0.004 | | | |
| 4 | | 15 | | 45391946 | | rs368488511 | | C | | T | | DUOX2 | | | | D | | | 4.07 | | | 1.71 | | | 660 | | | 580 | | | 6 | | | | | 9 | | | 9 | | | | 0.002 | | | |
| 4 | | 15 | | 45399533 | | rs76411432 | | C | | T | | DUOX2 | | | | . | | | 4.07 | | | 1.71 | | | 660 | | | 580 | | | 6 | | | | | 9 | | | 9 | | | | 0.005 | | | |
| 4 | | 15 | | 45399648 | | rs180671269 | | T | | A | | DUOX2 | | | | . | | | 4.07 | | | 1.71 | | | 660 | | | 580 | | | 6 | | | | | 9 | | | 9 | | | | 0.003 | | | |
| 4 | | 1 | | 54694412 | | rs75191666 | | G | | A | | SSBP3 | | | | . | | | 2.84 | | | 3.35 | | | 248 | | | 222 | | | 1 | | | | | 3 | | | 3 | | | | 0.0099 | | | |
| 4 | | 1 | | 54707891 | | rs199692606 | | A | | G | | SSBP3 | | | | . | | | 2.84 | | | 3.35 | | | 248 | | | 222 | | | 1 | | | | | 3 | | | 3 | | | | 0.001 | | | |
| 4 | | 15 | | 48512855 | | rs116848967 | | G | | A | | SLC12A1 | | | | D | | | 10.86 | | | 4.55 | | | 264 | | | 232 | | | 1 | | | | | 4 | | | 4 | | | | 0.003 | | | |
| group | | chr | | Pos | | id | | ref | | alt | | gene | | | | LR | | | **Gene burden ratio** | | | | | | **Total No. of alleles**  **in gene** | | | | | | | **No. alt alleles**  **in genes** | | | | | | | | **No. of**  **cases with**  **alt alleles** | | | | **Variant allele frequency** | | |
|  | |  | |  | |  | |  | |  | |  | | | |  | | | KG  East Asia | | | healthy  Ctrl | | | healthy  Ctrl | | | RA | | | healthy Ctrl | | | | | RA | | |  | | | | KG  East Asia | | | |
| 4 | | 15 | | 48566800 | | rs201516084 | | T | | C | | SLC12A1 | | | | D | | | 10.86 | | | 4.55 | | | 264 | | | 232 | | | 1 | | | | | 4 | | | 4 | | | | 0.005 | | | |
| 4 | | 15 | | 65766537 | | rs192889990 | | A | | G | | DPP8 | | | | . | | | 5.43 | | | . | | | 264 | | | 232 | | | 0 | | | | | 3 | | | 3 | | | | 0.002 | | | |
| 4 | | 15 | | 65790192 | | rs564795298 | | T | | C | | DPP8 | | | | . | | | 5.43 | | | . | | | 264 | | | 232 | | | 0 | | | | | 3 | | | 3 | | | | 0.001 | | | |
| 4 | | 15 | | 79298783 | | rs182075492 | | G | | C | | RASGRF1 | | | | . | | | 1.81 | | | . | | | 132 | | | 116 | | | 0 | | | | | 3 | | | 3 | | | | 0.005 | | | |
| 4 | | 15 | | 89869833 | | rs55962804 | | C | | T | | POLG | | | | . | | | 2.72 | | | 1.71 | | | 132 | | | 116 | | | 2 | | | | | 3 | | | 3 | | | | 0.0089 | | | |
| 4 | | 15 | | 99670848 | | . | | G | | A | | SYNM | | | | . | | | 3.62 | | | 2.28 | | | 264 | | | 232 | | | 1 | | | | | 2 | | | 2 | | | | 0.001 | | | |
| 4 | | 15 | | 99673059 | | . | | C | | T | | SYNM | | | | . | | | 3.62 | | | 2.28 | | | 264 | | | 232 | | | 1 | | | | | 2 | | | 2 | | | | 0.002 | | | |
| 4 | | 1 | | 6184131 | | rs200938629 | | T | | A | | CHD5 | | | | D | | | 1.81 | | | 2.28 | | | 396 | | | 348 | | | 1 | | | | | 2 | | | 2 | | | | 0.004 | | | |
| 4 | | 1 | | 6188678 | | rs193121978 | | C | | G | | CHD5 | | | | . | | | 1.81 | | | 2.28 | | | 396 | | | 348 | | | 1 | | | | | 2 | | | 2 | | | | 0.001 | | | |
| 4 | | 1 | | 6228290 | | rs571052710 | | C | | G | | CHD5 | | | | D | | | 1.81 | | | 2.28 | | | 396 | | | 348 | | | 1 | | | | | 2 | | | 2 | | | | 0.002 | | | |
| 4 | | 16 | | 19883621 | | rs200345676 | | G | | T | | GPRC5B | | | | D | | | 1.81 | | | 2.28 | | | 132 | | | 116 | | | 1 | | | | | 2 | | | 2 | | | | 0.0079 | | | |
| 4 | | 16 | | 27492392 | | rs200888316 | | C | | T | | GTF3C1 | | | | . | | | 5.43 | | | 1.71 | | | 264 | | | 232 | | | 2 | | | | | 3 | | | 3 | | | | 0.001 | | | |
| 4 | | 16 | | 27494449 | | rs536534746 | | G | | A | | GTF3C1 | | | | . | | | 5.43 | | | 1.71 | | | 264 | | | 232 | | | 2 | | | | | 3 | | | 3 | | | | 0.003 | | | |
| 4 | | 16 | | 424403 | | rs11641325 | | C | | T | | TMEM8A | | | | . | | | 5.48 | | | 3.42 | | | 262 | | | 230 | | | 1 | | | | | 3 | | | 3 | | | | 0.004 | | | |
| 4 | | 16 | | 426536 | | rs143874266 | | C | | T | | TMEM8A | | | | . | | | 5.48 | | | 3.42 | | | 262 | | | 230 | | | 1 | | | | | 3 | | | 3 | | | | 0.004 | | | |
| 4 | | 16 | | 4624799 | | rs574569398 | | C | | G | | C16orf96 | | | | . | | | 3.62 | | | 2.28 | | | 264 | | | 232 | | | 1 | | | | | 2 | | | 2 | | | | 0.001 | | | |
| 4 | | 16 | | 4644389 | | rs139232890 | | CAGG | | C | | C16orf96 | | | | . | | | 3.62 | | | 2.28 | | | 264 | | | 232 | | | 1 | | | | | 2 | | | 2 | | | | 0.003 | | | |
| 4 | | 16 | | 57935248 | | rs374813501 | | C | | G | | CNGB1 | | | | . | | | 3.62 | | | 4.55 | | | 528 | | | 464 | | | 1 | | | | | 4 | | | 4 | | | | 0.0069 | | | |
| group | | chr | | Pos | | id | | ref | | alt | | gene | | | | LR | | | **Gene burden ratio** | | | | | | **Total No. of alleles**  **in gene** | | | | | | | **No. alt alleles**  **in genes** | | | | | | | | **No. of**  **cases with**  **alt alleles** | | | | **Variant allele frequency** | | |
|  | |  | |  | |  | |  | |  | |  | | | |  | | | KG  East Asia | | | healthy  Ctrl | | | healthy  Ctrl | | | RA | | | healthy Ctrl | | | | | RA | | |  | | | | KG  East Asia | | | |
| 4 | | 16 | | 57984441 | | rs146170855 | | C | | T | | CNGB1 | | | | D | | | 3.62 | | | 4.55 | | | 528 | | | 464 | | | 1 | | | | | 4 | | | 4 | | | | 0.002 | | | |
| 4 | | 16 | | 57993840 | | rs201703193 | | C | | T | | CNGB1 | | | | D | | | 3.62 | | | 4.55 | | | 528 | | | 464 | | | 1 | | | | | 4 | | | 4 | | | | 0.001 | | | |
| 4 | | 16 | | 57996967 | | rs570828500 | | G | | A | | CNGB1 | | | | D | | | 3.62 | | | 4.55 | | | 528 | | | 464 | | | 1 | | | | | 4 | | | 4 | | | | 0.003 | | | |
| 4 | | 1 | | 6638781 | | rs201116489 | | C | | T | | TAS1R1 | | | | D | | | 2.72 | | | 1.71 | | | 264 | | | 232 | | | 2 | | | | | 3 | | | 3 | | | | 0.003 | | | |
| 4 | | 1 | | 6638995 | | rs150612979 | | C | | T | | TAS1R1 | | | | D | | | 2.72 | | | 1.71 | | | 264 | | | 232 | | | 2 | | | | | 3 | | | 3 | | | | 0.006 | | | |
| 4 | | 16 | | 75642789 | | rs191524413 | | G | | C | | ADAT1 | | | | D | | | 2.72 | | | . | | | 264 | | | 232 | | | 0 | | | | | 3 | | | 3 | | | | 0.003 | | | |
| 4 | | 16 | | 75646659 | | rs536106427 | | A | | T | | ADAT1 | | | | D | | | 2.72 | | | . | | | 264 | | | 232 | | | 0 | | | | | 3 | | | 3 | | | | 0.001 | | | |
| 4 | | 16 | | 84476135 | | rs189678245 | | A | | G | | ATP2C2 | | | | D | | | 3.62 | | | 2.28 | | | 396 | | | 348 | | | 1 | | | | | 2 | | | 2 | | | | 0.001 | | | |
| 4 | | 16 | | 84482136 | | rs138818397 | | A | | G | | ATP2C2 | | | | . | | | 3.62 | | | 2.28 | | | 396 | | | 348 | | | 1 | | | | | 2 | | | 2 | | | | 0.0089 | | | |
| 4 | | 16 | | 84485620 | | rs544756548 | | C | | T | | ATP2C2 | | | | D | | | 3.62 | | | 2.28 | | | 396 | | | 348 | | | 1 | | | | | 2 | | | 2 | | | | 0.001 | | | |
| 4 | | 17 | | 10429123 | | rs187438258 | | G | | A | | MYH2 | | | | D | | | 3.62 | | | 4.55 | | | 264 | | | 232 | | | 1 | | | | | 4 | | | 4 | | | | 0.002 | | | |
| 4 | | 17 | | 10432499 | | rs150830535 | | T | | C | | MYH2 | | | | D | | | 3.62 | | | 4.55 | | | 264 | | | 232 | | | 1 | | | | | 4 | | | 4 | | | | 0.002 | | | |
| 4 | | 17 | | 10541353 | | rs201166774 | | G | | A | | MYH3 | | | | . | | | 1.81 | | | . | | | 264 | | | 232 | | | 0 | | | | | 4 | | | 4 | | | | 0.006 | | | |
| 4 | | 17 | | 10558169 | | rs374786690 | | G | | C | | MYH3 | | | | . | | | 1.81 | | | . | | | 264 | | | 232 | | | 0 | | | | | 4 | | | 4 | | | | 0.002 | | | |
| 4 | | 17 | | 15554781 | | rs140413277 | | G | | T | | TRIM16 | | | | . | | | 1.81 | | | 2.28 | | | 132 | | | 116 | | | 1 | | | | | 2 | | | 2 | | | | 0.0089 | | | |
| 4 | | 17 | | 19246867 | | rs7221577 | | T | | C | | B9D1 | | | | . | | | 6.34 | | | 1.99 | | | 396 | | | 348 | | | 4 | | | | | 7 | | | 7 | | | | 0.0079 | | | |
| 4 | | 17 | | 19246919 | | rs556859873 | | G | | T | | B9D1 | | | | . | | | 6.34 | | | 1.99 | | | 396 | | | 348 | | | 4 | | | | | 7 | | | 7 | | | | 0.005 | | | |
| 4 | | 17 | | 19247075 | | rs4924987 | | G | | A | | B9D1 | | | | . | | | 6.34 | | | 1.99 | | | 396 | | | 348 | | | 4 | | | | | 7 | | | 7 | | | | 0.004 | | | |
| group | | chr | | Pos | | id | | ref | | alt | | gene | | | | LR | | | **Gene burden ratio** | | | | | | **Total No. of alleles**  **in gene** | | | | | | | **No. alt alleles**  **in genes** | | | | | | | | **No. of**  **cases with**  **alt alleles** | | | | **Variant allele frequency** | | |
|  | |  | |  | |  | |  | |  | |  | | | |  | | | KG  East Asia | | | healthy  Ctrl | | | healthy  Ctrl | | | RA | | | healthy Ctrl | | | | | RA | | |  | | | | KG  East Asia | | | |
| 4 | | 17 | | 26856125 | | rs188424977 | | G | | A | | FOXN1 | | | | D | | | 1.81 | | | 3.41 | | | 396 | | | 348 | | | 1 | | | | | 3 | | | 3 | | | | 0.002 | | | |
| 4 | | 17 | | 26861343 | | rs200401045 | | C | | T | | FOXN1 | | | | . | | | 1.81 | | | 3.41 | | | 396 | | | 348 | | | 1 | | | | | 3 | | | 3 | | | | 0.001 | | | |
| 4 | | 17 | | 26864171 | | rs187814037 | | C | | T | | FOXN1 | | | | D | | | 1.81 | | | 3.41 | | | 396 | | | 348 | | | 1 | | | | | 3 | | | 3 | | | | 0.005 | | | |
| 4 | | 17 | | 27233472 | | rs200441251 | | T | | C | | PHF12 | | | | D | | | 1.81 | | | 2.28 | | | 396 | | | 348 | | | 1 | | | | | 2 | | | 2 | | | | 0.001 | | | |
| 4 | | 17 | | 27234599 | | rs368783828 | | G | | A | | PHF12 | | | | . | | | 1.81 | | | 2.28 | | | 396 | | | 348 | | | 1 | | | | | 2 | | | 2 | | | | 0.001 | | | |
| 4 | | 17 | | 27240924 | | rs189300962 | | G | | C | | PHF12 | | | | D | | | 1.81 | | | 2.28 | | | 396 | | | 348 | | | 1 | | | | | 2 | | | 2 | | | | 0.002 | | | |
| 4 | | 17 | | 29226507 | | rs201170896 | | C | | T | | TEFM | | | | D | | | 7.24 | | | 1.52 | | | 132 | | | 116 | | | 3 | | | | | 4 | | | 4 | | | | 0.004 | | | |
| 4 | | 17 | | 3417212 | | rs547743528 | | T | | C | | TRPV3 | | | | . | | | 2.72 | | | 3.41 | | | 264 | | | 232 | | | 1 | | | | | 3 | | | 3 | | | | 0.001 | | | |
| 4 | | 17 | | 3417877 | | rs112791047 | | G | | A | | TRPV3 | | | | . | | | 2.72 | | | 3.41 | | | 264 | | | 232 | | | 1 | | | | | 3 | | | 3 | | | | 0.004 | | | |
| 4 | | 17 | | 38792637 | | rs199790447 | | C | | G | | SMARCE1 | | | | . | | | 1.81 | | | . | | | 132 | | | 116 | | | 0 | | | | | 3 | | | 3 | | | | 0.004 | | | |
| 4 | | 17 | | 38975376 | | rs565976410 | | C | | G | | KRT10 | | | | D | | | 1.81 | | | 2.28 | | | 264 | | | 232 | | | 1 | | | | | 2 | | | 2 | | | | 0.002 | | | |
| 4 | | 17 | | 38977344 | | rs200239146 | | G | | A | | KRT10 | | | | D | | | 1.81 | | | 2.28 | | | 264 | | | 232 | | | 1 | | | | | 2 | | | 2 | | | | 0.001 | | | |
| 4 | | 17 | | 39884092 | | . | | G | | A | | HAP1 | | | | . | | | 5.43 | | | 1.71 | | | 264 | | | 232 | | | 2 | | | | | 3 | | | 3 | | | | 0.003 | | | |
| 4 | | 17 | | 39890576 | | . | | C | | T | | HAP1 | | | | . | | | 5.43 | | | 1.71 | | | 264 | | | 232 | | | 2 | | | | | 3 | | | 3 | | | | 0.006 | | | |
| 4 | | 17 | | 46878711 | | rs184362955 | | G | | A | | TTLL6 | | | | D | | | 5.43 | | | 1.71 | | | 132 | | | 116 | | | 2 | | | | | 3 | | | 3 | | | | 0.001 | | | |
| 4 | | 17 | | 5347841 | | rs192062270 | | G | | A | | DHX33 | | | | . | | | 1.81 | | | 3.98 | | | 396 | | | 348 | | | 2 | | | | | 7 | | | 7 | | | | 0.004 | | | |
| 4 | | 17 | | 5364438 | | rs192014491 | | G | | A | | DHX33 | | | | . | | | 1.81 | | | 3.98 | | | 396 | | | 348 | | | 2 | | | | | 7 | | | 7 | | | | 0.003 | | | |
| 4 | | 17 | | 5371883 | | rs16954727 | | C | | G | | DHX33 | | | | . | | | 1.81 | | | 3.98 | | | 396 | | | 348 | | | 2 | | | | | 7 | | | 7 | | | | 0.0099 | | | |
| group | | chr | | Pos | | id | | ref | | alt | | gene | | | | LR | | | **Gene burden ratio** | | | | | | **Total No. of alleles**  **in gene** | | | | | | | **No. alt alleles**  **in genes** | | | | | | | | **No. of**  **cases with**  **alt alleles** | | | | **Variant allele frequency** | | |
|  | |  | |  | |  | |  | |  | |  | | | |  | | | KG  East Asia | | | healthy  Ctrl | | | healthy  Ctrl | | | RA | | | healthy Ctrl | | | | | RA | | |  | | | | KG  East Asia | | | |
| 4 | | 17 | | 64216866 | | rs181936071 | | A | | C | | APOH | | | | . | | | 3.62 | | | 2.28 | | | 264 | | | 232 | | | 1 | | | | | 2 | | | 2 | | | | 0.0069 | | | |
| 4 | | 17 | | 64219860 | | rs373658444 | | CA | | C | | APOH | | | | . | | | 3.62 | | | 2.28 | | | 264 | | | 232 | | | 1 | | | | | 2 | | | 2 | | | | 0.0069 | | | |
| 4 | | 17 | | 6546267 | | rs145492116 | | A | | G | | TXNDC17 | | | | . | | | 5.43 | | | 1.71 | | | 132 | | | 116 | | | 2 | | | | | 3 | | | 3 | | | | 0.0069 | | | |
| 4 | | 17 | | 67079395 | | rs117323775 | | G | | T | | ABCA6 | | | | D | | | 1.63 | | | 1.64 | | | 254 | | | 232 | | | 2 | | | | | 3 | | | 3 | | | | 0.001 | | | |
| 4 | | 17 | | 67121109 | | rs200376492 | | A | | G | | ABCA6 | | | | D | | | 1.63 | | | 1.64 | | | 254 | | | 232 | | | 2 | | | | | 3 | | | 3 | | | | 0.0079 | | | |
| 4 | | 17 | | 67246623 | | rs559974558 | | G | | A | | ABCA5 | | | | D | | | 2.9 | | | 1.5 | | | 652 | | | 580 | | | 6 | | | | | 8 | | | 8 | | | | 0.001 | | | |
| 4 | | 17 | | 67247973 | | rs201343208 | | G | | A | | ABCA5 | | | | D | | | 2.9 | | | 1.5 | | | 652 | | | 580 | | | 6 | | | | | 8 | | | 8 | | | | 0.001 | | | |
| 4 | | 17 | | 67250466 | | rs199641093 | | C | | T | | ABCA5 | | | | D | | | 2.9 | | | 1.5 | | | 652 | | | 580 | | | 6 | | | | | 8 | | | 8 | | | | 0.004 | | | |
| 4 | | 17 | | 67299017 | | rs201944918 | | A | | G | | ABCA5 | | | | D | | | 2.9 | | | 1.5 | | | 652 | | | 580 | | | 6 | | | | | 8 | | | 8 | | | | 0.0079 | | | |
| 4 | | 17 | | 67305519 | | rs199888749 | | G | | A | | ABCA5 | | | | D | | | 2.9 | | | 1.5 | | | 652 | | | 580 | | | 6 | | | | | 8 | | | 8 | | | | 0.0079 | | | |
| 4 | | 17 | | 73827216 | | rs140184929 | | C | | T | | UNC13D | | | | D | | | 5.43 | | | . | | | 264 | | | 232 | | | 0 | | | | | 3 | | | 3 | | | | 0.006 | | | |
| 4 | | 17 | | 73839609 | | rs527842266 | | C | | G | | UNC13D | | | | . | | | 5.43 | | | . | | | 264 | | | 232 | | | 0 | | | | | 3 | | | 3 | | | | 0.002 | | | |
| 4 | | 17 | | 7701543 | | rs141742705 | | G | | A | | DNAH2 | | | | D | | | 1.81 | | | 2.28 | | | 396 | | | 348 | | | 1 | | | | | 2 | | | 2 | | | | 0.001 | | | |
| 4 | | 17 | | 7705344 | | rs8073196 | | G | | C | | DNAH2 | | | | . | | | 1.81 | | | 2.28 | | | 396 | | | 348 | | | 1 | | | | | 2 | | | 2 | | | | 0.001 | | | |
| 4 | | 17 | | 7736250 | | rs201527036 | | G | | A | | DNAH2 | | | | . | | | 1.81 | | | 2.28 | | | 396 | | | 348 | | | 1 | | | | | 2 | | | 2 | | | | 0.001 | | | |
| 4 | | 17 | | 79684531 | | rs201577202 | | C | | T | | SLC25A10 | | | | . | | | 3.62 | | | 1.52 | | | 264 | | | 232 | | | 3 | | | | | 4 | | | 4 | | | | 0.001 | | | |
| 4 | | 17 | | 79684871 | | rs77609145 | | A | | T | | SLC25A10 | | | | D | | | 3.62 | | | 1.52 | | | 264 | | | 232 | | | 3 | | | | | 4 | | | 4 | | | | 0.006 | | | |
| 4 | | 18 | | 2707800 | | rs184984483 | | C | | T | | SMCHD1 | | | | . | | | 5.48 | | | 3.37 | | | 258 | | | 230 | | | 1 | | | | | 3 | | | 3 | | | | 0.001 | | | |
| group | | chr | | Pos | | id | | ref | | alt | | gene | | | | LR | | | **Gene burden ratio** | | | | | | **Total No. of alleles**  **in gene** | | | | | | | **No. alt alleles**  **in genes** | | | | | | | | **No. of**  **cases with**  **alt alleles** | | | | **Variant allele frequency** | | |
|  | |  | |  | |  | |  | |  | |  | | | |  | | | KG  East Asia | | | healthy  Ctrl | | | healthy  Ctrl | | | RA | | | healthy Ctrl | | | | | RA | | |  | | | | KG  East Asia | | | |
| 4 | | 18 | | 2777922 | | rs527648000 | | C | | T | | SMCHD1 | | | | . | | | 5.48 | | | 3.37 | | | 258 | | | 230 | | | 1 | | | | | 3 | | | 3 | | | | 0.005 | | | |
| 4 | | 18 | | 28911778 | | rs147775289 | | T | | C | | DSG1 | | | | D | | | 5.43 | | | 1.71 | | | 528 | | | 464 | | | 2 | | | | | 3 | | | 3 | | | | 0.003 | | | |
| 4 | | 18 | | 28934293 | | rs149191001 | | C | | T | | DSG1 | | | | D | | | 5.43 | | | 1.71 | | | 528 | | | 464 | | | 2 | | | | | 3 | | | 3 | | | | 0.001 | | | |
| 4 | | 18 | | 28934674 | | rs181411154 | | G | | A | | DSG1 | | | | D | | | 5.43 | | | 1.71 | | | 528 | | | 464 | | | 2 | | | | | 3 | | | 3 | | | | 0.001 | | | |
| 4 | | 18 | | 28934927 | | rs148488583 | | C | | G | | DSG1 | | | | D | | | 5.43 | | | 1.71 | | | 528 | | | 464 | | | 2 | | | | | 3 | | | 3 | | | | 0.004 | | | |
| 4 | | 18 | | 580853 | | rs114933134 | | G | | A | | CETN1 | | | | D | | | 3.62 | | | . | | | 132 | | | 116 | | | 0 | | | | | 4 | | | 4 | | | | 0.005 | | | |
| 4 | | 18 | | 61160178 | | rs370525785 | | T | | C | | SERPINB5 | | | | . | | | 3.62 | | | 2.28 | | | 264 | | | 232 | | | 1 | | | | | 2 | | | 2 | | | | 0.001 | | | |
| 4 | | 18 | | 61170818 | | rs185364126 | | G | | A | | SERPINB5 | | | | D | | | 3.62 | | | 2.28 | | | 264 | | | 232 | | | 1 | | | | | 2 | | | 2 | | | | 0.002 | | | |
| 4 | | 18 | | 61305002 | | rs201297323 | | T | | C | | SERPINB4 | | | | D | | | 1.81 | | | 3.41 | | | 264 | | | 232 | | | 1 | | | | | 3 | | | 3 | | | | 0.0069 | | | |
| 4 | | 18 | | 61305289 | | rs188021365 | | A | | T | | SERPINB4 | | | | . | | | 1.81 | | | 3.41 | | | 264 | | | 232 | | | 1 | | | | | 3 | | | 3 | | | | 0.005 | | | |
| 4 | | 1 | | 87380851 | | rs546745 | | A | | G | | HS2ST1 | | | | . | | | 3.62 | | | 2.28 | | | 264 | | | 232 | | | 1 | | | | | 2 | | | 2 | | | | 0.006 | | | |
| 4 | | 1 | | 87563514 | | rs143260332 | | G | | A | | HS2ST1 | | | | . | | | 3.62 | | | 2.28 | | | 264 | | | 232 | | | 1 | | | | | 2 | | | 2 | | | | 0.004 | | | |
| 4 | | 18 | | 76886315 | | rs200431802 | | C | | T | | ATP9B | | | | D | | | 3.62 | | | 2.28 | | | 264 | | | 232 | | | 1 | | | | | 2 | | | 2 | | | | 0.004 | | | |
| 4 | | 18 | | 77096664 | | rs201172611 | | G | | A | | ATP9B | | | | D | | | 3.62 | | | 2.28 | | | 264 | | | 232 | | | 1 | | | | | 2 | | | 2 | | | | 0.001 | | | |
| 4 | | 18 | | 9549345 | | rs199964908 | | G | | A | | PPP4R1 | | | | . | | | 2.72 | | | 1.71 | | | 132 | | | 116 | | | 2 | | | | | 3 | | | 3 | | | | 0.0079 | | | |
| 4 | | 19 | | 14071095 | | rs140301367 | | G | | A | | DCAF15 | | | | . | | | 5.43 | | | 3.41 | | | 132 | | | 116 | | | 1 | | | | | 3 | | | 3 | | | | 0.0079 | | | |
| 4 | | 19 | | 15285063 | | rs141320511 | | G | | T | | NOTCH3 | | | | D | | | 4.53 | | | 5.69 | | | 396 | | | 348 | | | 1 | | | | | 5 | | | 5 | | | | 0.0099 | | | |
| 4 | | 19 | | 15298126 | | rs201118034 | | G | | A | | NOTCH3 | | | | D | | | 4.53 | | | 5.69 | | | 396 | | | 348 | | | 1 | | | | | 5 | | | 5 | | | | 0.001 | | | |
| group | chr | | pos | | id | | ref | | | | | | alt | gene | | | LR | | | **Gene burden ratio** | | | | | | **Total No. of alleles**  **in gene** | | | | | | | | **No. alt alleles**  **in genes** | | | | | | | | **No. of**  **cases with**  **alt alleles** | | | | **Variant allele frequency** |
|  |  | |  | |  | |  | | | | | |  |  | | |  | | | KG  East Asia | | | healthy  Ctrl | | | healthy  Ctrl | | | RA | | | | healthy Ctrl | | | | RA | | | |  | | | | KG  East Asia | |
| 4 | 19 | | 15302951 | | rs202157633 | | G | | | | | | A | NOTCH3 | | | D | | | 4.53 | | | 5.69 | | | 396 | | | 348 | | | | 1 | | | | 5 | | | | 5 | | | | 0.005 | |
| 4 | 19 | | 39367429 | | rs58188607 | | G | | | | | | A | RINL | | | . | | | 3.62 | | | 2.28 | | | 132 | | | 116 | | | | 1 | | | | 2 | | | | 2 | | | | 0.006 | |
| 4 | 19 | | 44662139 | | rs201230189 | | G | | | | | | A | ZNF234 | | | D | | | 1.81 | | | 2.28 | | | 132 | | | 116 | | | | 1 | | | | 2 | | | | 2 | | | | 0.004 | |
| 4 | 19 | | 41837123 | | rs199982059 | | C | | | | | | T | TGFβ1 | | | . | | | 1.81 | | | . | | | 132 | | | 116 | | | | 0 | | | | 4 | | | | 4 | | | | 0.006 | |
| 4 | 19 | | 46184983 | | rs186639840 | | A | | | | | | T | GIPR | | | . | | | 1.81 | | | 2.28 | | | 132 | | | 116 | | | | 1 | | | | 2 | | | | 2 | | | | 0.0069 | |
| 4 | 19 | | 46242968 | | rs183304235 | | G | | | | | | C | BHMG1 | | | D | | | 2.72 | | | . | | | 132 | | | 116 | | | | 0 | | | | 3 | | | | 3 | | | | 0.0069 | |
| 4 | 19 | | 51470542 | | rs201586262 | | G | | | | | | T | KLK6 | | | D | | | 5.43 | | | . | | | 132 | | | 116 | | | | 0 | | | | 3 | | | | 3 | | | | 0.001 | |
| 4 | 1 | | 95303290 | | rs140889980 | | G | | | | | | A | SLC44A3 | | | D | | | 1.81 | | | . | | | 264 | | | 232 | | | | 0 | | | | 3 | | | | 3 | | | | 0.004 | |
| 4 | 1 | | 95322899 | | rs184943086 | | C | | | | | | T | SLC44A3 | | | . | | | 1.81 | | | . | | | 264 | | | 232 | | | | 0 | | | | 3 | | | | 3 | | | | 0.002 | |
| 4 | 19 | | 8140232 | | rs145316149 | | G | | | | | | A | FBN3 | | | D | | | 1.81 | | | 3.41 | | | 528 | | | 464 | | | | 1 | | | | 3 | | | | 3 | | | | 0.004 | |
| 4 | 19 | | 8150331 | | rs142940013 | | G | | | | | | A | FBN3 | | | D | | | 1.81 | | | 3.41 | | | 528 | | | 464 | | | | 1 | | | | 3 | | | | 3 | | | | 0.004 | |
| 4 | 19 | | 8155130 | | rs183278638 | | G | | | | | | A | FBN3 | | | D | | | 1.81 | | | 3.41 | | | 528 | | | 464 | | | | 1 | | | | 3 | | | | 3 | | | | 0.002 | |
| 4 | 19 | | 8188820 | | rs145435433 | | C | | | | | | T | FBN3 | | | D | | | 1.81 | | | 3.41 | | | 528 | | | 464 | | | | 1 | | | | 3 | | | | 3 | | | | 0.006 | |
| 4 | 19 | | 8979212 | | rs149481309 | | C | | | | | | T | MUC16 | | | . | | | 3.62 | | | 1.82 | | | 528 | | | 464 | | | | 5 | | | | 8 | | | | 8 | | | | 0.0089 | |
| 4 | 19 | | 9002496 | | rs553074376 | | C | | | | | | T | MUC16 | | | . | | | 3.62 | | | 1.82 | | | 528 | | | 464 | | | | 5 | | | | 8 | | | | 8 | | | | 0.006 | |
| 4 | 19 | | 9043416 | | rs17417801 | | G | | | | | | A | MUC16 | | | . | | | 3.62 | | | 1.82 | | | 528 | | | 464 | | | | 5 | | | | 8 | | | | 8 | | | | 0.0079 | |
| 4 | 19 | | 9056878 | | rs200934751 | | GAGA | | | | | | G | MUC16 | | | . | | | 3.62 | | | 1.82 | | | 528 | | | 464 | | | | 5 | | | | 8 | | | | 8 | | | | 0.0069 | |
| 4 | 20 | | 23433244 | | rs146114915 | | G | | | | | | A | CST11 | | | . | | | 5.43 | | | 3.41 | | | 132 | | | 116 | | | | 1 | | | | 3 | | | | 3 | | | | 0.0079 | |
| group | | chr | | Pos | | id | | ref | | alt | | gene | | | | LR | | | **Gene burden ratio** | | | | | | **Total No. of alleles**  **in gene** | | | | | | | **No. alt alleles**  **in genes** | | | | | | | | **No. of**  **cases with**  **alt alleles** | | | | **Variant allele frequency** | | |
|  | |  | |  | |  | |  | |  | |  | | | |  | | | KG  East Asia | | | healthy  Ctrl | | | healthy  Ctrl | | | RA | | | healthy Ctrl | | | | | RA | | |  | | | | KG  East Asia | | | |
| 4 | 20 | | 34117097 | | rs141795719 | | G | | | | | | A | C20orf173 | | | . | | | 3.62 | | | 2.28 | | | 132 | | | 116 | | | | 1 | | | | 2 | | | | 2 | | | | 0.006 | |
| 4 | 20 | | 37394884 | | rs141204447 | | G | | | | | | A | ACTR5 | | | D | | | 6.34 | | | 7.97 | | | 264 | | | 232 | | | | 1 | | | | 7 | | | | 7 | | | | 0.0069 | |
| 4 | 20 | | 37400374 | | rs3752289 | | C | | | | | | T | ACTR5 | | | D | | | 6.34 | | | 7.97 | | | 264 | | | 232 | | | | 1 | | | | 7 | | | | 7 | | | | 0.0089 | |
| 4 | 20 | | 39788407 | | rs201733074 | | T | | | | | | C | PLCG1 | | | . | | | 1.81 | | | 6.83 | | | 396 | | | 348 | | | | 1 | | | | 6 | | | | 6 | | | | 0.0079 | |
| 4 | 20 | | 39797820 | | rs547025579 | | GACCAGAACC | | | | | | G | PLCG1 | | | . | | | 1.81 | | | 6.83 | | | 396 | | | 348 | | | | 1 | | | | 6 | | | | 6 | | | | 0.0069 | |
| 4 | 20 | | 39798092 | | rs183538599 | | C | | | | | | T | PLCG1 | | | . | | | 1.81 | | | 6.83 | | | 396 | | | 348 | | | | 1 | | | | 6 | | | | 6 | | | | 0.0079 | |
| 4 | 20 | | 39974514 | | rs201526389 | | C | | | | | | T | LPIN3 | | | D | | | 3.62 | | | 2.28 | | | 396 | | | 348 | | | | 1 | | | | 2 | | | | 2 | | | | 0.0079 | |
| 4 | 20 | | 39981270 | | rs200870645 | | C | | | | | | G | LPIN3 | | | D | | | 3.62 | | | 2.28 | | | 396 | | | 348 | | | | 1 | | | | 2 | | | | 2 | | | | 0.002 | |
| 4 | 20 | | 39987396 | | rs202035187 | | T | | | | | | C | LPIN3 | | | D | | | 3.62 | | | 2.28 | | | 396 | | | 348 | | | | 1 | | | | 2 | | | | 2 | | | | 0.003 | |
| 4 | 20 | | 40052247 | | rs569454917 | | C | | | | | | G | CHD6 | | | D | | | 3.62 | | | 2.28 | | | 264 | | | 232 | | | | 1 | | | | 2 | | | | 2 | | | | 0.001 | |
| 4 | 20 | | 40161851 | | rs75576471 | | C | | | | | | G | CHD6 | | | D | | | 3.62 | | | 2.28 | | | 264 | | | 232 | | | | 1 | | | | 2 | | | | 2 | | | | 0.0069 | |
| 4 | 20 | | 45839488 | | rs144592314 | | G | | | | | | A | ZMYND8 | | | D | | | 3.62 | | | 2.28 | | | 132 | | | 116 | | | | 1 | | | | 2 | | | | 2 | | | | 0.003 | |
| 4 | 2 | | 101093702 | | rs149056157 | | C | | | | | | T | NMS | | | . | | | 1.81 | | | . | | | 132 | | | 116 | | | | 0 | | | | 5 | | | | 5 | | | | 0.0079 | |
| 4 | 2 | | 11295694 | | rs372049512 | | C | | | | | | A | PQLC3 | | | D | | | 1.81 | | | 2.28 | | | 132 | | | 116 | | | | 1 | | | | 2 | | | | 2 | | | | 0.004 | |
| 4 | 2 | | 118577378 | | rs145263993 | | A | | | | | | C | DDX18 | | | . | | | 5.43 | | | 1.71 | | | 132 | | | 116 | | | | 2 | | | | 3 | | | | 3 | | | | 0.004 | |
| 4 | 21 | | 34018908 | | rs115353088 | | A | | | | | | T | SYNJ1 | | | D | | | 2.41 | | | 2.26 | | | 394 | | | 348 | | | | 2 | | | | 4 | | | | 4 | | | | 0.001 | |
| 4 | 21 | | 34045841 | | rs115989459 | | G | | | | | | A | SYNJ1 | | | D | | | 2.41 | | | 2.26 | | | 394 | | | 348 | | | | 2 | | | | 4 | | | | 4 | | | | 0.003 | |
| 4 | | 21 | | 34048669 | | rs533995497 | | T | | C | | SYNJ1 | | | | . | | | 2.41 | | | 2.26 | | | 394 | | | 348 | | | 2 | | | | | 4 | | | 4 | | | | 0.001 | | | |

| group | | | | | | | | | | | chr | | | | | | | | | | | | Pos | | | | | | | | | | | | id | | | | | | | | | | | | ref | | | | alt | | | | | | | gene | | | | | | | | | | | | | | LR | | | | | | | **Gene burden ratio** | | | | | | | | | | | | | | | | | | | | | | | | | | | | | | **Total No. of alleles**  **in gene** | | | | | | | | | | | | | | | | | | | | | | | | | | **No. alt alleles**  **in genes** | | | | | | | | | | | | | | | | | | | | | | | | | | **No. of**  **cases with**  **alt alleles** | | | | | | | | | | | | | | | **Variant allele frequency** | | | | | | | | | | | | |
| --- | --- | --- | --- | --- | --- | --- | --- | --- | --- | --- | --- | --- | --- | --- | --- | --- | --- | --- | --- | --- | --- | --- | --- | --- | --- | --- | --- | --- | --- | --- | --- | --- | --- | --- | --- | --- | --- | --- | --- | --- | --- | --- | --- | --- | --- | --- | --- | --- | --- | --- | --- | --- | --- | --- | --- | --- | --- | --- | --- | --- | --- | --- | --- | --- | --- | --- | --- | --- | --- | --- | --- | --- | --- | --- | --- | --- | --- | --- | --- | --- | --- | --- | --- | --- | --- | --- | --- | --- | --- | --- | --- | --- | --- | --- | --- | --- | --- | --- | --- | --- | --- | --- | --- | --- | --- | --- | --- | --- | --- | --- | --- | --- | --- | --- | --- | --- | --- | --- | --- | --- | --- | --- | --- | --- | --- | --- | --- | --- | --- | --- | --- | --- | --- | --- | --- | --- | --- | --- | --- | --- | --- | --- | --- | --- | --- | --- | --- | --- | --- | --- | --- | --- | --- | --- | --- | --- | --- | --- | --- | --- | --- | --- | --- | --- | --- | --- | --- | --- | --- | --- | --- | --- | --- | --- | --- | --- | --- | --- | --- | --- | --- | --- | --- | --- | --- | --- | --- | --- |
|  | | | | | | | | | | |  | | | | | | | | | | | |  | | | | | | | | | | | |  | | | | | | | | | | | |  | | | |  | | | | | | |  | | | | | | | | | | | | | |  | | | | | | | KG  East Asia | | | | | | | | | | | | | | | | healthy  Ctrl | | | | | | | | | | | | | healthy  Ctrl | | | | | | | | | | | | | | RA | | | | | | | | | | | | healthy Ctrl | | | | | | | | | | | | | | | RA | | | | | | | | | | |  | | | | | | | | | | | | | | | KG  East Asia | | | | | | | | | | | | | |
| 4 | | | | | | | | | | | 21 | | | | | | | | | | | | 38072227 | | | | | | | | | | | | rs200569203 | | | | | | | | | | | | G | | | | A | | | | | | | SIM2 | | | | | | | | | | | | | | . | | | | | | | 1.81 | | | | | | | | | | | | | | | | 2.28 | | | | | | | | | | | | | 264 | | | | | | | | | | | | | | 232 | | | | | | | | | | | | 1 | | | | | | | | | | | | | | | 2 | | | | | | | | | | | 2 | | | | | | | | | | | | | | | 0.001 | | | | | | | | | | | | | |
| 4 | | | | | | | | | | | 21 | | | | | | | | | | | | 38098456 | | | | | | | | | | | | rs201356831 | | | | | | | | | | | | T | | | | C | | | | | | | SIM2 | | | | | | | | | | | | | | D | | | | | | | 1.81 | | | | | | | | | | | | | | | | 2.28 | | | | | | | | | | | | | 264 | | | | | | | | | | | | | | 232 | | | | | | | | | | | | 1 | | | | | | | | | | | | | | | 2 | | | | | | | | | | | 2 | | | | | | | | | | | | | | | 0.001 | | | | | | | | | | | | | |
| 4 | | | | | | | | | | | 2 | | | | | | | | | | | | 141115543 | | | | | | | | | | | | rs77178150 | | | | | | | | | | | | C | | | | T | | | | | | | LRP1B | | | | | | | | | | | | | | . | | | | | | | 2.72 | | | | | | | | | | | | | | | | 3.41 | | | | | | | | | | | | | 396 | | | | | | | | | | | | | | 348 | | | | | | | | | | | | 1 | | | | | | | | | | | | | | | 3 | | | | | | | | | | | 3 | | | | | | | | | | | | | | | 0.0089 | | | | | | | | | | | | | |
| 4 | | | | | | | | | | | 2 | | | | | | | | | | | | 141253160 | | | | | | | | | | | | rs572325724 | | | | | | | | | | | | T | | | | A | | | | | | | LRP1B | | | | | | | | | | | | | | D | | | | | | | 2.72 | | | | | | | | | | | | | | | | 3.41 | | | | | | | | | | | | | 396 | | | | | | | | | | | | | | 348 | | | | | | | | | | | | 1 | | | | | | | | | | | | | | | 3 | | | | | | | | | | | 3 | | | | | | | | | | | | | | | 0.002 | | | | | | | | | | | | | |
| 4 | | | | | | | | | | | 2 | | | | | | | | | | | | 141458125 | | | | | | | | | | | | rs369842040 | | | | | | | | | | | | G | | | | A | | | | | | | LRP1B | | | | | | | | | | | | | | D | | | | | | | 2.72 | | | | | | | | | | | | | | | | 3.41 | | | | | | | | | | | | | 396 | | | | | | | | | | | | | | 348 | | | | | | | | | | | | 1 | | | | | | | | | | | | | | | 3 | | | | | | | | | | | 3 | | | | | | | | | | | | | | | 0.001 | | | | | | | | | | | | | |
| 4 | | | | | | | | | | | 21 | | | | | | | | | | | | 47571485 | | | | | | | | | | | | rs199508525 | | | | | | | | | | | | C | | | | T | | | | | | | FTCD | | | | | | | | | | | | | | D | | | | | | | 1.81 | | | | | | | | | | | | | | | | 3.41 | | | | | | | | | | | | | 132 | | | | | | | | | | | | | | 116 | | | | | | | | | | | | 1 | | | | | | | | | | | | | | | 3 | | | | | | | | | | | 3 | | | | | | | | | | | | | | | 0.0069 | | | | | | | | | | | | | |
| 4 | | | | | | | | | | | 2 | | | | | | | | | | | | 178534249 | | | | | | | | | | | | rs142433460 | | | | | | | | | | | | T | | | | A | | | | | | | PDE11A | | | | | | | | | | | | | | D | | | | | | | 7.24 | | | | | | | | | | | | | | | | 4.55 | | | | | | | | | | | | | 264 | | | | | | | | | | | | | | 232 | | | | | | | | | | | | 1 | | | | | | | | | | | | | | | 4 | | | | | | | | | | | 4 | | | | | | | | | | | | | | | 0.004 | | | | | | | | | | | | | |
| 4 | | | | | | | | | | | 2 | | | | | | | | | | | | 178562138 | | | | | | | | | | | | rs201572288 | | | | | | | | | | | | A | | | | T | | | | | | | PDE11A | | | | | | | | | | | | | | D | | | | | | | 7.24 | | | | | | | | | | | | | | | | 4.55 | | | | | | | | | | | | | 264 | | | | | | | | | | | | | | 232 | | | | | | | | | | | | 1 | | | | | | | | | | | | | | | 4 | | | | | | | | | | | 4 | | | | | | | | | | | | | | | 0.002 | | | | | | | | | | | | | |
| 4 | | | | | | | | | | | 2 | | | | | | | | | | | | 179404792 | | | | | | | | | | | | rs556524594 | | | | | | | | | | | | C | | | | T | | | | | | | TTN | | | | | | | | | | | | | | . | | | | | | | 3.02 | | | | | | | | | | | | | | | | 1.9 | | | | | | | | | | | | | 1056 | | | | | | | | | | | | | | 928 | | | | | | | | | | | | 3 | | | | | | | | | | | | | | | 5 | | | | | | | | | | | 5 | | | | | | | | | | | | | | | 0.001 | | | | | | | | | | | | | |
| 4 | | | | | | | | | | | 2 | | | | | | | | | | | | 179425208 | | | | | | | | | | | | rs142478636 | | | | | | | | | | | | G | | | | T | | | | | | | TTN | | | | | | | | | | | | | | D | | | | | | | 3.02 | | | | | | | | | | | | | | | | 1.9 | | | | | | | | | | | | | 1056 | | | | | | | | | | | | | | 928 | | | | | | | | | | | | 3 | | | | | | | | | | | | | | | 5 | | | | | | | | | | | 5 | | | | | | | | | | | | | | | 0.004 | | | | | | | | | | | | | |
| 4 | | | | | | | | | | | 2 | | | | | | | | | | | | 179430305 | | | | | | | | | | | | rs185887755 | | | | | | | | | | | | G | | | | A | | | | | | | TTN | | | | | | | | | | | | | | D | | | | | | | 3.02 | | | | | | | | | | | | | | | | 1.9 | | | | | | | | | | | | | 1056 | | | | | | | | | | | | | | 928 | | | | | | | | | | | | 3 | | | | | | | | | | | | | | | 5 | | | | | | | | | | | 5 | | | | | | | | | | | | | | | 0.003 | | | | | | | | | | | | | |
| 4 | | | | | | | | | | | 2 | | | | | | | | | | | | 179437342 | | | | | | | | | | | | rs567446185 | | | | | | | | | | | | C | | | | T | | | | | | | TTN | | | | | | | | | | | | | | D | | | | | | | 3.02 | | | | | | | | | | | | | | | | 1.9 | | | | | | | | | | | | | 1056 | | | | | | | | | | | | | | 928 | | | | | | | | | | | | 3 | | | | | | | | | | | | | | | 5 | | | | | | | | | | | 5 | | | | | | | | | | | | | | | 0.001 | | | | | | | | | | | | | |
| 4 | | | | | | | | | | | 2 | | | | | | | | | | | | 179481839 | | | | | | | | | | | | rs144688960 | | | | | | | | | | | | C | | | | A | | | | | | | TTN | | | | | | | | | | | | | | . | | | | | | | 3.02 | | | | | | | | | | | | | | | | 1.9 | | | | | | | | | | | | | 1056 | | | | | | | | | | | | | | 928 | | | | | | | | | | | | 3 | | | | | | | | | | | | | | | 5 | | | | | | | | | | | 5 | | | | | | | | | | | | | | | 0.001 | | | | | | | | | | | | | |
| 4 | | | | | | | | | | | 2 | | | | | | | | | | | | 179504772 | | | | | | | | | | | | rs551963261 | | | | | | | | | | | | C | | | | T | | | | | | | TTN | | | | | | | | | | | | | | . | | | | | | | 3.02 | | | | | | | | | | | | | | | | 1.9 | | | | | | | | | | | | | 1056 | | | | | | | | | | | | | | 928 | | | | | | | | | | | | 3 | | | | | | | | | | | | | | | 5 | | | | | | | | | | | 5 | | | | | | | | | | | | | | | 0.001 | | | | | | | | | | | | | |
| 4 | | | | | | | | | | | 2 | | | | | | | | | | | | 179577222 | | | | | | | | | | | | rs186857044 | | | | | | | | | | | | C | | | | A | | | | | | | TTN | | | | | | | | | | | | | | D | | | | | | | 3.02 | | | | | | | | | | | | | | | | 1.9 | | | | | | | | | | | | | 1056 | | | | | | | | | | | | | | 928 | | | | | | | | | | | | 3 | | | | | | | | | | | | | | | 5 | | | | | | | | | | | 5 | | | | | | | | | | | | | | | 0.001 | | | | | | | | | | | | | |
| 4 | | | | | | | | | | | 2 | | | | | | | | | | | | 179585717 | | | | | | | | | | | | rs367826445 | | | | | | | | | | | | C | | | | T | | | | | | | TTN | | | | | | | | | | | | | | D | | | | | | | 3.02 | | | | | | | | | | | | | | | | 1.9 | | | | | | | | | | | | | 1056 | | | | | | | | | | | | | | 928 | | | | | | | | | | | | 3 | | | | | | | | | | | | | | | 5 | | | | | | | | | | | 5 | | | | | | | | | | | | | | | 0.002 | | | | | | | | | | | | | |
| 4 | | | | | | | | | | | 2 | | | | | | | | | | | | 196718225 | | | | | | | | | | | | rs139835496 | | | | | | | | | | | | G | | | | A | | | | | | | DNAH7 | | | | | | | | | | | | | | . | | | | | | | 2.72 | | | | | | | | | | | | | | | | . | | | | | | | | | | | | | 132 | | | | | | | | | | | | | | 116 | | | | | | | | | | | | 0 | | | | | | | | | | | | | | | 3 | | | | | | | | | | | 3 | | | | | | | | | | | | | | | 0.005 | | | | | | | | | | | | | |
| group | | | | | | | | | | | chr | | | | | | | | | | | | Pos | | | | | | | | | | | | id | | | | | | | | | | | | ref | | | | alt | | | | | | | gene | | | | | | | | | | | | | | LR | | | | | | | **Gene burden ratio** | | | | | | | | | | | | | | | | | | | | | | | | | | | | | | **Total No. of alleles**  **in gene** | | | | | | | | | | | | | | | | | | | | | | | | | | **No. alt alleles**  **in genes** | | | | | | | | | | | | | | | | | | | | | | | | | | **No. of**  **cases with**  **alt alleles** | | | | | | | | | | | | | | | **Variant allele frequency** | | | | | | | | | | | | |
|  | | | | | | | | | | |  | | | | | | | | | | | |  | | | | | | | | | | | |  | | | | | | | | | | | |  | | | |  | | | | | | |  | | | | | | | | | | | | | |  | | | | | | | KG  East Asia | | | | | | | | | | | | | | | | healthy  Ctrl | | | | | | | | | | | | | healthy  Ctrl | | | | | | | | | | | | | | RA | | | | | | | | | | | | healthy Ctrl | | | | | | | | | | | | | | | RA | | | | | | | | | | |  | | | | | | | | | | | | | | | KG  East Asia | | | | | | | | | | | | | |
| 4 | | | | | | | | | | | 2 | | | | | | | | | | | | 202356803 | | | | | | | | | | | | rs557048083 | | | | | | | | | | | | G | | | | A | | | | | | | ALS2CR11 | | | | | | | | | | | | | | . | | | | | | | 2.72 | | | | | | | | | | | | | | | | . | | | | | | | | | | | | | 264 | | | | | | | | | | | | | | 232 | | | | | | | | | | | | 0 | | | | | | | | | | | | | | | 3 | | | | | | | | | | | 3 | | | | | | | | | | | | | | | 0.001 | | | | | | | | | | | | | |
| 4 | | | | | | | | | | | 2 | | | | | | | | | | | | 202467972 | | | | | | | | | | | | rs148342903 | | | | | | | | | | | | C | | | | G | | | | | | | ALS2CR11 | | | | | | | | | | | | | | D | | | | | | | 2.72 | | | | | | | | | | | | | | | | . | | | | | | | | | | | | | 264 | | | | | | | | | | | | | | 232 | | | | | | | | | | | | 0 | | | | | | | | | | | | | | | 3 | | | | | | | | | | | 3 | | | | | | | | | | | | | | | 0.0069 | | | | | | | | | | | | | |
| 4 | | | | | | | | | | | 2 | | | | | | | | | | | | 203058233 | | | | | | | | | | | | rs13024221 | | | | | | | | | | | | T | | | | C | | | | | | | KIAA2012 | | | | | | | | | | | | | | . | | | | | | | 7.3 | | | | | | | | | | | | | | | | 4.59 | | | | | | | | | | | | | 264 | | | | | | | | | | | | | | 230 | | | | | | | | | | | | 1 | | | | | | | | | | | | | | | 4 | | | | | | | | | | | 4 | | | | | | | | | | | | | | | 0.003 | | | | | | | | | | | | | |
| 4 | | | | | | | | | | | 2 | | | | | | | | | | | | 203059076 | | | | | | | | | | | | rs141298049 | | | | | | | | | | | | G | | | | A | | | | | | | KIAA2012 | | | | | | | | | | | | | | . | | | | | | | 7.3 | | | | | | | | | | | | | | | | 4.59 | | | | | | | | | | | | | 264 | | | | | | | | | | | | | | 230 | | | | | | | | | | | | 1 | | | | | | | | | | | | | | | 4 | | | | | | | | | | | 4 | | | | | | | | | | | | | | | 0.006 | | | | | | | | | | | | | |
| 4 | | | | | | | | | | | 2 | | | | | | | | | | | | 208477907 | | | | | | | | | | | | rs192886645 | | | | | | | | | | | | G | | | | A | | | | | | | METTL21A | | | | | | | | | | | | | | . | | | | | | | 3.62 | | | | | | | | | | | | | | | | 2.28 | | | | | | | | | | | | | 132 | | | | | | | | | | | | | | 116 | | | | | | | | | | | | 1 | | | | | | | | | | | | | | | 2 | | | | | | | | | | | 2 | | | | | | | | | | | | | | | 0.002 | | | | | | | | | | | | | |
| 4 | | | | | | | | | | | 2 | | | | | | | | | | | | 213921827 | | | | | | | | | | | | rs150075012 | | | | | | | | | | | | T | | | | C | | | | | | | IKZF2 | | | | | | | | | | | | | | . | | | | | | | 2.72 | | | | | | | | | | | | | | | | 1.71 | | | | | | | | | | | | | 132 | | | | | | | | | | | | | | 116 | | | | | | | | | | | | 2 | | | | | | | | | | | | | | | 3 | | | | | | | | | | | 3 | | | | | | | | | | | | | | | 0.0089 | | | | | | | | | | | | | |
| 4 | | | | | | | | | | | 2 | | | | | | | | | | | | 219029361 | | | | | | | | | | | | rs201920477 | | | | | | | | | | | | C | | | | T | | | | | | | CXCR1 | | | | | | | | | | | | | | D | | | | | | | 3.62 | | | | | | | | | | | | | | | | 2.28 | | | | | | | | | | | | | 132 | | | | | | | | | | | | | | 116 | | | | | | | | | | | | 1 | | | | | | | | | | | | | | | 2 | | | | | | | | | | | 2 | | | | | | | | | | | | | | | 0.004 | | | | | | | | | | | | | |
| 4 | | | | | | | | | | | 22 | | | | | | | | | | | | 19868190 | | | | | | | | | | | | rs184640901 | | | | | | | | | | | | C | | | | G | | | | | | | TXNRD2 | | | | | | | | | | | | | | . | | | | | | | 2.72 | | | | | | | | | | | | | | | | 3.41 | | | | | | | | | | | | | 396 | | | | | | | | | | | | | | 348 | | | | | | | | | | | | 1 | | | | | | | | | | | | | | | 3 | | | | | | | | | | | 3 | | | | | | | | | | | | | | | 0.002 | | | | | | | | | | | | | |
| 4 | | | | | | | | | | | 22 | | | | | | | | | | | | 19870857 | | | | | | | | | | | | rs147383232 | | | | | | | | | | | | G | | | | A | | | | | | | TXNRD2 | | | | | | | | | | | | | | . | | | | | | | 2.72 | | | | | | | | | | | | | | | | 3.41 | | | | | | | | | | | | | 396 | | | | | | | | | | | | | | 348 | | | | | | | | | | | | 1 | | | | | | | | | | | | | | | 3 | | | | | | | | | | | 3 | | | | | | | | | | | | | | | 0.0089 | | | | | | | | | | | | | |
| 4 | | | | | | | | | | | 22 | | | | | | | | | | | | 26159232 | | | | | | | | | | | | rs79294358 | | | | | | | | | | | | C | | | | T | | | | | | | MYO18B | | | | | | | | | | | | | | D | | | | | | | 2.41 | | | | | | | | | | | | | | | | 4.55 | | | | | | | | | | | | | 396 | | | | | | | | | | | | | | 348 | | | | | | | | | | | | 1 | | | | | | | | | | | | | | | 4 | | | | | | | | | | | 4 | | | | | | | | | | | | | | | 0.0079 | | | | | | | | | | | | | |
| 4 | | | | | | | | | | | 22 | | | | | | | | | | | | 26166958 | | | | | | | | | | | | rs117430010 | | | | | | | | | | | | C | | | | T | | | | | | | MYO18B | | | | | | | | | | | | | | . | | | | | | | 2.41 | | | | | | | | | | | | | | | | 4.55 | | | | | | | | | | | | | 396 | | | | | | | | | | | | | | 348 | | | | | | | | | | | | 1 | | | | | | | | | | | | | | | 4 | | | | | | | | | | | 4 | | | | | | | | | | | | | | | 0.006 | | | | | | | | | | | | | |
| 4 | | | | | | | | | | | 22 | | | | | | | | | | | | 26264278 | | | | | | | | | | | | rs137859315 | | | | | | | | | | | | T | | | | C | | | | | | | MYO18B | | | | | | | | | | | | | | . | | | | | | | 2.41 | | | | | | | | | | | | | | | | 4.55 | | | | | | | | | | | | | 396 | | | | | | | | | | | | | | 348 | | | | | | | | | | | | 1 | | | | | | | | | | | | | | | 4 | | | | | | | | | | | 4 | | | | | | | | | | | | | | | 0.0079 | | | | | | | | | | | | | |
| 4 | | | | | | | | | | | 2 | | | | | | | | | | | | 227872169 | | | | | | | | | | | | rs192411379 | | | | | | | | | | | | T | | | | C | | | | | | | COL4A4 | | | | | | | | | | | | | | D | | | | | | | 2.72 | | | | | | | | | | | | | | | | 3.4 | | | | | | | | | | | | | 526 | | | | | | | | | | | | | | 464 | | | | | | | | | | | | 1 | | | | | | | | | | | | | | | 3 | | | | | | | | | | | 3 | | | | | | | | | | | | | | | 0.001 | | | | | | | | | | | | | |
| 4 | | | | | | | | | | | 2 | | | | | | | | | | | | 227920837 | | | | | | | | | | | | rs199710625 | | | | | | | | | | | | A | | | | G | | | | | | | COL4A4 | | | | | | | | | | | | | | . | | | | | | | 2.72 | | | | | | | | | | | | | | | | 3.4 | | | | | | | | | | | | | 526 | | | | | | | | | | | | | | 464 | | | | | | | | | | | | 1 | | | | | | | | | | | | | | | 3 | | | | | | | | | | | 3 | | | | | | | | | | | | | | | 0.004 | | | | | | | | | | | | | |
| 4 | | | | | | | | | | | 2 | | | | | | | | | | | | 227967506 | | | | | | | | | | | | rs373741172 | | | | | | | | | | | | C | | | | T | | | | | | | COL4A4 | | | | | | | | | | | | | | D | | | | | | | 2.72 | | | | | | | | | | | | | | | | 3.4 | | | | | | | | | | | | | 526 | | | | | | | | | | | | | | 464 | | | | | | | | | | | | 1 | | | | | | | | | | | | | | | 3 | | | | | | | | | | | 3 | | | | | | | | | | | | | | | 0.001 | | | | | | | | | | | | | |
| 4 | | | | | | | | | | | 2 | | | | | | | | | | | | 227985873 | | | | | | | | | | | | rs190570269 | | | | | | | | | | | | G | | | | C | | | | | | | COL4A4 | | | | | | | | | | | | | | . | | | | | | | 2.72 | | | | | | | | | | | | | | | | 3.4 | | | | | | | | | | | | | 526 | | | | | | | | | | | | | | 464 | | | | | | | | | | | | 1 | | | | | | | | | | | | | | | 3 | | | | | | | | | | | 3 | | | | | | | | | | | | | | | 0.006 | | | | | | | | | | | | | |
| 4 | | | | | | | | | | | 22 | | | | | | | | | | | | 29881766 | | | | | | | | | | | | rs201416955 | | | | | | | | | | | | G | | | | A | | | | | | | NEFH | | | | | | | | | | | | | | D | | | | | | | 1.81 | | | | | | | | | | | | | | | | 2.28 | | | | | | | | | | | | | 264 | | | | | | | | | | | | | | 232 | | | | | | | | | | | | 1 | | | | | | | | | | | | | | | 2 | | | | | | | | | | | 2 | | | | | | | | | | | | | | | 0.003 | | | | | | | | | | | | | |
| 4 | | | | | | | | | | | 22 | | | | | | | | | | | | 29881844 | | | | | | | | | | | | rs117036372 | | | | | | | | | | | | G | | | | A | | | | | | | NEFH | | | | | | | | | | | | | | . | | | | | | | 1.81 | | | | | | | | | | | | | | | | 2.28 | | | | | | | | | | | | | 264 | | | | | | | | | | | | | | 232 | | | | | | | | | | | | 1 | | | | | | | | | | | | | | | 2 | | | | | | | | | | | 2 | | | | | | | | | | | | | | | 0.006 | | | | | | | | | | | | | |
| group | | | | | | | | | | | chr | | | | | | | | | | | | Pos | | | | | | | | | | | | id | | | | | | | | | | | | ref | | | | alt | | | | | | | gene | | | | | | | | | | | | | | LR | | | | | | | **Gene burden ratio** | | | | | | | | | | | | | | | | | | | | | | | | | | | | | | **Total No. of alleles**  **in gene** | | | | | | | | | | | | | | | | | | | | | | | | | | **No. alt alleles**  **in genes** | | | | | | | | | | | | | | | | | | | | | | | | | | **No. of**  **cases with**  **alt alleles** | | | | | | | | | | | | | | | **Variant allele frequency** | | | | | | | | | | | | |
|  | | | | | | | | | | |  | | | | | | | | | | | |  | | | | | | | | | | | |  | | | | | | | | | | | |  | | | |  | | | | | | |  | | | | | | | | | | | | | |  | | | | | | | KG  East Asia | | | | | | | | | | | | | | | | healthy  Ctrl | | | | | | | | | | | | | healthy  Ctrl | | | | | | | | | | | | | | RA | | | | | | | | | | | | healthy Ctrl | | | | | | | | | | | | | | | RA | | | | | | | | | | |  | | | | | | | | | | | | | | | KG  East Asia | | | | | | | | | | | | | |
| 4 | | | | | | | | | | | 22 | | | | | | | | | | | | 31522450 | | | | | | | | | | | | rs150976596 | | | | | | | | | | | | G | | | | A | | | | | | | INPP5J | | | | | | | | | | | | | | D | | | | | | | 3.62 | | | | | | | | | | | | | | | | 1.9 | | | | | | | | | | | | | 264 | | | | | | | | | | | | | | 232 | | | | | | | | | | | | 3 | | | | | | | | | | | | | | | 5 | | | | | | | | | | | 5 | | | | | | | | | | | | | | | 0.0099 | | | | | | | | | | | | | |
| 4 | | | | | | | | | | | 22 | | | | | | | | | | | | 31522715 | | | | | | | | | | | | rs370874308 | | | | | | | | | | | | A | | | | T | | | | | | | INPP5J | | | | | | | | | | | | | | . | | | | | | | 3.62 | | | | | | | | | | | | | | | | 1.9 | | | | | | | | | | | | | 264 | | | | | | | | | | | | | | 232 | | | | | | | | | | | | 3 | | | | | | | | | | | | | | | 5 | | | | | | | | | | | 5 | | | | | | | | | | | | | | | 0.003 | | | | | | | | | | | | | |
| 4 | | | | | | | | | | | 22 | | | | | | | | | | | | 32614713 | | | | | | | | | | | | rs78144589 | | | | | | | | | | | | C | | | | T | | | | | | | SLC5A4 | | | | | | | | | | | | | | . | | | | | | | 5.43 | | | | | | | | | | | | | | | | 6.83 | | | | | | | | | | | | | 264 | | | | | | | | | | | | | | 232 | | | | | | | | | | | | 1 | | | | | | | | | | | | | | | 6 | | | | | | | | | | | 6 | | | | | | | | | | | | | | | 0.0099 | | | | | | | | | | | | | |
| 4 | | | | | | | | | | | 22 | | | | | | | | | | | | 32631002 | | | | | | | | | | | | rs554791323 | | | | | | | | | | | | T | | | | C | | | | | | | SLC5A4 | | | | | | | | | | | | | | D | | | | | | | 5.43 | | | | | | | | | | | | | | | | 6.83 | | | | | | | | | | | | | 264 | | | | | | | | | | | | | | 232 | | | | | | | | | | | | 1 | | | | | | | | | | | | | | | 6 | | | | | | | | | | | 6 | | | | | | | | | | | | | | | 0.001 | | | | | | | | | | | | | |
| 4 | | | | | | | | | | | 2 | | | | | | | | | | | | 234835171 | | | | | | | | | | | | rs188545335 | | | | | | | | | | | | G | | | | A | | | | | | | TRPM8 | | | | | | | | | | | | | | . | | | | | | | 1.81 | | | | | | | | | | | | | | | | 2.28 | | | | | | | | | | | | | 264 | | | | | | | | | | | | | | 232 | | | | | | | | | | | | 1 | | | | | | | | | | | | | | | 2 | | | | | | | | | | | 2 | | | | | | | | | | | | | | | 0.002 | | | | | | | | | | | | | |
| 4 | | | | | | | | | | | 2 | | | | | | | | | | | | 234890409 | | | | | | | | | | | | rs202160114 | | | | | | | | | | | | T | | | | C | | | | | | | TRPM8 | | | | | | | | | | | | | | . | | | | | | | 1.81 | | | | | | | | | | | | | | | | 2.28 | | | | | | | | | | | | | 264 | | | | | | | | | | | | | | 232 | | | | | | | | | | | | 1 | | | | | | | | | | | | | | | 2 | | | | | | | | | | | 2 | | | | | | | | | | | | | | | 0.001 | | | | | | | | | | | | | |
| 4 | | | | | | | | | | | 2 | | | | | | | | | | | | 242046783 | | | | | | | | | | | | rs199503351 | | | | | | | | | | | | G | | | | A | | | | | | | PASK | | | | | | | | | | | | | | . | | | | | | | 5.43 | | | | | | | | | | | | | | | | 3.41 | | | | | | | | | | | | | 396 | | | | | | | | | | | | | | 348 | | | | | | | | | | | | 1 | | | | | | | | | | | | | | | 3 | | | | | | | | | | | 3 | | | | | | | | | | | | | | | 0.002 | | | | | | | | | | | | | |
| 4 | | | | | | | | | | | 2 | | | | | | | | | | | | 242047581 | | | | | | | | | | | | rs563432464 | | | | | | | | | | | | C | | | | A | | | | | | | PASK | | | | | | | | | | | | | | . | | | | | | | 5.43 | | | | | | | | | | | | | | | | 3.41 | | | | | | | | | | | | | 396 | | | | | | | | | | | | | | 348 | | | | | | | | | | | | 1 | | | | | | | | | | | | | | | 3 | | | | | | | | | | | 3 | | | | | | | | | | | | | | | 0.001 | | | | | | | | | | | | | |
| 4 | | | | | | | | | | | 2 | | | | | | | | | | | | 242089016 | | | | | | | | | | | | rs187718988 | | | | | | | | | | | | G | | | | C | | | | | | | PASK | | | | | | | | | | | | | | . | | | | | | | 5.43 | | | | | | | | | | | | | | | | 3.41 | | | | | | | | | | | | | 396 | | | | | | | | | | | | | | 348 | | | | | | | | | | | | 1 | | | | | | | | | | | | | | | 3 | | | | | | | | | | | 3 | | | | | | | | | | | | | | | 0.001 | | | | | | | | | | | | | |
| 4 | | | | | | | | | | | 2 | | | | | | | | | | | | 242695306 | | | | | | | | | | | | rs149628174 | | | | | | | | | | | | C | | | | T | | | | | | | D2HGDH | | | | | | | | | | | | | | D | | | | | | | 4.53 | | | | | | | | | | | | | | | | 2.84 | | | | | | | | | | | | | 132 | | | | | | | | | | | | | | 116 | | | | | | | | | | | | 2 | | | | | | | | | | | | | | | 5 | | | | | | | | | | | 5 | | | | | | | | | | | | | | | 0.0099 | | | | | | | | | | | | | |
| 4 | | | | | | | | | | | 22 | | | | | | | | | | | | 43926729 | | | | | | | | | | | | rs201816198 | | | | | | | | | | | | C | | | | A | | | | | | | EFCAB6 | | | | | | | | | | | | | | D | | | | | | | 1.63 | | | | | | | | | | | | | | | | 3.41 | | | | | | | | | | | | | 264 | | | | | | | | | | | | | | 232 | | | | | | | | | | | | 1 | | | | | | | | | | | | | | | 3 | | | | | | | | | | | 3 | | | | | | | | | | | | | | | 0.0079 | | | | | | | | | | | | | |
| 4 | | | | | | | | | | | 22 | | | | | | | | | | | | 44030977 | | | | | | | | | | | | rs181939688 | | | | | | | | | | | | G | | | | C | | | | | | | EFCAB6 | | | | | | | | | | | | | | . | | | | | | | 1.63 | | | | | | | | | | | | | | | | 3.41 | | | | | | | | | | | | | 264 | | | | | | | | | | | | | | 232 | | | | | | | | | | | | 1 | | | | | | | | | | | | | | | 3 | | | | | | | | | | | 3 | | | | | | | | | | | | | | | 0.001 | | | | | | | | | | | | | |
| 4 | | | | | | | | | | | 22 | | | | | | | | | | | | 46664409 | | | | | | | | | | | | rs144175578 | | | | | | | | | | | | A | | | | G | | | | | | | TTC38 | | | | | | | | | | | | | | . | | | | | | | 3.62 | | | | | | | | | | | | | | | | . | | | | | | | | | | | | | 264 | | | | | | | | | | | | | | 232 | | | | | | | | | | | | 0 | | | | | | | | | | | | | | | 4 | | | | | | | | | | | 4 | | | | | | | | | | | | | | | 0.0099 | | | | | | | | | | | | | |
| 4 | | | | | | | | | | | 22 | | | | | | | | | | | | 46684376 | | | | | | | | | | | | rs202139216 | | | | | | | | | | | | C | | | | T | | | | | | | TTC38 | | | | | | | | | | | | | | . | | | | | | | 3.62 | | | | | | | | | | | | | | | | . | | | | | | | | | | | | | 264 | | | | | | | | | | | | | | 232 | | | | | | | | | | | | 0 | | | | | | | | | | | | | | | 4 | | | | | | | | | | | 4 | | | | | | | | | | | | | | | 0.001 | | | | | | | | | | | | | |
| 4 | | | | | | | | | | | 22 | | | | | | | | | | | | 51041663 | | | | | | | | | | | | rs41282359 | | | | | | | | | | | | C | | | | A | | | | | | | MAPK8IP2 | | | | | | | | | | | | | | . | | | | | | | 4.53 | | | | | | | | | | | | | | | | 2.84 | | | | | | | | | | | | | 924 | | | | | | | | | | | | | | 812 | | | | | | | | | | | | 2 | | | | | | | | | | | | | | | 5 | | | | | | | | | | | 5 | | | | | | | | | | | | | | | 0.002 | | | | | | | | | | | | | |
| 4 | | | | | | | | | | | 22 | | | | | | | | | | | | 51042484 | | | | | | | | | | | | rs56314791 | | | | | | | | | | | | C | | | | T | | | | | | | MAPK8IP2 | | | | | | | | | | | | | | . | | | | | | | 4.53 | | | | | | | | | | | | | | | | 2.84 | | | | | | | | | | | | | 924 | | | | | | | | | | | | | | 812 | | | | | | | | | | | | 2 | | | | | | | | | | | | | | | 5 | | | | | | | | | | | 5 | | | | | | | | | | | | | | | 0.002 | | | | | | | | | | | | | |
| 4 | | | | | | | | | | | 22 | | | | | | | | | | | | 51042861 | | | | | | | | | | | | rs9616795 | | | | | | | | | | | | C | | | | G | | | | | | | MAPK8IP2 | | | | | | | | | | | | | | . | | | | | | | 4.53 | | | | | | | | | | | | | | | | 2.84 | | | | | | | | | | | | | 924 | | | | | | | | | | | | | | 812 | | | | | | | | | | | | 2 | | | | | | | | | | | | | | | 5 | | | | | | | | | | | 5 | | | | | | | | | | | | | | | 0.004 | | | | | | | | | | | | | |
| 4 | | | | | | | | | | | 22 | | | | | | | | | | | | 51042864 | | | | | | | | | | | | rs571810591 | | | | | | | | | | | | G | | | | C | | | | | | | MAPK8IP2 | | | | | | | | | | | | | | . | | | | | | | 4.53 | | | | | | | | | | | | | | | | 2.84 | | | | | | | | | | | | | 924 | | | | | | | | | | | | | | 812 | | | | | | | | | | | | 2 | | | | | | | | | | | | | | | 5 | | | | | | | | | | | 5 | | | | | | | | | | | | | | | 0.001 | | | | | | | | | | | | | |
| group | | | | | | | | | | | chr | | | | | | | | | | | | Pos | | | | | | | | | | | | id | | | | | | | | | | | | ref | | | | alt | | | | | | | gene | | | | | | | | | | | | | | LR | | | | | | | **Gene burden ratio** | | | | | | | | | | | | | | | | | | | | | | | | | | | | | | **Total No. of alleles**  **in gene** | | | | | | | | | | | | | | | | | | | | | | | | | | **No. alt alleles**  **in genes** | | | | | | | | | | | | | | | | | | | | | | | | | | **No. of**  **cases with**  **alt alleles** | | | | | | | | | | | | | | | **Variant allele frequency** | | | | | | | | | | | | |
|  | | | | | | | | | | |  | | | | | | | | | | | |  | | | | | | | | | | | |  | | | | | | | | | | | |  | | | |  | | | | | | |  | | | | | | | | | | | | | |  | | | | | | | KG  East Asia | | | | | | | | | | | | | | | | healthy  Ctrl | | | | | | | | | | | | | healthy  Ctrl | | | | | | | | | | | | | | RA | | | | | | | | | | | | healthy Ctrl | | | | | | | | | | | | | | | RA | | | | | | | | | | |  | | | | | | | | | | | | | | | KG  East Asia | | | | | | | | | | | | | |
| 4 | | | | | | | | | | | 22 | | | | | | | | | | | | 51043374 | | | | | | | | | | | | rs550444582 | | | | | | | | | | | | G | | | | A | | | | | | | MAPK8IP2 | | | | | | | | | | | | | | . | | | | | | | 4.53 | | | | | | | | | | | | | | | | 2.84 | | | | | | | | | | | | | 924 | | | | | | | | | | | | | | 812 | | | | | | | | | | | | 2 | | | | | | | | | | | | | | | 5 | | | | | | | | | | | 5 | | | | | | | | | | | | | | | 0.002 | | | | | | | | | | | | | |
| 4 | | | | | | | | | | | 22 | | | | | | | | | | | | 51044243 | | | | | | | | | | | | rs916005 | | | | | | | | | | | | C | | | | T | | | | | | | MAPK8IP2 | | | | | | | | | | | | | | . | | | | | | | 4.53 | | | | | | | | | | | | | | | | 2.84 | | | | | | | | | | | | | 924 | | | | | | | | | | | | | | 812 | | | | | | | | | | | | 2 | | | | | | | | | | | | | | | 5 | | | | | | | | | | | 5 | | | | | | | | | | | | | | | 0.004 | | | | | | | | | | | | | |
| 4 | | | | | | | | | | | 22 | | | | | | | | | | | | 51045178 | | | | | | | | | | | | rs200208943 | | | | | | | | | | | | C | | | | T | | | | | | | MAPK8IP2 | | | | | | | | | | | | | | . | | | | | | | 4.53 | | | | | | | | | | | | | | | | 2.84 | | | | | | | | | | | | | 924 | | | | | | | | | | | | | | 812 | | | | | | | | | | | | 2 | | | | | | | | | | | | | | | 5 | | | | | | | | | | | 5 | | | | | | | | | | | | | | | 0.001 | | | | | | | | | | | | | |
| 4 | | | | | | | | | | | 2 | | | | | | | | | | | | 27721143 | | | | | | | | | | | | rs146175795 | | | | | | | | | | | | G | | | | A | | | | | | | GCKR | | | | | | | | | | | | | | D | | | | | | | 5.43 | | | | | | | | | | | | | | | | 3.41 | | | | | | | | | | | | | 264 | | | | | | | | | | | | | | 232 | | | | | | | | | | | | 1 | | | | | | | | | | | | | | | 3 | | | | | | | | | | | 3 | | | | | | | | | | | | | | | 0.0069 | | | | | | | | | | | | | |
| 4 | | | | | | | | | | | 2 | | | | | | | | | | | | 27730834 | | | | | | | | | | | | rs200225266 | | | | | | | | | | | | C | | | | T | | | | | | | GCKR | | | | | | | | | | | | | | . | | | | | | | 5.43 | | | | | | | | | | | | | | | | 3.41 | | | | | | | | | | | | | 264 | | | | | | | | | | | | | | 232 | | | | | | | | | | | | 1 | | | | | | | | | | | | | | | 3 | | | | | | | | | | | 3 | | | | | | | | | | | | | | | 0.004 | | | | | | | | | | | | | |
| 4 | | | | | | | | | | | 2 | | | | | | | | | | | | 31751329 | | | | | | | | | | | | rs9332966 | | | | | | | | | | | | G | | | | C | | | | | | | SRD5A2 | | | | | | | | | | | | | | . | | | | | | | 1.81 | | | | | | | | | | | | | | | | 2.28 | | | | | | | | | | | | | 264 | | | | | | | | | | | | | | 232 | | | | | | | | | | | | 1 | | | | | | | | | | | | | | | 2 | | | | | | | | | | | 2 | | | | | | | | | | | | | | | 0.005 | | | | | | | | | | | | | |
| 4 | | | | | | | | | | | 2 | | | | | | | | | | | | 31805775 | | | | | | | | | | | | rs550866120 | | | | | | | | | | | | C | | | | T | | | | | | | SRD5A2 | | | | | | | | | | | | | | . | | | | | | | 1.81 | | | | | | | | | | | | | | | | 2.28 | | | | | | | | | | | | | 264 | | | | | | | | | | | | | | 232 | | | | | | | | | | | | 1 | | | | | | | | | | | | | | | 2 | | | | | | | | | | | 2 | | | | | | | | | | | | | | | 0.003 | | | | | | | | | | | | | |
| 4 | | | | | | | | | | | 2 | | | | | | | | | | | | 46588019 | | | | | | | | | | | | rs150877473 | | | | | | | | | | | | C | | | | G | | | | | | | EPAS1 | | | | | | | | | | | | | | . | | | | | | | 2.72 | | | | | | | | | | | | | | | | 3.41 | | | | | | | | | | | | | 264 | | | | | | | | | | | | | | 232 | | | | | | | | | | | | 1 | | | | | | | | | | | | | | | 3 | | | | | | | | | | | 3 | | | | | | | | | | | | | | | 0.0099 | | | | | | | | | | | | | |
| 4 | | | | | | | | | | | 2 | | | | | | | | | | | | 46603672 | | | | | | | | | | | | rs187543960 | | | | | | | | | | | | C | | | | G | | | | | | | EPAS1 | | | | | | | | | | | | | | . | | | | | | | 2.72 | | | | | | | | | | | | | | | | 3.41 | | | | | | | | | | | | | 264 | | | | | | | | | | | | | | 232 | | | | | | | | | | | | 1 | | | | | | | | | | | | | | | 3 | | | | | | | | | | | 3 | | | | | | | | | | | | | | | 0.004 | | | | | | | | | | | | | |
| 4 | | | | | | | | | | | 2 | | | | | | | | | | | | 65571844 | | | | | | | | | | | | rs182442107 | | | | | | | | | | | | T | | | | C | | | | | | | SPRED2 | | | | | | | | | | | | | | . | | | | | | | 3.62 | | | | | | | | | | | | | | | | 2.28 | | | | | | | | | | | | | 132 | | | | | | | | | | | | | | 116 | | | | | | | | | | | | 1 | | | | | | | | | | | | | | | 2 | | | | | | | | | | | 2 | | | | | | | | | | | | | | | 0.002 | | | | | | | | | | | | | |
| 4 | | | | | | | | | | | 2 | | | | | | | | | | | | 98165911 | | | | | | | | | | | | rs1839230 | | | | | | | | | | | | T | | | | C | | | | | | | ANKRD36B | | | | | | | | | | | | | | . | | | | | | | 3.62 | | | | | | | | | | | | | | | | . | | | | | | | | | | | | | 132 | | | | | | | | | | | | | | 116 | | | | | | | | | | | | 0 | | | | | | | | | | | | | | | 4 | | | | | | | | | | | 4 | | | | | | | | | | | | | | | 0.0069 | | | | | | | | | | | | | |
| 4 | | | | | | | | | | | 3 | | | | | | | | | | | | 108147410 | | | | | | | | | | | | rs552849827 | | | | | | | | | | | | T | | | | C | | | | | | | MYH15 | | | | | | | | | | | | | | D | | | | | | | 3.02 | | | | | | | | | | | | | | | | 1.86 | | | | | | | | | | | | | 518 | | | | | | | | | | | | | | 464 | | | | | | | | | | | | 3 | | | | | | | | | | | | | | | 5 | | | | | | | | | | | 5 | | | | | | | | | | | | | | | 0.001 | | | | | | | | | | | | | |
| 4 | | | | | | | | | | | 3 | | | | | | | | | | | | 108178230 | | | | | | | | | | | | rs560378764 | | | | | | | | | | | | G | | | | A | | | | | | | MYH15 | | | | | | | | | | | | | | D | | | | | | | 3.02 | | | | | | | | | | | | | | | | 1.86 | | | | | | | | | | | | | 518 | | | | | | | | | | | | | | 464 | | | | | | | | | | | | 3 | | | | | | | | | | | | | | | 5 | | | | | | | | | | | 5 | | | | | | | | | | | | | | | 0.001 | | | | | | | | | | | | | |
| 4 | | | | | | | | | | | 3 | | | | | | | | | | | | 108219046 | | | | | | | | | | | | rs182324086 | | | | | | | | | | | | C | | | | A | | | | | | | MYH15 | | | | | | | | | | | | | | D | | | | | | | 3.02 | | | | | | | | | | | | | | | | 1.86 | | | | | | | | | | | | | 518 | | | | | | | | | | | | | | 464 | | | | | | | | | | | | 3 | | | | | | | | | | | | | | | 5 | | | | | | | | | | | 5 | | | | | | | | | | | | | | | 0.001 | | | | | | | | | | | | | |
| 4 | | | | | | | | | | | 3 | | | | | | | | | | | | 108220556 | | | | | | | | | | | | rs368131843 | | | | | | | | | | | | C | | | | T | | | | | | | MYH15 | | | | | | | | | | | | | | . | | | | | | | 3.02 | | | | | | | | | | | | | | | | 1.86 | | | | | | | | | | | | | 518 | | | | | | | | | | | | | | 464 | | | | | | | | | | | | 3 | | | | | | | | | | | | | | | 5 | | | | | | | | | | | 5 | | | | | | | | | | | | | | | 0.006 | | | | | | | | | | | | | |
| 4 | | | | | | | | | | | 3 | | | | | | | | | | | | 121713035 | | | | | | | | | | | | rs142746163 | | | | | | | | | | | | G | | | | A | | | | | | | ILDR1 | | | | | | | | | | | | | | . | | | | | | | 1.81 | | | | | | | | | | | | | | | | . | | | | | | | | | | | | | 264 | | | | | | | | | | | | | | 232 | | | | | | | | | | | | 0 | | | | | | | | | | | | | | | 3 | | | | | | | | | | | 3 | | | | | | | | | | | | | | | 0.0099 | | | | | | | | | | | | | |
| 4 | | | | | | | | | | | 3 | | | | | | | | | | | | 121724081 | | | | | | | | | | | | rs200883040 | | | | | | | | | | | | C | | | | G | | | | | | | ILDR1 | | | | | | | | | | | | | | . | | | | | | | 1.81 | | | | | | | | | | | | | | | | . | | | | | | | | | | | | | 264 | | | | | | | | | | | | | | 232 | | | | | | | | | | | | 0 | | | | | | | | | | | | | | | 3 | | | | | | | | | | | 3 | | | | | | | | | | | | | | | 0.003 | | | | | | | | | | | | | |
| 4 | | | | | | | | | | | 3 | | | | | | | | | | | | 124485068 | | | | | | | | | | | | rs200221434 | | | | | | | | | | | | C | | | | T | | | | | | | ITGB5 | | | | | | | | | | | | | | . | | | | | | | 2.72 | | | | | | | | | | | | | | | | . | | | | | | | | | | | | | 396 | | | | | | | | | | | | | | 348 | | | | | | | | | | | | 0 | | | | | | | | | | | | | | | 3 | | | | | | | | | | | 3 | | | | | | | | | | | | | | | 0.002 | | | | | | | | | | | | | |
| group | | | | | | | | | | | chr | | | | | | | | | | | | Pos | | | | | | | | | | | | id | | | | | | | | | | | | ref | | | | alt | | | | | | | gene | | | | | | | | | | | | | | LR | | | | | | | **Gene burden ratio** | | | | | | | | | | | | | | | | | | | | | | | | | | | | | | **Total No. of alleles**  **in gene** | | | | | | | | | | | | | | | | | | | | | | | | | | **No. alt alleles**  **in genes** | | | | | | | | | | | | | | | | | | | | | | | | | | **No. of**  **cases with**  **alt alleles** | | | | | | | | | | | | | | | **Variant allele frequency** | | | | | | | | | | | | |
|  | | | | | | | | | | |  | | | | | | | | | | | |  | | | | | | | | | | | |  | | | | | | | | | | | |  | | | |  | | | | | | |  | | | | | | | | | | | | | |  | | | | | | | KG  East Asia | | | | | | | | | | | | | | | | healthy  Ctrl | | | | | | | | | | | | | healthy  Ctrl | | | | | | | | | | | | | | RA | | | | | | | | | | | | healthy Ctrl | | | | | | | | | | | | | | | RA | | | | | | | | | | |  | | | | | | | | | | | | | | | KG  East Asia | | | | | | | | | | | | | |
| 4 | | | | | | | | | | | 3 | | | | | | | | | | | | 124492606 | | | | | | | | | | | | rs140023830 | | | | | | | | | | | | G | | | | A | | | | | | | ITGB5 | | | | | | | | | | | | | | D | | | | | | | 2.72 | | | | | | | | | | | | | | | | . | | | | | | | | | | | | | 396 | | | | | | | | | | | | | | 348 | | | | | | | | | | | | 0 | | | | | | | | | | | | | | | 3 | | | | | | | | | | | 3 | | | | | | | | | | | | | | | 0.0079 | | | | | | | | | | | | | |
| 4 | | | | | | | | | | | 3 | | | | | | | | | | | | 124567399 | | | | | | | | | | | | rs28372859 | | | | | | | | | | | | T | | | | A | | | | | | | ITGB5 | | | | | | | | | | | | | | D | | | | | | | 2.72 | | | | | | | | | | | | | | | | . | | | | | | | | | | | | | 396 | | | | | | | | | | | | | | 348 | | | | | | | | | | | | 0 | | | | | | | | | | | | | | | 3 | | | | | | | | | | | 3 | | | | | | | | | | | | | | | 0.002 | | | | | | | | | | | | | |
| 4 | | | | | | | | | | | 3 | | | | | | | | | | | | 124716667 | | | | | | | | | | | | rs181022733 | | | | | | | | | | | | G | | | | T | | | | | | | HEG1 | | | | | | | | | | | | | | D | | | | | | | 7.24 | | | | | | | | | | | | | | | | 2.28 | | | | | | | | | | | | | 396 | | | | | | | | | | | | | | 348 | | | | | | | | | | | | 2 | | | | | | | | | | | | | | | 4 | | | | | | | | | | | 4 | | | | | | | | | | | | | | | 0.003 | | | | | | | | | | | | | |
| 4 | | | | | | | | | | | 3 | | | | | | | | | | | | 124731800 | | | | | | | | | | | | rs183321802 | | | | | | | | | | | | T | | | | A | | | | | | | HEG1 | | | | | | | | | | | | | | D | | | | | | | 7.24 | | | | | | | | | | | | | | | | 2.28 | | | | | | | | | | | | | 396 | | | | | | | | | | | | | | 348 | | | | | | | | | | | | 2 | | | | | | | | | | | | | | | 4 | | | | | | | | | | | 4 | | | | | | | | | | | | | | | 0.004 | | | | | | | | | | | | | |
| 4 | | | | | | | | | | | 3 | | | | | | | | | | | | 124738285 | | | | | | | | | | | | rs200164121 | | | | | | | | | | | | G | | | | C | | | | | | | HEG1 | | | | | | | | | | | | | | D | | | | | | | 7.24 | | | | | | | | | | | | | | | | 2.28 | | | | | | | | | | | | | 396 | | | | | | | | | | | | | | 348 | | | | | | | | | | | | 2 | | | | | | | | | | | | | | | 4 | | | | | | | | | | | 4 | | | | | | | | | | | | | | | 0.0079 | | | | | | | | | | | | | |
| 4 | | | | | | | | | | | 3 | | | | | | | | | | | | 130120633 | | | | | | | | | | | | rs370632529 | | | | | | | | | | | | T | | | | G | | | | | | | COL6A5 | | | | | | | | | | | | | | D | | | | | | | 3.62 | | | | | | | | | | | | | | | | 1.52 | | | | | | | | | | | | | 528 | | | | | | | | | | | | | | 464 | | | | | | | | | | | | 3 | | | | | | | | | | | | | | | 4 | | | | | | | | | | | 4 | | | | | | | | | | | | | | | 0.002 | | | | | | | | | | | | | |
| 4 | | | | | | | | | | | 3 | | | | | | | | | | | | 130124457 | | | | | | | | | | | | rs537224684 | | | | | | | | | | | | A | | | | G | | | | | | | COL6A5 | | | | | | | | | | | | | | D | | | | | | | 3.62 | | | | | | | | | | | | | | | | 1.52 | | | | | | | | | | | | | 528 | | | | | | | | | | | | | | 464 | | | | | | | | | | | | 3 | | | | | | | | | | | | | | | 4 | | | | | | | | | | | 4 | | | | | | | | | | | | | | | 0.001 | | | | | | | | | | | | | |
| 4 | | | | | | | | | | | 3 | | | | | | | | | | | | 130145236 | | | | | | | | | | | | rs202221090 | | | | | | | | | | | | G | | | | A | | | | | | | COL6A5 | | | | | | | | | | | | | | D | | | | | | | 3.62 | | | | | | | | | | | | | | | | 1.52 | | | | | | | | | | | | | 528 | | | | | | | | | | | | | | 464 | | | | | | | | | | | | 3 | | | | | | | | | | | | | | | 4 | | | | | | | | | | | 4 | | | | | | | | | | | | | | | 0.003 | | | | | | | | | | | | | |
| 4 | | | | | | | | | | | 3 | | | | | | | | | | | | 130150614 | | | | | | | | | | | | rs79358579 | | | | | | | | | | | | C | | | | T | | | | | | | COL6A5 | | | | | | | | | | | | | | D | | | | | | | 3.62 | | | | | | | | | | | | | | | | 1.52 | | | | | | | | | | | | | 528 | | | | | | | | | | | | | | 464 | | | | | | | | | | | | 3 | | | | | | | | | | | | | | | 4 | | | | | | | | | | | 4 | | | | | | | | | | | | | | | 0.003 | | | | | | | | | | | | | |
| 4 | | | | | | | | | | | 3 | | | | | | | | | | | | 13420488 | | | | | | | | | | | | rs200748145 | | | | | | | | | | | | T | | | | C | | | | | | | NUP210 | | | | | | | | | | | | | | . | | | | | | | 5.43 | | | | | | | | | | | | | | | | . | | | | | | | | | | | | | 132 | | | | | | | | | | | | | | 116 | | | | | | | | | | | | 0 | | | | | | | | | | | | | | | 3 | | | | | | | | | | | 3 | | | | | | | | | | | | | | | 0.001 | | | | | | | | | | | | | |
| 4 | | | | | | | | | | | 3 | | | | | | | | | | | | 154861227 | | | | | | | | | | | | rs2304504 | | | | | | | | | | | | C | | | | T | | | | | | | MME | | | | | | | | | | | | | | . | | | | | | | 5.43 | | | | | | | | | | | | | | | | 1.71 | | | | | | | | | | | | | 396 | | | | | | | | | | | | | | 348 | | | | | | | | | | | | 2 | | | | | | | | | | | | | | | 3 | | | | | | | | | | | 3 | | | | | | | | | | | | | | | 0.003 | | | | | | | | | | | | | |
| 4 | | | | | | | | | | | 3 | | | | | | | | | | | | 154861228 | | | | | | | | | | | | rs182602615 | | | | | | | | | | | | G | | | | A | | | | | | | MME | | | | | | | | | | | | | | . | | | | | | | 5.43 | | | | | | | | | | | | | | | | 1.71 | | | | | | | | | | | | | 396 | | | | | | | | | | | | | | 348 | | | | | | | | | | | | 2 | | | | | | | | | | | | | | | 3 | | | | | | | | | | | 3 | | | | | | | | | | | | | | | 0.003 | | | | | | | | | | | | | |
| 4 | | | | | | | | | | | 3 | | | | | | | | | | | | 154886310 | | | | | | | | | | | | rs200308077 | | | | | | | | | | | | G | | | | A | | | | | | | MME | | | | | | | | | | | | | | D | | | | | | | 5.43 | | | | | | | | | | | | | | | | 1.71 | | | | | | | | | | | | | 396 | | | | | | | | | | | | | | 348 | | | | | | | | | | | | 2 | | | | | | | | | | | | | | | 3 | | | | | | | | | | | 3 | | | | | | | | | | | | | | | 0.002 | | | | | | | | | | | | | |
| 4 | | | | | | | | | | | 3 | | | | | | | | | | | | 183906578 | | | | | | | | | | | | rs531703061 | | | | | | | | | | | | C | | | | T | | | | | | | ABCF3 | | | | | | | | | | | | | | D | | | | | | | 5.43 | | | | | | | | | | | | | | | | 4.55 | | | | | | | | | | | | | 264 | | | | | | | | | | | | | | 232 | | | | | | | | | | | | 1 | | | | | | | | | | | | | | | 4 | | | | | | | | | | | 4 | | | | | | | | | | | | | | | 0.003 | | | | | | | | | | | | | |
| 4 | | | | | | | | | | | 3 | | | | | | | | | | | | 183911416 | | | | | | | | | | | | rs76223160 | | | | | | | | | | | | G | | | | A | | | | | | | ABCF3 | | | | | | | | | | | | | | D | | | | | | | 5.43 | | | | | | | | | | | | | | | | 4.55 | | | | | | | | | | | | | 264 | | | | | | | | | | | | | | 232 | | | | | | | | | | | | 1 | | | | | | | | | | | | | | | 4 | | | | | | | | | | | 4 | | | | | | | | | | | | | | | 0.006 | | | | | | | | | | | | | |
| 4 | | | | | | | | | | | 3 | | | | | | | | | | | | 183952934 | | | | | | | | | | | | rs374722127 | | | | | | | | | | | | C | | | | T | | | | | | | VWA5B2 | | | | | | | | | | | | | | . | | | | | | | 2.41 | | | | | | | | | | | | | | | | 1.52 | | | | | | | | | | | | | 264 | | | | | | | | | | | | | | 232 | | | | | | | | | | | | 3 | | | | | | | | | | | | | | | 4 | | | | | | | | | | | 4 | | | | | | | | | | | | | | | 0.001 | | | | | | | | | | | | | |
| 4 | | | | | | | | | | | 3 | | | | | | | | | | | | 183957472 | | | | | | | | | | | | rs565285822 | | | | | | | | | | | | C | | | | T | | | | | | | VWA5B2 | | | | | | | | | | | | | | . | | | | | | | 2.41 | | | | | | | | | | | | | | | | 1.52 | | | | | | | | | | | | | 264 | | | | | | | | | | | | | | 232 | | | | | | | | | | | | 3 | | | | | | | | | | | | | | | 4 | | | | | | | | | | | 4 | | | | | | | | | | | | | | | 0.0089 | | | | | | | | | | | | | |
| 4 | | | | | | | | | | | 3 | | | | | | | | | | | | 186937924 | | | | | | | | | | | | rs3774266 | | | | | | | | | | | | C | | | | T | | | | | | | MASP1 | | | | | | | | | | | | | | D | | | | | | | 3.62 | | | | | | | | | | | | | | | | 2.28 | | | | | | | | | | | | | 264 | | | | | | | | | | | | | | 232 | | | | | | | | | | | | 1 | | | | | | | | | | | | | | | 2 | | | | | | | | | | | 2 | | | | | | | | | | | | | | | 0.004 | | | | | | | | | | | | | |
| group | | | | | | | | | | | chr | | | | | | | | | | | | Pos | | | | | | | | | | | | id | | | | | | | | | | | | ref | | | | alt | | | | | | | gene | | | | | | | | | | | | | | LR | | | | | | | **Gene burden ratio** | | | | | | | | | | | | | | | | | | | | | | | | | | | | | | **Total No. of alleles**  **in gene** | | | | | | | | | | | | | | | | | | | | | | | | | | **No. alt alleles**  **in genes** | | | | | | | | | | | | | | | | | | | | | | | | | | **No. of**  **cases with**  **alt alleles** | | | | | | | | | | | | | | | **Variant allele frequency** | | | | | | | | | | | | |
|  | | | | | | | | | | |  | | | | | | | | | | | |  | | | | | | | | | | | |  | | | | | | | | | | | |  | | | |  | | | | | | |  | | | | | | | | | | | | | |  | | | | | | | KG  East Asia | | | | | | | | | | | | | | | | healthy  Ctrl | | | | | | | | | | | | | healthy  Ctrl | | | | | | | | | | | | | | RA | | | | | | | | | | | | healthy Ctrl | | | | | | | | | | | | | | | RA | | | | | | | | | | |  | | | | | | | | | | | | | | | KG  East Asia | | | | | | | | | | | | | |
| 4 | | | | | | | | | | | 3 | | | | | | | | | | | | 186953975 | | | | | | | | | | | | rs72549155 | | | | | | | | | | | | G | | | | C | | | | | | | MASP1 | | | | | | | | | | | | | | D | | | | | | | 3.62 | | | | | | | | | | | | | | | | 2.28 | | | | | | | | | | | | | 264 | | | | | | | | | | | | | | 232 | | | | | | | | | | | | 1 | | | | | | | | | | | | | | | 2 | | | | | | | | | | | 2 | | | | | | | | | | | | | | | 0.003 | | | | | | | | | | | | | |
| 4 | | | | | | | | | | | 3 | | | | | | | | | | | | 2928719 | | | | | | | | | | | | rs184171731 | | | | | | | | | | | | A | | | | C | | | | | | | CNTN4 | | | | | | | | | | | | | | . | | | | | | | 4.53 | | | | | | | | | | | | | | | | 5.69 | | | | | | | | | | | | | 396 | | | | | | | | | | | | | | 348 | | | | | | | | | | | | 1 | | | | | | | | | | | | | | | 5 | | | | | | | | | | | 5 | | | | | | | | | | | | | | | 0.003 | | | | | | | | | | | | | |
| 4 | | | | | | | | | | | 3 | | | | | | | | | | | | 3080611 | | | | | | | | | | | | rs10510251 | | | | | | | | | | | | G | | | | C | | | | | | | CNTN4 | | | | | | | | | | | | | | . | | | | | | | 4.53 | | | | | | | | | | | | | | | | 5.69 | | | | | | | | | | | | | 396 | | | | | | | | | | | | | | 348 | | | | | | | | | | | | 1 | | | | | | | | | | | | | | | 5 | | | | | | | | | | | 5 | | | | | | | | | | | | | | | 0.003 | | | | | | | | | | | | | |
| 4 | | | | | | | | | | | 3 | | | | | | | | | | | | 3081959 | | | | | | | | | | | | rs339284 | | | | | | | | | | | | T | | | | C | | | | | | | CNTN4 | | | | | | | | | | | | | | . | | | | | | | 4.53 | | | | | | | | | | | | | | | | 5.69 | | | | | | | | | | | | | 396 | | | | | | | | | | | | | | 348 | | | | | | | | | | | | 1 | | | | | | | | | | | | | | | 5 | | | | | | | | | | | 5 | | | | | | | | | | | | | | | 0.002 | | | | | | | | | | | | | |
| 4 | | | | | | | | | | | 3 | | | | | | | | | | | | 44762826 | | | | | | | | | | | | rs181738022 | | | | | | | | | | | | C | | | | T | | | | | | | ZNF502 | | | | | | | | | | | | | | D | | | | | | | 2.72 | | | | | | | | | | | | | | | | 3.41 | | | | | | | | | | | | | 264 | | | | | | | | | | | | | | 232 | | | | | | | | | | | | 2 | | | | | | | | | | | | | | | 6 | | | | | | | | | | | 6 | | | | | | | | | | | | | | | 0.0079 | | | | | | | | | | | | | |
| 4 | | | | | | | | | | | 3 | | | | | | | | | | | | 44762827 | | | | | | | | | | | | rs185260708 | | | | | | | | | | | | A | | | | T | | | | | | | ZNF502 | | | | | | | | | | | | | | D | | | | | | | 2.72 | | | | | | | | | | | | | | | | 3.41 | | | | | | | | | | | | | 264 | | | | | | | | | | | | | | 232 | | | | | | | | | | | | 2 | | | | | | | | | | | | | | | 6 | | | | | | | | | | | 6 | | | | | | | | | | | | | | | 0.0079 | | | | | | | | | | | | | |
| 4 | | | | | | | | | | | 3 | | | | | | | | | | | | 49850176 | | | | | | | | | | | | rs192575536 | | | | | | | | | | | | G | | | | A | | | | | | | UBA7 | | | | | | | | | | | | | | . | | | | | | | 3.62 | | | | | | | | | | | | | | | | 2.28 | | | | | | | | | | | | | 132 | | | | | | | | | | | | | | 116 | | | | | | | | | | | | 1 | | | | | | | | | | | | | | | 2 | | | | | | | | | | | 2 | | | | | | | | | | | | | | | 0.002 | | | | | | | | | | | | | |
| 4 | | | | | | | | | | | 3 | | | | | | | | | | | | 58107069 | | | | | | | | | | | | rs76471260 | | | | | | | | | | | | G | | | | A | | | | | | | FLNB | | | | | | | | | | | | | | D | | | | | | | 2.41 | | | | | | | | | | | | | | | | 4.55 | | | | | | | | | | | | | 396 | | | | | | | | | | | | | | 348 | | | | | | | | | | | | 1 | | | | | | | | | | | | | | | 4 | | | | | | | | | | | 4 | | | | | | | | | | | | | | | 0.002 | | | | | | | | | | | | | |
| 4 | | | | | | | | | | | 3 | | | | | | | | | | | | 58109123 | | | | | | | | | | | | rs199959926 | | | | | | | | | | | | G | | | | C | | | | | | | FLNB | | | | | | | | | | | | | | D | | | | | | | 2.41 | | | | | | | | | | | | | | | | 4.55 | | | | | | | | | | | | | 396 | | | | | | | | | | | | | | 348 | | | | | | | | | | | | 1 | | | | | | | | | | | | | | | 4 | | | | | | | | | | | 4 | | | | | | | | | | | | | | | 0.006 | | | | | | | | | | | | | |
| 4 | | | | | | | | | | | 3 | | | | | | | | | | | | 58134099 | | | | | | | | | | | | rs143066905 | | | | | | | | | | | | A | | | | G | | | | | | | FLNB | | | | | | | | | | | | | | . | | | | | | | 2.41 | | | | | | | | | | | | | | | | 4.55 | | | | | | | | | | | | | 396 | | | | | | | | | | | | | | 348 | | | | | | | | | | | | 1 | | | | | | | | | | | | | | | 4 | | | | | | | | | | | 4 | | | | | | | | | | | | | | | 0.0099 | | | | | | | | | | | | | |
| 4 | | | | | | | | | | | 3 | | | | | | | | | | | | 62309627 | | | | | | | | | | | | rs1881268 | | | | | | | | | | | | G | | | | C | | | | | | | C3orf14 | | | | | | | | | | | | | | . | | | | | | | 1.81 | | | | | | | | | | | | | | | | . | | | | | | | | | | | | | 264 | | | | | | | | | | | | | | 232 | | | | | | | | | | | | 0 | | | | | | | | | | | | | | | 3 | | | | | | | | | | | 3 | | | | | | | | | | | | | | | 0.001 | | | | | | | | | | | | | |
| 4 | | | | | | | | | | | 3 | | | | | | | | | | | | 62317022 | | | | | | | | | | | | rs186089632 | | | | | | | | | | | | C | | | | A | | | | | | | C3orf14 | | | | | | | | | | | | | | . | | | | | | | 1.81 | | | | | | | | | | | | | | | | . | | | | | | | | | | | | | 264 | | | | | | | | | | | | | | 232 | | | | | | | | | | | | 0 | | | | | | | | | | | | | | | 3 | | | | | | | | | | | 3 | | | | | | | | | | | | | | | 0.003 | | | | | | | | | | | | | |
| 4 | | | | | | | | | | | 4 | | | | | | | | | | | | 15599021 | | | | | | | | | | | | rs117667651 | | | | | | | | | | | | C | | | | A | | | | | | | CC2D2A | | | | | | | | | | | | | | . | | | | | | | 2.72 | | | | | | | | | | | | | | | | . | | | | | | | | | | | | | 132 | | | | | | | | | | | | | | 116 | | | | | | | | | | | | 0 | | | | | | | | | | | | | | | 3 | | | | | | | | | | | 3 | | | | | | | | | | | | | | | 0.0079 | | | | | | | | | | | | | |
| 4 | | | | | | | | | | | 4 | | | | | | | | | | | | 16168246 | | | | | | | | | | | | rs2271748 | | | | | | | | | | | | C | | | | T | | | | | | | TAPT1 | | | | | | | | | | | | | | . | | | | | | | 2.9 | | | | | | | | | | | | | | | | 4.55 | | | | | | | | | | | | | 132 | | | | | | | | | | | | | | 116 | | | | | | | | | | | | 1 | | | | | | | | | | | | | | | 4 | | | | | | | | | | | 4 | | | | | | | | | | | | | | | 0.0099 | | | | | | | | | | | | | |
| 4 | | | | | | | | | | | 4 | | | | | | | | | | | | 17842302 | | | | | | | | | | | | rs527561771 | | | | | | | | | | | | TAA | | | | T | | | | | | | NCAPG | | | | | | | | | | | | | | . | | | | | | | 2.76 | | | | | | | | | | | | | | | | 1.66 | | | | | | | | | | | | | 126 | | | | | | | | | | | | | | 114 | | | | | | | | | | | | 2 | | | | | | | | | | | | | | | 3 | | | | | | | | | | | 3 | | | | | | | | | | | | | | | 0.0079 | | | | | | | | | | | | | |
| 4 | | | | | | | | | | | 4 | | | | | | | | | | | | 26322320 | | | | | | | | | | | | rs200707132 | | | | | | | | | | | | A | | | | C | | | | | | | RBPJ | | | | | | | | | | | | | | . | | | | | | | 3.62 | | | | | | | | | | | | | | | | 2.28 | | | | | | | | | | | | | 132 | | | | | | | | | | | | | | 116 | | | | | | | | | | | | 1 | | | | | | | | | | | | | | | 2 | | | | | | | | | | | 2 | | | | | | | | | | | | | | | 0.0079 | | | | | | | | | | | | | |
| 4 | | | | | | | | | | | 4 | | | | | | | | | | | | 6596385 | | | | | | | | | | | | rs3216941 | | | | | | | | | | | | AC | | | | A | | | | | | | MAN2B2 | | | | | | | | | | | | | | . | | | | | | | 5.43 | | | | | | | | | | | | | | | | 3.41 | | | | | | | | | | | | | 132 | | | | | | | | | | | | | | 116 | | | | | | | | | | | | 1 | | | | | | | | | | | | | | | 3 | | | | | | | | | | | 3 | | | | | | | | | | | | | | | 0.006 | | | | | | | | | | | | | |
| 4 | | | | | | | | | | | 4 | | | | | | | | | | | | 674349 | | | | | | | | | | | | rs376244258 | | | | | | | | | | | | AC | | | | A | | | | | | | MYL5 | | | | | | | | | | | | | | . | | | | | | | 3.62 | | | | | | | | | | | | | | | | 2.28 | | | | | | | | | | | | | 132 | | | | | | | | | | | | | | 116 | | | | | | | | | | | | 1 | | | | | | | | | | | | | | | 2 | | | | | | | | | | | 2 | | | | | | | | | | | | | | | 0.006 | | | | | | | | | | | | | |
| group | | | | | | | | | chr | | | | | | | | | | | | pos | | | | | | | | | | | | id | | | | | | | | | | | | ref | | | | | | | | alt | | | | | | gene | | | | | | | | | | | | | | LR | | | | | | | | | **Gene burden ratio** | | | | | | | | | | | | | | | | | | | | | | | | | | | | **Total No. of alleles**  **in gene** | | | | | | | | | | | | | | | | | | | | | | | | | | **No. alt alleles**  **in genes** | | | | | | | | | | | | | | | | | | | | | | | | | | **No. of**  **cases with**  **alt alleles** | | | | | | | | | | | | | | | **Variant allele frequency** | | | | | | | | | | | |
|  | | | | | | | | |  | | | | | | | | | | | |  | | | | | | | | | | | |  | | | | | | | | | | | |  | | | | | | | |  | | | | | |  | | | | | | | | | | | | | |  | | | | | | | | | KG  East Asia | | | | | | | | | | | | | | | healthy  Ctrl | | | | | | | | | | | | | healthy  Ctrl | | | | | | | | | | | | | RA | | | | | | | | | | | | | healthy Ctrl | | | | | | | | | | | | | | RA | | | | | | | | | | | |  | | | | | | | | | | | | | | | KG  East Asia | | | | | | | | | | | |
| 4 | | | | | | | | | 4 | | | | | | | | | | | | 84378118 | | | | | | | | | | | | rs199634680 | | | | | | | | | | | | A | | | | | | | | AT | | | | | | MRPS18C | | | | | | | | | | | | | | . | | | | | | | | | 1.84 | | | | | | | | | | | | | | | 2.32 | | | | | | | | | | | | | 132 | | | | | | | | | | | | | 114 | | | | | | | | | | | | | 1 | | | | | | | | | | | | | | 2 | | | | | | | | | | | | 2 | | | | | | | | | | | | | | | 0.0079 | | | | | | | | | | | |
| 4 | | | | | | | | | 4 | | | | | | | | | | | | 96762012 | | | | | | | | | | | | rs144813590 | | | | | | | | | | | | C | | | | | | | | G | | | | | | PDHA2 | | | | | | | | | | | | | | D | | | | | | | | | 5.43 | | | | | | | | | | | | | | | . | | | | | | | | | | | | | 132 | | | | | | | | | | | | | 116 | | | | | | | | | | | | | 0 | | | | | | | | | | | | | | 3 | | | | | | | | | | | | 3 | | | | | | | | | | | | | | | 0.003 | | | | | | | | | | | |
| 4 | | | | | | | | | 4 | | | | | | | | | | | | 983115 | | | | | | | | | | | | rs143381873 | | | | | | | | | | | | G | | | | | | | | A | | | | | | SLC26A1 | | | | | | | | | | | | | | D | | | | | | | | | 3.62 | | | | | | | | | | | | | | | 1.52 | | | | | | | | | | | | | 528 | | | | | | | | | | | | | 464 | | | | | | | | | | | | | 3 | | | | | | | | | | | | | | 4 | | | | | | | | | | | | 4 | | | | | | | | | | | | | | | 0.005 | | | | | | | | | | | |
| 4 | | | | | | | | | 4 | | | | | | | | | | | | 983342 | | | | | | | | | | | | rs201608921 | | | | | | | | | | | | C | | | | | | | | T | | | | | | SLC26A1 | | | | | | | | | | | | | | D | | | | | | | | | 3.62 | | | | | | | | | | | | | | | 1.52 | | | | | | | | | | | | | 528 | | | | | | | | | | | | | 464 | | | | | | | | | | | | | 3 | | | | | | | | | | | | | | 4 | | | | | | | | | | | | 4 | | | | | | | | | | | | | | | 0.002 | | | | | | | | | | | |
| 4 | | | | | | | | | 4 | | | | | | | | | | | | 983810 | | | | | | | | | | | | rs563866785 | | | | | | | | | | | | G | | | | | | | | A | | | | | | SLC26A1 | | | | | | | | | | | | | | D | | | | | | | | | 3.62 | | | | | | | | | | | | | | | 1.52 | | | | | | | | | | | | | 528 | | | | | | | | | | | | | 464 | | | | | | | | | | | | | 3 | | | | | | | | | | | | | | 4 | | | | | | | | | | | | 4 | | | | | | | | | | | | | | | 0.001 | | | | | | | | | | | |
| 4 | | | | | | | | | 4 | | | | | | | | | | | | 984938 | | | | | | | | | | | | rs139024319 | | | | | | | | | | | | G | | | | | | | | A | | | | | | SLC26A1 | | | | | | | | | | | | | | D | | | | | | | | | 3.62 | | | | | | | | | | | | | | | 1.52 | | | | | | | | | | | | | 528 | | | | | | | | | | | | | 464 | | | | | | | | | | | | | 3 | | | | | | | | | | | | | | 4 | | | | | | | | | | | | 4 | | | | | | | | | | | | | | | 0.002 | | | | | | | | | | | |
| 4 | | | | | | | | | 5 | | | | | | | | | | | | 106716975 | | | | | | | | | | | | rs201008479 | | | | | | | | | | | | G | | | | | | | | A | | | | | | EFNA5 | | | | | | | | | | | | | | D | | | | | | | | | 2.41 | | | | | | | | | | | | | | | . | | | | | | | | | | | | | 132 | | | | | | | | | | | | | 116 | | | | | | | | | | | | | 0 | | | | | | | | | | | | | | 4 | | | | | | | | | | | | 4 | | | | | | | | | | | | | | | 0.006 | | | | | | | | | | | |
| 4 | | | | | | | | | 5 | | | | | | | | | | | | 118862922 | | | | | | | | | | | | rs190659146 | | | | | | | | | | | | T | | | | | | | | C | | | | | | HSD17B4 | | | | | | | | | | | | | | . | | | | | | | | | 3.62 | | | | | | | | | | | | | | | . | | | | | | | | | | | | | 132 | | | | | | | | | | | | | 116 | | | | | | | | | | | | | 0 | | | | | | | | | | | | | | 4 | | | | | | | | | | | | 4 | | | | | | | | | | | | | | | 0.005 | | | | | | | | | | | |
| 4 | | | | | | | | | 5 | | | | | | | | | | | | 130815199 | | | | | | | | | | | | rs187240567 | | | | | | | | | | | | T | | | | | | | | C | | | | | | RAPGEF6 | | | | | | | | | | | | | | . | | | | | | | | | 3.62 | | | | | | | | | | | | | | | 2.28 | | | | | | | | | | | | | 264 | | | | | | | | | | | | | 232 | | | | | | | | | | | | | 1 | | | | | | | | | | | | | | 2 | | | | | | | | | | | | 2 | | | | | | | | | | | | | | | 0.001 | | | | | | | | | | | |
| 4 | | | | | | | | | 5 | | | | | | | | | | | | 130841167 | | | | | | | | | | | | rs201819833 | | | | | | | | | | | | G | | | | | | | | A | | | | | | RAPGEF6 | | | | | | | | | | | | | | D | | | | | | | | | 3.62 | | | | | | | | | | | | | | | 2.28 | | | | | | | | | | | | | 264 | | | | | | | | | | | | | 232 | | | | | | | | | | | | | 1 | | | | | | | | | | | | | | 2 | | | | | | | | | | | | 2 | | | | | | | | | | | | | | | 0.002 | | | | | | | | | | | |
| 4 | | | | | | | | | 5 | | | | | | | | | | | | 134102599 | | | | | | | | | | | | rs200408238 | | | | | | | | | | | | C | | | | | | | | G | | | | | | DDX46 | | | | | | | | | | | | | | . | | | | | | | | | 2.41 | | | | | | | | | | | | | | | 2.28 | | | | | | | | | | | | | 264 | | | | | | | | | | | | | 232 | | | | | | | | | | | | | 2 | | | | | | | | | | | | | | 4 | | | | | | | | | | | | 4 | | | | | | | | | | | | | | | 0.001 | | | | | | | | | | | |
| 4 | | | | | | | | | 5 | | | | | | | | | | | | 134143635 | | | | | | | | | | | | rs200296518 | | | | | | | | | | | | A | | | | | | | | G | | | | | | DDX46 | | | | | | | | | | | | | | . | | | | | | | | | 2.41 | | | | | | | | | | | | | | | 2.28 | | | | | | | | | | | | | 264 | | | | | | | | | | | | | 232 | | | | | | | | | | | | | 2 | | | | | | | | | | | | | | 4 | | | | | | | | | | | | 4 | | | | | | | | | | | | | | | 0.0069 | | | | | | | | | | | |
| 4 | | | | | | | | | 5 | | | | | | | | | | | | 137506607 | | | | | | | | | | | | rs191895585 | | | | | | | | | | | | G | | | | | | | | A | | | | | | BRD8 | | | | | | | | | | | | | | . | | | | | | | | | 3.62 | | | | | | | | | | | | | | | 2.28 | | | | | | | | | | | | | 132 | | | | | | | | | | | | | 116 | | | | | | | | | | | | | 1 | | | | | | | | | | | | | | 2 | | | | | | | | | | | | 2 | | | | | | | | | | | | | | | 0.005 | | | | | | | | | | | |
| 4 | | | | | | | | | 5 | | | | | | | | | | | | 1495038 | | | | | | | | | | | | rs201521332 | | | | | | | | | | | | G | | | | | | | | A | | | | | | LPCAT1 | | | | | | | | | | | | | | . | | | | | | | | | 5.53 | | | | | | | | | | | | | | | . | | | | | | | | | | | | | 126 | | | | | | | | | | | | | 114 | | | | | | | | | | | | | 0 | | | | | | | | | | | | | | 3 | | | | | | | | | | | | 3 | | | | | | | | | | | | | | | 0.0069 | | | | | | | | | | | |
| 4 | | | | | | | | | 5 | | | | | | | | | | | | 156675967 | | | | | | | | | | | | rs34482255 | | | | | | | | | | | | C | | | | | | | | T | | | | | | ITK | | | | | | | | | | | | | | D | | | | | | | | | 1.81 | | | | | | | | | | | | | | | 2.28 | | | | | | | | | | | | | 132 | | | | | | | | | | | | | 116 | | | | | | | | | | | | | 1 | | | | | | | | | | | | | | 2 | | | | | | | | | | | | 2 | | | | | | | | | | | | | | | 0.005 | | | | | | | | | | | |
| 4 | | | | | | | | | 5 | | | | | | | | | | | | 180477285 | | | | | | | | | | | | rs200884524 | | | | | | | | | | | | C | | | | | | | | T | | | | | | BTNL9 | | | | | | | | | | | | | | . | | | | | | | | | 1.81 | | | | | | | | | | | | | | | 1.71 | | | | | | | | | | | | | 264 | | | | | | | | | | | | | 232 | | | | | | | | | | | | | 2 | | | | | | | | | | | | | | 3 | | | | | | | | | | | | 3 | | | | | | | | | | | | | | | 0.001 | | | | | | | | | | | |
| 4 | | | | | | | | | 5 | | | | | | | | | | | | 180483533 | | | | | | | | | | | | rs373494500 | | | | | | | | | | | | T | | | | | | | | C | | | | | | BTNL9 | | | | | | | | | | | | | | . | | | | | | | | | 1.81 | | | | | | | | | | | | | | | 1.71 | | | | | | | | | | | | | 264 | | | | | | | | | | | | | 232 | | | | | | | | | | | | | 2 | | | | | | | | | | | | | | 3 | | | | | | | | | | | | 3 | | | | | | | | | | | | | | | 0.0069 | | | | | | | | | | | |
| 4 | | | | | | | | | 5 | | | | | | | | | | | | 38337618 | | | | | | | | | | | | rs376475358 | | | | | | | | | | | | G | | | | | | | | A | | | | | | EGFLAM | | | | | | | | | | | | | | . | | | | | | | | | 3.62 | | | | | | | | | | | | | | | 2.28 | | | | | | | | | | | | | 264 | | | | | | | | | | | | | 232 | | | | | | | | | | | | | 1 | | | | | | | | | | | | | | 2 | | | | | | | | | | | | 2 | | | | | | | | | | | | | | | 0.003 | | | | | | | | | | | |
| group | | | | | | | | | chr | | | | | | | | | | | | Pos | | | | | | | | | | | | id | | | | | | | | | | | | ref | | | | | | | | alt | | | | | | gene | | | | | | | | | | | | | | LR | | | | | | | | | **Gene burden ratio** | | | | | | | | | | | | | | | | | | | | | | | | | | | | **Total No. of alleles**  **in gene** | | | | | | | | | | | | | | | | | | | | | | | | | | **No. alt alleles**  **in genes** | | | | | | | | | | | | | | | | | | | | | | | | | | **No. of**  **cases with**  **alt alleles** | | | | | | | | | | | | | | | **Variant allele frequency** | | | | | | | | | | | |
|  | | | | | | | | |  | | | | | | | | | | | |  | | | | | | | | | | | |  | | | | | | | | | | | |  | | | | | | | |  | | | | | |  | | | | | | | | | | | | | |  | | | | | | | | | KG  East Asia | | | | | | | | | | | | | | | healthy  Ctrl | | | | | | | | | | | | | healthy  Ctrl | | | | | | | | | | | | | RA | | | | | | | | | | | | | healthy Ctrl | | | | | | | | | | | | | | RA | | | | | | | | | | | |  | | | | | | | | | | | | | | | KG  East Asia | | | | | | | | | | | |
| 4 | | | | | | | | | 5 | | | | | | | | | | | | 38425202 | | | | | | | | | | | | rs201409353 | | | | | | | | | | | | A | | | | | | | | G | | | | | | EGFLAM | | | | | | | | | | | | | | . | | | | | | | | | 3.62 | | | | | | | | | | | | | | | 2.28 | | | | | | | | | | | | | 264 | | | | | | | | | | | | | 232 | | | | | | | | | | | | | 1 | | | | | | | | | | | | | | 2 | | | | | | | | | | | | 2 | | | | | | | | | | | | | | | 0.001 | | | | | | | | | | | |
| 4 | | | | | | | | | 5 | | | | | | | | | | | | 43675682 | | | | | | | | | | | | rs80011859 | | | | | | | | | | | | C | | | | | | | | A | | | | | | NNT | | | | | | | | | | | | | | D | | | | | | | | | 3.62 | | | | | | | | | | | | | | | 2.28 | | | | | | | | | | | | | 264 | | | | | | | | | | | | | 232 | | | | | | | | | | | | | 1 | | | | | | | | | | | | | | 2 | | | | | | | | | | | | 2 | | | | | | | | | | | | | | | 0.002 | | | | | | | | | | | |
| 4 | | | | | | | | | 5 | | | | | | | | | | | | 43677914 | | | | | | | | | | | | rs144007922 | | | | | | | | | | | | C | | | | | | | | T | | | | | | NNT | | | | | | | | | | | | | | . | | | | | | | | | 3.62 | | | | | | | | | | | | | | | 2.28 | | | | | | | | | | | | | 264 | | | | | | | | | | | | | 232 | | | | | | | | | | | | | 1 | | | | | | | | | | | | | | 2 | | | | | | | | | | | | 2 | | | | | | | | | | | | | | | 0.004 | | | | | | | | | | | |
| 4 | | | | | | | | | 5 | | | | | | | | | | | | 94245090 | | | | | | | | | | | | rs543643285 | | | | | | | | | | | | G | | | | | | | | GA | | | | | | MCTP1 | | | | | | | | | | | | | | . | | | | | | | | | 11.05 | | | | | | | | | | | | | | | 2.28 | | | | | | | | | | | | | 260 | | | | | | | | | | | | | 228 | | | | | | | | | | | | | 6 | | | | | | | | | | | | | | 12 | | | | | | | | | | | | 12 | | | | | | | | | | | | | | | 0.001 | | | | | | | | | | | |
| 4 | | | | | | | | | 5 | | | | | | | | | | | | 94259730 | | | | | | | | | | | | rs555638017 | | | | | | | | | | | | GAAAC | | | | | | | | G | | | | | | MCTP1 | | | | | | | | | | | | | | . | | | | | | | | | 11.05 | | | | | | | | | | | | | | | 2.28 | | | | | | | | | | | | | 260 | | | | | | | | | | | | | 228 | | | | | | | | | | | | | 6 | | | | | | | | | | | | | | 12 | | | | | | | | | | | | 12 | | | | | | | | | | | | | | | 0.006 | | | | | | | | | | | |
| 4 | | | | | | | | | 5 | | | | | | | | | | | | 95226800 | | | | | | | | | | | | rs560418641 | | | | | | | | | | | | C | | | | | | | | T | | | | | | ELL2 | | | | | | | | | | | | | | . | | | | | | | | | 1.81 | | | | | | | | | | | | | | | 2.28 | | | | | | | | | | | | | 264 | | | | | | | | | | | | | 232 | | | | | | | | | | | | | 1 | | | | | | | | | | | | | | 2 | | | | | | | | | | | | 2 | | | | | | | | | | | | | | | 0.001 | | | | | | | | | | | |
| 4 | | | | | | | | | 5 | | | | | | | | | | | | 95278698 | | | | | | | | | | | | rs74836108 | | | | | | | | | | | | G | | | | | | | | A | | | | | | ELL2 | | | | | | | | | | | | | | . | | | | | | | | | 1.81 | | | | | | | | | | | | | | | 2.28 | | | | | | | | | | | | | 264 | | | | | | | | | | | | | 232 | | | | | | | | | | | | | 1 | | | | | | | | | | | | | | 2 | | | | | | | | | | | | 2 | | | | | | | | | | | | | | | 0.0069 | | | | | | | | | | | |
| 4 | | | | | | | | | 6 | | | | | | | | | | | | 106960382 | | | | | | | | | | | | rs201789082 | | | | | | | | | | | | G | | | | | | | | C | | | | | | AIM1 | | | | | | | | | | | | | | D | | | | | | | | | 3.62 | | | | | | | | | | | | | | | 2.28 | | | | | | | | | | | | | 396 | | | | | | | | | | | | | 348 | | | | | | | | | | | | | 1 | | | | | | | | | | | | | | 2 | | | | | | | | | | | | 2 | | | | | | | | | | | | | | | 0.005 | | | | | | | | | | | |
| 4 | | | | | | | | | 6 | | | | | | | | | | | | 106968092 | | | | | | | | | | | | rs371163103 | | | | | | | | | | | | TC | | | | | | | | T | | | | | | AIM1 | | | | | | | | | | | | | | . | | | | | | | | | 3.62 | | | | | | | | | | | | | | | 2.28 | | | | | | | | | | | | | 396 | | | | | | | | | | | | | 348 | | | | | | | | | | | | | 1 | | | | | | | | | | | | | | 2 | | | | | | | | | | | | 2 | | | | | | | | | | | | | | | 0.003 | | | | | | | | | | | |
| 4 | | | | | | | | | 6 | | | | | | | | | | | | 106991468 | | | | | | | | | | | | rs147230945 | | | | | | | | | | | | G | | | | | | | | A | | | | | | AIM1 | | | | | | | | | | | | | | D | | | | | | | | | 3.62 | | | | | | | | | | | | | | | 2.28 | | | | | | | | | | | | | 396 | | | | | | | | | | | | | 348 | | | | | | | | | | | | | 1 | | | | | | | | | | | | | | 2 | | | | | | | | | | | | 2 | | | | | | | | | | | | | | | 0.001 | | | | | | | | | | | |
| 4 | | | | | | | | | 6 | | | | | | | | | | | | 110636694 | | | | | | | | | | | | rs141656597 | | | | | | | | | | | | T | | | | | | | | C | | | | | | METTL24 | | | | | | | | | | | | | | . | | | | | | | | | 1.81 | | | | | | | | | | | | | | | 2.28 | | | | | | | | | | | | | 132 | | | | | | | | | | | | | 116 | | | | | | | | | | | | | 1 | | | | | | | | | | | | | | 2 | | | | | | | | | | | | 2 | | | | | | | | | | | | | | | 0.0099 | | | | | | | | | | | |
| 4 | | | | | | | | | 6 | | | | | | | | | | | | 127899925 | | | | | | | | | | | | rs141247870 | | | | | | | | | | | | C | | | | | | | | T | | | | | | C6orf58 | | | | | | | | | | | | | | . | | | | | | | | | 5.43 | | | | | | | | | | | | | | | 3.41 | | | | | | | | | | | | | 132 | | | | | | | | | | | | | 116 | | | | | | | | | | | | | 1 | | | | | | | | | | | | | | 3 | | | | | | | | | | | | 3 | | | | | | | | | | | | | | | 0.0079 | | | | | | | | | | | |
| 4 | | | | | | | | | 6 | | | | | | | | | | | | 147014059 | | | | | | | | | | | | rs192310446 | | | | | | | | | | | | T | | | | | | | | C | | | | | | ADGB | | | | | | | | | | | | | | . | | | | | | | | | 1.81 | | | | | | | | | | | | | | | . | | | | | | | | | | | | | 132 | | | | | | | | | | | | | 116 | | | | | | | | | | | | | 0 | | | | | | | | | | | | | | 3 | | | | | | | | | | | | 3 | | | | | | | | | | | | | | | 0.006 | | | | | | | | | | | |
| 4 | | | | | | | | | 6 | | | | | | | | | | | | 149959701 | | | | | | | | | | | | rs375809770 | | | | | | | | | | | | AAAG | | | | | | | | A | | | | | | KATNA1 | | | | | | | | | | | | | | . | | | | | | | | | 5.43 | | | | | | | | | | | | | | | . | | | | | | | | | | | | | 132 | | | | | | | | | | | | | 116 | | | | | | | | | | | | | 0 | | | | | | | | | | | | | | 3 | | | | | | | | | | | | 3 | | | | | | | | | | | | | | | 0.005 | | | | | | | | | | | |
| 4 | | | | | | | | | 6 | | | | | | | | | | | | 158454680 | | | | | | | | | | | | rs147847428 | | | | | | | | | | | | G | | | | | | | | A | | | | | | SYNJ2 | | | | | | | | | | | | | | D | | | | | | | | | 2.41 | | | | | | | | | | | | | | | 4.55 | | | | | | | | | | | | | 264 | | | | | | | | | | | | | 232 | | | | | | | | | | | | | 1 | | | | | | | | | | | | | | 4 | | | | | | | | | | | | 4 | | | | | | | | | | | | | | | 0.0079 | | | | | | | | | | | |
| 4 | | | | | | | | | 6 | | | | | | | | | | | | 158516905 | | | | | | | | | | | | rs202164985 | | | | | | | | | | | | C | | | | | | | | G | | | | | | SYNJ2 | | | | | | | | | | | | | | D | | | | | | | | | 2.41 | | | | | | | | | | | | | | | 4.55 | | | | | | | | | | | | | 264 | | | | | | | | | | | | | 232 | | | | | | | | | | | | | 1 | | | | | | | | | | | | | | 4 | | | | | | | | | | | | 4 | | | | | | | | | | | | | | | 0.001 | | | | | | | | | | | |
| 4 | | | | | | | | | 6 | | | | | | | | | | | | 20113198 | | | | | | | | | | | | rs199830796 | | | | | | | | | | | | G | | | | | | | | A | | | | | | MBOAT1 | | | | | | | | | | | | | | D | | | | | | | | | 5.43 | | | | | | | | | | | | | | | . | | | | | | | | | | | | | 264 | | | | | | | | | | | | | 232 | | | | | | | | | | | | | 0 | | | | | | | | | | | | | | 3 | | | | | | | | | | | | 3 | | | | | | | | | | | | | | | 0.004 | | | | | | | | | | | |
| 4 | | | | | | | | | 6 | | | | | | | | | | | | 20118693 | | | | | | | | | | | | rs553353326 | | | | | | | | | | | | G | | | | | | | | A | | | | | | MBOAT1 | | | | | | | | | | | | | | D | | | | | | | | | 5.43 | | | | | | | | | | | | | | | . | | | | | | | | | | | | | 264 | | | | | | | | | | | | | 232 | | | | | | | | | | | | | 0 | | | | | | | | | | | | | | 3 | | | | | | | | | | | | 3 | | | | | | | | | | | | | | | 0.001 | | | | | | | | | | | |
| group | | | | | | | | | chr | | | | | | | | | | | | Pos | | | | | | | | | | | | id | | | | | | | | | | | | ref | | | | | | | | alt | | | | | | gene | | | | | | | | | | | | | | LR | | | | | | | | | **Gene burden ratio** | | | | | | | | | | | | | | | | | | | | | | | | | | | | **Total No. of alleles**  **in gene** | | | | | | | | | | | | | | | | | | | | | | | | | | **No. alt alleles**  **in genes** | | | | | | | | | | | | | | | | | | | | | | | | | | **No. of**  **cases with**  **alt alleles** | | | | | | | | | | | | | | | **Variant allele frequency** | | | | | | | | | | | |
|  | | | | | | | | |  | | | | | | | | | | | |  | | | | | | | | | | | |  | | | | | | | | | | | |  | | | | | | | |  | | | | | |  | | | | | | | | | | | | | |  | | | | | | | | | KG  East Asia | | | | | | | | | | | | | | | healthy  Ctrl | | | | | | | | | | | | | healthy  Ctrl | | | | | | | | | | | | | RA | | | | | | | | | | | | | healthy Ctrl | | | | | | | | | | | | | | RA | | | | | | | | | | | |  | | | | | | | | | | | | | | | KG  East Asia | | | | | | | | | | | |
| 4 | | | | | | | | | 6 | | | | | | | | | | | | 33134570 | | | | | | | | | | | | . | | | | | | | | | | | | G | | | | | | | | A | | | | | | COL11A2 | | | | | | | | | | | | | | D | | | | | | | | | 1.81 | | | | | | | | | | | | | | | 3.79 | | | | | | | | | | | | | 660 | | | | | | | | | | | | | 580 | | | | | | | | | | | | | 3 | | | | | | | | | | | | | | 10 | | | | | | | | | | | | 10 | | | | | | | | | | | | | | | 0.006 | | | | | | | | | | | |
| 4 | | | | | | | | | 6 | | | | | | | | | | | | 33138929 | | | | | | | | | | | | . | | | | | | | | | | | | G | | | | | | | | A | | | | | | COL11A2 | | | | | | | | | | | | | | D | | | | | | | | | 1.81 | | | | | | | | | | | | | | | 3.79 | | | | | | | | | | | | | 660 | | | | | | | | | | | | | 580 | | | | | | | | | | | | | 3 | | | | | | | | | | | | | | 10 | | | | | | | | | | | | 10 | | | | | | | | | | | | | | | 0.0099 | | | | | | | | | | | |
| 4 | | | | | | | | | 6 | | | | | | | | | | | | 33146518 | | | | | | | | | | | | . | | | | | | | | | | | | A | | | | | | | | C | | | | | | COL11A2 | | | | | | | | | | | | | | . | | | | | | | | | 1.81 | | | | | | | | | | | | | | | 3.79 | | | | | | | | | | | | | 660 | | | | | | | | | | | | | 580 | | | | | | | | | | | | | 3 | | | | | | | | | | | | | | 10 | | | | | | | | | | | | 10 | | | | | | | | | | | | | | | 0.004 | | | | | | | | | | | |
| 4 | | | | | | | | | 6 | | | | | | | | | | | | 33147564 | | | | | | | | | | | | . | | | | | | | | | | | | C | | | | | | | | A | | | | | | COL11A2 | | | | | | | | | | | | | | D | | | | | | | | | 1.81 | | | | | | | | | | | | | | | 3.79 | | | | | | | | | | | | | 660 | | | | | | | | | | | | | 580 | | | | | | | | | | | | | 3 | | | | | | | | | | | | | | 10 | | | | | | | | | | | | 10 | | | | | | | | | | | | | | | 0.001 | | | | | | | | | | | |
| 4 | | | | | | | | | 6 | | | | | | | | | | | | 33154514 | | | | | | | | | | | | . | | | | | | | | | | | | C | | | | | | | | A | | | | | | COL11A2 | | | | | | | | | | | | | | D | | | | | | | | | 1.81 | | | | | | | | | | | | | | | 3.79 | | | | | | | | | | | | | 660 | | | | | | | | | | | | | 580 | | | | | | | | | | | | | 3 | | | | | | | | | | | | | | 10 | | | | | | | | | | | | 10 | | | | | | | | | | | | | | | 0.0089 | | | | | | | | | | | |
| 4 | | | | | | | | | 6 | | | | | | | | | | | | 35438350 | | | | | | | | | | | | rs187631484 | | | | | | | | | | | | C | | | | | | | | T | | | | | | MIR7111 | | | | | | | | | | | | | | . | | | | | | | | | 2.72 | | | | | | | | | | | | | | | 1.71 | | | | | | | | | | | | | 132 | | | | | | | | | | | | | 116 | | | | | | | | | | | | | 2 | | | | | | | | | | | | | | 3 | | | | | | | | | | | | 3 | | | | | | | | | | | | | | | 0.0069 | | | | | | | | | | | |
| 4 | | | | | | | | | 6 | | | | | | | | | | | | 35438350 | | | | | | | | | | | | rs187631484 | | | | | | | | | | | | C | | | | | | | | T | | | | | | RPL10A | | | | | | | | | | | | | | . | | | | | | | | | 2.72 | | | | | | | | | | | | | | | 1.71 | | | | | | | | | | | | | 132 | | | | | | | | | | | | | 116 | | | | | | | | | | | | | 2 | | | | | | | | | | | | | | 3 | | | | | | | | | | | | 3 | | | | | | | | | | | | | | | 0.0069 | | | | | | | | | | | |
| 4 | | | | | | | | | 6 | | | | | | | | | | | | 39883802 | | | | | | | | | | | | rs34757428 | | | | | | | | | | | | A | | | | | | | | T | | | | | | MOCS1 | | | | | | | | | | | | | | . | | | | | | | | | 5.73 | | | | | | | | | | | | | | | 3.6 | | | | | | | | | | | | | 132 | | | | | | | | | | | | | 110 | | | | | | | | | | | | | 1 | | | | | | | | | | | | | | 3 | | | | | | | | | | | | 3 | | | | | | | | | | | | | | | 0.0069 | | | | | | | | | | | |
| 4 | | | | | | | | | 6 | | | | | | | | | | | | 42933047 | | | | | | | | | | | | rs187435179 | | | | | | | | | | | | A | | | | | | | | G | | | | | | PEX6 | | | | | | | | | | | | | | D | | | | | | | | | 3.62 | | | | | | | | | | | | | | | 2.28 | | | | | | | | | | | | | 132 | | | | | | | | | | | | | 116 | | | | | | | | | | | | | 1 | | | | | | | | | | | | | | 2 | | | | | | | | | | | | 2 | | | | | | | | | | | | | | | 0.0089 | | | | | | | | | | | |
| 4 | | | | | | | | | 6 | | | | | | | | | | | | 43160731 | | | | | | | | | | | | rs568565110 | | | | | | | | | | | | C | | | | | | | | G | | | | | | CUL9 | | | | | | | | | | | | | | . | | | | | | | | | 5.43 | | | | | | | | | | | | | | | . | | | | | | | | | | | | | 396 | | | | | | | | | | | | | 348 | | | | | | | | | | | | | 0 | | | | | | | | | | | | | | 3 | | | | | | | | | | | | 3 | | | | | | | | | | | | | | | 0.001 | | | | | | | | | | | |
| 4 | | | | | | | | | 6 | | | | | | | | | | | | 43170522 | | | | | | | | | | | | rs200509434 | | | | | | | | | | | | G | | | | | | | | T | | | | | | CUL9 | | | | | | | | | | | | | | D | | | | | | | | | 5.43 | | | | | | | | | | | | | | | . | | | | | | | | | | | | | 396 | | | | | | | | | | | | | 348 | | | | | | | | | | | | | 0 | | | | | | | | | | | | | | 3 | | | | | | | | | | | | 3 | | | | | | | | | | | | | | | 0.0069 | | | | | | | | | | | |
| 4 | | | | | | | | | 6 | | | | | | | | | | | | 43172581 | | | | | | | | | | | | rs80345623 | | | | | | | | | | | | G | | | | | | | | A | | | | | | CUL9 | | | | | | | | | | | | | | D | | | | | | | | | 5.43 | | | | | | | | | | | | | | | . | | | | | | | | | | | | | 396 | | | | | | | | | | | | | 348 | | | | | | | | | | | | | 0 | | | | | | | | | | | | | | 3 | | | | | | | | | | | | 3 | | | | | | | | | | | | | | | 0.0099 | | | | | | | | | | | |
| 4 | | | | | | | | | 6 | | | | | | | | | | | | 49416648 | | | | | | | | | | | | rs199555550 | | | | | | | | | | | | G | | | | | | | | A | | | | | | MUT | | | | | | | | | | | | | | . | | | | | | | | | 9.05 | | | | | | | | | | | | | | | 5.32 | | | | | | | | | | | | | 370 | | | | | | | | | | | | | 348 | | | | | | | | | | | | | 1 | | | | | | | | | | | | | | 5 | | | | | | | | | | | | 5 | | | | | | | | | | | | | | | 0.0079 | | | | | | | | | | | |
| 4 | | | | | | | | | 6 | | | | | | | | | | | | 49425591 | | | | | | | | | | | | rs200908035 | | | | | | | | | | | | T | | | | | | | | C | | | | | | MUT | | | | | | | | | | | | | | D | | | | | | | | | 9.05 | | | | | | | | | | | | | | | 5.32 | | | | | | | | | | | | | 370 | | | | | | | | | | | | | 348 | | | | | | | | | | | | | 1 | | | | | | | | | | | | | | 5 | | | | | | | | | | | | 5 | | | | | | | | | | | | | | | 0.0079 | | | | | | | | | | | |
| 4 | | | | | | | | | 6 | | | | | | | | | | | | 49425720 | | | | | | | | | | | | rs528689712 | | | | | | | | | | | | T | | | | | | | | C | | | | | | MUT | | | | | | | | | | | | | | D | | | | | | | | | 9.05 | | | | | | | | | | | | | | | 5.32 | | | | | | | | | | | | | 370 | | | | | | | | | | | | | 348 | | | | | | | | | | | | | 1 | | | | | | | | | | | | | | 5 | | | | | | | | | | | | 5 | | | | | | | | | | | | | | | 0.001 | | | | | | | | | | | |
| 4 | | | | | | | | | 6 | | | | | | | | | | | | 72678681 | | | | | | | | | | | | rs532652925 | | | | | | | | | | | | C | | | | | | | | G | | | | | | RIMS1 | | | | | | | | | | | | | | . | | | | | | | | | 1.81 | | | | | | | | | | | | | | | 2.28 | | | | | | | | | | | | | 264 | | | | | | | | | | | | | 232 | | | | | | | | | | | | | 1 | | | | | | | | | | | | | | 2 | | | | | | | | | | | | 2 | | | | | | | | | | | | | | | 0.001 | | | | | | | | | | | |
| 4 | | | | | | | | | 6 | | | | | | | | | | | | 72974764 | | | | | | | | | | | | rs564292772 | | | | | | | | | | | | C | | | | | | | | A | | | | | | RIMS1 | | | | | | | | | | | | | | . | | | | | | | | | 1.81 | | | | | | | | | | | | | | | 2.28 | | | | | | | | | | | | | 264 | | | | | | | | | | | | | 232 | | | | | | | | | | | | | 1 | | | | | | | | | | | | | | 2 | | | | | | | | | | | | 2 | | | | | | | | | | | | | | | 0.003 | | | | | | | | | | | |
| 4 | | | | | | | | | 6 | | | | | | | | | | | | 76540124 | | | | | | | | | | | | rs187811833 | | | | | | | | | | | | C | | | | | | | | T | | | | | | MYO6 | | | | | | | | | | | | | | . | | | | | | | | | 1.81 | | | | | | | | | | | | | | | . | | | | | | | | | | | | | 130 | | | | | | | | | | | | | 116 | | | | | | | | | | | | | 0 | | | | | | | | | | | | | | 3 | | | | | | | | | | | | 3 | | | | | | | | | | | | | | | 0.0089 | | | | | | | | | | | |
| group | | | | | | chr | | | | | | | | | | | | Pos | | | | | | | | | | | | id | | | | | | | | | | | | ref | | | | | | | | | | | | | alt | | | | | | | | gene | | | | | | | | | | | | | LR | | | | | | | | | | | **Gene burden ratio** | | | | | | | | | | | | | | | | | | | | | | | | | | **Total No. of alleles**  **in gene** | | | | | | | | | | | | | | | | | | | | | | | | | | | **No. alt alleles**  **in genes** | | | | | | | | | | | | | | | | | | | | | | | | | | **No. of**  **cases with**  **alt alleles** | | | | | | | | | | | | | | **Variant allele frequency** | | | | | | | | |
|  | | | | | |  | | | | | | | | | | | |  | | | | | | | | | | | |  | | | | | | | | | | | |  | | | | | | | | | | | | |  | | | | | | | |  | | | | | | | | | | | | |  | | | | | | | | | | | KG  East Asia | | | | | | | | | | | | | healthy  Ctrl | | | | | | | | | | | | | healthy  Ctrl | | | | | | | | | | | | | RA | | | | | | | | | | | | | healthy Ctrl | | | | | | | | | | | | | | RA | | | | | | | | | | | |  | | | | | | | | | | | | | | KG  East Asia | | | | | | | | | |
| 4 | | | | | | 6 | | | | | | | | | | | | 89977391 | | | | | | | | | | | | rs146618576 | | | | | | | | | | | | T | | | | | | | | | | | | | C | | | | | | | | GABRR2 | | | | | | | | | | | | | . | | | | | | | | | | | 7.24 | | | | | | | | | | | | | 3.03 | | | | | | | | | | | | | 396 | | | | | | | | | | | | | 348 | | | | | | | | | | | | | 3 | | | | | | | | | | | | | | 8 | | | | | | | | | | | | 8 | | | | | | | | | | | | | | 0.002 | | | | | | | | | |
| 4 | | | | | | 6 | | | | | | | | | | | | 89977789 | | | | | | | | | | | | rs141423190 | | | | | | | | | | | | A | | | | | | | | | | | | | C | | | | | | | | GABRR2 | | | | | | | | | | | | | D | | | | | | | | | | | 7.24 | | | | | | | | | | | | | 3.03 | | | | | | | | | | | | | 396 | | | | | | | | | | | | | 348 | | | | | | | | | | | | | 3 | | | | | | | | | | | | | | 8 | | | | | | | | | | | | 8 | | | | | | | | | | | | | | 0.0079 | | | | | | | | | |
| 4 | | | | | | 6 | | | | | | | | | | | | 89978946 | | | | | | | | | | | | rs188424932 | | | | | | | | | | | | G | | | | | | | | | | | | | A | | | | | | | | GABRR2 | | | | | | | | | | | | | D | | | | | | | | | | | 7.24 | | | | | | | | | | | | | 3.03 | | | | | | | | | | | | | 396 | | | | | | | | | | | | | 348 | | | | | | | | | | | | | 3 | | | | | | | | | | | | | | 8 | | | | | | | | | | | | 8 | | | | | | | | | | | | | | 0.003 | | | | | | | | | |
| 4 | | | | | | 7 | | | | | | | | | | | | 100357429 | | | | | | | | | | | | rs374243234 | | | | | | | | | | | | C | | | | | | | | | | | | | T | | | | | | | | ZAN | | | | | | | | | | | | | . | | | | | | | | | | | 1.81 | | | | | | | | | | | | | 3.41 | | | | | | | | | | | | | 396 | | | | | | | | | | | | | 348 | | | | | | | | | | | | | 1 | | | | | | | | | | | | | | 3 | | | | | | | | | | | | 3 | | | | | | | | | | | | | | 0.001 | | | | | | | | | |
| 4 | | | | | | 7 | | | | | | | | | | | | 100363045 | | | | | | | | | | | | rs184742914 | | | | | | | | | | | | A | | | | | | | | | | | | | T | | | | | | | | ZAN | | | | | | | | | | | | | . | | | | | | | | | | | 1.81 | | | | | | | | | | | | | 3.41 | | | | | | | | | | | | | 396 | | | | | | | | | | | | | 348 | | | | | | | | | | | | | 1 | | | | | | | | | | | | | | 3 | | | | | | | | | | | | 3 | | | | | | | | | | | | | | 0.003 | | | | | | | | | |
| 4 | | | | | | 7 | | | | | | | | | | | | 100389715 | | | | | | | | | | | | rs369936309 | | | | | | | | | | | | C | | | | | | | | | | | | | T | | | | | | | | ZAN | | | | | | | | | | | | | D | | | | | | | | | | | 1.81 | | | | | | | | | | | | | 3.41 | | | | | | | | | | | | | 396 | | | | | | | | | | | | | 348 | | | | | | | | | | | | | 1 | | | | | | | | | | | | | | 3 | | | | | | | | | | | | 3 | | | | | | | | | | | | | | 0.003 | | | | | | | | | |
| 4 | | | | | | 7 | | | | | | | | | | | | 100656178 | | | | | | | | | | | | rs74570695 | | | | | | | | | | | | G | | | | | | | | | | | | | A | | | | | | | | MUC12 | | | | | | | | | | | | | . | | | | | | | | | | | 2.72 | | | | | | | | | | | | | 3.41 | | | | | | | | | | | | | 132 | | | | | | | | | | | | | 116 | | | | | | | | | | | | | 1 | | | | | | | | | | | | | | 3 | | | | | | | | | | | | 3 | | | | | | | | | | | | | | 0.003 | | | | | | | | | |
| 4 | | | | | | 7 | | | | | | | | | | | | 117144344 | | | | | | | | | | | | rs1800073 | | | | | | | | | | | | C | | | | | | | | | | | | | T | | | | | | | | CFTR | | | | | | | | | | | | | D | | | | | | | | | | | 3.62 | | | | | | | | | | | | | 2.26 | | | | | | | | | | | | | 654 | | | | | | | | | | | | | 580 | | | | | | | | | | | | | 2 | | | | | | | | | | | | | | 4 | | | | | | | | | | | | 4 | | | | | | | | | | | | | | 0.002 | | | | | | | | | |
| 4 | | | | | | 7 | | | | | | | | | | | | 117171053 | | | | | | | | | | | | rs141723617 | | | | | | | | | | | | T | | | | | | | | | | | | | C | | | | | | | | CFTR | | | | | | | | | | | | | D | | | | | | | | | | | 3.62 | | | | | | | | | | | | | 2.26 | | | | | | | | | | | | | 654 | | | | | | | | | | | | | 580 | | | | | | | | | | | | | 2 | | | | | | | | | | | | | | 4 | | | | | | | | | | | | 4 | | | | | | | | | | | | | | 0.0079 | | | | | | | | | |
| 4 | | | | | | 7 | | | | | | | | | | | | 117199578 | | | | | | | | | | | | rs138427145 | | | | | | | | | | | | A | | | | | | | | | | | | | T | | | | | | | | CFTR | | | | | | | | | | | | | D | | | | | | | | | | | 3.62 | | | | | | | | | | | | | 2.26 | | | | | | | | | | | | | 654 | | | | | | | | | | | | | 580 | | | | | | | | | | | | | 2 | | | | | | | | | | | | | | 4 | | | | | | | | | | | | 4 | | | | | | | | | | | | | | 0.001 | | | | | | | | | |
| 4 | | | | | | 7 | | | | | | | | | | | | 117235045 | | | | | | | | | | | | rs397508395 | | | | | | | | | | | | G | | | | | | | | | | | | | A | | | | | | | | CFTR | | | | | | | | | | | | | D | | | | | | | | | | | 3.62 | | | | | | | | | | | | | 2.26 | | | | | | | | | | | | | 654 | | | | | | | | | | | | | 580 | | | | | | | | | | | | | 2 | | | | | | | | | | | | | | 4 | | | | | | | | | | | | 4 | | | | | | | | | | | | | | 0.001 | | | | | | | | | |
| 4 | | | | | | 7 | | | | | | | | | | | | 117307076 | | | | | | | | | | | | rs4148725 | | | | | | | | | | | | C | | | | | | | | | | | | | T | | | | | | | | CFTR | | | | | | | | | | | | | D | | | | | | | | | | | 3.62 | | | | | | | | | | | | | 2.26 | | | | | | | | | | | | | 654 | | | | | | | | | | | | | 580 | | | | | | | | | | | | | 2 | | | | | | | | | | | | | | 4 | | | | | | | | | | | | 4 | | | | | | | | | | | | | | 0.006 | | | | | | | | | |
| 4 | | | | | | 7 | | | | | | | | | | | | 131888058 | | | | | | | | | | | | rs143605398 | | | | | | | | | | | | TGCTAGGC  CCCAGCC | | | | | | | | | | | | | T | | | | | | | | PLXNA4 | | | | | | | | | | | | | . | | | | | | | | | | | 1.81 | | | | | | | | | | | | | 2.28 | | | | | | | | | | | | | 264 | | | | | | | | | | | | | 232 | | | | | | | | | | | | | 1 | | | | | | | | | | | | | | 2 | | | | | | | | | | | | 2 | | | | | | | | | | | | | | 0.0099 | | | | | | | | | |
| 4 | | | | | | 7 | | | | | | | | | | | | 131888117 | | | | | | | | | | | | rs181597184 | | | | | | | | | | | | C | | | | | | | | | | | | | T | | | | | | | | PLXNA4 | | | | | | | | | | | | | D | | | | | | | | | | | 1.81 | | | | | | | | | | | | | 2.28 | | | | | | | | | | | | | 264 | | | | | | | | | | | | | 232 | | | | | | | | | | | | | 1 | | | | | | | | | | | | | | 2 | | | | | | | | | | | | 2 | | | | | | | | | | | | | | 0.003 | | | | | | | | | |
| 4 | | | | | | 7 | | | | | | | | | | | | 139260038 | | | | | | | | | | | | rs56338252 | | | | | | | | | | | | T | | | | | | | | | | | | | C | | | | | | | | HIPK2 | | | | | | | | | | | | | . | | | | | | | | | | | 2.41 | | | | | | | | | | | | | 2.28 | | | | | | | | | | | | | 264 | | | | | | | | | | | | | 232 | | | | | | | | | | | | | 2 | | | | | | | | | | | | | | 4 | | | | | | | | | | | | 3 | | | | | | | | | | | | | | 0.003 | | | | | | | | | |
| 4 | | | | | | 7 | | | | | | | | | | | | 143017768 | | | | | | | | | | | | rs201509501 | | | | | | | | | | | | C | | | | | | | | | | | | | T | | | | | | | | CLCN1 | | | | | | | | | | | | | D | | | | | | | | | | | 5.43 | | | | | | | | | | | | | 3.41 | | | | | | | | | | | | | 264 | | | | | | | | | | | | | 232 | | | | | | | | | | | | | 1 | | | | | | | | | | | | | | 3 | | | | | | | | | | | | 3 | | | | | | | | | | | | | | 0.002 | | | | | | | | | |
| 4 | | | | | | 7 | | | | | | | | | | | | 143029550 | | | | | | | | | | | | rs202119213 | | | | | | | | | | | | C | | | | | | | | | | | | | T | | | | | | | | CLCN1 | | | | | | | | | | | | | D | | | | | | | | | | | 5.43 | | | | | | | | | | | | | 3.41 | | | | | | | | | | | | | 264 | | | | | | | | | | | | | 232 | | | | | | | | | | | | | 1 | | | | | | | | | | | | | | 3 | | | | | | | | | | | | 3 | | | | | | | | | | | | | | 0.003 | | | | | | | | | |
| group | | | | | | | | | | | | chr | | | | | | | | | | | | Pos | | | | | | | | | | | | id | | | | | | | | | | | | ref | | | | | | | | | | | | | | | | alt | | | | | | | gene | | | | | | | | | | | | | | | | | LR | | | | | | **Gene burden ratio** | | | | | | | | | | | | | | | | | | | | | | | | | **Total No. of alleles**  **in gene** | | | | | | | | | | | | | | | | | | | | | | | | | | | **No. alt alleles**  **in genes** | | | | | | | | | | | | | | | | | | | | | | | | | **No. of**  **cases with**  **alt alleles** | | | | | | | | | | | | | | **Variant allele frequency** | | | |
|  | | | | | | | | | | | |  | | | | | | | | | | | |  | | | | | | | | | | | |  | | | | | | | | | | | |  | | | | | | | | | | | | | | | |  | | | | | | |  | | | | | | | | | | | | | | | | |  | | | | | | KG  East Asia | | | | | | | | | | | | healthy  Ctrl | | | | | | | | | | | | | | healthy  Ctrl | | | | | | | | | | | | RA | | | | | | | | | | | | | | | healthy Ctrl | | | | | | | | | | | RA | | | | | | | | | | | | | |  | | | | | | | | | | | | | | KG  East Asia | | |
| 4 | | | | | | 7 | | | | | | | | | | | | 26678881 | | | | | | | | | | | | rs78525926 | | | | | | | | | | | | G | | | | | | | | | | | | | A | | | | | | | | C7orf71 | | | | | | | | | | | | | . | | | | | | | | | | | 3.62 | | | | | | | | | | | | | . | | | | | | | | | | | | | 264 | | | | | | | | | | | | | 232 | | | | | | | | | | | | | 0 | | | | | | | | | | | | | | 4 | | | | | | | | | | | | 4 | | | | | | | | | | | | | | 0.004 | | | | | | | | | |
| 4 | | | | | | 7 | | | | | | | | | | | | 26678910 | | | | | | | | | | | | rs111516571 | | | | | | | | | | | | C | | | | | | | | | | | | | T | | | | | | | | C7orf71 | | | | | | | | | | | | | . | | | | | | | | | | | 3.62 | | | | | | | | | | | | | . | | | | | | | | | | | | | 264 | | | | | | | | | | | | | 232 | | | | | | | | | | | | | 0 | | | | | | | | | | | | | | 4 | | | | | | | | | | | | 4 | | | | | | | | | | | | | | 0.004 | | | | | | | | | |
| 4 | | | | | | 7 | | | | | | | | | | | | 29535662 | | | | | | | | | | | | rs3815512 | | | | | | | | | | | | G | | | | | | | | | | | | | C | | | | | | | | CHN2 | | | | | | | | | | | | | . | | | | | | | | | | | 3.62 | | | | | | | | | | | | | 2.28 | | | | | | | | | | | | | 132 | | | | | | | | | | | | | 116 | | | | | | | | | | | | | 1 | | | | | | | | | | | | | | 2 | | | | | | | | | | | | 2 | | | | | | | | | | | | | | 0.003 | | | | | | | | | |
| 4 | | | | | | 7 | | | | | | | | | | | | 47342658 | | | | | | | | | | | | rs192974657 | | | | | | | | | | | | G | | | | | | | | | | | | | A | | | | | | | | TNS3 | | | | | | | | | | | | | D | | | | | | | | | | | 3.62 | | | | | | | | | | | | | 2.28 | | | | | | | | | | | | | 264 | | | | | | | | | | | | | 232 | | | | | | | | | | | | | 1 | | | | | | | | | | | | | | 2 | | | | | | | | | | | | 2 | | | | | | | | | | | | | | 0.002 | | | | | | | | | |
| 4 | | | | | | 7 | | | | | | | | | | | | 47454718 | | | | | | | | | | | | rs187456873 | | | | | | | | | | | | C | | | | | | | | | | | | | T | | | | | | | | TNS3 | | | | | | | | | | | | | D | | | | | | | | | | | 3.62 | | | | | | | | | | | | | 2.28 | | | | | | | | | | | | | 264 | | | | | | | | | | | | | 232 | | | | | | | | | | | | | 1 | | | | | | | | | | | | | | 2 | | | | | | | | | | | | 2 | | | | | | | | | | | | | | 0.002 | | | | | | | | | |
| 4 | | | | | | 7 | | | | | | | | | | | | 75192236 | | | | | | | | | | | | . | | | | | | | | | | | | C | | | | | | | | | | | | | A | | | | | | | | HIP1 | | | | | | | | | | | | | . | | | | | | | | | | | 3.62 | | | | | | | | | | | | | 4.55 | | | | | | | | | | | | | 264 | | | | | | | | | | | | | 232 | | | | | | | | | | | | | 1 | | | | | | | | | | | | | | 4 | | | | | | | | | | | | 4 | | | | | | | | | | | | | | 0.0089 | | | | | | | | | |
| 4 | | | | | | 7 | | | | | | | | | | | | 75210547 | | | | | | | | | | | | . | | | | | | | | | | | | A | | | | | | | | | | | | | T | | | | | | | | HIP1 | | | | | | | | | | | | | . | | | | | | | | | | | 3.62 | | | | | | | | | | | | | 4.55 | | | | | | | | | | | | | 264 | | | | | | | | | | | | | 232 | | | | | | | | | | | | | 1 | | | | | | | | | | | | | | 4 | | | | | | | | | | | | 4 | | | | | | | | | | | | | | 0.001 | | | | | | | | | |
| 4 | | | | | | 8 | | | | | | | | | | | | 116426988 | | | | | | | | | | | | rs181035264 | | | | | | | | | | | | T | | | | | | | | | | | | | A | | | | | | | | TRPS1 | | | | | | | | | | | | | D | | | | | | | | | | | 5.43 | | | | | | | | | | | | | 1.71 | | | | | | | | | | | | | 264 | | | | | | | | | | | | | 232 | | | | | | | | | | | | | 2 | | | | | | | | | | | | | | 3 | | | | | | | | | | | | 3 | | | | | | | | | | | | | | 0.003 | | | | | | | | | |
| 4 | | | | | | 8 | | | | | | | | | | | | 116631783 | | | | | | | | | | | | rs202001185 | | | | | | | | | | | | T | | | | | | | | | | | | | C | | | | | | | | TRPS1 | | | | | | | | | | | | | D | | | | | | | | | | | 5.43 | | | | | | | | | | | | | 1.71 | | | | | | | | | | | | | 264 | | | | | | | | | | | | | 232 | | | | | | | | | | | | | 2 | | | | | | | | | | | | | | 3 | | | | | | | | | | | | 3 | | | | | | | | | | | | | | 0.004 | | | | | | | | | |
| 4 | | | | | | 8 | | | | | | | | | | | | 133900823 | | | | | | | | | | | | rs368037086 | | | | | | | | | | | | C | | | | | | | | | | | | | T | | | | | | | | TG | | | | | | | | | | | | | . | | | | | | | | | | | 1.84 | | | | | | | | | | | | | . | | | | | | | | | | | | | 260 | | | | | | | | | | | | | 228 | | | | | | | | | | | | | 0 | | | | | | | | | | | | | | 3 | | | | | | | | | | | | 3 | | | | | | | | | | | | | | 0.002 | | | | | | | | | |
| 4 | | | | | | 8 | | | | | | | | | | | | 133981695 | | | | | | | | | | | | rs189500765 | | | | | | | | | | | | C | | | | | | | | | | | | | T | | | | | | | | TG | | | | | | | | | | | | | . | | | | | | | | | | | 1.84 | | | | | | | | | | | | | . | | | | | | | | | | | | | 260 | | | | | | | | | | | | | 228 | | | | | | | | | | | | | 0 | | | | | | | | | | | | | | 3 | | | | | | | | | | | | 3 | | | | | | | | | | | | | | 0.004 | | | | | | | | | |
| 4 | | | | | | 8 | | | | | | | | | | | | 144895478 | | | | | | | | | | | | . | | | | | | | | | | | | C | | | | | | | | | | | | | A | | | | | | | | SCRIB | | | | | | | | | | | | | . | | | | | | | | | | | 1.81 | | | | | | | | | | | | | 4.55 | | | | | | | | | | | | | 132 | | | | | | | | | | | | | 116 | | | | | | | | | | | | | 1 | | | | | | | | | | | | | | 4 | | | | | | | | | | | | 4 | | | | | | | | | | | | | | 0.006 | | | | | | | | | |
| 4 | | | | | | 8 | | | | | | | | | | | | 145694225 | | | | | | | | | | | | rs193131687 | | | | | | | | | | | | C | | | | | | | | | | | | | T | | | | | | | | KIFC2 | | | | | | | | | | | | | . | | | | | | | | | | | 5.43 | | | | | | | | | | | | | . | | | | | | | | | | | | | 132 | | | | | | | | | | | | | 116 | | | | | | | | | | | | | 0 | | | | | | | | | | | | | | 3 | | | | | | | | | | | | 3 | | | | | | | | | | | | | | 0.003 | | | | | | | | | |
| 4 | | | | | | 8 | | | | | | | | | | | | 145736896 | | | | | | | | | | | | rs557256260 | | | | | | | | | | | | C | | | | | | | | | | | | | T | | | | | | | | RECQL4 | | | | | | | | | | | | | . | | | | | | | | | | | 1.81 | | | | | | | | | | | | | 1.71 | | | | | | | | | | | | | 660 | | | | | | | | | | | | | 580 | | | | | | | | | | | | | 4 | | | | | | | | | | | | | | 6 | | | | | | | | | | | | 6 | | | | | | | | | | | | | | 0.001 | | | | | | | | | |
| 4 | | | | | | 8 | | | | | | | | | | | | 145738985 | | | | | | | | | | | | rs536831548 | | | | | | | | | | | | G | | | | | | | | | | | | | C | | | | | | | | RECQL4 | | | | | | | | | | | | | . | | | | | | | | | | | 1.81 | | | | | | | | | | | | | 1.71 | | | | | | | | | | | | | 660 | | | | | | | | | | | | | 580 | | | | | | | | | | | | | 4 | | | | | | | | | | | | | | 6 | | | | | | | | | | | | 6 | | | | | | | | | | | | | | 0.001 | | | | | | | | | |
| 4 | | | | | | 8 | | | | | | | | | | | | 145741388 | | | | | | | | | | | | rs200097701 | | | | | | | | | | | | C | | | | | | | | | | | | | G | | | | | | | | RECQL4 | | | | | | | | | | | | | . | | | | | | | | | | | 1.81 | | | | | | | | | | | | | 1.71 | | | | | | | | | | | | | 660 | | | | | | | | | | | | | 580 | | | | | | | | | | | | | 4 | | | | | | | | | | | | | | 6 | | | | | | | | | | | | 6 | | | | | | | | | | | | | | 0.005 | | | | | | | | | |
| 4 | | | | | | 8 | | | | | | | | | | | | 145741602 | | | | | | | | | | | | rs34633809 | | | | | | | | | | | | C | | | | | | | | | | | | | T | | | | | | | | RECQL4 | | | | | | | | | | | | | . | | | | | | | | | | | 1.81 | | | | | | | | | | | | | 1.71 | | | | | | | | | | | | | 660 | | | | | | | | | | | | | 580 | | | | | | | | | | | | | 4 | | | | | | | | | | | | | | 6 | | | | | | | | | | | | 6 | | | | | | | | | | | | | | 0.0089 | | | | | | | | | |
| 4 | | | | | | 8 | | | | | | | | | | | | 145742799 | | | | | | | | | | | | rs34642881 | | | | | | | | | | | | T | | | | | | | | | | | | | C | | | | | | | | RECQL4 | | | | | | | | | | | | | . | | | | | | | | | | | 1.81 | | | | | | | | | | | | | 1.71 | | | | | | | | | | | | | 660 | | | | | | | | | | | | | 580 | | | | | | | | | | | | | 4 | | | | | | | | | | | | | | 6 | | | | | | | | | | | | 6 | | | | | | | | | | | | | | 0.0079 | | | | | | | | | |
| group | | | | | | | | | | | | chr | | | | | | | | | | | | Pos | | | | | | | | | | | | id | | | | | | | | | | | | ref | | | | | | | | | | | | | | | | alt | | | | | | | gene | | | | | | | | | | | | | | | | | LR | | | | | | **Gene burden ratio** | | | | | | | | | | | | | | | | | | | | | | | | | **Total No. of alleles**  **in gene** | | | | | | | | | | | | | | | | | | | | | | | | | | | **No. alt alleles**  **in genes** | | | | | | | | | | | | | | | | | | | | | | | | | **No. of**  **cases with**  **alt alleles** | | | | | | | | | | | | | | **Variant allele frequency** | | | |
|  | | | | | | | | | | | |  | | | | | | | | | | | |  | | | | | | | | | | | |  | | | | | | | | | | | |  | | | | | | | | | | | | | | | |  | | | | | | |  | | | | | | | | | | | | | | | | |  | | | | | | KG  East Asia | | | | | | | | | | | | healthy  Ctrl | | | | | | | | | | | | | | healthy  Ctrl | | | | | | | | | | | | RA | | | | | | | | | | | | | | | healthy Ctrl | | | | | | | | | | | RA | | | | | | | | | | | | | |  | | | | | | | | | | | | | | KG  East Asia | | |
| 4 | | | | | | | | | | | | 8 | | | | | | | | | | | | 17400906 | | | | | | | | | | | | rs12680645 | | | | | | | | | | | | G | | | | | | | | | | | | | | | | A | | | | | | | SLC7A2 | | | | | | | | | | | | | | | | | D | | | | | | 2.26 | | | | | | | | | | | | 2.84 | | | | | | | | | | | | | | 396 | | | | | | | | | | | | 348 | | | | | | | | | | | | | | | 2 | | | | | | | | | | | 5 | | | | | | | | | | | | | | 5 | | | | | | | | | | | | | | 0.0079 | | |
| 4 | | | | | | | | | | | | 8 | | | | | | | | | | | | 17407821 | | | | | | | | | | | | rs188973136 | | | | | | | | | | | | C | | | | | | | | | | | | | | | | G | | | | | | | SLC7A2 | | | | | | | | | | | | | | | | | D | | | | | | 2.26 | | | | | | | | | | | | 2.84 | | | | | | | | | | | | | | 396 | | | | | | | | | | | | 348 | | | | | | | | | | | | | | | 2 | | | | | | | | | | | 5 | | | | | | | | | | | | | | 5 | | | | | | | | | | | | | | 0.006 | | |
| 4 | | | | | | | | | | | | 8 | | | | | | | | | | | | 17417839 | | | | | | | | | | | | rs201373242 | | | | | | | | | | | | A | | | | | | | | | | | | | | | | G | | | | | | | SLC7A2 | | | | | | | | | | | | | | | | | D | | | | | | 2.26 | | | | | | | | | | | | 2.84 | | | | | | | | | | | | | | 396 | | | | | | | | | | | | 348 | | | | | | | | | | | | | | | 2 | | | | | | | | | | | 5 | | | | | | | | | | | | | | 5 | | | | | | | | | | | | | | 0.001 | | |
| 4 | | | | | | | | | | | | 8 | | | | | | | | | | | | 24771326 | | | | | | | | | | | | rs182011677 | | | | | | | | | | | | C | | | | | | | | | | | | | | | | G | | | | | | | NEFM | | | | | | | | | | | | | | | | | D | | | | | | 2.72 | | | | | | | | | | | | . | | | | | | | | | | | | | | 264 | | | | | | | | | | | | 232 | | | | | | | | | | | | | | | 0 | | | | | | | | | | | 3 | | | | | | | | | | | | | | 3 | | | | | | | | | | | | | | 0.004 | | |
| 4 | | | | | | | | | | | | 8 | | | | | | | | | | | | 24775743 | | | | | | | | | | | | rs557507354 | | | | | | | | | | | | C | | | | | | | | | | | | | | | | T | | | | | | | NEFM | | | | | | | | | | | | | | | | | D | | | | | | 2.72 | | | | | | | | | | | | . | | | | | | | | | | | | | | 264 | | | | | | | | | | | | 232 | | | | | | | | | | | | | | | 0 | | | | | | | | | | | 3 | | | | | | | | | | | | | | 3 | | | | | | | | | | | | | | 0.001 | | |
| 4 | | | | | | | | | | | | 8 | | | | | | | | | | | | 25293853 | | | | | | | | | | | | rs2271114 | | | | | | | | | | | | A | | | | | | | | | | | | | | | | G | | | | | | | KCTD9 | | | | | | | | | | | | | | | | | . | | | | | | 3.68 | | | | | | | | | | | | 2.25 | | | | | | | | | | | | | | 128 | | | | | | | | | | | | 114 | | | | | | | | | | | | | | | 1 | | | | | | | | | | | 2 | | | | | | | | | | | | | | 2 | | | | | | | | | | | | | | 0.005 | | |
| 4 | | | | | | | | | | | | 8 | | | | | | | | | | | | 41470357 | | | | | | | | | | | | rs372694683 | | | | | | | | | | | | C | | | | | | | | | | | | | | | | T | | | | | | | GPAT4 | | | | | | | | | | | | | | | | | . | | | | | | 3.62 | | | | | | | | | | | | 2.28 | | | | | | | | | | | | | | 132 | | | | | | | | | | | | 116 | | | | | | | | | | | | | | | 1 | | | | | | | | | | | 2 | | | | | | | | | | | | | | 2 | | | | | | | | | | | | | | 0.003 | | |
| 4 | | | | | | | | | | | | 8 | | | | | | | | | | | | 43155706 | | | | | | | | | | | | rs182905752 | | | | | | | | | | | | C | | | | | | | | | | | | | | | | T | | | | | | | POTEA | | | | | | | | | | | | | | | | | . | | | | | | 3.68 | | | | | | | | | | | | 2.23 | | | | | | | | | | | | | | 254 | | | | | | | | | | | | 228 | | | | | | | | | | | | | | | 1 | | | | | | | | | | | 2 | | | | | | | | | | | | | | 2 | | | | | | | | | | | | | | 0.001 | | |
| 4 | | | | | | | | | | | | 8 | | | | | | | | | | | | 43211970 | | | | | | | | | | | | rs534445172 | | | | | | | | | | | | A | | | | | | | | | | | | | | | | C | | | | | | | POTEA | | | | | | | | | | | | | | | | | . | | | | | | 3.68 | | | | | | | | | | | | 2.23 | | | | | | | | | | | | | | 254 | | | | | | | | | | | | 228 | | | | | | | | | | | | | | | 1 | | | | | | | | | | | 2 | | | | | | | | | | | | | | 2 | | | | | | | | | | | | | | 0.001 | | |
| 4 | | | | | | | | | | | | 8 | | | | | | | | | | | | 89086826 | | | | | | | | | | | | rs200104505 | | | | | | | | | | | | T | | | | | | | | | | | | | | | | A | | | | | | | MMP16 | | | | | | | | | | | | | | | | | . | | | | | | 3.62 | | | | | | | | | | | | 2.28 | | | | | | | | | | | | | | 132 | | | | | | | | | | | | 116 | | | | | | | | | | | | | | | 1 | | | | | | | | | | | 2 | | | | | | | | | | | | | | 2 | | | | | | | | | | | | | | 0.0099 | | |
| 4 | | | | | | | | | | | | 9 | | | | | | | | | | | | 136917570 | | | | | | | | | | | | rs200913664 | | | | | | | | | | | | G | | | | | | | | | | | | | | | | A | | | | | | | BRD3 | | | | | | | | | | | | | | | | | . | | | | | | 3.62 | | | | | | | | | | | | 2.28 | | | | | | | | | | | | | | 132 | | | | | | | | | | | | 116 | | | | | | | | | | | | | | | 1 | | | | | | | | | | | 2 | | | | | | | | | | | | | | 2 | | | | | | | | | | | | | | 0.0069 | | |
| 4 | | | | | | | | | | | | 9 | | | | | | | | | | | | 138235867 | | | | | | | | | | | | rs531171616 | | | | | | | | | | | | G | | | | | | | | | | | | | | | | A | | | | | | | C9orf62 | | | | | | | | | | | | | | | | | . | | | | | | 3.62 | | | | | | | | | | | | 2.28 | | | | | | | | | | | | | | 264 | | | | | | | | | | | | 232 | | | | | | | | | | | | | | | 1 | | | | | | | | | | | 2 | | | | | | | | | | | | | | 2 | | | | | | | | | | | | | | 0.001 | | |
| 4 | | | | | | | | | | | | 9 | | | | | | | | | | | | 138236230 | | | | | | | | | | | | rs560393458 | | | | | | | | | | | | T | | | | | | | | | | | | | | | | G | | | | | | | C9orf62 | | | | | | | | | | | | | | | | | . | | | | | | 3.62 | | | | | | | | | | | | 2.28 | | | | | | | | | | | | | | 264 | | | | | | | | | | | | 232 | | | | | | | | | | | | | | | 1 | | | | | | | | | | | 2 | | | | | | | | | | | | | | 2 | | | | | | | | | | | | | | 0.004 | | |
| 4 | | | | | | | | | | | | 9 | | | | | | | | | | | | 139008659 | | | | | | | | | | | | rs373479265 | | | | | | | | | | | | C | | | | | | | | | | | | | | | | T | | | | | | | C9orf69 | | | | | | | | | | | | | | | | | . | | | | | | 3.62 | | | | | | | | | | | | 2.28 | | | | | | | | | | | | | | 132 | | | | | | | | | | | | 116 | | | | | | | | | | | | | | | 1 | | | | | | | | | | | 2 | | | | | | | | | | | | | | 2 | | | | | | | | | | | | | | 0.003 | | |
| 4 | | | | | | | | | | | | 9 | | | | | | | | | | | | 139360706 | | | | | | | | | | | | rs189523223 | | | | | | | | | | | | C | | | | | | | | | | | | | | | | T | | | | | | | SEC16A | | | | | | | | | | | | | | | | | . | | | | | | 7.24 | | | | | | | | | | | | . | | | | | | | | | | | | | | 264 | | | | | | | | | | | | 232 | | | | | | | | | | | | | | | 0 | | | | | | | | | | | 4 | | | | | | | | | | | | | | 4 | | | | | | | | | | | | | | 0.004 | | |
| 4 | | | | | | | | | | | | 9 | | | | | | | | | | | | 139372145 | | | | | | | | | | | | rs192612248 | | | | | | | | | | | | G | | | | | | | | | | | | | | | | T | | | | | | | SEC16A | | | | | | | | | | | | | | | | | . | | | | | | 7.24 | | | | | | | | | | | | . | | | | | | | | | | | | | | 264 | | | | | | | | | | | | 232 | | | | | | | | | | | | | | | 0 | | | | | | | | | | | 4 | | | | | | | | | | | | | | 4 | | | | | | | | | | | | | | 0.005 | | |
| 4 | | | | | | | | | | | | 9 | | | | | | | | | | | | 15571614 | | | | | | | | | | | | rs182199324 | | | | | | | | | | | | T | | | | | | | | | | | | | | | | C | | | | | | | CCDC171 | | | | | | | | | | | | | | | | | . | | | | | | 3.62 | | | | | | | | | | | | 2.28 | | | | | | | | | | | | | | 396 | | | | | | | | | | | | 348 | | | | | | | | | | | | | | | 1 | | | | | | | | | | | 2 | | | | | | | | | | | | | | 2 | | | | | | | | | | | | | | 0.002 | | |
| 4 | | | | | | | | | | | | 9 | | | | | | | | | | | | 15745503 | | | | | | | | | | | | rs530529615 | | | | | | | | | | | | A | | | | | | | | | | | | | | | | T | | | | | | | CCDC171 | | | | | | | | | | | | | | | | | . | | | | | | 3.62 | | | | | | | | | | | | 2.28 | | | | | | | | | | | | | | 396 | | | | | | | | | | | | 348 | | | | | | | | | | | | | | | 1 | | | | | | | | | | | 2 | | | | | | | | | | | | | | 2 | | | | | | | | | | | | | | 0.003 | | |
| group | | | | chr | | | | | | | | | | | pos | | | | | | | | | | | | | id | | | | | | | | | | | | ref | | | | | | | | | | | | | | | | | alt | | | | | | | | | | | | | gene | | | | | | | | | | | | | | | LR | | | | | | **Gene burden ratio** | | | | | | | | | | | | | | | | | | | | | | | | | **Total No. of alleles**  **in gene** | | | | | | | | | | | | | | | | | | | | | | | | | | | **No. alt alleles**  **in genes** | | | | | | | | | | | | | | | | | | | | | | | | | | | **No. of**  **cases with**  **alt alleles** | | | | | | | | | | | | | | **Variant allele frequency** | | | | |
|  | | | |  | | | | | | | | | | |  | | | | | | | | | | | | |  | | | | | | | | | | | |  | | | | | | | | | | | | | | | | |  | | | | | | | | | | | | |  | | | | | | | | | | | | | | |  | | | | | | KG  East Asia | | | | | | | | | | | | healthy  Ctrl | | | | | | | | | | | | | healthy  Ctrl | | | | | | | | | | | | | RA | | | | | | | | | | | | | | healthy Ctrl | | | | | | | | | | | | | RA | | | | | | | | | | | | | |  | | | | | | | | | | | | | | KG  East Asia | | | | |
| 4 | | | | | | | | | | | | 9 | | | | | | | | | | | | 15777828 | | | | | | | | | | | | rs202139088 | | | | | | | | | | | | T | | | | | | | | | | | | | | | | G | | | | | | | CCDC171 | | | | | | | | | | | | | | | | | . | | | | | | 3.62 | | | | | | | | | | | | 2.28 | | | | | | | | | | | | | | 396 | | | | | | | | | | | | 348 | | | | | | | | | | | | | | | 1 | | | | | | | | | | | 2 | | | | | | | | | | | | | | 2 | | | | | | | | | | | | | | 0.002 | | |
| 4 | | | | | | | | | | | | 9 | | | | | | | | | | | | 439392 | | | | | | | | | | | | rs117109271 | | | | | | | | | | | | A | | | | | | | | | | | | | | | | G | | | | | | | DOCK8 | | | | | | | | | | | | | | | | | . | | | | | | 1.81 | | | | | | | | | | | | 1.52 | | | | | | | | | | | | | | 264 | | | | | | | | | | | | 232 | | | | | | | | | | | | | | | 3 | | | | | | | | | | | 4 | | | | | | | | | | | | | | 4 | | | | | | | | | | | | | | 0.0099 | | |
| 4 | | | | | | | | | | | | 9 | | | | | | | | | | | | 441423 | | | | | | | | | | | | rs188141951 | | | | | | | | | | | | C | | | | | | | | | | | | | | | | T | | | | | | | DOCK8 | | | | | | | | | | | | | | | | | . | | | | | | 1.81 | | | | | | | | | | | | 1.52 | | | | | | | | | | | | | | 264 | | | | | | | | | | | | 232 | | | | | | | | | | | | | | | 3 | | | | | | | | | | | 4 | | | | | | | | | | | | | | 4 | | | | | | | | | | | | | | 0.003 | | |
| 4 | | | | | | | | | | | | 9 | | | | | | | | | | | | 5968714 | | | | | | | | | | | | rs183413824 | | | | | | | | | | | | C | | | | | | | | | | | | | | | | T | | | | | | | KIAA2026 | | | | | | | | | | | | | | | | | D | | | | | | 3.62 | | | | | | | | | | | | 2.28 | | | | | | | | | | | | | | 132 | | | | | | | | | | | | 116 | | | | | | | | | | | | | | | 1 | | | | | | | | | | | 2 | | | | | | | | | | | | | | 2 | | | | | | | | | | | | | | 0.005 | | |
| 4 | | | | | | | | | | | | 9 | | | | | | | | | | | | 72517162 | | | | | | | | | | | | rs12344550 | | | | | | | | | | | | T | | | | | | | | | | | | | | | | C | | | | | | | C9orf135 | | | | | | | | | | | | | | | | | . | | | | | | 3.62 | | | | | | | | | | | | . | | | | | | | | | | | | | | 264 | | | | | | | | | | | | 232 | | | | | | | | | | | | | | | 0 | | | | | | | | | | | 4 | | | | | | | | | | | | | | 4 | | | | | | | | | | | | | | 0.005 | | |
| 4 | | | | | | | | | | | | 9 | | | | | | | | | | | | 72517293 | | | | | | | | | | | | rs11140833 | | | | | | | | | | | | G | | | | | | | | | | | | | | | | T | | | | | | | C9orf135 | | | | | | | | | | | | | | | | | . | | | | | | 3.62 | | | | | | | | | | | | . | | | | | | | | | | | | | | 264 | | | | | | | | | | | | 232 | | | | | | | | | | | | | | | 0 | | | | | | | | | | | 4 | | | | | | | | | | | | | | 4 | | | | | | | | | | | | | | 0.005 | | |
| 4 | | | | | | | | | | | | X | | | | | | | | | | | | 8759354 | | | | | | | | | | | | rs768509456 | | | | | | | | | | | | A | | | | | | | | | | | | | | | | T | | | | | | | FAM9A | | | | | | | | | | | | | | | | | . | | | | | | 3.69 | | | | | | | | | | | | 4.56 | | | | | | | | | | | | | | 262 | | | | | | | | | | | | 230 | | | | | | | | | | | | | | | 1 | | | | | | | | | | | 4 | | | | | | | | | | | | | | 4 | | | | | | | | | | | | | | 0.0013 | | |
| 4 | | | | | | | | | | | | X | | | | | | | | | | | | 8763309 | | | | | | | | | | | | rs774169916 | | | | | | | | | | | | GCTGCTGCTG  CTGCGGCTT | | | | | | | | | | | | | | | | G | | | | | | | FAM9A | | | | | | | | | | | | | | | | | . | | | | | | 3.69 | | | | | | | | | | | | 4.56 | | | | | | | | | | | | | | 262 | | | | | | | | | | | | 230 | | | | | | | | | | | | | | | 1 | | | | | | | | | | | 4 | | | | | | | | | | | | | | 4 | | | | | | | | | | | | | | 0.0026 | | |
| 4 | | | | | | | | | | | | X | | | | | | | | | | | | 96136645 | | | | | | | | | | | | rs778550013 | | | | | | | | | | | | C | | | | | | | | | | | | | | | | T | | | | | | | DIAPH2 | | | | | | | | | | | | | | | | | D | | | | | | 3.66 | | | | | | | | | | | | 2.24 | | | | | | | | | | | | | | 260 | | | | | | | | | | | | 232 | | | | | | | | | | | | | | | 2 | | | | | | | | | | | 4 | | | | | | | | | | | | | | 3 | | | | | | | | | | | | | | 0.0013 | | |
| 5 | | | | | | | | | | | | 11 | | | | | | | | | | | | 17394037 | | | | | | | | | | | | rs61406813 | | | | | | | | | | | | CTT | | | | | | | | | | | | | | | | C | | | | | | | NCR3LG1 | | | | | | | | | | | | | | | | | . | | | | | | 5.43 | | | | | | | | | | | | 3.41 | | | | | | | | | | | | | | 132 | | | | | | | | | | | | 116 | | | | | | | | | | | | | | | 2 | | | | | | | | | | | 6 | | | | | | | | | | | | | | 5 | | | | | | | | | | | | | | 0.0089 | | |
| 5 | | | | | | | | | | | | 1 | | | | | | | | | | | | 21926063 | | | | | | | | | | | | rs61014678 | | | | | | | | | | | | C | | | | | | | | | | | | | | | | T | | | | | | | RAP1GAP | | | | | | | | | | | | | | | | | D | | | | | | 7.24 | | | | | | | | | | | | 4.55 | | | | | | | | | | | | | | 264 | | | | | | | | | | | | 232 | | | | | | | | | | | | | | | 2 | | | | | | | | | | | 8 | | | | | | | | | | | | | | 7 | | | | | | | | | | | | | | 0.0069 | | |
| 5 | | | | | | | | | | | | 2 | | | | | | | | | | | | 113342071 | | | | | | | | | | | | rs528909726 | | | | | | | | | | | | G | | | | | | | | | | | | | | | | A | | | | | | | CHCHD5 | | | | | | | | | | | | | | | | | . | | | | | | . | | | | | | | | | | | | . | | | | | | | | | | | | | | 124 | | | | | | | | | | | | 90 | | | | | | | | | | | | | | | 0 | | | | | | | | | | | 2 | | | | | | | | | | | | | | 1 | | | | | | | | | | | | | | 0.002 | | |
| 5 | | | | | | | | | | | | 7 | | | | | | | | | | | | 139285351 | | | | | | | | | | | | rs3735196 | | | | | | | | | | | | C | | | | | | | | | | | | | | | | G | | | | | | | HIPK2 | | | | | | | | | | | | | | | | | . | | | | | | 2.41 | | | | | | | | | | | | 2.28 | | | | | | | | | | | | | | 264 | | | | | | | | | | | | 232 | | | | | | | | | | | | | | | 2 | | | | | | | | | | | 4 | | | | | | | | | | | | | | 3 | | | | | | | | | | | | | | 0.0089 | | |
| 5 | | | | | | | | | | | | X | | | | | | | | | | | | 96396659 | | | | | | | | | | | | rs363755 | | | | | | | | | | | | C | | | | | | | | | | | | | | | | T | | | | | | | DIAPH2 | | | | | | | | | | | | | | | | | . | | | | | | 3.66 | | | | | | | | | | | | 2.24 | | | | | | | | | | | | | | 260 | | | | | | | | | | | | 232 | | | | | | | | | | | | | | | 2 | | | | | | | | | | | 4 | | | | | | | | | | | | | | 3 | | | | | | | | | | | | | | 0.0052 | | |
| 6 | | | | | | | | | | | | 10 | | | | | | | | | | | | 126714641 | | | | | | | | | | | | rs12571821 | | | | | | | | | | | | G | | | | | | | | | | | | | | | | C | | | | | | | CTBP2 | | | | | | | | | | | | | | | | | . | | | | | | 1.81 | | | | | | | | | | | | 1.52 | | | | | | | | | | | | | | 132 | | | | | | | | | | | | 116 | | | | | | | | | | | | | | | 3 | | | | | | | | | | | 4 | | | | | | | | | | | | | | 4 | | | | | | | | | | | | | | 0.0446 | | |
| 6 | | | | | | | | | | | | 10 | | | | | | | | | | | | 28970433 | | | | | | | | | | | | rs79472556 | | | | | | | | | | | | G | | | | | | | | | | | | | | | | C | | | | | | | BAMBI | | | | | | | | | | | | | | | | | D | | | | | | 1.81 | | | | | | | | | | | | 2.28 | | | | | | | | | | | | | | 264 | | | | | | | | | | | | 232 | | | | | | | | | | | | | | | 1 | | | | | | | | | | | 2 | | | | | | | | | | | | | | 2 | | | | | | | | | | | | | | 0.0159 | | |
| 6 | | | | 10 | | | | | | | | | | | 28970914 | | | | | | | | | | | | | rs750723477 | | | | | | | | | | | | C | | | | | | | | | | | | | | | | | T | | | | | | | | | | | | | BAMBI | | | | | | | | | | | | | | | . | | | | | | 1.81 | | | | | | | | | | | | 2.28 | | | | | | | | | | | | | 264 | | | | | | | | | | | | | 232 | | | | | | | | | | | | | | 1 | | | | | | | | | | | | | 2 | | | | | | | | | | | | | | 2 | | | | | | | | | | | | | | . | | | | |
| group | | | | chr | | | | | | | | | | | pos | | | | | | | | | | | | | id | | | | | | | | | | | | ref | | | | | | | | | | | | | | | | | alt | | | | | | | | | | | | | gene | | | | | | | | | | | | | | | LR | | | | | | **Gene burden ratio** | | | | | | | | | | | | | | | | | | | | | | | | | **Total No. of alleles**  **in gene** | | | | | | | | | | | | | | | | | | | | | | | | | | | **No. alt alleles**  **in genes** | | | | | | | | | | | | | | | | | | | | | | | | | | | **No. of**  **cases with**  **alt alleles** | | | | | | | | | | | | | | **Variant allele frequency** | | | | |
|  | | | |  | | | | | | | | | | |  | | | | | | | | | | | | |  | | | | | | | | | | | |  | | | | | | | | | | | | | | | | |  | | | | | | | | | | | | |  | | | | | | | | | | | | | | |  | | | | | | KG  East Asia | | | | | | | | | | | | healthy  Ctrl | | | | | | | | | | | | | healthy  Ctrl | | | | | | | | | | | | | RA | | | | | | | | | | | | | | healthy Ctrl | | | | | | | | | | | | | RA | | | | | | | | | | | | | |  | | | | | | | | | | | | | | KG  East Asia | | | | |
| 6 | | | | 10 | | | | | | | | | | | 61956386 | | | | | | | | | | | | | rs34796699 | | | | | | | | | | | | GA | | | | | | | | | | | | | | | | | G | | | | | | | | | | | | | ANK3 | | | | | | | | | | | | | | | . | | | | | | 2.45 | | | | | | | | | | | | 1.6 | | | | | | | | | | | | | 240 | | | | | | | | | | | | | 236 | | | | | | | | | | | | | | 14 | | | | | | | | | | | | | 22 | | | | | | | | | | | | | | 19 | | | | | | | | | | | | | | . | | | | |
| 6 | | | | 10 | | | | | | | | | | | 61956386 | | | | | | | | | | | | | rs772920191 | | | | | | | | | | | | G | | | | | | | | | | | | | | | | | GAAA | | | | | | | | | | | | | ANK3 | | | | | | | | | | | | | | | . | | | | | | 2.45 | | | | | | | | | | | | 1.6 | | | | | | | | | | | | | 240 | | | | | | | | | | | | | 236 | | | | | | | | | | | | | | 14 | | | | | | | | | | | | | 22 | | | | | | | | | | | | | | 19 | | | | | | | | | | | | | | . | | | | |
| 6 | | | | 10 | | | | | | | | | | | 61956386 | | | | | | | | | | | | | rs772920191 | | | | | | | | | | | | G | | | | | | | | | | | | | | | | | GA | | | | | | | | | | | | | ANK3 | | | | | | | | | | | | | | | . | | | | | | 2.45 | | | | | | | | | | | | 1.6 | | | | | | | | | | | | | 240 | | | | | | | | | | | | | 236 | | | | | | | | | | | | | | 14 | | | | | | | | | | | | | 22 | | | | | | | | | | | | | | 19 | | | | | | | | | | | | | | . | | | | |
| 6 | | | | 10 | | | | | | | | | | | 62023781 | | | | | | | | | | | | | rs144841334 | | | | | | | | | | | | G | | | | | | | | | | | | | | | | | A | | | | | | | | | | | | | ANK3 | | | | | | | | | | | | | | | . | | | | | | 2.45 | | | | | | | | | | | | 1.6 | | | | | | | | | | | | | 240 | | | | | | | | | | | | | 236 | | | | | | | | | | | | | | 14 | | | | | | | | | | | | | 22 | | | | | | | | | | | | | | 19 | | | | | | | | | | | | | | 0.0268 | | | | |
| 6 | | | | 10 | | | | | | | | | | | 81901943 | | | | | | | | | | | | | rs7080405 | | | | | | | | | | | | G | | | | | | | | | | | | | | | | | C | | | | | | | | | | | | | PLAC9 | | | | | | | | | | | | | | | . | | | | | | 3.62 | | | | | | | | | | | | 2.28 | | | | | | | | | | | | | 132 | | | | | | | | | | | | | 116 | | | | | | | | | | | | | | 1 | | | | | | | | | | | | | 2 | | | | | | | | | | | | | | 2 | | | | | | | | | | | | | | 0.0188 | | | | |
| 6 | | | | 10 | | | | | | | | | | | 81926637 | | | | | | | | | | | | | rs777020491 | | | | | | | | | | | | G | | | | | | | | | | | | | | | | | A | | | | | | | | | | | | | ANXA11 | | | | | | | | | | | | | | | . | | | | | | 2.72 | | | | | | | | | | | | 1.71 | | | | | | | | | | | | | 264 | | | | | | | | | | | | | 232 | | | | | | | | | | | | | | 2 | | | | | | | | | | | | | 3 | | | | | | | | | | | | | | 3 | | | | | | | | | | | | | | . | | | | |
| 6 | | | | 10 | | | | | | | | | | | 81926750 | | | | | | | | | | | | | rs34332933 | | | | | | | | | | | | G | | | | | | | | | | | | | | | | | C | | | | | | | | | | | | | ANXA11 | | | | | | | | | | | | | | | . | | | | | | 2.72 | | | | | | | | | | | | 1.71 | | | | | | | | | | | | | 264 | | | | | | | | | | | | | 232 | | | | | | | | | | | | | | 2 | | | | | | | | | | | | | 3 | | | | | | | | | | | | | | 3 | | | | | | | | | | | | | | 0.0188 | | | | |
| 6 | | | | 10 | | | | | | | | | | | 94822756 | | | | | | | | | | | | | rs58993699 | | | | | | | | | | | | C | | | | | | | | | | | | | | | | | T | | | | | | | | | | | | | CYP26C1 | | | | | | | | | | | | | | | . | | | | | | 2.41 | | | | | | | | | | | | 1.52 | | | | | | | | | | | | | 132 | | | | | | | | | | | | | 116 | | | | | | | | | | | | | | 3 | | | | | | | | | | | | | 4 | | | | | | | | | | | | | | 4 | | | | | | | | | | | | | | 0.0119 | | | | |
| 6 | | | | 10 | | | | | | | | | | | 94834060 | | | | | | | | | | | | | rs185421897 | | | | | | | | | | | | C | | | | | | | | | | | | | | | | | T | | | | | | | | | | | | | CYP26A1 | | | | | | | | | | | | | | | . | | | | | | 1.96 | | | | | | | | | | | | 2.11 | | | | | | | | | | | | | 396 | | | | | | | | | | | | | 348 | | | | | | | | | | | | | | 7 | | | | | | | | | | | | | 13 | | | | | | | | | | | | | | 11 | | | | | | | | | | | | | | 0.0139 | | | | |
| 6 | | | | 10 | | | | | | | | | | | 94835071 | | | | | | | | | | | | | rs80188100 | | | | | | | | | | | | A | | | | | | | | | | | | | | | | | G | | | | | | | | | | | | | CYP26A1 | | | | | | | | | | | | | | | . | | | | | | 1.96 | | | | | | | | | | | | 2.11 | | | | | | | | | | | | | 396 | | | | | | | | | | | | | 348 | | | | | | | | | | | | | | 7 | | | | | | | | | | | | | 13 | | | | | | | | | | | | | | 11 | | | | | | | | | | | | | | 0.0119 | | | | |
| 6 | | | | 10 | | | | | | | | | | | 94835072 | | | | | | | | | | | | | rs75053982 | | | | | | | | | | | | G | | | | | | | | | | | | | | | | | A | | | | | | | | | | | | | CYP26A1 | | | | | | | | | | | | | | | . | | | | | | 1.96 | | | | | | | | | | | | 2.11 | | | | | | | | | | | | | 396 | | | | | | | | | | | | | 348 | | | | | | | | | | | | | | 7 | | | | | | | | | | | | | 13 | | | | | | | | | | | | | | 11 | | | | | | | | | | | | | | 0.0119 | | | | |
| 6 | | | | 1 | | | | | | | | | | | 10042683 | | | | | | | | | | | | | rs138626416 | | | | | | | | | | | | G | | | | | | | | | | | | | | | | | A | | | | | | | | | | | | | NMNAT1 | | | | | | | | | | | | | | | D | | | | | | 1.81 | | | | | | | | | | | | . | | | | | | | | | | | | | 132 | | | | | | | | | | | | | 116 | | | | | | | | | | | | | | 0 | | | | | | | | | | | | | 3 | | | | | | | | | | | | | | 3 | | | | | | | | | | | | | | 0.0159 | | | | |
| 6 | | | | 11 | | | | | | | | | | | 121323228 | | | | | | | | | | | | | . | | | | | | | | | | | | G | | | | | | | | | | | | | | | | | A | | | | | | | | | | | | | SORL1 | | | | | | | | | | | | | | | D | | | | | | 7.24 | | | | | | | | | | | | 2.28 | | | | | | | | | | | | | 792 | | | | | | | | | | | | | 696 | | | | | | | | | | | | | | 2 | | | | | | | | | | | | | 4 | | | | | | | | | | | | | | 4 | | | | | | | | | | | | | | . | | | | |
| 6 | | | | 11 | | | | | | | | | | | 121430331 | | | | | | | | | | | | | . | | | | | | | | | | | | T | | | | | | | | | | | | | | | | | C | | | | | | | | | | | | | SORL1 | | | | | | | | | | | | | | | D | | | | | | 7.24 | | | | | | | | | | | | 2.28 | | | | | | | | | | | | | 792 | | | | | | | | | | | | | 696 | | | | | | | | | | | | | | 2 | | | | | | | | | | | | | 4 | | | | | | | | | | | | | | 4 | | | | | | | | | | | | | | . | | | | |
| 6 | | | | 11 | | | | | | | | | | | 121440905 | | | | | | | | | | | | | rs753319585 | | | | | | | | | | | | G | | | | | | | | | | | | | | | | | A | | | | | | | | | | | | | SORL1 | | | | | | | | | | | | | | | D | | | | | | 7.24 | | | | | | | | | | | | 2.28 | | | | | | | | | | | | | 792 | | | | | | | | | | | | | 696 | | | | | | | | | | | | | | 2 | | | | | | | | | | | | | 4 | | | | | | | | | | | | | | 4 | | | | | | | | | | | | | | . | | | | |
| 6 | | | | 11 | | | | | | | | | | | 121457048 | | | | | | | | | | | | | rs146197030 | | | | | | | | | | | | T | | | | | | | | | | | | | | | | | G | | | | | | | | | | | | | SORL1 | | | | | | | | | | | | | | | . | | | | | | 7.24 | | | | | | | | | | | | 2.28 | | | | | | | | | | | | | 792 | | | | | | | | | | | | | 696 | | | | | | | | | | | | | | 2 | | | | | | | | | | | | | 4 | | | | | | | | | | | | | | 4 | | | | | | | | | | | | | | . | | | | |
| 6 | | | | 11 | | | | | | | | | | | 121460027 | | | | | | | | | | | | | rs752525626 | | | | | | | | | | | | G | | | | | | | | | | | | | | | | | C | | | | | | | | | | | | | SORL1 | | | | | | | | | | | | | | | D | | | | | | 7.24 | | | | | | | | | | | | 2.28 | | | | | | | | | | | | | 792 | | | | | | | | | | | | | 696 | | | | | | | | | | | | | | 2 | | | | | | | | | | | | | 4 | | | | | | | | | | | | | | 4 | | | | | | | | | | | | | | . | | | | |
| 6 | | | | 11 | | | | | | | | | | | 121489543 | | | | | | | | | | | | | rs751110498 | | | | | | | | | | | | A | | | | | | | | | | | | | | | | | C | | | | | | | | | | | | | SORL1 | | | | | | | | | | | | | | | D | | | | | | 7.24 | | | | | | | | | | | | 2.28 | | | | | | | | | | | | | 792 | | | | | | | | | | | | | 696 | | | | | | | | | | | | | | 2 | | | | | | | | | | | | | 4 | | | | | | | | | | | | | | 4 | | | | | | | | | | | | | | . | | | | |
| group | | | | | | | | | | chr | | | | | | | | | | | | Pos | | | | | | | | | | | | id | | | | | | | | | | | | ref | | | | | | | alt | | | | | | gene | | | | | | | | | | | | | | LR | | | | | | | | | **Gene burden ratio** | | | | | | | | | | | | | | | | | | | | | | | | | | | | **Total No. of alleles**  **in gene** | | | | | | | | | | | | | | | | | | | | | | | | | | **No. alt alleles**  **in genes** | | | | | | | | | | | | | | | | | | | | | | | | | | **No. of**  **cases with**  **alt alleles** | | | | | | | | | | | | | | | **Variant allele frequency** | | | | | | | | | | | |
|  | | | | | | | | | |  | | | | | | | | | | | |  | | | | | | | | | | | |  | | | | | | | | | | | |  | | | | | | |  | | | | | |  | | | | | | | | | | | | | |  | | | | | | | | | KG  East Asia | | | | | | | | | | | | | | | healthy  Ctrl | | | | | | | | | | | | | healthy  Ctrl | | | | | | | | | | | | | RA | | | | | | | | | | | | | healthy Ctrl | | | | | | | | | | | | | | RA | | | | | | | | | | | |  | | | | | | | | | | | | | | | KG  East Asia | | | | | | | | | | | |
| 6 | | | | | | | | | | 1 | | | | | | | | | | | | 116933040 | | | | | | | | | | | | rs28622933 | | | | | | | | | | | | C | | | | | | | G | | | | | | ATP1A1 | | | | | | | | | | | | | | . | | | | | | | | | 2.41 | | | | | | | | | | | | | | | 2.28 | | | | | | | | | | | | | 132 | | | | | | | | | | | | | 116 | | | | | | | | | | | | | 4 | | | | | | | | | | | | | | 8 | | | | | | | | | | | | 8 | | | | | | | | | | | | | | | 0.0268 | | | | | | | | | | | |
| 6 | | | | | | | | | | 11 | | | | | | | | | | | | 22232870 | | | | | | | | | | | | rs78987921 | | | | | | | | | | | | G | | | | | | | A | | | | | | ANO5 | | | | | | | | | | | | | | . | | | | | | | | | 3.62 | | | | | | | | | | | | | | | 4.55 | | | | | | | | | | | | | 264 | | | | | | | | | | | | | 232 | | | | | | | | | | | | | 1 | | | | | | | | | | | | | | 4 | | | | | | | | | | | | 4 | | | | | | | | | | | | | | | 0.0109 | | | | | | | | | | | |
| 6 | | | | | | | | | | 11 | | | | | | | | | | | | 22239801 | | | | | | | | | | | | . | | | | | | | | | | | | C | | | | | | | T | | | | | | ANO5 | | | | | | | | | | | | | | . | | | | | | | | | 3.62 | | | | | | | | | | | | | | | 4.55 | | | | | | | | | | | | | 264 | | | | | | | | | | | | | 232 | | | | | | | | | | | | | 1 | | | | | | | | | | | | | | 4 | | | | | | | | | | | | 4 | | | | | | | | | | | | | | | . | | | | | | | | | | | |
| 6 | | | | | | | | | | 1 | | | | | | | | | | | | 145414790 | | | | | | | | | | | | . | | | | | | | | | | | | G | | | | | | | C | | | | | | HFE2 | | | | | | | | | | | | | | . | | | | | | | | | 2.72 | | | | | | | | | | | | | | | . | | | | | | | | | | | | | 132 | | | | | | | | | | | | | 116 | | | | | | | | | | | | | 0 | | | | | | | | | | | | | | 3 | | | | | | | | | | | | 3 | | | | | | | | | | | | | | | 0.0129 | | | | | | | | | | | |
| 6 | | | | | | | | | | 11 | | | | | | | | | | | | 487419 | | | | | | | | | | | | . | | | | | | | | | | | | G | | | | | | | A | | | | | | PTDSS2 | | | | | | | | | | | | | | . | | | | | | | | | 3.62 | | | | | | | | | | | | | | | 4.55 | | | | | | | | | | | | | 396 | | | | | | | | | | | | | 348 | | | | | | | | | | | | | 1 | | | | | | | | | | | | | | 4 | | | | | | | | | | | | 4 | | | | | | | | | | | | | | | . | | | | | | | | | | | |
| 6 | | | | | | | | | | 11 | | | | | | | | | | | | 489522 | | | | | | | | | | | | rs375041205 | | | | | | | | | | | | C | | | | | | | T | | | | | | PTDSS2 | | | | | | | | | | | | | | . | | | | | | | | | 3.62 | | | | | | | | | | | | | | | 4.55 | | | | | | | | | | | | | 396 | | | | | | | | | | | | | 348 | | | | | | | | | | | | | 1 | | | | | | | | | | | | | | 4 | | | | | | | | | | | | 4 | | | | | | | | | | | | | | | . | | | | | | | | | | | |
| 6 | | | | | | | | | | 11 | | | | | | | | | | | | 490077 | | | | | | | | | | | | rs374769797 | | | | | | | | | | | | G | | | | | | | A | | | | | | PTDSS2 | | | | | | | | | | | | | | . | | | | | | | | | 3.62 | | | | | | | | | | | | | | | 4.55 | | | | | | | | | | | | | 396 | | | | | | | | | | | | | 348 | | | | | | | | | | | | | 1 | | | | | | | | | | | | | | 4 | | | | | | | | | | | | 4 | | | | | | | | | | | | | | | . | | | | | | | | | | | |
| 6 | | | | | | | | | | 1 | | | | | | | | | | | | 15834360 | | | | | | | | | | | | rs2020902 | | | | | | | | | | | | A | | | | | | | G | | | | | | CASP9 | | | | | | | | | | | | | | . | | | | | | | | | 1.81 | | | | | | | | | | | | | | | 1.71 | | | | | | | | | | | | | 132 | | | | | | | | | | | | | 116 | | | | | | | | | | | | | 2 | | | | | | | | | | | | | | 3 | | | | | | | | | | | | 3 | | | | | | | | | | | | | | | 0.0317 | | | | | | | | | | | |
| 6 | | | | | | | | | | 1 | | | | | | | | | | | | 15860803 | | | | | | | | | | | | rs11583306 | | | | | | | | | | | | C | | | | | | | T | | | | | | DNAJC16 | | | | | | | | | | | | | | . | | | | | | | | | 2.17 | | | | | | | | | | | | | | | 3.41 | | | | | | | | | | | | | 132 | | | | | | | | | | | | | 116 | | | | | | | | | | | | | 2 | | | | | | | | | | | | | | 6 | | | | | | | | | | | | 6 | | | | | | | | | | | | | | | 0.0466 | | | | | | | | | | | |
| 6 | | | | | | | | | | 1 | | | | | | | | | | | | 159410340 | | | | | | | | | | | | rs12409540 | | | | | | | | | | | | T | | | | | | | A | | | | | | OR10J1 | | | | | | | | | | | | | | . | | | | | | | | | 1.51 | | | | | | | | | | | | | | | 5.69 | | | | | | | | | | | | | 132 | | | | | | | | | | | | | 116 | | | | | | | | | | | | | 1 | | | | | | | | | | | | | | 5 | | | | | | | | | | | | 5 | | | | | | | | | | | | | | | 0.0278 | | | | | | | | | | | |
| 6 | | | | | | | | | | 1 | | | | | | | | | | | | 160011511 | | | | | | | | | | | | rs3795339 | | | | | | | | | | | | C | | | | | | | T | | | | | | KCNJ10 | | | | | | | | | | | | | | D | | | | | | | | | 2.72 | | | | | | | | | | | | | | | 1.71 | | | | | | | | | | | | | 132 | | | | | | | | | | | | | 116 | | | | | | | | | | | | | 2 | | | | | | | | | | | | | | 3 | | | | | | | | | | | | 3 | | | | | | | | | | | | | | | 0.0129 | | | | | | | | | | | |
| 6 | | | | | | | | | | 11 | | | | | | | | | | | | 613605 | | | | | | | | | | | | . | | | | | | | | | | | | G | | | | | | | C | | | | | | IRF7 | | | | | | | | | | | | | | . | | | | | | | | | 1.58 | | | | | | | | | | | | | | | 2.65 | | | | | | | | | | | | | 526 | | | | | | | | | | | | | 464 | | | | | | | | | | | | | 3 | | | | | | | | | | | | | | 7 | | | | | | | | | | | | 7 | | | | | | | | | | | | | | | . | | | | | | | | | | | |
| 6 | | | | | | | | | | 11 | | | | | | | | | | | | 615010 | | | | | | | | | | | | rs12290989 | | | | | | | | | | | | G | | | | | | | T | | | | | | IRF7 | | | | | | | | | | | | | | . | | | | | | | | | 1.58 | | | | | | | | | | | | | | | 2.65 | | | | | | | | | | | | | 526 | | | | | | | | | | | | | 464 | | | | | | | | | | | | | 3 | | | | | | | | | | | | | | 7 | | | | | | | | | | | | 7 | | | | | | | | | | | | | | | 0.0208 | | | | | | | | | | | |
| 6 | | | | | | | | | | 11 | | | | | | | | | | | | 615011 | | | | | | | | | | | | rs12272434 | | | | | | | | | | | | A | | | | | | | T | | | | | | IRF7 | | | | | | | | | | | | | | . | | | | | | | | | 1.58 | | | | | | | | | | | | | | | 2.65 | | | | | | | | | | | | | 526 | | | | | | | | | | | | | 464 | | | | | | | | | | | | | 3 | | | | | | | | | | | | | | 7 | | | | | | | | | | | | 7 | | | | | | | | | | | | | | | 0.0208 | | | | | | | | | | | |
| 6 | | | | | | | | | | 11 | | | | | | | | | | | | 615087 | | | | | | | | | | | | rs761513714 | | | | | | | | | | | | G | | | | | | | T | | | | | | IRF7 | | | | | | | | | | | | | | . | | | | | | | | | 1.58 | | | | | | | | | | | | | | | 2.65 | | | | | | | | | | | | | 526 | | | | | | | | | | | | | 464 | | | | | | | | | | | | | 3 | | | | | | | | | | | | | | 7 | | | | | | | | | | | | 7 | | | | | | | | | | | | | | | . | | | | | | | | | | | |
| 6 | | | | | | | | | | 1 | | | | | | | | | | | | 16382911 | | | | | | | | | | | | rs72474563 | | | | | | | | | | | | A | | | | | | | G | | | | | | CLCNKB | | | | | | | | | | | | | | . | | | | | | | | | 5.43 | | | | | | | | | | | | | | | 1.71 | | | | | | | | | | | | | 132 | | | | | | | | | | | | | 116 | | | | | | | | | | | | | 4 | | | | | | | | | | | | | | 6 | | | | | | | | | | | | 6 | | | | | | | | | | | | | | | 0.0357 | | | | | | | | | | | |
| 6 | | | | | | | | | | 11 | | | | | | | | | | | | 7060977 | | | | | | | | | | | | rs76274604 | | | | | | | | | | | | A | | | | | | | T | | | | | | NLRP14 | | | | | | | | | | | | | | . | | | | | | | | | 1.51 | | | | | | | | | | | | | | | 2.84 | | | | | | | | | | | | | 132 | | | | | | | | | | | | | 116 | | | | | | | | | | | | | 2 | | | | | | | | | | | | | | 5 | | | | | | | | | | | | 5 | | | | | | | | | | | | | | | 0.0327 | | | | | | | | | | | |
| 6 | | | | | | | | | | 11 | | | | | | | | | | | | 74082748 | | | | | | | | | | | | rs117508615 | | | | | | | | | | | | C | | | | | | | T | | | | | | PGM2L1 | | | | | | | | | | | | | | . | | | | | | | | | 1.81 | | | | | | | | | | | | | | | 1.71 | | | | | | | | | | | | | 132 | | | | | | | | | | | | | 116 | | | | | | | | | | | | | 4 | | | | | | | | | | | | | | 6 | | | | | | | | | | | | 6 | | | | | | | | | | | | | | | 0.0387 | | | | | | | | | | | |
| group | | chr | | | | | | | | | | | pos | | | | | | | | | | | | id | | | | | | | | | | | | | ref | | | | | | | | | | | alt | | | | | | | | | | | | | | | | | | gene | | | | | | | | | | | | | LR | | | | | | | | | | | | **Gene burden ratio** | | | | | | | | | | | | | | | | | | | | | | | | | | **Total No. of alleles**  **in gene** | | | | | | | | | | | | | | | | | | | | | | | | | | | **No. alt alleles**  **in genes** | | | | | | | | | | | | | | | | | | | | | | | | **No. of**  **cases with**  **alt alleles** | | | | | | | | | | | | | **Variant allele frequency** | | | | | | |
|  | |  | | | | | | | | | | |  | | | | | | | | | | | |  | | | | | | | | | | | | |  | | | | | | | | | | |  | | | | | | | | | | | | | | | | | |  | | | | | | | | | | | | |  | | | | | | | | | | | | KG  East Asia | | | | | | | | | | | | | healthy  Ctrl | | | | | | | | | | | | | healthy  Ctrl | | | | | | | | | | | | | RA | | | | | | | | | | | | | | healthy Ctrl | | | | | | | | | | | | RA | | | | | | | | | | | | |  | | | | | | | | | | | | | KG  East Asia | | | | | |
| 6 | | | | | | | | | | 1 | | | | | | | | | | | | 177247693 | | | | | | | | | | | | rs138799872 | | | | | | | | | | | | C | | | | | | | T | | | | | | BRINP2 | | | | | | | | | | | | | | . | | | | | | | | | 3.62 | | | | | | | | | | | | | | | 2.28 | | | | | | | | | | | | | 132 | | | | | | | | | | | | | 116 | | | | | | | | | | | | | 2 | | | | | | | | | | | | | | 4 | | | | | | | | | | | | 4 | | | | | | | | | | | | | | | 0.0119 | | | | | | | | | | | |
| 6 | | | | | | | | | | 1 | | | | | | | | | | | | 182821420 | | | | | | | | | | | | rs573535598 | | | | | | | | | | | | AAGG | | | | | | | A | | | | | | DHX9 | | | | | | | | | | | | | | . | | | | | | | | | 1.81 | | | | | | | | | | | | | | | 2.28 | | | | | | | | | | | | | 132 | | | | | | | | | | | | | 116 | | | | | | | | | | | | | 1 | | | | | | | | | | | | | | 2 | | | | | | | | | | | | 2 | | | | | | | | | | | | | | | 0.0109 | | | | | | | | | | | |
| 6 | | | | | | | | | | 12 | | | | | | | | | | | | 1023218 | | | | | | | | | | | | rs4987207 | | | | | | | | | | | | G | | | | | | | T | | | | | | RAD52 | | | | | | | | | | | | | | . | | | | | | | | | 1.81 | | | | | | | | | | | | | | | 1.71 | | | | | | | | | | | | | 396 | | | | | | | | | | | | | 348 | | | | | | | | | | | | | 14 | | | | | | | | | | | | | | 21 | | | | | | | | | | | | 21 | | | | | | | | | | | | | | | 0.0268 | | | | | | | | | | | |
| 6 | | | | | | | | | | 12 | | | | | | | | | | | | 1036304 | | | | | | | | | | | | rs2286030 | | | | | | | | | | | | C | | | | | | | T | | | | | | RAD52 | | | | | | | | | | | | | | . | | | | | | | | | 1.81 | | | | | | | | | | | | | | | 1.71 | | | | | | | | | | | | | 396 | | | | | | | | | | | | | 348 | | | | | | | | | | | | | 14 | | | | | | | | | | | | | | 21 | | | | | | | | | | | | 21 | | | | | | | | | | | | | | | 0.0347 | | | | | | | | | | | |
| 6 | | | | | | | | | | 12 | | | | | | | | | | | | 1038978 | | | | | | | | | | | | rs35278212 | | | | | | | | | | | | C | | | | | | | CT | | | | | | RAD52 | | | | | | | | | | | | | | . | | | | | | | | | 1.81 | | | | | | | | | | | | | | | 1.71 | | | | | | | | | | | | | 396 | | | | | | | | | | | | | 348 | | | | | | | | | | | | | 14 | | | | | | | | | | | | | | 21 | | | | | | | | | | | | 21 | | | | | | | | | | | | | | | 0.0635 | | | | | | | | | | | |
| 6 | | | | | | | | | | 12 | | | | | | | | | | | | 109719311 | | | | | | | | | | | | rs146550988 | | | | | | | | | | | | C | | | | | | | T | | | | | | FOXN4 | | | | | | | | | | | | | | D | | | | | | | | | 1.81 | | | | | | | | | | | | | | | 2.28 | | | | | | | | | | | | | 132 | | | | | | | | | | | | | 116 | | | | | | | | | | | | | 1 | | | | | | | | | | | | | | 2 | | | | | | | | | | | | 2 | | | | | | | | | | | | | | | 0.0109 | | | | | | | | | | | |
| 6 | | | | | | | | | | 12 | | | | | | | | | | | | 11001963 | | | | | | | | | | | | . | | | | | | | | | | | | AT | | | | | | | A | | | | | | PRR4 | | | | | | | | | | | | | | . | | | | | | | | | 1.81 | | | | | | | | | | | | | | | 2.24 | | | | | | | | | | | | | 130 | | | | | | | | | | | | | 116 | | | | | | | | | | | | | 1 | | | | | | | | | | | | | | 2 | | | | | | | | | | | | 2 | | | | | | | | | | | | | | | 0.0149 | | | | | | | | | | | |
| 6 | | | | | | | | | | 12 | | | | | | | | | | | | 113592306 | | | | | | | | | | | | rs200344876 | | | | | | | | | | | | G | | | | | | | GC | | | | | | CFAP73 | | | | | | | | | | | | | | . | | | | | | | | | 2.26 | | | | | | | | | | | | | | | 2.84 | | | | | | | | | | | | | 132 | | | | | | | | | | | | | 116 | | | | | | | | | | | | | 2 | | | | | | | | | | | | | | 5 | | | | | | | | | | | | 5 | | | | | | | | | | | | | | | 0.0169 | | | | | | | | | | | |
| 6 | | | | | | | | | | 12 | | | | | | | | | | | | 16377347 | | | | | | | | | | | | rs117974895 | | | | | | | | | | | | C | | | | | | | T | | | | | | SLC15A5 | | | | | | | | | | | | | | . | | | | | | | | | 1.51 | | | | | | | | | | | | | | | 2.84 | | | | | | | | | | | | | 132 | | | | | | | | | | | | | 116 | | | | | | | | | | | | | 2 | | | | | | | | | | | | | | 5 | | | | | | | | | | | | 5 | | | | | | | | | | | | | | | 0.0337 | | | | | | | | | | | |
| 6 | | | | | | | | | | 1 | | | | | | | | | | | | 227968222 | | | | | | | | | | | | rs12568707 | | | | | | | | | | | | C | | | | | | | T | | | | | | SNAP47 | | | | | | | | | | | | | | . | | | | | | | | | 2.26 | | | | | | | | | | | | | | | 1.9 | | | | | | | | | | | | | 132 | | | | | | | | | | | | | 116 | | | | | | | | | | | | | 3 | | | | | | | | | | | | | | 5 | | | | | | | | | | | | 5 | | | | | | | | | | | | | | | 0.0188 | | | | | | | | | | | |
| 6 | | | | | | | | | | 1 | | | | | | | | | | | | 22924364 | | | | | | | | | | | | rs72651347 | | | | | | | | | | | | G | | | | | | | A | | | | | | EPHA8 | | | | | | | | | | | | | | . | | | | | | | | | 1.81 | | | | | | | | | | | | | | | 6.83 | | | | | | | | | | | | | 264 | | | | | | | | | | | | | 232 | | | | | | | | | | | | | 1 | | | | | | | | | | | | | | 6 | | | | | | | | | | | | 6 | | | | | | | | | | | | | | | 0.0179 | | | | | | | | | | | |
| 6 | | | | | | | | | | 1 | | | | | | | | | | | | 22927298 | | | | | | | | | | | | rs569320402 | | | | | | | | | | | | C | | | | | | | T | | | | | | EPHA8 | | | | | | | | | | | | | | D | | | | | | | | | 1.81 | | | | | | | | | | | | | | | 6.83 | | | | | | | | | | | | | 264 | | | | | | | | | | | | | 232 | | | | | | | | | | | | | 1 | | | | | | | | | | | | | | 6 | | | | | | | | | | | | 6 | | | | | | | | | | | | | | | . | | | | | | | | | | | |
| 6 | | | | | | | | | | 12 | | | | | | | | | | | | 404774 | | | | | | | | | | | | rs140234136 | | | | | | | | | | | | G | | | | | | | T | | | | | | KDM5A | | | | | | | | | | | | | | D | | | | | | | | | 2.53 | | | | | | | | | | | | | | | 1.69 | | | | | | | | | | | | | 420 | | | | | | | | | | | | | 356 | | | | | | | | | | | | | 7 | | | | | | | | | | | | | | 10 | | | | | | | | | | | | 10 | | | | | | | | | | | | | | | 0.0129 | | | | | | | | | | | |
| 6 | | | | | | | | | | 12 | | | | | | | | | | | | 417081 | | | | | | | | | | | | rs373321785 | | | | | | | | | | | | G | | | | | | | A | | | | | | KDM5A | | | | | | | | | | | | | | D | | | | | | | | | 2.53 | | | | | | | | | | | | | | | 1.69 | | | | | | | | | | | | | 420 | | | | | | | | | | | | | 356 | | | | | | | | | | | | | 7 | | | | | | | | | | | | | | 10 | | | | | | | | | | | | 10 | | | | | | | | | | | | | | | . | | | | | | | | | | | |
| 6 | | | | | | | | | | 12 | | | | | | | | | | | | 432376 | | | | | | | | | | | | rs751710770 | | | | | | | | | | | | CAAAA | | | | | | | C | | | | | | KDM5A | | | | | | | | | | | | | | . | | | | | | | | | 2.53 | | | | | | | | | | | | | | | 1.69 | | | | | | | | | | | | | 420 | | | | | | | | | | | | | 356 | | | | | | | | | | | | | 7 | | | | | | | | | | | | | | 10 | | | | | | | | | | | | 10 | | | | | | | | | | | | | | | . | | | | | | | | | | | |
| 6 | | | | | | | | | | 12 | | | | | | | | | | | | 432376 | | | | | | | | | | | | rs756310068 | | | | | | | | | | | | CAAA | | | | | | | C | | | | | | KDM5A | | | | | | | | | | | | | | . | | | | | | | | | 2.53 | | | | | | | | | | | | | | | 1.69 | | | | | | | | | | | | | 420 | | | | | | | | | | | | | 356 | | | | | | | | | | | | | 7 | | | | | | | | | | | | | | 10 | | | | | | | | | | | | 10 | | | | | | | | | | | | | | | . | | | | | | | | | | | |
| 6 | | | | | | | | | | 12 | | | | | | | | | | | | 498088 | | | | | | | | | | | | rs117819701 | | | | | | | | | | | | G | | | | | | | A | | | | | | KDM5A | | | | | | | | | | | | | | . | | | | | | | | | 2.53 | | | | | | | | | | | | | | | 1.69 | | | | | | | | | | | | | 420 | | | | | | | | | | | | | 356 | | | | | | | | | | | | | 7 | | | | | | | | | | | | | | 10 | | | | | | | | | | | | 10 | | | | | | | | | | | | | | | 0.0119 | | | | | | | | | | | |
| group | | chr | | | | | | | | | | | pos | | | | | | | | | | | | id | | | | | | | | | | | | | ref | | | | | | | | | | | alt | | | | | | | | | | | | | | | | | | gene | | | | | | | | | | | | | LR | | | | | | | | | | | | **Gene burden ratio** | | | | | | | | | | | | | | | | | | | | | | | | | | **Total No. of alleles**  **in gene** | | | | | | | | | | | | | | | | | | | | | | | | | | | **No. alt alleles**  **in genes** | | | | | | | | | | | | | | | | | | | | | | | | **No. of**  **cases with**  **alt alleles** | | | | | | | | | | | | | **Variant allele frequency** | | | | | | |
|  | |  | | | | | | | | | | |  | | | | | | | | | | | |  | | | | | | | | | | | | |  | | | | | | | | | | |  | | | | | | | | | | | | | | | | | |  | | | | | | | | | | | | |  | | | | | | | | | | | | KG  East Asia | | | | | | | | | | | | | healthy  Ctrl | | | | | | | | | | | | | healthy  Ctrl | | | | | | | | | | | | | RA | | | | | | | | | | | | | | healthy Ctrl | | | | | | | | | | | | RA | | | | | | | | | | | | |  | | | | | | | | | | | | | KG  East Asia | | | | | |
| 6 | | 12 | | | | | | | | | | | 51510213 | | | | | | | | | | | | rs77417603 | | | | | | | | | | | | | T | | | | | | | | | | | A | | | | | | | | | | | | | | | | | | TFCP2 | | | | | | | | | | | | | . | | | | | | | | | | | | 1.58 | | | | | | | | | | | | | 1.99 | | | | | | | | | | | | | 132 | | | | | | | | | | | | | 116 | | | | | | | | | | | | | | 4 | | | | | | | | | | | | 7 | | | | | | | | | | | | | 6 | | | | | | | | | | | | | 0.0228 | | | | | |
| 6 | | 12 | | | | | | | | | | | 63974439 | | | | | | | | | | | | rs61935050 | | | | | | | | | | | | | C | | | | | | | | | | | T | | | | | | | | | | | | | | | | | | DPY19L2 | | | | | | | | | | | | | . | | | | | | | | | | | | 2.41 | | | | | | | | | | | | | 2.24 | | | | | | | | | | | | | 130 | | | | | | | | | | | | | 116 | | | | | | | | | | | | | | 2 | | | | | | | | | | | | 4 | | | | | | | | | | | | | 4 | | | | | | | | | | | | | 0.0129 | | | | | |
| 6 | | 1 | | | | | | | | | | | 26566248 | | | | | | | | | | | | rs775335757 | | | | | | | | | | | | | T | | | | | | | | | | | C | | | | | | | | | | | | | | | | | | CEP85 | | | | | | | | | | | | | . | | | | | | | | | | | | 1.81 | | | | | | | | | | | | | 2.28 | | | | | | | | | | | | | 264 | | | | | | | | | | | | | 232 | | | | | | | | | | | | | | 2 | | | | | | | | | | | | 4 | | | | | | | | | | | | | 3 | | | | | | | | | | | | | . | | | | | |
| 6 | | 1 | | | | | | | | | | | 26601570 | | | | | | | | | | | | rs11577318 | | | | | | | | | | | | | A | | | | | | | | | | | G | | | | | | | | | | | | | | | | | | CEP85 | | | | | | | | | | | | | . | | | | | | | | | | | | 1.81 | | | | | | | | | | | | | 2.28 | | | | | | | | | | | | | 264 | | | | | | | | | | | | | 232 | | | | | | | | | | | | | | 2 | | | | | | | | | | | | 4 | | | | | | | | | | | | | 3 | | | | | | | | | | | | | 0.0228 | | | | | |
| 6 | | 1 | | | | | | | | | | | 27943525 | | | | | | | | | | | | rs2231876 | | | | | | | | | | | | | G | | | | | | | | | | | C | | | | | | | | | | | | | | | | | | FGR | | | | | | | | | | | | | . | | | | | | | | | | | | 1.63 | | | | | | | | | | | | | 1.71 | | | | | | | | | | | | | 132 | | | | | | | | | | | | | 116 | | | | | | | | | | | | | | 6 | | | | | | | | | | | | 9 | | | | | | | | | | | | | 9 | | | | | | | | | | | | | 0.0308 | | | | | |
| 6 | | 13 | | | | | | | | | | | 26155953 | | | | | | | | | | | | rs7335339 | | | | | | | | | | | | | G | | | | | | | | | | | C | | | | | | | | | | | | | | | | | | ATP8A2 | | | | | | | | | | | | | . | | | | | | | | | | | | 2.41 | | | | | | | | | | | | | 4.55 | | | | | | | | | | | | | 132 | | | | | | | | | | | | | 116 | | | | | | | | | | | | | | 1 | | | | | | | | | | | | 4 | | | | | | | | | | | | | 3 | | | | | | | | | | | | | 0.0248 | | | | | |
| 6 | | 1 | | | | | | | | | | | 39466786 | | | | | | | | | | | | rs10888613 | | | | | | | | | | | | | C | | | | | | | | | | | G | | | | | | | | | | | | | | | | | | AKIRIN1 | | | | | | | | | | | | | . | | | | | | | | | | | | 1.63 | | | | | | | | | | | | | 2.56 | | | | | | | | | | | | | 132 | | | | | | | | | | | | | 116 | | | | | | | | | | | | | | 8 | | | | | | | | | | | | 18 | | | | | | | | | | | | | 18 | | | | | | | | | | | | | 0.1052 | | | | | |
| 6 | | 14 | | | | | | | | | | | 39784010 | | | | | | | | | | | | rs539446066 | | | | | | | | | | | | | A | | | | | | | | | | | ATGTG | | | | | | | | | | | | | | | | | | CTAGE5 | | | | | | | | | | | | | . | | | | | | | | | | | | 1.64 | | | | | | | | | | | | | 3 | | | | | | | | | | | | | 128 | | | | | | | | | | | | | 128 | | | | | | | | | | | | | | 4 | | | | | | | | | | | | 12 | | | | | | | | | | | | | 12 | | | | | | | | | | | | | . | | | | | |
| 6 | | 14 | | | | | | | | | | | 39784010 | | | | | | | | | | | | rs539446066 | | | | | | | | | | | | | A | | | | | | | | | | | ATGTGTG | | | | | | | | | | | | | | | | | | CTAGE5 | | | | | | | | | | | | | . | | | | | | | | | | | | 1.64 | | | | | | | | | | | | | 3 | | | | | | | | | | | | | 128 | | | | | | | | | | | | | 128 | | | | | | | | | | | | | | 4 | | | | | | | | | | | | 12 | | | | | | | | | | | | | 12 | | | | | | | | | | | | | 0.0615 | | | | | |
| 6 | | 14 | | | | | | | | | | | 39784010 | | | | | | | | | | | | rs539446066 | | | | | | | | | | | | | A | | | | | | | | | | | ATGTGTGTG | | | | | | | | | | | | | | | | | | CTAGE5 | | | | | | | | | | | | | . | | | | | | | | | | | | 1.64 | | | | | | | | | | | | | 3 | | | | | | | | | | | | | 128 | | | | | | | | | | | | | 128 | | | | | | | | | | | | | | 4 | | | | | | | | | | | | 12 | | | | | | | | | | | | | 12 | | | | | | | | | | | | | . | | | | | |
| 6 | | 14 | | | | | | | | | | | 39784010 | | | | | | | | | | | | rs539446066 | | | | | | | | | | | | | A | | | | | | | | | | | ATGTGTGTGTG | | | | | | | | | | | | | | | | | | CTAGE5 | | | | | | | | | | | | | . | | | | | | | | | | | | 1.64 | | | | | | | | | | | | | 3 | | | | | | | | | | | | | 128 | | | | | | | | | | | | | 128 | | | | | | | | | | | | | | 4 | | | | | | | | | | | | 12 | | | | | | | | | | | | | 12 | | | | | | | | | | | | | . | | | | | |
| 6 | | 1 | | | | | | | | | | | 47403818 | | | | | | | | | | | | rs79056865 | | | | | | | | | | | | | A | | | | | | | | | | | G | | | | | | | | | | | | | | | | | | CYP4A11 | | | | | | | | | | | | | . | | | | | | | | | | | | 2.72 | | | | | | | | | | | | | 1.71 | | | | | | | | | | | | | 132 | | | | | | | | | | | | | 116 | | | | | | | | | | | | | | 8 | | | | | | | | | | | | 12 | | | | | | | | | | | | | 12 | | | | | | | | | | | | | 0.0536 | | | | | |
| 6 | | 15 | | | | | | | | | | | 48512900 | | | | | | | | | | | | rs749613571 | | | | | | | | | | | | | G | | | | | | | | | | | A | | | | | | | | | | | | | | | | | | SLC12A1 | | | | | | | | | | | | | D | | | | | | | | | | | | 10.86 | | | | | | | | | | | | | 2.28 | | | | | | | | | | | | | 396 | | | | | | | | | | | | | 348 | | | | | | | | | | | | | | 1 | | | | | | | | | | | | 2 | | | | | | | | | | | | | 2 | | | | | | | | | | | | | . | | | | | |
| 6 | | 15 | | | | | | | | | | | 48580692 | | | | | | | | | | | | rs537641866 | | | | | | | | | | | | | G | | | | | | | | | | | A | | | | | | | | | | | | | | | | | | SLC12A1 | | | | | | | | | | | | | D | | | | | | | | | | | | 10.86 | | | | | | | | | | | | | 2.28 | | | | | | | | | | | | | 396 | | | | | | | | | | | | | 348 | | | | | | | | | | | | | | 1 | | | | | | | | | | | | 2 | | | | | | | | | | | | | 2 | | | | | | | | | | | | | . | | | | | |
| 6 | | 15 | | | | | | | | | | | 48594989 | | | | | | | | | | | | rs755737521 | | | | | | | | | | | | | G | | | | | | | | | | | T | | | | | | | | | | | | | | | | | | SLC12A1 | | | | | | | | | | | | | D | | | | | | | | | | | | 10.86 | | | | | | | | | | | | | 2.28 | | | | | | | | | | | | | 396 | | | | | | | | | | | | | 348 | | | | | | | | | | | | | | 1 | | | | | | | | | | | | 2 | | | | | | | | | | | | | 2 | | | | | | | | | | | | | . | | | | | |
| 6 | | 1 | | | | | | | | | | | 55076238 | | | | | | | | | | | | rs7535372 | | | | | | | | | | | | | C | | | | | | | | | | | A | | | | | | | | | | | | | | | | | | FAM151A | | | | | | | | | | | | | . | | | | | | | | | | | | 1.62 | | | | | | | | | | | | | 1.53 | | | | | | | | | | | | | 130 | | | | | | | | | | | | | 116 | | | | | | | | | | | | | | 19 | | | | | | | | | | | | 26 | | | | | | | | | | | | | 22 | | | | | | | | | | | | | 0.1687 | | | | | |
| 6 | | 15 | | | | | | | | | | | 55484910 | | | | | | | | | | | | rs3759863 | | | | | | | | | | | | | G | | | | | | | | | | | A | | | | | | | | | | | | | | | | | | RSL24D1 | | | | | | | | | | | | | . | | | | | | | | | | | | 1.81 | | | | | | | | | | | | | 1.55 | | | | | | | | | | | | | 132 | | | | | | | | | | | | | 116 | | | | | | | | | | | | | | 11 | | | | | | | | | | | | 15 | | | | | | | | | | | | | 14 | | | | | | | | | | | | | 0.0685 | | | | | |
| 6 | | 15 | | | | | | | | | | | 75628507 | | | | | | | | | | | | . | | | | | | | | | | | | | C | | | | | | | | | | | T | | | | | | | | | | | | | | | | | | COMMD4 | | | | | | | | | | | | | . | | | | | | | | | | | | 1.81 | | | | | | | | | | | | | 2.28 | | | | | | | | | | | | | 264 | | | | | | | | | | | | | 232 | | | | | | | | | | | | | | 1 | | | | | | | | | | | | 2 | | | | | | | | | | | | | 2 | | | | | | | | | | | | | . | | | | | |
| group | chr | | | | | | | | | | | | | | | pos | | | | | | | | | | | id | | | | | | | | | | ref | | | | | | | | | | | | | | | | | | | | | | | | | | | | | alt | | | | | | | | | | | gene | | | | | | | | | | | | | | | | LR | | | **Gene burden ratio** | | | | | | | | | | | | | | | | | | | | | | | | | **Total No. of alleles**  **in gene** | | | | | | | | | | | | | | | | | | | | | | | | | | | **No. alt alleles**  **in genes** | | | | | | | | | | | | | | | | | | | | | | | | | **No. of**  **cases with**  **alt alleles** | | | | | | | | | | | | | | **Variant allele frequency** | |
|  |  | | | | | | | | | | | | | | |  | | | | | | | | | | |  | | | | | | | | | |  | | | | | | | | | | | | | | | | | | | | | | | | | | | | |  | | | | | | | | | | |  | | | | | | | | | | | | | | | |  | | | KG  East Asia | | | | | | | | | | | healthy  Ctrl | | | | | | | | | | | | | | healthy  Ctrl | | | | | | | | | | | | RA | | | | | | | | | | | | | | | healthy Ctrl | | | | | | | | | | | RA | | | | | | | | | | | | | | |  | | | | | | | | | | | | | | KG  East Asia |
| 6 | | | 15 | | | | | | | | | | | 75631598 | | | | | | | | | | | | rs200129803 | | | | | | | | | | | | | C | | | | | | | | | | | T | | | | | | | | | | | | | | | | | | COMMD4 | | | | | | | | | | | | | . | | | | | | | | | | | 1.81 | | | | | | | | | | | | 2.28 | | | | | | | | | | | | | 264 | | | | | | | | | | | | | 232 | | | | | | | | | | | | | | 1 | | | | | | | | | | | 2 | | | | | | | | | | | | | 2 | | | | | | | | | | | | | | 0.0149 | | | | | | |
| 6 | | | 16 | | | | | | | | | | | 1825689 | | | | | | | | | | | | rs3826055 | | | | | | | | | | | | | C | | | | | | | | | | | T | | | | | | | | | | | | | | | | | | EME2 | | | | | | | | | | | | | . | | | | | | | | | | | 4.53 | | | | | | | | | | | | 5.69 | | | | | | | | | | | | | 264 | | | | | | | | | | | | | 232 | | | | | | | | | | | | | | 1 | | | | | | | | | | | 5 | | | | | | | | | | | | | 5 | | | | | | | | | | | | | | 0.0129 | | | | | | |
| 6 | | | 16 | | | | | | | | | | | 1825789 | | | | | | | | | | | | rs746707908 | | | | | | | | | | | | | T | | | | | | | | | | | C | | | | | | | | | | | | | | | | | | EME2 | | | | | | | | | | | | | . | | | | | | | | | | | 4.53 | | | | | | | | | | | | 5.69 | | | | | | | | | | | | | 264 | | | | | | | | | | | | | 232 | | | | | | | | | | | | | | 1 | | | | | | | | | | | 5 | | | | | | | | | | | | | 5 | | | | | | | | | | | | | | . | | | | | | |
| 6 | | | 16 | | | | | | | | | | | 2017804 | | | | | | | | | | | | rs146108433 | | | | | | | | | | | | | T | | | | | | | | | | | G | | | | | | | | | | | | | | | | | | RNF151 | | | | | | | | | | | | | D | | | | | | | | | | | 3.62 | | | | | | | | | | | | 2.28 | | | | | | | | | | | | | 132 | | | | | | | | | | | | | 116 | | | | | | | | | | | | | | 4 | | | | | | | | | | | 8 | | | | | | | | | | | | | 7 | | | | | | | | | | | | | | 0.0387 | | | | | | |
| 6 | | | 16 | | | | | | | | | | | 30455945 | | | | | | | | | | | | rs146596728 | | | | | | | | | | | | | A | | | | | | | | | | | C | | | | | | | | | | | | | | | | | | SEPHS2 | | | | | | | | | | | | | . | | | | | | | | | | | 2.72 | | | | | | | | | | | | 1.71 | | | | | | | | | | | | | 264 | | | | | | | | | | | | | 232 | | | | | | | | | | | | | | 2 | | | | | | | | | | | 3 | | | | | | | | | | | | | 3 | | | | | | | | | | | | | | 0.0248 | | | | | | |
| 6 | | | 16 | | | | | | | | | | | 30456188 | | | | | | | | | | | | rs550048089 | | | | | | | | | | | | | G | | | | | | | | | | | A | | | | | | | | | | | | | | | | | | SEPHS2 | | | | | | | | | | | | | . | | | | | | | | | | | 2.72 | | | | | | | | | | | | 1.71 | | | | | | | | | | | | | 264 | | | | | | | | | | | | | 232 | | | | | | | | | | | | | | 2 | | | | | | | | | | | 3 | | | | | | | | | | | | | 3 | | | | | | | | | | | | | | . | | | | | | |
| 6 | | | 16 | | | | | | | | | | | 31004812 | | | | | | | | | | | | rs12445568 | | | | | | | | | | | | | T | | | | | | | | | | | C | | | | | | | | | | | | | | | | | | STX1B | | | | | | | | | | | | | . | | | | | | | | | | | 1.91 | | | | | | | | | | | | 1.8 | | | | | | | | | | | | | 132 | | | | | | | | | | | | | 116 | | | | | | | | | | | | | | 12 | | | | | | | | | | | 19 | | | | | | | | | | | | | 17 | | | | | | | | | | | | | | 0.1002 | | | | | | |
| 6 | | | 16 | | | | | | | | | | | 67198846 | | | | | | | | | | | | rs115335849 | | | | | | | | | | | | | C | | | | | | | | | | | T | | | | | | | | | | | | | | | | | | HSF4 | | | | | | | | | | | | | . | | | | | | | | | | | 2.41 | | | | | | | | | | | | 1.52 | | | | | | | | | | | | | 132 | | | | | | | | | | | | | 116 | | | | | | | | | | | | | | 3 | | | | | | | | | | | 4 | | | | | | | | | | | | | 4 | | | | | | | | | | | | | | 0.0278 | | | | | | |
| 6 | | | 16 | | | | | | | | | | | 733320 | | | | | | | | | | | | . | | | | | | | | | | | | | A | | | | | | | | | | | T | | | | | | | | | | | | | | | | | | JMJD8 | | | | | | | | | | | | | . | | | | | | | | | | | 2.72 | | | | | | | | | | | | 1.71 | | | | | | | | | | | | | 264 | | | | | | | | | | | | | 232 | | | | | | | | | | | | | | 2 | | | | | | | | | | | 3 | | | | | | | | | | | | | 3 | | | | | | | | | | | | | | . | | | | | | |
| 6 | | | 16 | | | | | | | | | | | 733604 | | | | | | | | | | | | rs79868981 | | | | | | | | | | | | | G | | | | | | | | | | | A | | | | | | | | | | | | | | | | | | JMJD8 | | | | | | | | | | | | | . | | | | | | | | | | | 2.72 | | | | | | | | | | | | 1.71 | | | | | | | | | | | | | 264 | | | | | | | | | | | | | 232 | | | | | | | | | | | | | | 2 | | | | | | | | | | | 3 | | | | | | | | | | | | | 3 | | | | | | | | | | | | | | 0.0129 | | | | | | |
| 6 | | | 1 | | | | | | | | | | | 67442275 | | | | | | | | | | | | rs11208979 | | | | | | | | | | | | | C | | | | | | | | | | | T | | | | | | | | | | | | | | | | | | MIER1 | | | | | | | | | | | | | . | | | | | | | | | | | 1.66 | | | | | | | | | | | | 1.93 | | | | | | | | | | | | | 132 | | | | | | | | | | | | | 116 | | | | | | | | | | | | | | 13 | | | | | | | | | | | 22 | | | | | | | | | | | | | 20 | | | | | | | | | | | | | | 0.1171 | | | | | | |
| 6 | | | 17 | | | | | | | | | | | 16342833 | | | | | | | | | | | | rs11871958 | | | | | | | | | | | | | T | | | | | | | | | | | C | | | | | | | | | | | | | | | | | | LRRC75A-AS1 | | | | | | | | | | | | | . | | | | | | | | | | | 1.81 | | | | | | | | | | | | 2.11 | | | | | | | | | | | | | 132 | | | | | | | | | | | | | 116 | | | | | | | | | | | | | | 7 | | | | | | | | | | | 13 | | | | | | | | | | | | | 13 | | | | | | | | | | | | | | 0.0823 | | | | | | |
| 6 | | | 17 | | | | | | | | | | | 18022218 | | | | | | | | | | | | rs765495851 | | | | | | | | | | | | | G | | | | | | | | | | | A | | | | | | | | | | | | | | | | | | MYO15A | | | | | | | | | | | | | D | | | | | | | | | | | 2.04 | | | | | | | | | | | | 1.71 | | | | | | | | | | | | | 1056 | | | | | | | | | | | | | 928 | | | | | | | | | | | | | | 4 | | | | | | | | | | | 6 | | | | | | | | | | | | | 6 | | | | | | | | | | | | | | . | | | | | | |
| 6 | | | 17 | | | | | | | | | | | 18023739 | | | | | | | | | | | | rs766303371 | | | | | | | | | | | | | T | | | | | | | | | | | G | | | | | | | | | | | | | | | | | | MYO15A | | | | | | | | | | | | | D | | | | | | | | | | | 2.04 | | | | | | | | | | | | 1.71 | | | | | | | | | | | | | 1056 | | | | | | | | | | | | | 928 | | | | | | | | | | | | | | 4 | | | | | | | | | | | 6 | | | | | | | | | | | | | 6 | | | | | | | | | | | | | | . | | | | | | |
| 6 | | | 17 | | | | | | | | | | | 18028490 | | | | | | | | | | | | . | | | | | | | | | | | | | A | | | | | | | | | | | G | | | | | | | | | | | | | | | | | | MYO15A | | | | | | | | | | | | | D | | | | | | | | | | | 2.04 | | | | | | | | | | | | 1.71 | | | | | | | | | | | | | 1056 | | | | | | | | | | | | | 928 | | | | | | | | | | | | | | 4 | | | | | | | | | | | 6 | | | | | | | | | | | | | 6 | | | | | | | | | | | | | | . | | | | | | |
| 6 | | | 17 | | | | | | | | | | | 18043856 | | | | | | | | | | | | . | | | | | | | | | | | | | A | | | | | | | | | | | G | | | | | | | | | | | | | | | | | | MYO15A | | | | | | | | | | | | | D | | | | | | | | | | | 2.04 | | | | | | | | | | | | 1.71 | | | | | | | | | | | | | 1056 | | | | | | | | | | | | | 928 | | | | | | | | | | | | | | 4 | | | | | | | | | | | 6 | | | | | | | | | | | | | 6 | | | | | | | | | | | | | | . | | | | | | |
| 6 | | | 17 | | | | | | | | | | | 18051423 | | | | | | | | | | | | . | | | | | | | | | | | | | A | | | | | | | | | | | G | | | | | | | | | | | | | | | | | | MYO15A | | | | | | | | | | | | | D | | | | | | | | | | | 2.04 | | | | | | | | | | | | 1.71 | | | | | | | | | | | | | 1056 | | | | | | | | | | | | | 928 | | | | | | | | | | | | | | 4 | | | | | | | | | | | 6 | | | | | | | | | | | | | 6 | | | | | | | | | | | | | | . | | | | | | |
| group | chr | | | | | | | | | | | | | | | pos | | | | | | | | | | | id | | | | | | | | | | ref | | | | | | | | | | | | | | | | | | | | | | | | | | | | | alt | | | | | | | | | | | gene | | | | | | | | | | | | | | | | LR | | | **Gene burden ratio** | | | | | | | | | | | | | | | | | | | | | | | | | **Total No. of alleles**  **in gene** | | | | | | | | | | | | | | | | | | | | | | | | | | | **No. alt alleles**  **in genes** | | | | | | | | | | | | | | | | | | | | | | | | | **No. of**  **cases with**  **alt alleles** | | | | | | | | | | | | | | **Variant allele frequency** | |
|  |  | | | | | | | | | | | | | | |  | | | | | | | | | | |  | | | | | | | | | |  | | | | | | | | | | | | | | | | | | | | | | | | | | | | |  | | | | | | | | | | |  | | | | | | | | | | | | | | | |  | | | KG  East Asia | | | | | | | | | | | healthy  Ctrl | | | | | | | | | | | | | | healthy  Ctrl | | | | | | | | | | | | RA | | | | | | | | | | | | | | | healthy Ctrl | | | | | | | | | | | RA | | | | | | | | | | | | | | |  | | | | | | | | | | | | | | KG  East Asia |
| 6 | 17 | | | | | | | | | | | | | | | 18057215 | | | | | | | | | | | rs9916193 | | | | | | | | | | C | | | | | | | | | | | | | | | | | | | | | | | | | | | | | G | | | | | | | | | | | MYO15A | | | | | | | | | | | | | | | | . | | | 2.04 | | | | | | | | | | | 1.71 | | | | | | | | | | | | | | 1056 | | | | | | | | | | | | 928 | | | | | | | | | | | | | | | 4 | | | | | | | | | | | 6 | | | | | | | | | | | | | | | 6 | | | | | | | | | | | | | | 0.0119 |
| 6 | 17 | | | | | | | | | | | | | | | 18064722 | | | | | | | | | | | rs140029076 | | | | | | | | | | C | | | | | | | | | | | | | | | | | | | | | | | | | | | | | T | | | | | | | | | | | MYO15A | | | | | | | | | | | | | | | | D | | | 2.04 | | | | | | | | | | | 1.71 | | | | | | | | | | | | | | 1056 | | | | | | | | | | | | 928 | | | | | | | | | | | | | | | 4 | | | | | | | | | | | 6 | | | | | | | | | | | | | | | 6 | | | | | | | | | | | | | | . |
| 6 | 17 | | | | | | | | | | | | | | | 18071031 | | | | | | | | | | | rs201763265 | | | | | | | | | | C | | | | | | | | | | | | | | | | | | | | | | | | | | | | | T | | | | | | | | | | | MYO15A | | | | | | | | | | | | | | | | D | | | 2.04 | | | | | | | | | | | 1.71 | | | | | | | | | | | | | | 1056 | | | | | | | | | | | | 928 | | | | | | | | | | | | | | | 4 | | | | | | | | | | | 6 | | | | | | | | | | | | | | | 6 | | | | | | | | | | | | | | . |
| 6 | 17 | | | | | | | | | | | | | | | 38511509 | | | | | | | | | | | rs188616110 | | | | | | | | | | C | | | | | | | | | | | | | | | | | | | | | | | | | | | | | T | | | | | | | | | | | RARA | | | | | | | | | | | | | | | | . | | | 4.53 | | | | | | | | | | | 2.84 | | | | | | | | | | | | | | 132 | | | | | | | | | | | | 116 | | | | | | | | | | | | | | | 2 | | | | | | | | | | | 5 | | | | | | | | | | | | | | | 5 | | | | | | | | | | | | | | 0.0149 |
| 6 | 17 | | | | | | | | | | | | | | | 47486684 | | | | | | | | | | | rs2277637 | | | | | | | | | | T | | | | | | | | | | | | | | | | | | | | | | | | | | | | | C | | | | | | | | | | | PHB | | | | | | | | | | | | | | | | . | | | 1.65 | | | | | | | | | | | 1.71 | | | | | | | | | | | | | | 132 | | | | | | | | | | | | 116 | | | | | | | | | | | | | | | 14 | | | | | | | | | | | 21 | | | | | | | | | | | | | | | 17 | | | | | | | | | | | | | | 0.123 |
| 6 | 17 | | | | | | | | | | | | | | | 67079441 | | | | | | | | | | | rs143326198 | | | | | | | | | | C | | | | | | | | | | | | | | | | | | | | | | | | | | | | | G | | | | | | | | | | | ABCA6 | | | | | | | | | | | | | | | | D | | | 1.63 | | | | | | | | | | | 2.26 | | | | | | | | | | | | | | 656 | | | | | | | | | | | | 580 | | | | | | | | | | | | | | | 2 | | | | | | | | | | | 4 | | | | | | | | | | | | | | | 4 | | | | | | | | | | | | | | 0.0119 |
| 6 | 17 | | | | | | | | | | | | | | | 67081193 | | | | | | | | | | | rs527461596 | | | | | | | | | | G | | | | | | | | | | | | | | | | | | | | | | | | | | | | | C | | | | | | | | | | | ABCA6 | | | | | | | | | | | | | | | | D | | | 1.63 | | | | | | | | | | | 2.26 | | | | | | | | | | | | | | 656 | | | | | | | | | | | | 580 | | | | | | | | | | | | | | | 2 | | | | | | | | | | | 4 | | | | | | | | | | | | | | | 4 | | | | | | | | | | | | | | . |
| 6 | 17 | | | | | | | | | | | | | | | 67109811 | | | | | | | | | | | rs777203184 | | | | | | | | | | T | | | | | | | | | | | | | | | | | | | | | | | | | | | | | G | | | | | | | | | | | ABCA6 | | | | | | | | | | | | | | | | D | | | 1.63 | | | | | | | | | | | 2.26 | | | | | | | | | | | | | | 656 | | | | | | | | | | | | 580 | | | | | | | | | | | | | | | 2 | | | | | | | | | | | 4 | | | | | | | | | | | | | | | 4 | | | | | | | | | | | | | | . |
| 6 | 17 | | | | | | | | | | | | | | | 67121068 | | | | | | | | | | | . | | | | | | | | | | A | | | | | | | | | | | | | | | | | | | | | | | | | | | | | G | | | | | | | | | | | ABCA6 | | | | | | | | | | | | | | | | D | | | 1.63 | | | | | | | | | | | 2.26 | | | | | | | | | | | | | | 656 | | | | | | | | | | | | 580 | | | | | | | | | | | | | | | 2 | | | | | | | | | | | 4 | | | | | | | | | | | | | | | 4 | | | | | | | | | | | | | | . |
| 6 | 17 | | | | | | | | | | | | | | | 67124939 | | | | | | | | | | | . | | | | | | | | | | A | | | | | | | | | | | | | | | | | | | | | | | | | | | | | C | | | | | | | | | | | ABCA6 | | | | | | | | | | | | | | | | D | | | 1.63 | | | | | | | | | | | 2.26 | | | | | | | | | | | | | | 656 | | | | | | | | | | | | 580 | | | | | | | | | | | | | | | 2 | | | | | | | | | | | 4 | | | | | | | | | | | | | | | 4 | | | | | | | | | | | | | | . |
| 6 | 1 | | | | | | | | | | | | | | | 78420930 | | | | | | | | | | | rs151051327 | | | | | | | | | | T | | | | | | | | | | | | | | | | | | | | | | | | | | | | | C | | | | | | | | | | | FUBP1 | | | | | | | | | | | | | | | | . | | | 1.52 | | | | | | | | | | | 1.76 | | | | | | | | | | | | | | 396 | | | | | | | | | | | | 346 | | | | | | | | | | | | | | | 13 | | | | | | | | | | | 20 | | | | | | | | | | | | | | | 19 | | | | | | | | | | | | | | 0.0109 |
| 6 | 1 | | | | | | | | | | | | | | | 78429408 | | | | | | | | | | | rs2274257 | | | | | | | | | | G | | | | | | | | | | | | | | | | | | | | | | | | | | | | | C | | | | | | | | | | | FUBP1 | | | | | | | | | | | | | | | | . | | | 1.52 | | | | | | | | | | | 1.76 | | | | | | | | | | | | | | 396 | | | | | | | | | | | | 346 | | | | | | | | | | | | | | | 13 | | | | | | | | | | | 20 | | | | | | | | | | | | | | | 19 | | | | | | | | | | | | | | 0.0903 |
| 6 | 1 | | | | | | | | | | | | | | | 78432563 | | | | | | | | | | | . | | | | | | | | | | A | | | | | | | | | | | | | | | | | | | | | | | | | | | | | G | | | | | | | | | | | FUBP1 | | | | | | | | | | | | | | | | . | | | 1.52 | | | | | | | | | | | 1.76 | | | | | | | | | | | | | | 396 | | | | | | | | | | | | 346 | | | | | | | | | | | | | | | 13 | | | | | | | | | | | 20 | | | | | | | | | | | | | | | 19 | | | | | | | | | | | | | | . |
| 6 | 18 | | | | | | | | | | | | | | | 50432706 | | | | | | | | | | | rs17389547 | | | | | | | | | | A | | | | | | | | | | | | | | | | | | | | | | | | | | | | | C | | | | | | | | | | | DCC | | | | | | | | | | | | | | | | . | | | 1.81 | | | | | | | | | | | 1.51 | | | | | | | | | | | | | | 396 | | | | | | | | | | | | 348 | | | | | | | | | | | | | | | 40 | | | | | | | | | | | 53 | | | | | | | | | | | | | | | 43 | | | | | | | | | | | | | | 0.0109 |
| 6 | 18 | | | | | | | | | | | | | | | 50912515 | | | | | | | | | | | rs3764494 | | | | | | | | | | G | | | | | | | | | | | | | | | | | | | | | | | | | | | | | A | | | | | | | | | | | DCC | | | | | | | | | | | | | | | | . | | | 1.81 | | | | | | | | | | | 1.51 | | | | | | | | | | | | | | 396 | | | | | | | | | | | | 348 | | | | | | | | | | | | | | | 40 | | | | | | | | | | | 53 | | | | | | | | | | | | | | | 43 | | | | | | | | | | | | | | 0.0238 |
| 6 | 18 | | | | | | | | | | | | | | | 50937026 | | | | | | | | | | | rs11873515 | | | | | | | | | | A | | | | | | | | | | | | | | | | | | | | | | | | | | | | | G | | | | | | | | | | | DCC | | | | | | | | | | | | | | | | . | | | 1.81 | | | | | | | | | | | 1.51 | | | | | | | | | | | | | | 396 | | | | | | | | | | | | 348 | | | | | | | | | | | | | | | 40 | | | | | | | | | | | 53 | | | | | | | | | | | | | | | 43 | | | | | | | | | | | | | | 0.2252 |
| 6 | 19 | | | | | | | | | | | | | | | 13318672 | | | | | | | | | | | . | | | | | | | | | | CCTGCTGCTGCTGCT  GCTGCTGCTGCTG | | | | | | | | | | | | | | | | | | | | | | | | | | | | | C | | | | | | | | | | | CACNA1A | | | | | | | | | | | | | | | | . | | | 2.45 | | | | | | | | | | | 2.06 | | | | | | | | | | | | | | 708 | | | | | | | | | | | | 668 | | | | | | | | | | | | | | | 36 | | | | | | | | | | | 70 | | | | | | | | | | | | | | | 70 | | | | | | | | | | | | | | . |
| group | | | chr | | | | | | | | | | | pos | | | | | | | | | | | | id | | | | | | | | | | | | | ref | | | | | | | | | | | | | | | | | alt | | | | | | | | | | | | | gene | | | | | | | | | | | | | | LR | | | | | | | **Gene burden ratio** | | | | | | | | | | | | | | | | | | | | | | | | | **Total No. of alleles**  **in gene** | | | | | | | | | | | | | | | | | | | | | | | | | | | **No. alt alleles**  **in genes** | | | | | | | | | | | | | | | | | | | | | | | | | | **No. of**  **cases with**  **alt alleles** | | | | | | | | | | | | | | **Variant allele frequency** | | | | | | |
|  | | |  | | | | | | | | | | |  | | | | | | | | | | | |  | | | | | | | | | | | | |  | | | | | | | | | | | | | | | | |  | | | | | | | | | | | | |  | | | | | | | | | | | | | |  | | | | | | | KG  East Asia | | | | | | | | | | | | healthy  Ctrl | | | | | | | | | | | | | healthy  Ctrl | | | | | | | | | | | | | RA | | | | | | | | | | | | | | healthy Ctrl | | | | | | | | | | | | | RA | | | | | | | | | | | | |  | | | | | | | | | | | | | | KG  East Asia | | | | | | |
| 6 | | | 19 | | | | | | | | | | | 13318672 | | | | | | | | | | | | rs16054 | | | | | | | | | | | | | CCTGCTG | | | | | | | | | | | | | | | | | C | | | | | | | | | | | | | CACNA1A | | | | | | | | | | | | | | . | | | | | | | 2.45 | | | | | | | | | | | | 2.06 | | | | | | | | | | | | | 708 | | | | | | | | | | | | | 668 | | | | | | | | | | | | | | 36 | | | | | | | | | | | | | 70 | | | | | | | | | | | | | 70 | | | | | | | | | | | | | | . | | | | | | |
| 6 | | | 19 | | | | | | | | | | | 13318672 | | | | | | | | | | | | rs370146696 | | | | | | | | | | | | | CCTG | | | | | | | | | | | | | | | | | C | | | | | | | | | | | | | CACNA1A | | | | | | | | | | | | | | . | | | | | | | 2.45 | | | | | | | | | | | | 2.06 | | | | | | | | | | | | | 708 | | | | | | | | | | | | | 668 | | | | | | | | | | | | | | 36 | | | | | | | | | | | | | 70 | | | | | | | | | | | | | 70 | | | | | | | | | | | | | | 0.0714 | | | | | | |
| 6 | | | 19 | | | | | | | | | | | 13318672 | | | | | | | | | | | | rs753460234 | | | | | | | | | | | | | C | | | | | | | | | | | | | | | | | CCTG | | | | | | | | | | | | | CACNA1A | | | | | | | | | | | | | | . | | | | | | | 2.45 | | | | | | | | | | | | 2.06 | | | | | | | | | | | | | 708 | | | | | | | | | | | | | 668 | | | | | | | | | | | | | | 36 | | | | | | | | | | | | | 70 | | | | | | | | | | | | | 70 | | | | | | | | | | | | | | . | | | | | | |
| 6 | | | 19 | | | | | | | | | | | 13318672 | | | | | | | | | | | | rs753460234 | | | | | | | | | | | | | C | | | | | | | | | | | | | | | | | CCTGCTG | | | | | | | | | | | | | CACNA1A | | | | | | | | | | | | | | . | | | | | | | 2.45 | | | | | | | | | | | | 2.06 | | | | | | | | | | | | | 708 | | | | | | | | | | | | | 668 | | | | | | | | | | | | | | 36 | | | | | | | | | | | | | 70 | | | | | | | | | | | | | 70 | | | | | | | | | | | | | | . | | | | | | |
| 6 | | | 19 | | | | | | | | | | | 13318672 | | | | | | | | | | | | rs765169827 | | | | | | | | | | | | | CCTGCTGCTG  CTGCTGCTG | | | | | | | | | | | | | | | | | C | | | | | | | | | | | | | CACNA1A | | | | | | | | | | | | | | . | | | | | | | 2.45 | | | | | | | | | | | | 2.06 | | | | | | | | | | | | | 708 | | | | | | | | | | | | | 668 | | | | | | | | | | | | | | 36 | | | | | | | | | | | | | 70 | | | | | | | | | | | | | 70 | | | | | | | | | | | | | | . | | | | | | |
| 6 | | | 19 | | | | | | | | | | | 13394158 | | | | | | | | | | | | . | | | | | | | | | | | | | T | | | | | | | | | | | | | | | | | C | | | | | | | | | | | | | CACNA1A | | | | | | | | | | | | | | D | | | | | | | 2.45 | | | | | | | | | | | | 2.06 | | | | | | | | | | | | | 708 | | | | | | | | | | | | | 668 | | | | | | | | | | | | | | 36 | | | | | | | | | | | | | 70 | | | | | | | | | | | | | 70 | | | | | | | | | | | | | | . | | | | | | |
| 6 | | | 19 | | | | | | | | | | | 13410044 | | | | | | | | | | | | . | | | | | | | | | | | | | C | | | | | | | | | | | | | | | | | G | | | | | | | | | | | | | CACNA1A | | | | | | | | | | | | | | D | | | | | | | 2.45 | | | | | | | | | | | | 2.06 | | | | | | | | | | | | | 708 | | | | | | | | | | | | | 668 | | | | | | | | | | | | | | 36 | | | | | | | | | | | | | 70 | | | | | | | | | | | | | 70 | | | | | | | | | | | | | | . | | | | | | |
| 6 | | | 19 | | | | | | | | | | | 13616741 | | | | | | | | | | | | . | | | | | | | | | | | | | G | | | | | | | | | | | | | | | | | A | | | | | | | | | | | | | CACNA1A | | | | | | | | | | | | | | . | | | | | | | 2.45 | | | | | | | | | | | | 2.06 | | | | | | | | | | | | | 708 | | | | | | | | | | | | | 668 | | | | | | | | | | | | | | 36 | | | | | | | | | | | | | 70 | | | | | | | | | | | | | 70 | | | | | | | | | | | | | | . | | | | | | |
| 6 | | | 19 | | | | | | | | | | | 13616977 | | | | | | | | | | | | rs15999 | | | | | | | | | | | | | G | | | | | | | | | | | | | | | | | A | | | | | | | | | | | | | CACNA1A | | | | | | | | | | | | | | D | | | | | | | 2.45 | | | | | | | | | | | | 2.06 | | | | | | | | | | | | | 708 | | | | | | | | | | | | | 668 | | | | | | | | | | | | | | 36 | | | | | | | | | | | | | 70 | | | | | | | | | | | | | 70 | | | | | | | | | | | | | | 0.0159 | | | | | | |
| 6 | | | 19 | | | | | | | | | | | 1367226 | | | | | | | | | | | | rs11668809 | | | | | | | | | | | | | G | | | | | | | | | | | | | | | | | A | | | | | | | | | | | | | MUM1 | | | | | | | | | | | | | | . | | | | | | | 1.81 | | | | | | | | | | | | 2.28 | | | | | | | | | | | | | 132 | | | | | | | | | | | | | 116 | | | | | | | | | | | | | | 1 | | | | | | | | | | | | | 2 | | | | | | | | | | | | | 2 | | | | | | | | | | | | | | 0.0149 | | | | | | |
| 6 | | | 19 | | | | | | | | | | | 32083223 | | | | | | | | | | | | rs11880125 | | | | | | | | | | | | | A | | | | | | | | | | | | | | | | | G | | | | | | | | | | | | | THEG5 | | | | | | | | | | | | | | . | | | | | | | 3.02 | | | | | | | | | | | | 2.84 | | | | | | | | | | | | | 264 | | | | | | | | | | | | | 232 | | | | | | | | | | | | | | 4 | | | | | | | | | | | | | 10 | | | | | | | | | | | | | 10 | | | | | | | | | | | | | | 0.0179 | | | | | | |
| 6 | | | 19 | | | | | | | | | | | 32083250 | | | | | | | | | | | | rs79323410 | | | | | | | | | | | | | T | | | | | | | | | | | | | | | | | C | | | | | | | | | | | | | THEG5 | | | | | | | | | | | | | | . | | | | | | | 3.02 | | | | | | | | | | | | 2.84 | | | | | | | | | | | | | 264 | | | | | | | | | | | | | 232 | | | | | | | | | | | | | | 4 | | | | | | | | | | | | | 10 | | | | | | | | | | | | | 10 | | | | | | | | | | | | | | 0.0179 | | | | | | |
| 6 | | | 19 | | | | | | | | | | | 36394245 | | | | | | | | | | | | rs74258162 | | | | | | | | | | | | | T | | | | | | | | | | | | | | | | | C | | | | | | | | | | | | | HCST | | | | | | | | | | | | | | . | | | | | | | 1.81 | | | | | | | | | | | | 3.41 | | | | | | | | | | | | | 132 | | | | | | | | | | | | | 116 | | | | | | | | | | | | | | 2 | | | | | | | | | | | | | 6 | | | | | | | | | | | | | 6 | | | | | | | | | | | | | | 0.0357 | | | | | | |
| 6 | | | 19 | | | | | | | | | | | 38572367 | | | | | | | | | | | | rs562186095 | | | | | | | | | | | | | GGCCACC | | | | | | | | | | | | | | | | | G | | | | | | | | | | | | | SIPA1L3 | | | | | | | | | | | | | | . | | | | | | | 4.66 | | | | | | | | | | | | 2.17 | | | | | | | | | | | | | 392 | | | | | | | | | | | | | 348 | | | | | | | | | | | | | | 14 | | | | | | | | | | | | | 27 | | | | | | | | | | | | | 27 | | | | | | | | | | | | | | . | | | | | | |
| 6 | | | 19 | | | | | | | | | | | 38572367 | | | | | | | | | | | | rs569252662 | | | | | | | | | | | | | G | | | | | | | | | | | | | | | | | GGCCACC | | | | | | | | | | | | | SIPA1L3 | | | | | | | | | | | | | | . | | | | | | | 4.66 | | | | | | | | | | | | 2.17 | | | | | | | | | | | | | 392 | | | | | | | | | | | | | 348 | | | | | | | | | | | | | | 14 | | | | | | | | | | | | | 27 | | | | | | | | | | | | | 27 | | | | | | | | | | | | | | 0.0337 | | | | | | |
| 6 | | | 19 | | | | | | | | | | | 38590722 | | | | | | | | | | | | . | | | | | | | | | | | | | C | | | | | | | | | | | | | | | | | T | | | | | | | | | | | | | SIPA1L3 | | | | | | | | | | | | | | D | | | | | | | 4.66 | | | | | | | | | | | | 2.17 | | | | | | | | | | | | | 392 | | | | | | | | | | | | | 348 | | | | | | | | | | | | | | 14 | | | | | | | | | | | | | 27 | | | | | | | | | | | | | 27 | | | | | | | | | | | | | | . | | | | | | |
| 6 | | | 19 | | | | | | | | | | | 39421820 | | | | | | | | | | | | rs2304116 | | | | | | | | | | | | | T | | | | | | | | | | | | | | | | | G | | | | | | | | | | | | | MRPS12 | | | | | | | | | | | | | | . | | | | | | | 3.62 | | | | | | | | | | | | 2.28 | | | | | | | | | | | | | 132 | | | | | | | | | | | | | 116 | | | | | | | | | | | | | | 1 | | | | | | | | | | | | | 2 | | | | | | | | | | | | | 2 | | | | | | | | | | | | | | 0.0119 | | | | | | |
| group | | | | | | | | chr | | | | | | | | | | | | Pos | | | | | | | | | | | | id | | | | | | | | | | | | ref | | | | | | | | alt | | | | | | | | | gene | | | | | | | | | | | | | LR | | | | | | | | | | **Gene burden ratio** | | | | | | | | | | | | | | | | | | | | | | | | | | | **Total No. of alleles**  **in gene** | | | | | | | | | | | | | | | | | | | | | | | | | | **No. alt alleles**  **in genes** | | | | | | | | | | | | | | | | | | | | | | | | | | **No. of**  **cases with**  **alt alleles** | | | | | | | | | | | | | | | **Variant allele frequency** | | | | | | | | | | |
|  | | | | | | | |  | | | | | | | | | | | |  | | | | | | | | | | | |  | | | | | | | | | | | |  | | | | | | | |  | | | | | | | | |  | | | | | | | | | | | | |  | | | | | | | | | | KG  East Asia | | | | | | | | | | | | | | healthy  Ctrl | | | | | | | | | | | | | healthy  Ctrl | | | | | | | | | | | | | RA | | | | | | | | | | | | | healthy Ctrl | | | | | | | | | | | | | | RA | | | | | | | | | | | |  | | | | | | | | | | | | | | | KG  East Asia | | | | | | | | | | |
| 6 | | | 19 | | | | | | | | | | | 39591919 | | | | | | | | | | | | rs182155157 | | | | | | | | | | | | | C | | | | | | | | | | | | | | | | | G | | | | | | | | | | | | | ACP7 | | | | | | | | | | | | | | . | | | | | | | 1.92 | | | | | | | | | | | | 1.93 | | | | | | | | | | | | | 264 | | | | | | | | | | | | | 232 | | | | | | | | | | | | | | 10 | | | | | | | | | | | | | 17 | | | | | | | | | | | | | 16 | | | | | | | | | | | | | | 0.0387 | | | | | | |
| 6 | | | 19 | | | | | | | | | | | 39592099 | | | | | | | | | | | | rs186807855 | | | | | | | | | | | | | T | | | | | | | | | | | | | | | | | C | | | | | | | | | | | | | ACP7 | | | | | | | | | | | | | | . | | | | | | | 1.92 | | | | | | | | | | | | 1.93 | | | | | | | | | | | | | 264 | | | | | | | | | | | | | 232 | | | | | | | | | | | | | | 10 | | | | | | | | | | | | | 17 | | | | | | | | | | | | | 16 | | | | | | | | | | | | | | 0.0179 | | | | | | |
| 6 | | | 19 | | | | | | | | | | | 4099225 | | | | | | | | | | | | rs200371894 | | | | | | | | | | | | | G | | | | | | | | | | | | | | | | | A | | | | | | | | | | | | | MAP2K2 | | | | | | | | | | | | | | D | | | | | | | 1.81 | | | | | | | | | | | | 2.28 | | | | | | | | | | | | | 264 | | | | | | | | | | | | | 232 | | | | | | | | | | | | | | 1 | | | | | | | | | | | | | 2 | | | | | | | | | | | | | 2 | | | | | | | | | | | | | | 0.0119 | | | | | | |
| 6 | | | 19 | | | | | | | | | | | 4099246 | | | | | | | | | | | | . | | | | | | | | | | | | | G | | | | | | | | | | | | | | | | | A | | | | | | | | | | | | | MAP2K2 | | | | | | | | | | | | | | D | | | | | | | 1.81 | | | | | | | | | | | | 2.28 | | | | | | | | | | | | | 264 | | | | | | | | | | | | | 232 | | | | | | | | | | | | | | 1 | | | | | | | | | | | | | 2 | | | | | | | | | | | | | 2 | | | | | | | | | | | | | | . | | | | | | |
| 6 | | | 19 | | | | | | | | | | | 51582802 | | | | | | | | | | | | rs199715229 | | | | | | | | | | | | | C | | | | | | | | | | | | | | | | | T | | | | | | | | | | | | | KLK14 | | | | | | | | | | | | | | D | | | | | | | 2.72 | | | | | | | | | | | | 1.71 | | | | | | | | | | | | | 264 | | | | | | | | | | | | | 232 | | | | | | | | | | | | | | 2 | | | | | | | | | | | | | 3 | | | | | | | | | | | | | 3 | | | | | | | | | | | | | | 0.0119 | | | | | | |
| 6 | | | 19 | | | | | | | | | | | 51585822 | | | | | | | | | | | | rs769468261 | | | | | | | | | | | | | G | | | | | | | | | | | | | | | | | A | | | | | | | | | | | | | KLK14 | | | | | | | | | | | | | | . | | | | | | | 2.72 | | | | | | | | | | | | 1.71 | | | | | | | | | | | | | 264 | | | | | | | | | | | | | 232 | | | | | | | | | | | | | | 2 | | | | | | | | | | | | | 3 | | | | | | | | | | | | | 3 | | | | | | | | | | | | | | . | | | | | | |
| 6 | | | 19 | | | | | | | | | | | 51729577 | | | | | | | | | | | | rs201473304 | | | | | | | | | | | | | ACCCAACAAC  TGGTATCTTT | | | | | | | | | | | | | | | | | A | | | | | | | | | | | | | CD33 | | | | | | | | | | | | | | . | | | | | | | 2.11 | | | | | | | | | | | | 1.99 | | | | | | | | | | | | | 132 | | | | | | | | | | | | | 116 | | | | | | | | | | | | | | 4 | | | | | | | | | | | | | 7 | | | | | | | | | | | | | 7 | | | | | | | | | | | | | | 0.0258 | | | | | | |
| 6 | | | 19 | | | | | | | | | | | 53554075 | | | | | | | | | | | | rs1650983 | | | | | | | | | | | | | A | | | | | | | | | | | | | | | | | G | | | | | | | | | | | | | ERVV-2 | | | | | | | | | | | | | | . | | | | | | | 1.81 | | | | | | | | | | | | 4.55 | | | | | | | | | | | | | 132 | | | | | | | | | | | | | 116 | | | | | | | | | | | | | | 1 | | | | | | | | | | | | | 4 | | | | | | | | | | | | | 4 | | | | | | | | | | | | | | 0.0188 | | | | | | |
| 6 | | | 19 | | | | | | | | | | | 54872594 | | | | | | | | | | | | . | | | | | | | | | | | | | G | | | | | | | | | | | | | | | | | A | | | | | | | | | | | | | LAIR1 | | | | | | | | | | | | | | . | | | | | | | 1.81 | | | | | | | | | | | | 1.9 | | | | | | | | | | | | | 396 | | | | | | | | | | | | | 348 | | | | | | | | | | | | | | 9 | | | | | | | | | | | | | 15 | | | | | | | | | | | | | 15 | | | | | | | | | | | | | | 0.0317 | | | | | | |
| 6 | | | 19 | | | | | | | | | | | 54872611 | | | | | | | | | | | | . | | | | | | | | | | | | | A | | | | | | | | | | | | | | | | | T | | | | | | | | | | | | | LAIR1 | | | | | | | | | | | | | | . | | | | | | | 1.81 | | | | | | | | | | | | 1.9 | | | | | | | | | | | | | 396 | | | | | | | | | | | | | 348 | | | | | | | | | | | | | | 9 | | | | | | | | | | | | | 15 | | | | | | | | | | | | | 15 | | | | | | | | | | | | | | 0.0308 | | | | | | |
| 6 | | | 19 | | | | | | | | | | | 54872698 | | | | | | | | | | | | . | | | | | | | | | | | | | C | | | | | | | | | | | | | | | | | G | | | | | | | | | | | | | LAIR1 | | | | | | | | | | | | | | . | | | | | | | 1.81 | | | | | | | | | | | | 1.9 | | | | | | | | | | | | | 396 | | | | | | | | | | | | | 348 | | | | | | | | | | | | | | 9 | | | | | | | | | | | | | 15 | | | | | | | | | | | | | 15 | | | | | | | | | | | | | | 0.0308 | | | | | | |
| 6 | | | 19 | | | | | | | | | | | 55739813 | | | | | | | | | | | | rs10419308 | | | | | | | | | | | | | G | | | | | | | | | | | | | | | | | A | | | | | | | | | | | | | TMEM86B | | | | | | | | | | | | | | . | | | | | | | 1.53 | | | | | | | | | | | | 1.79 | | | | | | | | | | | | | 132 | | | | | | | | | | | | | 116 | | | | | | | | | | | | | | 7 | | | | | | | | | | | | | 11 | | | | | | | | | | | | | 11 | | | | | | | | | | | | | | 0.0704 | | | | | | |
| 6 | | | 19 | | | | | | | | | | | 57956740 | | | | | | | | | | | | rs148699125 | | | | | | | | | | | | | C | | | | | | | | | | | | | | | | | CA | | | | | | | | | | | | | ZNF749 | | | | | | | | | | | | | | . | | | | | | | 1.81 | | | | | | | | | | | | 6.83 | | | | | | | | | | | | | 132 | | | | | | | | | | | | | 116 | | | | | | | | | | | | | | 1 | | | | | | | | | | | | | 6 | | | | | | | | | | | | | 6 | | | | | | | | | | | | | | 0.0357 | | | | | | |
| 6 | | | 19 | | | | | | | | | | | 58118371 | | | | | | | | | | | | rs78803667 | | | | | | | | | | | | | G | | | | | | | | | | | | | | | | | A | | | | | | | | | | | | | ZNF530 | | | | | | | | | | | | | | D | | | | | | | 4.53 | | | | | | | | | | | | 1.9 | | | | | | | | | | | | | 132 | | | | | | | | | | | | | 116 | | | | | | | | | | | | | | 3 | | | | | | | | | | | | | 5 | | | | | | | | | | | | | 5 | | | | | | | | | | | | | | 0.0159 | | | | | | |
| 6 | | | 19 | | | | | | | | | | | 6147453 | | | | | | | | | | | | rs16993408 | | | | | | | | | | | | | G | | | | | | | | | | | | | | | | | C | | | | | | | | | | | | | ACSBG2 | | | | | | | | | | | | | | . | | | | | | | 12.07 | | | | | | | | | | | | 1.9 | | | | | | | | | | | | | 396 | | | | | | | | | | | | | 348 | | | | | | | | | | | | | | 6 | | | | | | | | | | | | | 10 | | | | | | | | | | | | | 9 | | | | | | | | | | | | | | 0.0119 | | | | | | |
| 6 | | | 19 | | | | | | | | | | | 6161219 | | | | | | | | | | | | rs78713134 | | | | | | | | | | | | | C | | | | | | | | | | | | | | | | | T | | | | | | | | | | | | | ACSBG2 | | | | | | | | | | | | | | . | | | | | | | 12.07 | | | | | | | | | | | | 1.9 | | | | | | | | | | | | | 396 | | | | | | | | | | | | | 348 | | | | | | | | | | | | | | 6 | | | | | | | | | | | | | 10 | | | | | | | | | | | | | 9 | | | | | | | | | | | | | | 0.0238 | | | | | | |
| group | | | | | | | | chr | | | | | | | | | | | | Pos | | | | | | | | | | | | id | | | | | | | | | | | | ref | | | | | | | | alt | | | | | | | | | gene | | | | | | | | | | | | | LR | | | | | | | | | | **Gene burden ratio** | | | | | | | | | | | | | | | | | | | | | | | | | | | **Total No. of alleles**  **in gene** | | | | | | | | | | | | | | | | | | | | | | | | | | **No. alt alleles**  **in genes** | | | | | | | | | | | | | | | | | | | | | | | | | | **No. of**  **cases with**  **alt alleles** | | | | | | | | | | | | | | | **Variant allele frequency** | | | | | | | | | | |
|  | | | | | | | |  | | | | | | | | | | | |  | | | | | | | | | | | |  | | | | | | | | | | | |  | | | | | | | |  | | | | | | | | |  | | | | | | | | | | | | |  | | | | | | | | | | KG  East Asia | | | | | | | | | | | | | | healthy  Ctrl | | | | | | | | | | | | | healthy  Ctrl | | | | | | | | | | | | | RA | | | | | | | | | | | | | healthy Ctrl | | | | | | | | | | | | | | RA | | | | | | | | | | | |  | | | | | | | | | | | | | | | KG  East Asia | | | | | | | | | | |
| 6 | | | | | | | | 20 | | | | | | | | | | | | 31040031 | | | | | | | | | | | | rs2236156 | | | | | | | | | | | | C | | | | | | | | T | | | | | | | | | NOL4L | | | | | | | | | | | | | . | | | | | | | | | | 5.43 | | | | | | | | | | | | | | . | | | | | | | | | | | | | 132 | | | | | | | | | | | | | 116 | | | | | | | | | | | | | 0 | | | | | | | | | | | | | | 3 | | | | | | | | | | | | 3 | | | | | | | | | | | | | | | 0.0248 | | | | | | | | | | |
| 6 | | | | | | | | 20 | | | | | | | | | | | | 31672812 | | | | | | | | | | | | rs71349705 | | | | | | | | | | | | C | | | | | | | | T | | | | | | | | | BPIFB4 | | | | | | | | | | | | | . | | | | | | | | | | 2.72 | | | | | | | | | | | | | | 1.71 | | | | | | | | | | | | | 264 | | | | | | | | | | | | | 232 | | | | | | | | | | | | | 2 | | | | | | | | | | | | | | 3 | | | | | | | | | | | | 3 | | | | | | | | | | | | | | | . | | | | | | | | | | |
| 6 | | | | | | | | 20 | | | | | | | | | | | | 31677295 | | | | | | | | | | | | rs142982767 | | | | | | | | | | | | C | | | | | | | | T | | | | | | | | | BPIFB4 | | | | | | | | | | | | | . | | | | | | | | | | 2.72 | | | | | | | | | | | | | | 1.71 | | | | | | | | | | | | | 264 | | | | | | | | | | | | | 232 | | | | | | | | | | | | | 2 | | | | | | | | | | | | | | 3 | | | | | | | | | | | | 3 | | | | | | | | | | | | | | | 0.0179 | | | | | | | | | | |
| 6 | | | | | | | | 20 | | | | | | | | | | | | 34572606 | | | | | | | | | | | | rs6142471 | | | | | | | | | | | | A | | | | | | | | G | | | | | | | | | CNBD2 | | | | | | | | | | | | | D | | | | | | | | | | 1.81 | | | | | | | | | | | | | | 4.55 | | | | | | | | | | | | | 132 | | | | | | | | | | | | | 116 | | | | | | | | | | | | | 1 | | | | | | | | | | | | | | 4 | | | | | | | | | | | | 3 | | | | | | | | | | | | | | | 0.0278 | | | | | | | | | | |
| 6 | | | | | | | | 20 | | | | | | | | | | | | 44511257 | | | | | | | | | | | | rs35972756 | | | | | | | | | | | | G | | | | | | | | A | | | | | | | | | ZSWIM1 | | | | | | | | | | | | | . | | | | | | | | | | 2.72 | | | | | | | | | | | | | | 1.71 | | | | | | | | | | | | | 132 | | | | | | | | | | | | | 116 | | | | | | | | | | | | | 2 | | | | | | | | | | | | | | 3 | | | | | | | | | | | | 3 | | | | | | | | | | | | | | | 0.0159 | | | | | | | | | | |
| 6 | | | | | | | | 20 | | | | | | | | | | | | 44676727 | | | | | | | | | | | | rs12481488 | | | | | | | | | | | | T | | | | | | | | A | | | | | | | | | SLC12A5 | | | | | | | | | | | | | . | | | | | | | | | | 1.81 | | | | | | | | | | | | | | 2.28 | | | | | | | | | | | | | 132 | | | | | | | | | | | | | 116 | | | | | | | | | | | | | 2 | | | | | | | | | | | | | | 4 | | | | | | | | | | | | 4 | | | | | | | | | | | | | | | 0.0238 | | | | | | | | | | |
| 6 | | | | | | | | 21 | | | | | | | | | | | | 43412786 | | | | | | | | | | | | rs200509586 | | | | | | | | | | | | GTCA | | | | | | | | G | | | | | | | | | ZBTB21 | | | | | | | | | | | | | . | | | | | | | | | | 5.43 | | | | | | | | | | | | | | 3.41 | | | | | | | | | | | | | 132 | | | | | | | | | | | | | 116 | | | | | | | | | | | | | 2 | | | | | | | | | | | | | | 6 | | | | | | | | | | | | 6 | | | | | | | | | | | | | | | 0.0109 | | | | | | | | | | |
| 6 | | | | | | | | 19 | | | | | | | | | | | | 6183085 | | | | | | | | | | | | . | | | | | | | | | | | | A | | | | | | | | ATAG | | | | | | | | | ACSBG2 | | | | | | | | | | | | | . | | | | | | | | | | 12.07 | | | | | | | | | | | | | | 1.9 | | | | | | | | | | | | | 396 | | | | | | | | | | | | | 348 | | | | | | | | | | | | | 6 | | | | | | | | | | | | | | 10 | | | | | | | | | | | | 9 | | | | | | | | | | | | | | | . | | | | | | | | | | |
| 6 | | | | | | | | 21 | | | | | | | | | | | | 44293806 | | | | | | | | | | | | rs146400491 | | | | | | | | | | | | G | | | | | | | | A | | | | | | | | | WDR4 | | | | | | | | | | | | | . | | | | | | | | | | 3.02 | | | | | | | | | | | | | | 5.69 | | | | | | | | | | | | | 132 | | | | | | | | | | | | | 116 | | | | | | | | | | | | | 1 | | | | | | | | | | | | | | 5 | | | | | | | | | | | | 4 | | | | | | | | | | | | | | | 0.0208 | | | | | | | | | | |
| 6 | | | | | | | | 21 | | | | | | | | | | | | 44488667 | | | | | | | | | | | | . | | | | | | | | | | | | C | | | | | | | | T | | | | | | | | | CBS | | | | | | | | | | | | | D | | | | | | | | | | 3.62 | | | | | | | | | | | | | | 2.28 | | | | | | | | | | | | | 264 | | | | | | | | | | | | | 232 | | | | | | | | | | | | | 2 | | | | | | | | | | | | | | 4 | | | | | | | | | | | | 4 | | | | | | | | | | | | | | | . | | | | | | | | | | |
| 6 | | | | | | | | 21 | | | | | | | | | | | | 44492252 | | | | | | | | | | | | rs201827340 | | | | | | | | | | | | G | | | | | | | | A | | | | | | | | | CBS | | | | | | | | | | | | | D | | | | | | | | | | 3.62 | | | | | | | | | | | | | | 2.28 | | | | | | | | | | | | | 264 | | | | | | | | | | | | | 232 | | | | | | | | | | | | | 2 | | | | | | | | | | | | | | 4 | | | | | | | | | | | | 4 | | | | | | | | | | | | | | | 0.0129 | | | | | | | | | | |
| 6 | | | | | | | | 2 | | | | | | | | | | | | 169791766 | | | | | | | | | | | | . | | | | | | | | | | | | G | | | | | | | | A | | | | | | | | | ABCB11 | | | | | | | | | | | | | D | | | | | | | | | | 3.02 | | | | | | | | | | | | | | 2.84 | | | | | | | | | | | | | 396 | | | | | | | | | | | | | 348 | | | | | | | | | | | | | 2 | | | | | | | | | | | | | | 5 | | | | | | | | | | | | 5 | | | | | | | | | | | | | | | . | | | | | | | | | | |
| 6 | | | | | | | | 2 | | | | | | | | | | | | 169801131 | | | | | | | | | | | | rs118109635 | | | | | | | | | | | | G | | | | | | | | A | | | | | | | | | ABCB11 | | | | | | | | | | | | | D | | | | | | | | | | 3.02 | | | | | | | | | | | | | | 2.84 | | | | | | | | | | | | | 396 | | | | | | | | | | | | | 348 | | | | | | | | | | | | | 2 | | | | | | | | | | | | | | 5 | | | | | | | | | | | | 5 | | | | | | | | | | | | | | | 0.0129 | | | | | | | | | | |
| 6 | | | | | | | | 2 | | | | | | | | | | | | 169853135 | | | | | | | | | | | | . | | | | | | | | | | | | A | | | | | | | | G | | | | | | | | | ABCB11 | | | | | | | | | | | | | . | | | | | | | | | | 3.02 | | | | | | | | | | | | | | 2.84 | | | | | | | | | | | | | 396 | | | | | | | | | | | | | 348 | | | | | | | | | | | | | 2 | | | | | | | | | | | | | | 5 | | | | | | | | | | | | 5 | | | | | | | | | | | | | | | . | | | | | | | | | | |
| 6 | | | | | | | | 2 | | | | | | | | | | | | 175304621 | | | | | | | | | | | | rs67227536 | | | | | | | | | | | | C | | | | | | | | G | | | | | | | | | GPR155 | | | | | | | | | | | | | . | | | | | | | | | | 3.62 | | | | | | | | | | | | | | 2.28 | | | | | | | | | | | | | 264 | | | | | | | | | | | | | 232 | | | | | | | | | | | | | 2 | | | | | | | | | | | | | | 4 | | | | | | | | | | | | 4 | | | | | | | | | | | | | | | 0.0139 | | | | | | | | | | |
| 6 | | | | | | | | 2 | | | | | | | | | | | | 175333632 | | | | | | | | | | | | rs28588913 | | | | | | | | | | | | G | | | | | | | | A | | | | | | | | | GPR155 | | | | | | | | | | | | | . | | | | | | | | | | 3.62 | | | | | | | | | | | | | | 2.28 | | | | | | | | | | | | | 264 | | | | | | | | | | | | | 232 | | | | | | | | | | | | | 2 | | | | | | | | | | | | | | 4 | | | | | | | | | | | | 4 | | | | | | | | | | | | | | | 0.0248 | | | | | | | | | | |
| 6 | | | | | | | | 22 | | | | | | | | | | | | 25603008 | | | | | | | | | | | | rs13055430 | | | | | | | | | | | | C | | | | | | | | T | | | | | | | | | CRYBB3 | | | | | | | | | | | | | . | | | | | | | | | | 3.62 | | | | | | | | | | | | | | 3.03 | | | | | | | | | | | | | 132 | | | | | | | | | | | | | 116 | | | | | | | | | | | | | 3 | | | | | | | | | | | | | | 8 | | | | | | | | | | | | 8 | | | | | | | | | | | | | | | 0.0268 | | | | | | | | | | |
| 6 | | | | | | | | 22 | | | | | | | | | | | | 31521167 | | | | | | | | | | | | rs150867939 | | | | | | | | | | | | C | | | | | | | | T | | | | | | | | | INPP5J | | | | | | | | | | | | | D | | | | | | | | | | 3.62 | | | | | | | | | | | | | | 2.28 | | | | | | | | | | | | | 924 | | | | | | | | | | | | | 812 | | | | | | | | | | | | | 3 | | | | | | | | | | | | | | 6 | | | | | | | | | | | | 6 | | | | | | | | | | | | | | | 0.0129 | | | | | | | | | | |
| group | | | | | | | chr | | | | | | | | | | | | Pos | | | | | | | | | | | | id | | | | | | | | | | | | ref | | | | | | | | | | | alt | | | | | | | | gene | | | | | | | | | | | | | LR | | | | | | | | | | | **Gene burden ratio** | | | | | | | | | | | | | | | | | | | | | | | | | | **Total No. of alleles**  **in gene** | | | | | | | | | | | | | | | | | | | | | | | | | | **No. alt alleles**  **in genes** | | | | | | | | | | | | | | | | | | | | | | | | | | **No. of**  **cases with**  **alt alleles** | | | | | | | | | | | | | | | **Variant allele frequency** | | | | | | | | | |
|  | | | | | | |  | | | | | | | | | | | |  | | | | | | | | | | | |  | | | | | | | | | | | |  | | | | | | | | | | |  | | | | | | | |  | | | | | | | | | | | | |  | | | | | | | | | | | KG  East Asia | | | | | | | | | | | | | healthy  Ctrl | | | | | | | | | | | | | healthy  Ctrl | | | | | | | | | | | | | RA | | | | | | | | | | | | | healthy Ctrl | | | | | | | | | | | | | | RA | | | | | | | | | | | |  | | | | | | | | | | | | | | | KG  East Asia | | | | | | | | | |
| 6 | | | | | | | | 22 | | | | | | | | | | | | 31521324 | | | | | | | | | | | | . | | | | | | | | | | | | C | | | | | | | | A | | | | | | | | | INPP5J | | | | | | | | | | | | | D | | | | | | | | | | 3.62 | | | | | | | | | | | | | | 2.28 | | | | | | | | | | | | | 924 | | | | | | | | | | | | | 812 | | | | | | | | | | | | | 3 | | | | | | | | | | | | | | 6 | | | | | | | | | | | | 6 | | | | | | | | | | | | | | | . | | | | | | | | | | |
| 6 | | | | | | | | 22 | | | | | | | | | | | | 31521552 | | | | | | | | | | | | rs774897780 | | | | | | | | | | | | G | | | | | | | | A | | | | | | | | | INPP5J | | | | | | | | | | | | | D | | | | | | | | | | 3.62 | | | | | | | | | | | | | | 2.28 | | | | | | | | | | | | | 924 | | | | | | | | | | | | | 812 | | | | | | | | | | | | | 3 | | | | | | | | | | | | | | 6 | | | | | | | | | | | | 6 | | | | | | | | | | | | | | | . | | | | | | | | | | |
| 6 | | | | | | | | 22 | | | | | | | | | | | | 31522468 | | | | | | | | | | | | rs767028605 | | | | | | | | | | | | G | | | | | | | | A | | | | | | | | | INPP5J | | | | | | | | | | | | | D | | | | | | | | | | 3.62 | | | | | | | | | | | | | | 2.28 | | | | | | | | | | | | | 924 | | | | | | | | | | | | | 812 | | | | | | | | | | | | | 3 | | | | | | | | | | | | | | 6 | | | | | | | | | | | | 6 | | | | | | | | | | | | | | | . | | | | | | | | | | |
| 6 | | | | | | | | 22 | | | | | | | | | | | | 31524007 | | | | | | | | | | | | rs769593351 | | | | | | | | | | | | A | | | | | | | | G | | | | | | | | | INPP5J | | | | | | | | | | | | | D | | | | | | | | | | 3.62 | | | | | | | | | | | | | | 2.28 | | | | | | | | | | | | | 924 | | | | | | | | | | | | | 812 | | | | | | | | | | | | | 3 | | | | | | | | | | | | | | 6 | | | | | | | | | | | | 6 | | | | | | | | | | | | | | | . | | | | | | | | | | |
| 6 | | | | | | | | 22 | | | | | | | | | | | | 31524578 | | | | | | | | | | | | rs202068549 | | | | | | | | | | | | C | | | | | | | | T | | | | | | | | | INPP5J | | | | | | | | | | | | | D | | | | | | | | | | 3.62 | | | | | | | | | | | | | | 2.28 | | | | | | | | | | | | | 924 | | | | | | | | | | | | | 812 | | | | | | | | | | | | | 3 | | | | | | | | | | | | | | 6 | | | | | | | | | | | | 6 | | | | | | | | | | | | | | | . | | | | | | | | | | |
| 6 | | | | | | | | 22 | | | | | | | | | | | | 31530095 | | | | | | | | | | | | . | | | | | | | | | | | | G | | | | | | | | C | | | | | | | | | INPP5J | | | | | | | | | | | | | D | | | | | | | | | | 3.62 | | | | | | | | | | | | | | 2.28 | | | | | | | | | | | | | 924 | | | | | | | | | | | | | 812 | | | | | | | | | | | | | 3 | | | | | | | | | | | | | | 6 | | | | | | | | | | | | 6 | | | | | | | | | | | | | | | . | | | | | | | | | | |
| 6 | | | | | | | | 2 | | | | | | | | | | | | 232458085 | | | | | | | | | | | | rs145183277 | | | | | | | | | | | | TGAGA | | | | | | | | T | | | | | | | | | C2orf57 | | | | | | | | | | | | | . | | | | | | | | | | 2.09 | | | | | | | | | | | | | | 2.84 | | | | | | | | | | | | | 132 | | | | | | | | | | | | | 116 | | | | | | | | | | | | | 6 | | | | | | | | | | | | | | 15 | | | | | | | | | | | | 15 | | | | | | | | | | | | | | | 0.0536 | | | | | | | | | | |
| 6 | | | | | | | | 22 | | | | | | | | | | | | 32992729 | | | | | | | | | | | | rs191241866 | | | | | | | | | | | | G | | | | | | | | A | | | | | | | | | SYN3 | | | | | | | | | | | | | . | | | | | | | | | | 5.43 | | | | | | | | | | | | | | . | | | | | | | | | | | | | 132 | | | | | | | | | | | | | 116 | | | | | | | | | | | | | 0 | | | | | | | | | | | | | | 3 | | | | | | | | | | | | 3 | | | | | | | | | | | | | | | 0.0129 | | | | | | | | | | |
| 6 | | | | | | | | 2 | | | | | | | | | | | | 237246998 | | | | | | | | | | | | . | | | | | | | | | | | | G | | | | | | | | T | | | | | | | | | IQCA1 | | | | | | | | | | | | | D | | | | | | | | | | 2.72 | | | | | | | | | | | | | | 1.71 | | | | | | | | | | | | | 264 | | | | | | | | | | | | | 232 | | | | | | | | | | | | | 2 | | | | | | | | | | | | | | 3 | | | | | | | | | | | | 3 | | | | | | | | | | | | | | | . | | | | | | | | | | |
| 6 | | | | | | | | 2 | | | | | | | | | | | | 237247013 | | | | | | | | | | | | rs186626813 | | | | | | | | | | | | G | | | | | | | | A | | | | | | | | | IQCA1 | | | | | | | | | | | | | D | | | | | | | | | | 2.72 | | | | | | | | | | | | | | 1.71 | | | | | | | | | | | | | 264 | | | | | | | | | | | | | 232 | | | | | | | | | | | | | 2 | | | | | | | | | | | | | | 3 | | | | | | | | | | | | 3 | | | | | | | | | | | | | | | 0.0129 | | | | | | | | | | |
| 6 | | | | | | | | 2 | | | | | | | | | | | | 239344663 | | | | | | | | | | | | rs11904390 | | | | | | | | | | | | T | | | | | | | | A | | | | | | | | | ASB1 | | | | | | | | | | | | | . | | | | | | | | | | 1.97 | | | | | | | | | | | | | | 3.41 | | | | | | | | | | | | | 132 | | | | | | | | | | | | | 116 | | | | | | | | | | | | | 4 | | | | | | | | | | | | | | 12 | | | | | | | | | | | | 12 | | | | | | | | | | | | | | | 0.0595 | | | | | | | | | | |
| 6 | | | | | | | | 22 | | | | | | | | | | | | 40801855 | | | | | | | | | | | | rs188579679 | | | | | | | | | | | | C | | | | | | | | T | | | | | | | | | SGSM3 | | | | | | | | | | | | | . | | | | | | | | | | 2.72 | | | | | | | | | | | | | | . | | | | | | | | | | | | | 132 | | | | | | | | | | | | | 116 | | | | | | | | | | | | | 0 | | | | | | | | | | | | | | 6 | | | | | | | | | | | | 6 | | | | | | | | | | | | | | | 0.0169 | | | | | | | | | | |
| 6 | | | | | | | | 22 | | | | | | | | | | | | 43933284 | | | | | | | | | | | | rs3833393 | | | | | | | | | | | | CCT | | | | | | | | C | | | | | | | | | EFCAB6 | | | | | | | | | | | | | . | | | | | | | | | | 1.63 | | | | | | | | | | | | | | 2.28 | | | | | | | | | | | | | 264 | | | | | | | | | | | | | 232 | | | | | | | | | | | | | 2 | | | | | | | | | | | | | | 4 | | | | | | | | | | | | 4 | | | | | | | | | | | | | | | 0.0159 | | | | | | | | | | |
| 6 | | | | | | | | 22 | | | | | | | | | | | | 44131813 | | | | | | | | | | | | . | | | | | | | | | | | | C | | | | | | | | T | | | | | | | | | EFCAB6 | | | | | | | | | | | | | D | | | | | | | | | | 1.63 | | | | | | | | | | | | | | 2.28 | | | | | | | | | | | | | 264 | | | | | | | | | | | | | 232 | | | | | | | | | | | | | 2 | | | | | | | | | | | | | | 4 | | | | | | | | | | | | 4 | | | | | | | | | | | | | | | . | | | | | | | | | | |
| 6 | | | | | | | | 22 | | | | | | | | | | | | 46668317 | | | | | | | | | | | | rs779119363 | | | | | | | | | | | | A | | | | | | | | G | | | | | | | | | TTC38 | | | | | | | | | | | | | . | | | | | | | | | | 3.62 | | | | | | | | | | | | | | 1.71 | | | | | | | | | | | | | 396 | | | | | | | | | | | | | 348 | | | | | | | | | | | | | 2 | | | | | | | | | | | | | | 3 | | | | | | | | | | | | 3 | | | | | | | | | | | | | | | . | | | | | | | | | | |
| 6 | | | | | | | | 22 | | | | | | | | | | | | 46679924 | | | | | | | | | | | | rs201314224 | | | | | | | | | | | | G | | | | | | | | C | | | | | | | | | TTC38 | | | | | | | | | | | | | D | | | | | | | | | | 3.62 | | | | | | | | | | | | | | 1.71 | | | | | | | | | | | | | 396 | | | | | | | | | | | | | 348 | | | | | | | | | | | | | 2 | | | | | | | | | | | | | | 3 | | | | | | | | | | | | 3 | | | | | | | | | | | | | | | . | | | | | | | | | | |
| 6 | | | | | | | | 22 | | | | | | | | | | | | 46684341 | | | | | | | | | | | | rs763990471 | | | | | | | | | | | | G | | | | | | | | A | | | | | | | | | TTC38 | | | | | | | | | | | | | . | | | | | | | | | | 3.62 | | | | | | | | | | | | | | 1.71 | | | | | | | | | | | | | 396 | | | | | | | | | | | | | 348 | | | | | | | | | | | | | 2 | | | | | | | | | | | | | | 3 | | | | | | | | | | | | 3 | | | | | | | | | | | | | | | . | | | | | | | | | | |
| 6 | | | | | | | | 22 | | | | | | | | | | | | 50754445 | | | | | | | | | | | | rs80243206 | | | | | | | | | | | | A | | | | | | | | T | | | | | | | | | DENND6B | | | | | | | | | | | | | . | | | | | | | | | | 1.51 | | | | | | | | | | | | | | 2.84 | | | | | | | | | | | | | 264 | | | | | | | | | | | | | 232 | | | | | | | | | | | | | 2 | | | | | | | | | | | | | | 5 | | | | | | | | | | | | 5 | | | | | | | | | | | | | | | 0.0198 | | | | | | | | | | |
| group | | | | | | | chr | | | | | | | | | | | | Pos | | | | | | | | | | | | id | | | | | | | | | | | | ref | | | | | | | | | | | alt | | | | | | | | gene | | | | | | | | | | | | | LR | | | | | | | | | | | **Gene burden ratio** | | | | | | | | | | | | | | | | | | | | | | | | | | **Total No. of alleles**  **in gene** | | | | | | | | | | | | | | | | | | | | | | | | | | **No. alt alleles**  **in genes** | | | | | | | | | | | | | | | | | | | | | | | | | | **No. of**  **cases with**  **alt alleles** | | | | | | | | | | | | | | | **Variant allele frequency** | | | | | | | | | |
|  | | | | | | |  | | | | | | | | | | | |  | | | | | | | | | | | |  | | | | | | | | | | | |  | | | | | | | | | | |  | | | | | | | |  | | | | | | | | | | | | |  | | | | | | | | | | | KG  East Asia | | | | | | | | | | | | | healthy  Ctrl | | | | | | | | | | | | | healthy  Ctrl | | | | | | | | | | | | | RA | | | | | | | | | | | | | healthy Ctrl | | | | | | | | | | | | | | RA | | | | | | | | | | | |  | | | | | | | | | | | | | | | KG  East Asia | | | | | | | | | |
| 6 | | | | | | | 22 | | | | | | | | | | | | 50756452 | | | | | | | | | | | | rs73439320 | | | | | | | | | | | | A | | | | | | | | | | | G | | | | | | | | DENND6B | | | | | | | | | | | | | . | | | | | | | | | | | 1.51 | | | | | | | | | | | | | 2.84 | | | | | | | | | | | | | 264 | | | | | | | | | | | | | 232 | | | | | | | | | | | | | 2 | | | | | | | | | | | | | | 5 | | | | | | | | | | | | 5 | | | | | | | | | | | | | | | 0.0208 | | | | | | | | | |
| 6 | | | | | | | 2 | | | | | | | | | | | | 27729343 | | | | | | | | | | | | . | | | | | | | | | | | | C | | | | | | | | | | | A | | | | | | | | GCKR | | | | | | | | | | | | | . | | | | | | | | | | | 5.43 | | | | | | | | | | | | | 2.28 | | | | | | | | | | | | | 264 | | | | | | | | | | | | | 232 | | | | | | | | | | | | | 1 | | | | | | | | | | | | | | 2 | | | | | | | | | | | | 2 | | | | | | | | | | | | | | | . | | | | | | | | | |
| 6 | | | | | | | 2 | | | | | | | | | | | | 27729453 | | | | | | | | | | | | . | | | | | | | | | | | | G | | | | | | | | | | | A | | | | | | | | GCKR | | | | | | | | | | | | | . | | | | | | | | | | | 5.43 | | | | | | | | | | | | | 2.28 | | | | | | | | | | | | | 264 | | | | | | | | | | | | | 232 | | | | | | | | | | | | | 1 | | | | | | | | | | | | | | 2 | | | | | | | | | | | | 2 | | | | | | | | | | | | | | | . | | | | | | | | | |
| 6 | | | | | | | 2 | | | | | | | | | | | | 28634790 | | | | | | | | | | | | rs12624279 | | | | | | | | | | | | G | | | | | | | | | | | A | | | | | | | | FOSL2 | | | | | | | | | | | | | . | | | | | | | | | | | 1.65 | | | | | | | | | | | | | 1.63 | | | | | | | | | | | | | 132 | | | | | | | | | | | | | 116 | | | | | | | | | | | | | 21 | | | | | | | | | | | | | | 30 | | | | | | | | | | | | 25 | | | | | | | | | | | | | | | 0.1835 | | | | | | | | | |
| 6 | | | | | | | 2 | | | | | | | | | | | | 31412347 | | | | | | | | | | | | rs78099670 | | | | | | | | | | | | G | | | | | | | | | | | A | | | | | | | | CAPN14 | | | | | | | | | | | | | . | | | | | | | | | | | 5.07 | | | | | | | | | | | | | 1.59 | | | | | | | | | | | | | 660 | | | | | | | | | | | | | 580 | | | | | | | | | | | | | 5 | | | | | | | | | | | | | | 7 | | | | | | | | | | | | 7 | | | | | | | | | | | | | | | 0.0179 | | | | | | | | | |
| 6 | | | | | | | 2 | | | | | | | | | | | | 31414833 | | | | | | | | | | | | . | | | | | | | | | | | | G | | | | | | | | | | | T | | | | | | | | CAPN14 | | | | | | | | | | | | | D | | | | | | | | | | | 5.07 | | | | | | | | | | | | | 1.59 | | | | | | | | | | | | | 660 | | | | | | | | | | | | | 580 | | | | | | | | | | | | | 5 | | | | | | | | | | | | | | 7 | | | | | | | | | | | | 7 | | | | | | | | | | | | | | | . | | | | | | | | | |
| 6 | | | | | | | 2 | | | | | | | | | | | | 31414844 | | | | | | | | | | | | rs147299374 | | | | | | | | | | | | C | | | | | | | | | | | T | | | | | | | | CAPN14 | | | | | | | | | | | | | D | | | | | | | | | | | 5.07 | | | | | | | | | | | | | 1.59 | | | | | | | | | | | | | 660 | | | | | | | | | | | | | 580 | | | | | | | | | | | | | 5 | | | | | | | | | | | | | | 7 | | | | | | | | | | | | 7 | | | | | | | | | | | | | | | . | | | | | | | | | |
| 6 | | | | | | | 2 | | | | | | | | | | | | 31414959 | | | | | | | | | | | | rs141014145 | | | | | | | | | | | | A | | | | | | | | | | | G | | | | | | | | CAPN14 | | | | | | | | | | | | | D | | | | | | | | | | | 5.07 | | | | | | | | | | | | | 1.59 | | | | | | | | | | | | | 660 | | | | | | | | | | | | | 580 | | | | | | | | | | | | | 5 | | | | | | | | | | | | | | 7 | | | | | | | | | | | | 7 | | | | | | | | | | | | | | | 0.0149 | | | | | | | | | |
| 6 | | | | | | | 2 | | | | | | | | | | | | 31422395 | | | | | | | | | | | | rs200657395 | | | | | | | | | | | | TCTC | | | | | | | | | | | T | | | | | | | | CAPN14 | | | | | | | | | | | | | . | | | | | | | | | | | 5.07 | | | | | | | | | | | | | 1.59 | | | | | | | | | | | | | 660 | | | | | | | | | | | | | 580 | | | | | | | | | | | | | 5 | | | | | | | | | | | | | | 7 | | | | | | | | | | | | 7 | | | | | | | | | | | | | | | 0.0139 | | | | | | | | | |
| 6 | | | | | | | 2 | | | | | | | | | | | | 47399601 | | | | | | | | | | | | rs4953472 | | | | | | | | | | | | A | | | | | | | | | | | G | | | | | | | | CALM2 | | | | | | | | | | | | | . | | | | | | | | | | | 3.62 | | | | | | | | | | | | | 1.71 | | | | | | | | | | | | | 132 | | | | | | | | | | | | | 116 | | | | | | | | | | | | | 4 | | | | | | | | | | | | | | 6 | | | | | | | | | | | | 6 | | | | | | | | | | | | | | | 0.0327 | | | | | | | | | |
| 6 | | | | | | | 3 | | | | | | | | | | | | 107097080 | | | | | | | | | | | | rs138204694 | | | | | | | | | | | | CAAATG | | | | | | | | | | | C | | | | | | | | CCDC54 | | | | | | | | | | | | | . | | | | | | | | | | | 1.81 | | | | | | | | | | | | | 7.97 | | | | | | | | | | | | | 132 | | | | | | | | | | | | | 116 | | | | | | | | | | | | | 1 | | | | | | | | | | | | | | 7 | | | | | | | | | | | | 7 | | | | | | | | | | | | | | | 0.0317 | | | | | | | | | |
| 6 | | | | | | | 3 | | | | | | | | | | | | 111828384 | | | | | | | | | | | | rs397949663 | | | | | | | | | | | | G | | | | | | | | | | | GT | | | | | | | | C3orf52 | | | | | | | | | | | | | . | | | | | | | | | | | 1.56 | | | | | | | | | | | | | 1.54 | | | | | | | | | | | | | 132 | | | | | | | | | | | | | 116 | | | | | | | | | | | | | 28 | | | | | | | | | | | | | | 38 | | | | | | | | | | | | 33 | | | | | | | | | | | | | | | 0.2569 | | | | | | | | | |
| 6 | | | | | | | 3 | | | | | | | | | | | | 119242443 | | | | | | | | | | | | rs58978800 | | | | | | | | | | | | C | | | | | | | | | | | T | | | | | | | | TIMMDC1 | | | | | | | | | | | | | . | | | | | | | | | | | 1.81 | | | | | | | | | | | | | 4.55 | | | | | | | | | | | | | 132 | | | | | | | | | | | | | 116 | | | | | | | | | | | | | 1 | | | | | | | | | | | | | | 4 | | | | | | | | | | | | 4 | | | | | | | | | | | | | | | 0.0258 | | | | | | | | | |
| 6 | | | | | | | 3 | | | | | | | | | | | | 122459290 | | | | | | | | | | | | rs16338 | | | | | | | | | | | | G | | | | | | | | | | | GAGA | | | | | | | | HSPBAP1 | | | | | | | | | | | | | . | | | | | | | | | | | 1.93 | | | | | | | | | | | | | 1.52 | | | | | | | | | | | | | 264 | | | | | | | | | | | | | 232 | | | | | | | | | | | | | 24 | | | | | | | | | | | | | | 32 | | | | | | | | | | | | 30 | | | | | | | | | | | | | | | 0.126 | | | | | | | | | |
| 6 | | | | | | | 3 | | | | | | | | | | | | 122459732 | | | | | | | | | | | | rs35887395 | | | | | | | | | | | | G | | | | | | | | | | | A | | | | | | | | HSPBAP1 | | | | | | | | | | | | | . | | | | | | | | | | | 1.93 | | | | | | | | | | | | | 1.52 | | | | | | | | | | | | | 264 | | | | | | | | | | | | | 232 | | | | | | | | | | | | | 24 | | | | | | | | | | | | | | 32 | | | | | | | | | | | | 30 | | | | | | | | | | | | | | | 0.126 | | | | | | | | | |
| 6 | | | | | | | 3 | | | | | | | | | | | | 169546730 | | | | | | | | | | | | rs149140811 | | | | | | | | | | | | C | | | | | | | | | | | T | | | | | | | | LRRIQ4 | | | | | | | | | | | | | . | | | | | | | | | | | 2.11 | | | | | | | | | | | | | 1.99 | | | | | | | | | | | | | 132 | | | | | | | | | | | | | 116 | | | | | | | | | | | | | 4 | | | | | | | | | | | | | | 7 | | | | | | | | | | | | 6 | | | | | | | | | | | | | | | 0.0357 | | | | | | | | | |
| 6 | | | | | | | 3 | | | | | | | | | | | | 183908937 | | | | | | | | | | | | rs765039315 | | | | | | | | | | | | C | | | | | | | | | | | T | | | | | | | | ABCF3 | | | | | | | | | | | | | D | | | | | | | | | | | 5.43 | | | | | | | | | | | | | 1.71 | | | | | | | | | | | | | 396 | | | | | | | | | | | | | 348 | | | | | | | | | | | | | 2 | | | | | | | | | | | | | | 3 | | | | | | | | | | | | 3 | | | | | | | | | | | | | | | . | | | | | | | | | |
| 6 | | | | | | | 3 | | | | | | | | | | | | 183910604 | | | | | | | | | | | | rs118183801 | | | | | | | | | | | | T | | | | | | | | | | | C | | | | | | | | ABCF3 | | | | | | | | | | | | | D | | | | | | | | | | | 5.43 | | | | | | | | | | | | | 1.71 | | | | | | | | | | | | | 396 | | | | | | | | | | | | | 348 | | | | | | | | | | | | | 2 | | | | | | | | | | | | | | 3 | | | | | | | | | | | | 3 | | | | | | | | | | | | | | | 0.0169 | | | | | | | | | |
| group | | | | | | | | chr | | | | | | | | | | | | Pos | | | | | | | | | | | | id | | | | | | | | | | | | ref | | | | | | | | alt | | | | | | | | | gene | | | | | | | | | | | | | LR | | | | | | | | | | **Gene burden ratio** | | | | | | | | | | | | | | | | | | | | | | | | | | | **Total No. of alleles**  **in gene** | | | | | | | | | | | | | | | | | | | | | | | | | | **No. alt alleles**  **in genes** | | | | | | | | | | | | | | | | | | | | | | | | | | **No. of**  **cases with**  **alt alleles** | | | | | | | | | | | | | | | **Variant allele frequency** | | | | | | | | | | |
|  | | | | | | | |  | | | | | | | | | | | |  | | | | | | | | | | | |  | | | | | | | | | | | |  | | | | | | | |  | | | | | | | | |  | | | | | | | | | | | | |  | | | | | | | | | | KG  East Asia | | | | | | | | | | | | | | healthy  Ctrl | | | | | | | | | | | | | healthy  Ctrl | | | | | | | | | | | | | RA | | | | | | | | | | | | | healthy Ctrl | | | | | | | | | | | | | | RA | | | | | | | | | | | |  | | | | | | | | | | | | | | | KG  East Asia | | | | | | | | | | |
| 6 | | | | | | | 3 | | | | | | | | | | | | 183911455 | | | | | | | | | | | | . | | | | | | | | | | | | A | | | | | | | | | | | G | | | | | | | | ABCF3 | | | | | | | | | | | | | D | | | | | | | | | | | 5.43 | | | | | | | | | | | | | 1.71 | | | | | | | | | | | | | 396 | | | | | | | | | | | | | 348 | | | | | | | | | | | | | 2 | | | | | | | | | | | | | | 3 | | | | | | | | | | | | 3 | | | | | | | | | | | | | | | . | | | | | | | | | |
| 6 | | | | | | | 3 | | | | | | | | | | | | 187451313 | | | | | | | | | | | | rs140944763 | | | | | | | | | | | | T | | | | | | | | | | | A | | | | | | | | BCL6 | | | | | | | | | | | | | . | | | | | | | | | | | 1.55 | | | | | | | | | | | | | 6.83 | | | | | | | | | | | | | 132 | | | | | | | | | | | | | 116 | | | | | | | | | | | | | 1 | | | | | | | | | | | | | | 6 | | | | | | | | | | | | 6 | | | | | | | | | | | | | | | 0.0228 | | | | | | | | | |
| 6 | | | | | | | 3 | | | | | | | | | | | | 32030579 | | | | | | | | | | | | rs373566244 | | | | | | | | | | | | GT | | | | | | | | | | | G | | | | | | | | ZNF860 | | | | | | | | | | | | | . | | | | | | | | | | | 2.72 | | | | | | | | | | | | | 1.71 | | | | | | | | | | | | | 132 | | | | | | | | | | | | | 116 | | | | | | | | | | | | | 2 | | | | | | | | | | | | | | 3 | | | | | | | | | | | | 3 | | | | | | | | | | | | | | | 0.0129 | | | | | | | | | |
| 6 | | | | | | | 3 | | | | | | | | | | | | 50219709 | | | | | | | | | | | | rs12639175 | | | | | | | | | | | | A | | | | | | | | | | | G | | | | | | | | SEMA3F | | | | | | | | | | | | | . | | | | | | | | | | | 1.81 | | | | | | | | | | | | | 2.28 | | | | | | | | | | | | | 132 | | | | | | | | | | | | | 116 | | | | | | | | | | | | | 1 | | | | | | | | | | | | | | 2 | | | | | | | | | | | | 2 | | | | | | | | | | | | | | | 0.0129 | | | | | | | | | |
| 6 | | | | | | | 4 | | | | | | | | | | | | 121719584 | | | | | | | | | | | | rs35363618 | | | | | | | | | | | | T | | | | | | | | | | | TA | | | | | | | | PRDM5 | | | | | | | | | | | | | . | | | | | | | | | | | 1.77 | | | | | | | | | | | | | 1.99 | | | | | | | | | | | | | 132 | | | | | | | | | | | | | 116 | | | | | | | | | | | | | 24 | | | | | | | | | | | | | | 42 | | | | | | | | | | | | 34 | | | | | | | | | | | | | | | 0.1885 | | | | | | | | | |
| 6 | | | | | | | 4 | | | | | | | | | | | | 16181283 | | | | | | | | | | | | rs78942971 | | | | | | | | | | | | A | | | | | | | | | | | G | | | | | | | | TAPT1 | | | | | | | | | | | | | . | | | | | | | | | | | 2.9 | | | | | | | | | | | | | 2.25 | | | | | | | | | | | | | 98 | | | | | | | | | | | | | 116 | | | | | | | | | | | | | 3 | | | | | | | | | | | | | | 8 | | | | | | | | | | | | 8 | | | | | | | | | | | | | | | 0.0248 | | | | | | | | | |
| 6 | | | | | | | 4 | | | | | | | | | | | | 177109395 | | | | | | | | | | | | rs200650536 | | | | | | | | | | | | T | | | | | | | | | | | G | | | | | | | | SPATA4 | | | | | | | | | | | | | . | | | | | | | | | | | 2.72 | | | | | | | | | | | | | 3.39 | | | | | | | | | | | | | 262 | | | | | | | | | | | | | 232 | | | | | | | | | | | | | 1 | | | | | | | | | | | | | | 3 | | | | | | | | | | | | 3 | | | | | | | | | | | | | | | 0.0149 | | | | | | | | | |
| 6 | | | | | | | 4 | | | | | | | | | | | | 177116495 | | | | | | | | | | | | . | | | | | | | | | | | | C | | | | | | | | | | | A | | | | | | | | SPATA4 | | | | | | | | | | | | | . | | | | | | | | | | | 2.72 | | | | | | | | | | | | | 3.39 | | | | | | | | | | | | | 262 | | | | | | | | | | | | | 232 | | | | | | | | | | | | | 1 | | | | | | | | | | | | | | 3 | | | | | | | | | | | | 3 | | | | | | | | | | | | | | | . | | | | | | | | | |
| 6 | | | | | | | 4 | | | | | | | | | | | | 2233893 | | | | | | | | | | | | rs117602484 | | | | | | | | | | | | A | | | | | | | | | | | G | | | | | | | | HAUS3 | | | | | | | | | | | | | . | | | | | | | | | | | 3.32 | | | | | | | | | | | | | 1.66 | | | | | | | | | | | | | 210 | | | | | | | | | | | | | 190 | | | | | | | | | | | | | 2 | | | | | | | | | | | | | | 3 | | | | | | | | | | | | 3 | | | | | | | | | | | | | | | 0.0119 | | | | | | | | | |
| 6 | | | | | | | 4 | | | | | | | | | | | | 2240347 | | | | | | | | | | | | rs376063631 | | | | | | | | | | | | C | | | | | | | | | | | T | | | | | | | | HAUS3 | | | | | | | | | | | | | D | | | | | | | | | | | 3.32 | | | | | | | | | | | | | 1.66 | | | | | | | | | | | | | 210 | | | | | | | | | | | | | 190 | | | | | | | | | | | | | 2 | | | | | | | | | | | | | | 3 | | | | | | | | | | | | 3 | | | | | | | | | | | | | | | . | | | | | | | | | |
| 6 | | | | | | | 4 | | | | | | | | | | | | 69094459 | | | | | | | | | | | | rs75647314 | | | | | | | | | | | | C | | | | | | | | | | | A | | | | | | | | TMPRSS11B | | | | | | | | | | | | | . | | | | | | | | | | | 2.11 | | | | | | | | | | | | | 1.59 | | | | | | | | | | | | | 264 | | | | | | | | | | | | | 232 | | | | | | | | | | | | | 5 | | | | | | | | | | | | | | 7 | | | | | | | | | | | | 7 | | | | | | | | | | | | | | | 0.0278 | | | | | | | | | |
| 6 | | | | | | | 4 | | | | | | | | | | | | 69096987 | | | | | | | | | | | | rs575638339 | | | | | | | | | | | | C | | | | | | | | | | | T | | | | | | | | TMPRSS11B | | | | | | | | | | | | | D | | | | | | | | | | | 2.11 | | | | | | | | | | | | | 1.59 | | | | | | | | | | | | | 264 | | | | | | | | | | | | | 232 | | | | | | | | | | | | | 5 | | | | | | | | | | | | | | 7 | | | | | | | | | | | | 7 | | | | | | | | | | | | | | | . | | | | | | | | | |
| 6 | | | | | | | 4 | | | | | | | | | | | | 71888240 | | | | | | | | | | | | rs67437265 | | | | | | | | | | | | C | | | | | | | | | | | T | | | | | | | | DCK | | | | | | | | | | | | | D | | | | | | | | | | | 1.81 | | | | | | | | | | | | | 1.82 | | | | | | | | | | | | | 132 | | | | | | | | | | | | | 116 | | | | | | | | | | | | | 5 | | | | | | | | | | | | | | 8 | | | | | | | | | | | | 8 | | | | | | | | | | | | | | | 0.0387 | | | | | | | | | |
| 6 | | | | | | | 4 | | | | | | | | | | | | 76447062 | | | | | | | | | | | | rs76333976 | | | | | | | | | | | | G | | | | | | | | | | | C | | | | | | | | THAP6 | | | | | | | | | | | | | D | | | | | | | | | | | 1.81 | | | | | | | | | | | | | 2.28 | | | | | | | | | | | | | 132 | | | | | | | | | | | | | 116 | | | | | | | | | | | | | 1 | | | | | | | | | | | | | | 2 | | | | | | | | | | | | 2 | | | | | | | | | | | | | | | 0.0129 | | | | | | | | | |
| 6 | | | | | | | | 4 | | | | | | | | | | | | 76581064 | | | | | | | | | | | | rs6823013 | | | | | | | | | | | | C | | | | | | | | T | | | | | | | | | G3BP2 | | | | | | | | | | | | | . | | | | | | | | | | 1.81 | | | | | | | | | | | | | | 1.71 | | | | | | | | | | | | | 132 | | | | | | | | | | | | | 116 | | | | | | | | | | | | | 2 | | | | | | | | | | | | | | 3 | | | | | | | | | | | | 3 | | | | | | | | | | | | | | | 0.0198 | | | | | | | | | | |
| 6 | | | | | | | | 5 | | | | | | | | | | | | 140182074 | | | | | | | | | | | | rs17844259 | | | | | | | | | | | | G | | | | | | | | A | | | | | | | | | PCDHA3 | | | | | | | | | | | | | D | | | | | | | | | | 3.62 | | | | | | | | | | | | | | 2.28 | | | | | | | | | | | | | 132 | | | | | | | | | | | | | 116 | | | | | | | | | | | | | 2 | | | | | | | | | | | | | | 4 | | | | | | | | | | | | 4 | | | | | | | | | | | | | | | 0.0179 | | | | | | | | | | |
| 6 | | | | | | | | 5 | | | | | | | | | | | | 26886200 | | | | | | | | | | | | rs41271091 | | | | | | | | | | | | C | | | | | | | | A | | | | | | | | | CDH9 | | | | | | | | | | | | | . | | | | | | | | | | 1.84 | | | | | | | | | | | | | | 1.87 | | | | | | | | | | | | | 128 | | | | | | | | | | | | | 114 | | | | | | | | | | | | | 12 | | | | | | | | | | | | | | 20 | | | | | | | | | | | | 20 | | | | | | | | | | | | | | | 0.0972 | | | | | | | | | | |
| 6 | | | | | | | | 5 | | | | | | | | | | | | 70898466 | | | | | | | | | | | | . | | | | | | | | | | | | T | | | | | | | | TC | | | | | | | | | MCCC2 | | | | | | | | | | | | | . | | | | | | | | | | 1.81 | | | | | | | | | | | | | | 1.71 | | | | | | | | | | | | | 264 | | | | | | | | | | | | | 232 | | | | | | | | | | | | | 2 | | | | | | | | | | | | | | 3 | | | | | | | | | | | | 3 | | | | | | | | | | | | | | | 0.0179 | | | | | | | | | | |
| group | | | | | | | | chr | | | | | | | | | | | | Pos | | | | | | | | | | | | id | | | | | | | | | | | | ref | | | | | | | | alt | | | | | | | | | gene | | | | | | | | | | | | | LR | | | | | | | | | | **Gene burden ratio** | | | | | | | | | | | | | | | | | | | | | | | | | | | **Total No. of alleles**  **in gene** | | | | | | | | | | | | | | | | | | | | | | | | | | **No. alt alleles**  **in genes** | | | | | | | | | | | | | | | | | | | | | | | | | | **No. of**  **cases with**  **alt alleles** | | | | | | | | | | | | | | | **Variant allele frequency** | | | | | | | | | | |
|  | | | | | | | |  | | | | | | | | | | | |  | | | | | | | | | | | |  | | | | | | | | | | | |  | | | | | | | |  | | | | | | | | |  | | | | | | | | | | | | |  | | | | | | | | | | KG  East Asia | | | | | | | | | | | | | | healthy  Ctrl | | | | | | | | | | | | | healthy  Ctrl | | | | | | | | | | | | | RA | | | | | | | | | | | | | healthy Ctrl | | | | | | | | | | | | | | RA | | | | | | | | | | | |  | | | | | | | | | | | | | | | KG  East Asia | | | | | | | | | | |
| 6 | | | | | | | | 5 | | | | | | | | | | | | 70922542 | | | | | | | | | | | | rs549784997 | | | | | | | | | | | | C | | | | | | | | T | | | | | | | | | MCCC2 | | | | | | | | | | | | | D | | | | | | | | | | 1.81 | | | | | | | | | | | | | | 1.71 | | | | | | | | | | | | | 264 | | | | | | | | | | | | | 232 | | | | | | | | | | | | | 2 | | | | | | | | | | | | | | 3 | | | | | | | | | | | | 3 | | | | | | | | | | | | | | | . | | | | | | | | | | |
| 6 | | | | | | | | 6 | | | | | | | | | | | | 109721228 | | | | | | | | | | | | rs4946972 | | | | | | | | | | | | A | | | | | | | | C | | | | | | | | | PPIL6 | | | | | | | | | | | | | . | | | | | | | | | | 1.93 | | | | | | | | | | | | | | 1.82 | | | | | | | | | | | | | 132 | | | | | | | | | | | | | 116 | | | | | | | | | | | | | 10 | | | | | | | | | | | | | | 16 | | | | | | | | | | | | 15 | | | | | | | | | | | | | | | 0.1032 | | | | | | | | | | |
| 6 | | | | | | | | 6 | | | | | | | | | | | | 109763947 | | | | | | | | | | | | rs35444917 | | | | | | | | | | | | TC | | | | | | | | T | | | | | | | | | SMPD2 | | | | | | | | | | | | | . | | | | | | | | | | 1.81 | | | | | | | | | | | | | | 1.82 | | | | | | | | | | | | | 132 | | | | | | | | | | | | | 116 | | | | | | | | | | | | | 10 | | | | | | | | | | | | | | 16 | | | | | | | | | | | | 15 | | | | | | | | | | | | | | | 0.1032 | | | | | | | | | | |
| 6 | | | | | | | | 6 | | | | | | | | | | | | 116783619 | | | | | | | | | | | | rs117361304 | | | | | | | | | | | | T | | | | | | | | G | | | | | | | | | FAM26F | | | | | | | | | | | | | . | | | | | | | | | | 2.63 | | | | | | | | | | | | | | 3.03 | | | | | | | | | | | | | 132 | | | | | | | | | | | | | 116 | | | | | | | | | | | | | 6 | | | | | | | | | | | | | | 16 | | | | | | | | | | | | 16 | | | | | | | | | | | | | | | 0.0635 | | | | | | | | | | |
| 6 | | | | | | | | 6 | | | | | | | | | | | | 136554647 | | | | | | | | | | | | rs2274141 | | | | | | | | | | | | A | | | | | | | | T | | | | | | | | | MTFR2 | | | | | | | | | | | | | . | | | | | | | | | | 2.02 | | | | | | | | | | | | | | 2.15 | | | | | | | | | | | | | 112 | | | | | | | | | | | | | 104 | | | | | | | | | | | | | 1 | | | | | | | | | | | | | | 2 | | | | | | | | | | | | 2 | | | | | | | | | | | | | | | 0.0407 | | | | | | | | | | |
| 6 | | | | | | | | 6 | | | | | | | | | | | | 167738715 | | | | | | | | | | | | rs12526096 | | | | | | | | | | | | G | | | | | | | | A | | | | | | | | | TTLL2 | | | | | | | | | | | | | . | | | | | | | | | | 3.62 | | | | | | | | | | | | | | 3.03 | | | | | | | | | | | | | 132 | | | | | | | | | | | | | 116 | | | | | | | | | | | | | 3 | | | | | | | | | | | | | | 8 | | | | | | | | | | | | 8 | | | | | | | | | | | | | | | 0.0347 | | | | | | | | | | |
| 6 | | | | | | | | 6 | | | | | | | | | | | | 25691362 | | | | | | | | | | | | rs17492659 | | | | | | | | | | | | C | | | | | | | | T | | | | | | | | | SCGN | | | | | | | | | | | | | . | | | | | | | | | | 1.81 | | | | | | | | | | | | | | 1.99 | | | | | | | | | | | | | 132 | | | | | | | | | | | | | 116 | | | | | | | | | | | | | 4 | | | | | | | | | | | | | | 7 | | | | | | | | | | | | 7 | | | | | | | | | | | | | | | 0.0417 | | | | | | | | | | |
| 6 | | | | | | | | 6 | | | | | | | | | | | | 26507069 | | | | | | | | | | | | rs188130447 | | | | | | | | | | | | T | | | | | | | | C | | | | | | | | | BTN1A1 | | | | | | | | | | | | | . | | | | | | | | | | 1.81 | | | | | | | | | | | | | | 4.55 | | | | | | | | | | | | | 264 | | | | | | | | | | | | | 232 | | | | | | | | | | | | | 1 | | | | | | | | | | | | | | 4 | | | | | | | | | | | | 4 | | | | | | | | | | | | | | | 0.0208 | | | | | | | | | | |
| 6 | | | | | | | | 6 | | | | | | | | | | | | 26509379 | | | | | | | | | | | | rs752379950 | | | | | | | | | | | | C | | | | | | | | T | | | | | | | | | BTN1A1 | | | | | | | | | | | | | . | | | | | | | | | | 1.81 | | | | | | | | | | | | | | 4.55 | | | | | | | | | | | | | 264 | | | | | | | | | | | | | 232 | | | | | | | | | | | | | 1 | | | | | | | | | | | | | | 4 | | | | | | | | | | | | 4 | | | | | | | | | | | | | | | . | | | | | | | | | | |
| 6 | | | | | | | | 6 | | | | | | | | | | | | 32361841 | | | | | | | | | | | | . | | | | | | | | | | | | T | | | | | | | | C | | | | | | | | | BTNL2 | | | | | | | | | | | | | . | | | | | | | | | | 1.85 | | | | | | | | | | | | | | 1.67 | | | | | | | | | | | | | 660 | | | | | | | | | | | | | 580 | | | | | | | | | | | | | 53 | | | | | | | | | | | | | | 78 | | | | | | | | | | | | 74 | | | | | | | | | | | | | | | 0.0417 | | | | | | | | | | |
| 6 | | | | | | | | 6 | | | | | | | | | | | | 32361842 | | | | | | | | | | | | . | | | | | | | | | | | | G | | | | | | | | A | | | | | | | | | BTNL2 | | | | | | | | | | | | | . | | | | | | | | | | 1.85 | | | | | | | | | | | | | | 1.67 | | | | | | | | | | | | | 660 | | | | | | | | | | | | | 580 | | | | | | | | | | | | | 53 | | | | | | | | | | | | | | 78 | | | | | | | | | | | | 74 | | | | | | | | | | | | | | | 0.0417 | | | | | | | | | | |
| 6 | | | | | | | | 6 | | | | | | | | | | | | 32369554 | | | | | | | | | | | | . | | | | | | | | | | | | G | | | | | | | | A | | | | | | | | | BTNL2 | | | | | | | | | | | | | . | | | | | | | | | | 1.85 | | | | | | | | | | | | | | 1.67 | | | | | | | | | | | | | 660 | | | | | | | | | | | | | 580 | | | | | | | | | | | | | 53 | | | | | | | | | | | | | | 78 | | | | | | | | | | | | 74 | | | | | | | | | | | | | | | 0.1171 | | | | | | | | | | |
| 6 | | | | | | | | 6 | | | | | | | | | | | | 32369586 | | | | | | | | | | | | . | | | | | | | | | | | | GAA | | | | | | | | G | | | | | | | | | BTNL2 | | | | | | | | | | | | | . | | | | | | | | | | 1.85 | | | | | | | | | | | | | | 1.67 | | | | | | | | | | | | | 660 | | | | | | | | | | | | | 580 | | | | | | | | | | | | | 53 | | | | | | | | | | | | | | 78 | | | | | | | | | | | | 74 | | | | | | | | | | | | | | | . | | | | | | | | | | |
| 6 | | | | | | | | 6 | | | | | | | | | | | | 32370969 | | | | | | | | | | | | . | | | | | | | | | | | | TG | | | | | | | | T | | | | | | | | | BTNL2 | | | | | | | | | | | | | . | | | | | | | | | | 1.85 | | | | | | | | | | | | | | 1.67 | | | | | | | | | | | | | 660 | | | | | | | | | | | | | 580 | | | | | | | | | | | | | 53 | | | | | | | | | | | | | | 78 | | | | | | | | | | | | 74 | | | | | | | | | | | | | | | 0.1984 | | | | | | | | | | |
| 6 | | | | | | | | 6 | | | | | | | | | | | | 36929653 | | | | | | | | | | | | rs144897670 | | | | | | | | | | | | C | | | | | | | | T | | | | | | | | | PI16 | | | | | | | | | | | | | . | | | | | | | | | | 1.81 | | | | | | | | | | | | | | 3.41 | | | | | | | | | | | | | 132 | | | | | | | | | | | | | 116 | | | | | | | | | | | | | 1 | | | | | | | | | | | | | | 3 | | | | | | | | | | | | 3 | | | | | | | | | | | | | | | 0.0129 | | | | | | | | | | |
| 6 | | | | | | | | 6 | | | | | | | | | | | | 39330207 | | | | | | | | | | | | rs9349115 | | | | | | | | | | | | C | | | | | | | | G | | | | | | | | | KIF6 | | | | | | | | | | | | | . | | | | | | | | | | 1.81 | | | | | | | | | | | | | | . | | | | | | | | | | | | | 132 | | | | | | | | | | | | | 116 | | | | | | | | | | | | | 0 | | | | | | | | | | | | | | 7 | | | | | | | | | | | | 7 | | | | | | | | | | | | | | | 0.0367 | | | | | | | | | | |
| 6 | | | | | | | | 6 | | | | | | | | | | | | 87994504 | | | | | | | | | | | | rs35259282 | | | | | | | | | | | | C | | | | | | | | T | | | | | | | | | GJB7 | | | | | | | | | | | | | D | | | | | | | | | | 1.81 | | | | | | | | | | | | | | 4.55 | | | | | | | | | | | | | 264 | | | | | | | | | | | | | 232 | | | | | | | | | | | | | 1 | | | | | | | | | | | | | | 4 | | | | | | | | | | | | 4 | | | | | | | | | | | | | | | 0.0188 | | | | | | | | | | |
| 6 | | | | | | | | 6 | | | | | | | | | | | | 87994537 | | | | | | | | | | | | rs112552839 | | | | | | | | | | | | G | | | | | | | | A | | | | | | | | | GJB7 | | | | | | | | | | | | | D | | | | | | | | | | 1.81 | | | | | | | | | | | | | | 4.55 | | | | | | | | | | | | | 264 | | | | | | | | | | | | | 232 | | | | | | | | | | | | | 1 | | | | | | | | | | | | | | 4 | | | | | | | | | | | | 4 | | | | | | | | | | | | | | | . | | | | | | | | | | |
| group | | | | | chr | | | | | | | | | | | | pos | | | | | | | | | | | | id | | | | | | | | | | | | ref | | | | | | | | | | | | | | | | | | | alt | | | | | gene | | | | | | | | | | | | | LR | | | | | | | | | | | **Gene burden ratio** | | | | | | | | | | | | | | | | | | | | | | | | | **Total No. of alleles**  **in gene** | | | | | | | | | | | | | | | | | | | | | | | | | | | **No. alt alleles**  **in genes** | | | | | | | | | | | | | | | | | | | | | | | | | | **No. of**  **cases with**  **alt alleles** | | | | | | | | | | | | | | **Variant allele frequency** | | | | | | | |
|  | | | | |  | | | | | | | | | | | |  | | | | | | | | | | | |  | | | | | | | | | | | |  | | | | | | | | | | | | | | | | | | |  | | | | |  | | | | | | | | | | | | |  | | | | | | | | | | | KG  East Asia | | | | | | | | | | | | healthy  Ctrl | | | | | | | | | | | | | healthy  Ctrl | | | | | | | | | | | | | RA | | | | | | | | | | | | | | healthy Ctrl | | | | | | | | | | | | | RA | | | | | | | | | | | | |  | | | | | | | | | | | | | | KG  East Asia | | | | | | | |
| 6 | | | | | 7 | | | | | | | | | | | | 123267310 | | | | | | | | | | | | rs116956332 | | | | | | | | | | | | C | | | | | | | | | | | | | | | | | | | T | | | | | ASB15 | | | | | | | | | | | | | . | | | | | | | | | | | 3.62 | | | | | | | | | | | | 2.28 | | | | | | | | | | | | | 132 | | | | | | | | | | | | | 116 | | | | | | | | | | | | | | 1 | | | | | | | | | | | | | 2 | | | | | | | | | | | | | 2 | | | | | | | | | | | | | | 0.0208 | | | | | | | |
| 6 | | | | | 7 | | | | | | | | | | | | 12727793 | | | | | | | | | | | | rs117537847 | | | | | | | | | | | | G | | | | | | | | | | | | | | | | | | | A | | | | | ARL4A | | | | | | | | | | | | | . | | | | | | | | | | | 1.61 | | | | | | | | | | | | 2.03 | | | | | | | | | | | | | 132 | | | | | | | | | | | | | 114 | | | | | | | | | | | | | | 4 | | | | | | | | | | | | | 7 | | | | | | | | | | | | | 7 | | | | | | | | | | | | | | 0.0466 | | | | | | | |
| 6 | | | | | 7 | | | | | | | | | | | | 40277228 | | | | | | | | | | | | rs76667176 | | | | | | | | | | | | T | | | | | | | | | | | | | | | | | | | C | | | | | SUGCT | | | | | | | | | | | | | . | | | | | | | | | | | 1.51 | | | | | | | | | | | | . | | | | | | | | | | | | | 396 | | | | | | | | | | | | | 348 | | | | | | | | | | | | | | 0 | | | | | | | | | | | | | 5 | | | | | | | | | | | | | 5 | | | | | | | | | | | | | | 0.0119 | | | | | | | |
| 6 | | | | | 7 | | | | | | | | | | | | 40899963 | | | | | | | | | | | | rs751805172 | | | | | | | | | | | | C | | | | | | | | | | | | | | | | | | | G | | | | | SUGCT | | | | | | | | | | | | | D | | | | | | | | | | | 1.51 | | | | | | | | | | | | . | | | | | | | | | | | | | 396 | | | | | | | | | | | | | 348 | | | | | | | | | | | | | | 0 | | | | | | | | | | | | | 5 | | | | | | | | | | | | | 5 | | | | | | | | | | | | | | . | | | | | | | |
| 6 | | | | | 7 | | | | | | | | | | | | 40899965 | | | | | | | | | | | | rs767692645 | | | | | | | | | | | | C | | | | | | | | | | | | | | | | | | | G | | | | | SUGCT | | | | | | | | | | | | | D | | | | | | | | | | | 1.51 | | | | | | | | | | | | . | | | | | | | | | | | | | 396 | | | | | | | | | | | | | 348 | | | | | | | | | | | | | | 0 | | | | | | | | | | | | | 5 | | | | | | | | | | | | | 5 | | | | | | | | | | | | | | . | | | | | | | |
| 6 | | | | | 7 | | | | | | | | | | | | 72984917 | | | | | | | | | | | | . | | | | | | | | | | | | CGTT | | | | | | | | | | | | | | | | | | | C | | | | | TBL2 | | | | | | | | | | | | | . | | | | | | | | | | | 1.81 | | | | | | | | | | | | 2.84 | | | | | | | | | | | | | 132 | | | | | | | | | | | | | 116 | | | | | | | | | | | | | | 2 | | | | | | | | | | | | | 5 | | | | | | | | | | | | | 5 | | | | | | | | | | | | | | 0.0159 | | | | | | | |
| 6 | | | | | 7 | | | | | | | | | | | | 73083744 | | | | | | | | | | | | . | | | | | | | | | | | | C | | | | | | | | | | | | | | | | | | | T | | | | | VPS37D | | | | | | | | | | | | | . | | | | | | | | | | | 5.43 | | | | | | | | | | | | 1.71 | | | | | | | | | | | | | 132 | | | | | | | | | | | | | 116 | | | | | | | | | | | | | | 2 | | | | | | | | | | | | | 3 | | | | | | | | | | | | | 3 | | | | | | | | | | | | | | 0.0129 | | | | | | | |
| 6 | | | | | 7 | | | | | | | | | | | | 73630406 | | | | | | | | | | | | . | | | | | | | | | | | | G | | | | | | | | | | | | | | | | | | | A | | | | | LAT2 | | | | | | | | | | | | | . | | | | | | | | | | | 1.97 | | | | | | | | | | | | 2.28 | | | | | | | | | | | | | 132 | | | | | | | | | | | | | 116 | | | | | | | | | | | | | | 6 | | | | | | | | | | | | | 12 | | | | | | | | | | | | | 11 | | | | | | | | | | | | | | 0.0446 | | | | | | | |
| 6 | | | | | 7 | | | | | | | | | | | | 7476098 | | | | | | | | | | | | rs11984435 | | | | | | | | | | | | T | | | | | | | | | | | | | | | | | | | C | | | | | COL28A1 | | | | | | | | | | | | | . | | | | | | | | | | | 2.41 | | | | | | | | | | | | 2.28 | | | | | | | | | | | | | 396 | | | | | | | | | | | | | 348 | | | | | | | | | | | | | | 4 | | | | | | | | | | | | | 8 | | | | | | | | | | | | | 8 | | | | | | | | | | | | | | 0.0208 | | | | | | | |
| 6 | | | | | 7 | | | | | | | | | | | | 7491996 | | | | | | | | | | | | rs148703211 | | | | | | | | | | | | G | | | | | | | | | | | | | | | | | | | C | | | | | COL28A1 | | | | | | | | | | | | | D | | | | | | | | | | | 2.41 | | | | | | | | | | | | 2.28 | | | | | | | | | | | | | 396 | | | | | | | | | | | | | 348 | | | | | | | | | | | | | | 4 | | | | | | | | | | | | | 8 | | | | | | | | | | | | | 8 | | | | | | | | | | | | | | 0.0109 | | | | | | | |
| 6 | | | | | 7 | | | | | | | | | | | | 7559695 | | | | | | | | | | | | . | | | | | | | | | | | | G | | | | | | | | | | | | | | | | | | | C | | | | | COL28A1 | | | | | | | | | | | | | D | | | | | | | | | | | 2.41 | | | | | | | | | | | | 2.28 | | | | | | | | | | | | | 396 | | | | | | | | | | | | | 348 | | | | | | | | | | | | | | 4 | | | | | | | | | | | | | 8 | | | | | | | | | | | | | 8 | | | | | | | | | | | | | | . | | | | | | | |
| 6 | | | | | 8 | | | | | | | | | | | | 10677699 | | | | | | | | | | | | rs61757720 | | | | | | | | | | | | A | | | | | | | | | | | | | | | | | | | T | | | | | PINX1 | | | | | | | | | | | | | . | | | | | | | | | | | 2.74 | | | | | | | | | | | | 1.72 | | | | | | | | | | | | | 264 | | | | | | | | | | | | | 230 | | | | | | | | | | | | | | 2 | | | | | | | | | | | | | 3 | | | | | | | | | | | | | 3 | | | | | | | | | | | | | | 0.0139 | | | | | | | |
| 6 | | | | | 8 | | | | | | | | | | | | 10692283 | | | | | | | | | | | | rs746583094 | | | | | | | | | | | | G | | | | | | | | | | | | | | | | | | | A | | | | | PINX1 | | | | | | | | | | | | | D | | | | | | | | | | | 2.74 | | | | | | | | | | | | 1.72 | | | | | | | | | | | | | 264 | | | | | | | | | | | | | 230 | | | | | | | | | | | | | | 2 | | | | | | | | | | | | | 3 | | | | | | | | | | | | | 3 | | | | | | | | | | | | | | . | | | | | | | |
| 6 | | | | | 8 | | | | | | | | | | | | 116599274 | | | | | | | | | | | | rs745384526 | | | | | | | | | | | | G | | | | | | | | | | | | | | | | | | | A | | | | | TRPS1 | | | | | | | | | | | | | D | | | | | | | | | | | 5.43 | | | | | | | | | | | | 2.28 | | | | | | | | | | | | | 264 | | | | | | | | | | | | | 232 | | | | | | | | | | | | | | 1 | | | | | | | | | | | | | 2 | | | | | | | | | | | | | 2 | | | | | | | | | | | | | | . | | | | | | | |
| 6 | | | | | 8 | | | | | | | | | | | | 116599415 | | | | | | | | | | | | . | | | | | | | | | | | | T | | | | | | | | | | | | | | | | | | | C | | | | | TRPS1 | | | | | | | | | | | | | D | | | | | | | | | | | 5.43 | | | | | | | | | | | | 2.28 | | | | | | | | | | | | | 264 | | | | | | | | | | | | | 232 | | | | | | | | | | | | | | 1 | | | | | | | | | | | | | 2 | | | | | | | | | | | | | 2 | | | | | | | | | | | | | | . | | | | | | | |
| 6 | | | | | 8 | | | | | | | | | | | | 30697414 | | | | | | | | | | | | rs149889016 | | | | | | | | | | | | A | | | | | | | | | | | | | | | | | | | G | | | | | TEX15 | | | | | | | | | | | | | . | | | | | | | | | | | 3.62 | | | | | | | | | | | | 2.24 | | | | | | | | | | | | | 130 | | | | | | | | | | | | | 116 | | | | | | | | | | | | | | 1 | | | | | | | | | | | | | 2 | | | | | | | | | | | | | 2 | | | | | | | | | | | | | | 0.0188 | | | | | | | |
| 6 | | | | | 9 | | | | | | | | | | | | 137998709 | | | | | | | | | | | | rs35408956 | | | | | | | | | | | | T | | | | | | | | | | | | | | | | | | | A | | | | | OLFM1 | | | | | | | | | | | | | . | | | | | | | | | | | 2.07 | | | | | | | | | | | | 2.28 | | | | | | | | | | | | | 132 | | | | | | | | | | | | | 116 | | | | | | | | | | | | | | 4 | | | | | | | | | | | | | 8 | | | | | | | | | | | | | 8 | | | | | | | | | | | | | | 0.0367 | | | | | | | |
| 6 | | | | | 9 | | | | | | | | | | | | 140248783 | | | | | | | | | | | | rs372878424 | | | | | | | | | | | | G | | | | | | | | | | | | | | | | | | | A | | | | | EXD3 | | | | | | | | | | | | | . | | | | | | | | | | | 1.81 | | | | | | | | | | | | 2.28 | | | | | | | | | | | | | 264 | | | | | | | | | | | | | 232 | | | | | | | | | | | | | | 1 | | | | | | | | | | | | | 2 | | | | | | | | | | | | | 2 | | | | | | | | | | | | | | . | | | | | | | |
| group | | | | | chr | | | | | | | | | | | | pos | | | | | | | | | | | | id | | | | | | | | | | | | ref | | | | | | | | | | | | | | | | | | | alt | | | | | gene | | | | | | | | | | | | | LR | | | | | | | | | | | **Gene burden ratio** | | | | | | | | | | | | | | | | | | | | | | | | | **Total No. of alleles**  **in gene** | | | | | | | | | | | | | | | | | | | | | | | | | | | **No. alt alleles**  **in genes** | | | | | | | | | | | | | | | | | | | | | | | | | | **No. of**  **cases with**  **alt alleles** | | | | | | | | | | | | | | **Variant allele frequency** | | | | | | | |
|  | | | | |  | | | | | | | | | | | |  | | | | | | | | | | | |  | | | | | | | | | | | |  | | | | | | | | | | | | | | | | | | |  | | | | |  | | | | | | | | | | | | |  | | | | | | | | | | | KG  East Asia | | | | | | | | | | | | healthy  Ctrl | | | | | | | | | | | | | healthy  Ctrl | | | | | | | | | | | | | RA | | | | | | | | | | | | | | healthy Ctrl | | | | | | | | | | | | | RA | | | | | | | | | | | | |  | | | | | | | | | | | | | | KG  East Asia | | | | | | | |
| 6 | | | | | 9 | | | | | | | | | | | | 140249147 | | | | | | | | | | | | rs143654067 | | | | | | | | | | | | C | | | | | | | | | | | | | | | | | | | T | | | | | EXD3 | | | | | | | | | | | | | . | | | | | | | | | | | 1.81 | | | | | | | | | | | | 2.28 | | | | | | | | | | | | | 264 | | | | | | | | | | | | | 232 | | | | | | | | | | | | | | 1 | | | | | | | | | | | | | 2 | | | | | | | | | | | | | 2 | | | | | | | | | | | | | | 0.0119 | | | | | | | |
| 6 | | | | | 9 | | | | | | | | | | | | 5892552 | | | | | | | | | | | | rs148372841 | | | | | | | | | | | | G | | | | | | | | | | | | | | | | | | | C | | | | | MLANA | | | | | | | | | | | | | . | | | | | | | | | | | 4.53 | | | | | | | | | | | | 5.69 | | | | | | | | | | | | | 132 | | | | | | | | | | | | | 116 | | | | | | | | | | | | | | 1 | | | | | | | | | | | | | 5 | | | | | | | | | | | | | 5 | | | | | | | | | | | | | | 0.0139 | | | | | | | |
| 6 | | | | | X | | | | | | | | | | | | 2871176 | | | | | | | | | | | | rs56393981 | | | | | | | | | | | | G | | | | | | | | | | | | | | | | | | | A | | | | | ARSE | | | | | | | | | | | | | . | | | | | | | | | | | 2.74 | | | | | | | | | | | | 3.41 | | | | | | | | | | | | | 132 | | | | | | | | | | | | | 116 | | | | | | | | | | | | | | 2 | | | | | | | | | | | | | 6 | | | | | | | | | | | | | 5 | | | | | | | | | | | | | | 0.0262 | | | | | | | |
| 6 | | | | | X | | | | | | | | | | | | 49114808 | | | | | | | | | | | | . | | | | | | | | | | | | C | | | | | | | | | | | | | | | | | | | A | | | | | FOXP3 | | | | | | | | | | | | | D | | | | | | | | | | | 2.06 | | | | | | | | | | | | 5.12 | | | | | | | | | | | | | 132 | | | | | | | | | | | | | 116 | | | | | | | | | | | | | | 2 | | | | | | | | | | | | | 9 | | | | | | | | | | | | | 8 | | | | | | | | | | | | | | 0.0393 | | | | | | | |
| 6 | | | | | X | | | | | | | | | | | | 8763309 | | | | | | | | | | | | . | | | | | | | | | | | | GCTGCTGCTG  CTGCGGCTT | | | | | | | | | | | | | | | | | | | * | | | | | FAM9A | | | | | | | | | | | | | . | | | | | | | | | | | 3.69 | | | | | | | | | | | | . | | | | | | | | | | | | | 130 | | | | | | | | | | | | | 114 | | | | | | | | | | | | | | 0 | | | | | | | | | | | | | 4 | | | | | | | | | | | | | 4 | | | | | | | | | | | | | | . | | | | | | | |
