# Supplementary material for: Identification of potential genetic causal variants for rheumatoid arthritis by whole-exome sequencing
Source: Oncotarget. 2017 Nov 22;8(67):111119–29. doi: 10.18632/oncotarget.22630 (PMC5762310; doi:10.18632/oncotarget.22630)
Supplement: Supplementary file 4 [file oncotarget-08-111119-s004.docx]

**Supplementary Table 4:** **Candidate variant list from RA disease duration comparison.**

| group | chr | pos | id | ref | alt | gene | LR | **Gene burden ratio** | | **Total No. of alleles**  **in gene** | | **No. alt alleles**  **in genes** | | **No. of ≥3-Year**  **cases with**  **alt alleles** | **Variant allele frequency** |
| --- | --- | --- | --- | --- | --- | --- | --- | --- | --- | --- | --- | --- | --- | --- | --- |
|  |  |  |  |  |  |  |  | KG  East Asia | ≤1-Year | ≤1-Year | ≥3-Year | ≤1-Year | ≥3-Year |  | KG  East Asia |
| 2 | 10 | 101829514 | rs61751507 | C | T | CPN1 | T | 2.28 | 2.26 | 52 | 46 | 1 | 2 | 2 | 0.0248 |
| 2 | 1 | 115220086 | rs121912682 | C | T | AMPD1 | D | 4.57 | . | 52 | 46 | 0 | 3 | 3 | 0.0109 |
| 2 | 11 | 18291302 | rs79681911 | G | A | SAA1 | T | 3.04 | 2.26 | 52 | 46 | 1 | 2 | 2 | 0.0198 |
| 2 | 5 | 149212243 | rs7732671 | G | C | PPARGC1B | T | 1.76 | 1.88 | 52 | 46 | 3 | 5 | 5 | 0.0625 |
| 2 | 7 | 138417791 | rs3807153 | A | G | ATP6V0A4 | T | 1.71 | . | 52 | 46 | 0 | 3 | 3 | 0.0516 |
| 2 | X | 38229135 | rs72554348 | G | C | OTC | . | 4.61 | 4.35 | 50 | 46 | 1 | 4 | 3 | 0.0144 |
| 4 | 10 | 135086331 | rs536126291 | C | T | ADAM8 | . | 9.13 | 2.26 | 104 | 92 | 1 | 2 | 2 | 0.002 |
| 4 | 10 | 135087305 | rs3810960 | G | A | ADAM8 | . | 9.13 | 2.26 | 104 | 92 | 1 | 2 | 2 | 0.003 |
| 4 | 10 | 25144247 | rs199794379 | A | G | PRTFDC1 | D | 9.13 | 2.26 | 104 | 92 | 1 | 2 | 2 | 0.004 |
| 4 | 10 | 25147326 | rs199983667 | C | A | PRTFDC1 | D | 9.13 | 2.26 | 104 | 92 | 1 | 2 | 2 | 0.001 |
| 4 | 10 | 70987060 | rs10823320 | A | G | HKDC1 | D | 18.26 | . | 208 | 184 | 0 | 4 | 4 | 0.001 |
| 4 | 10 | 70992606 | rs575180113 | G | A | HKDC1 | D | 18.26 | . | 208 | 184 | 0 | 4 | 4 | 0.003 |
| 4 | 10 | 71002935 | rs185650169 | C | T | HKDC1 | . | 18.26 | . | 208 | 184 | 0 | 4 | 4 | 0.0069 |
| 4 | 10 | 71021004 | rs143285779 | C | T | HKDC1 | D | 18.26 | . | 208 | 184 | 0 | 4 | 4 | 0.001 |
| 4 | 1 | 100174455 | rs192583899 | T | C | FRRS1 | . | 3.04 | 2.26 | 104 | 92 | 1 | 2 | 2 | 0.003 |
| 4 | 1 | 100177969 | rs187278122 | A | G | FRRS1 | . | 3.04 | 2.26 | 104 | 92 | 1 | 2 | 2 | 0.006 |
| 4 | 11 | 130060344 | rs199819888 | C | T | ST14 | . | 1.52 | 2.26 | 156 | 138 | 1 | 2 | 2 | 0.003 |
| 4 | 11 | 130064039 | rs76687780 | C | G | ST14 | . | 1.52 | 2.26 | 156 | 138 | 1 | 2 | 2 | 0.006 |
| 4 | 1 | 11826069 | rs560354825 | G | C | C1orf167 | D | 6.09 | 1.51 | 156 | 138 | 3 | 4 | 4 | 0.002 |
| 4 | 1 | 11826663 | rs374366683 | G | A | C1orf167 | . | 6.09 | 1.51 | 156 | 138 | 3 | 4 | 4 | 0.004 |
| 4 | 1 | 11844289 | rs76627351 | C | T | C1orf167 | . | 6.09 | 1.51 | 156 | 138 | 3 | 4 | 4 | 0.0079 |
| 4 | 1 | 146759364 | . | C | T | CHD1L | . | 13.7 | . | 48 | 46 | 0 | 3 | 3 | 0.001 |
| 4 | 11 | 74716666 | rs202090872 | G | C | NEU3 | D | 9.13 | 2.26 | 156 | 138 | 1 | 2 | 2 | 0.003 |
| 4 | 11 | 74716935 | rs200629627 | G | A | NEU3 | D | 9.13 | 2.26 | 156 | 138 | 1 | 2 | 2 | 0.003 |
| 4 | 11 | 74717001 | rs539514716 | C | T | NEU3 | D | 9.13 | 2.26 | 156 | 138 | 1 | 2 | 2 | 0.002 |
| 4 | 1 | 176853472 | rs79630456 | C | T | ASTN1 | . | 4.57 | 2.26 | 52 | 46 | 1 | 2 | 2 | 0.0089 |
| 4 | 1 | 19566783 | rs201918168 | C | T | EMC1 | . | 3.04 | 2.26 | 52 | 46 | 1 | 2 | 2 | 0.0079 |
| 4 | 1 | 197070906 | rs118010078 | C | T | ASPM | D | 6.85 | 3.39 | 104 | 92 | 1 | 3 | 3 | 0.0069 |
| 4 | 1 | 197072871 | rs144969324 | C | T | ASPM | D | 6.85 | 3.39 | 104 | 92 | 1 | 3 | 3 | 0.005 |
| 4 | 12 | 21011481 | rs145334570 | C | A | SLCO1B3 | D | 6.85 | . | 52 | 46 | 0 | 3 | 3 | 0.0099 |

| group | chr | pos | id | ref | alt | gene | LR | **Gene burden ratio** | | **Total No. of alleles**  **in gene** | | **No. alt alleles**  **in genes** | | **No. of ≥3-Year**  **cases with**  **alt alleles** | **Variant allele frequency** |
| --- | --- | --- | --- | --- | --- | --- | --- | --- | --- | --- | --- | --- | --- | --- | --- |
|  |  |  |  |  |  |  |  | KG  East Asia | ≤1-Year | ≤1-Year | ≥3-Year | ≤1-Year | ≥3-Year |  | KG  East Asia |
| 4 | 1 | 36056256 | rs114404250 | G | A | TFAP2E | D | 6.85 | . | 52 | 46 | 0 | 3 | 3 | 0.0069 |
| 4 | 14 | 21793077 | rs543867152 | C | T | RPGRIP1 | D | 13.7 | 3.39 | 104 | 92 | 1 | 3 | 3 | 0.001 |
| 4 | 14 | 21793236 | rs7157052 | G | A | RPGRIP1 | . | 13.7 | 3.39 | 104 | 92 | 1 | 3 | 3 | 0.002 |
| 4 | 14 | 94703898 | rs148831396 | G | T | PPP4R4 | . | 9.13 | 2.26 | 52 | 46 | 1 | 2 | 2 | 0.005 |
| 4 | 15 | 39876498 | rs200938835 | T | C | THBS1 | . | 4.57 | 2.26 | 156 | 138 | 1 | 2 | 2 | 0.002 |
| 4 | 15 | 39881204 | rs200366954 | A | G | THBS1 | D | 4.57 | 2.26 | 156 | 138 | 1 | 2 | 2 | 0.001 |
| 4 | 15 | 39886402 | rs185847032 | G | A | THBS1 | . | 4.57 | 2.26 | 156 | 138 | 1 | 2 | 2 | 0.002 |
| 4 | 15 | 48512855 | rs116848967 | G | A | SLC12A1 | D | 9.13 | 2.26 | 104 | 92 | 1 | 2 | 2 | 0.003 |
| 4 | 15 | 48566800 | rs201516084 | T | C | SLC12A1 | D | 9.13 | 2.26 | 104 | 92 | 1 | 2 | 2 | 0.005 |
| 4 | 15 | 79298783 | rs182075492 | G | C | RASGRF1 | . | 3.04 | 2.26 | 52 | 46 | 1 | 2 | 2 | 0.005 |
| 4 | 16 | 27492392 | rs200888316 | C | T | GTF3C1 | . | 9.13 | 2.26 | 104 | 92 | 1 | 2 | 2 | 0.001 |
| 4 | 16 | 27494449 | rs536534746 | G | A | GTF3C1 | . | 9.13 | 2.26 | 104 | 92 | 1 | 2 | 2 | 0.003 |
| 4 | 16 | 57935248 | rs374813501 | C | G | CNGB1 | . | 6.85 | 3.39 | 208 | 184 | 1 | 3 | 3 | 0.0069 |
| 4 | 16 | 57984441 | rs146170855 | C | T | CNGB1 | D | 6.85 | 3.39 | 208 | 184 | 1 | 3 | 3 | 0.002 |
| 4 | 16 | 57993840 | rs201703193 | C | T | CNGB1 | D | 6.85 | 3.39 | 208 | 184 | 1 | 3 | 3 | 0.001 |
| 4 | 16 | 57996967 | rs570828500 | G | A | CNGB1 | D | 6.85 | 3.39 | 208 | 184 | 1 | 3 | 3 | 0.003 |
| 4 | 1 | 6638781 | rs201116489 | C | T | TAS1R1 | D | 4.57 | 2.26 | 104 | 92 | 1 | 2 | 2 | 0.003 |
| 4 | 1 | 6638995 | rs150612979 | C | T | TAS1R1 | D | 4.57 | 2.26 | 104 | 92 | 1 | 2 | 2 | 0.006 |
| 4 | 16 | 70916556 | rs9932260 | G | A | HYDIN | . | 18.26 | 1.51 | 52 | 46 | 3 | 4 | 4 | 0.002 |
| 4 | 17 | 10398298 | rs140873918 | G | T | MYH1 | D | 3.04 | 2.26 | 416 | 368 | 2 | 4 | 4 | 0.002 |
| 4 | 17 | 10401217 | rs148588034 | C | T | MYH1 | D | 3.04 | 2.26 | 416 | 368 | 2 | 4 | 4 | 0.0069 |
| 4 | 17 | 10402103 | rs3744564 | G | A | MYH1 | D | 3.04 | 2.26 | 416 | 368 | 2 | 4 | 4 | 0.003 |
| 4 | 17 | 10408323 | rs534110923 | G | C | MYH1 | D | 3.04 | 2.26 | 416 | 368 | 2 | 4 | 4 | 0.005 |
| 4 | 17 | 10408380 | rs191339081 | T | A | MYH1 | D | 3.04 | 2.26 | 416 | 368 | 2 | 4 | 4 | 0.005 |
| 4 | 17 | 10412897 | rs534998190 | C | T | MYH1 | D | 3.04 | 2.26 | 416 | 368 | 2 | 4 | 4 | 0.002 |
| 4 | 17 | 10417137 | rs141592934 | C | T | MYH1 | . | 3.04 | 2.26 | 416 | 368 | 2 | 4 | 4 | 0.0069 |
| 4 | 17 | 10419751 | rs535620022 | C | T | MYH1 | . | 3.04 | 2.26 | 416 | 368 | 2 | 4 | 4 | 0.001 |
| 4 | 17 | 10541353 | rs201166774 | G | A | MYH3 | . | 2.28 | 2.26 | 104 | 92 | 1 | 2 | 2 | 0.006 |
| 4 | 17 | 10558169 | rs374786690 | G | C | MYH3 | . | 2.28 | 2.26 | 104 | 92 | 1 | 2 | 2 | 0.002 |
| 4 | 17 | 26856125 | rs188424977 | G | A | FOXN1 | D | 3.04 | 2.26 | 156 | 138 | 1 | 2 | 2 | 0.002 |

| group | chr | pos | id | ref | alt | gene | LR | **Gene burden ratio** | | **Total No. of alleles**  **in gene** | | **No. alt alleles**  **in genes** | | **No. of ≥3-Year**  **cases with**  **alt alleles** | | **Variant allele frequency** | |
| --- | --- | --- | --- | --- | --- | --- | --- | --- | --- | --- | --- | --- | --- | --- | --- | --- | --- |
|  |  |  |  |  |  |  |  | KG  East Asia | ≤1-Year | ≤1-Year | ≥3-Year | ≤1-Year | ≥3-Year | |  | | KG  East Asia |
| 4 | 17 | 26861343 | rs200401045 | C | T | FOXN1 | . | 3.04 | 2.26 | 156 | 138 | 1 | 2 | | 2 | | 0.001 |
| 4 | 17 | 26864171 | rs187814037 | C | T | FOXN1 | D | 3.04 | 2.26 | 156 | 138 | 1 | 2 | | 2 | | 0.005 |
| 4 | 17 | 46878711 | rs184362955 | G | A | TTLL6 | D | 13.7 | . | 52 | 46 | 0 | 3 | | 3 | | 0.001 |
| 4 | 17 | 48245315 | rs186669379 | C | T | SGCA | D | 1.83 | 2.26 | 156 | 138 | 1 | 2 | | 2 | | 0.006 |
| 4 | 17 | 48245924 | rs200945974 | G | A | SGCA | D | 1.83 | 2.26 | 156 | 138 | 1 | 2 | | 2 | | 0.001 |
| 4 | 17 | 48246530 | rs138254713 | G | A | SGCA | D | 1.83 | 2.26 | 156 | 138 | 1 | 2 | | 2 | | 0.0079 |
| 4 | 17 | 67079395 | rs117323775 | G | T | ABCA6 | D | 9.13 | 2.26 | 104 | 92 | 1 | 2 | | 2 | | 0.001 |
| 4 | 17 | 67121109 | rs200376492 | A | G | ABCA6 | D | 9.13 | 2.26 | 104 | 92 | 1 | 2 | | 2 | | 0.0079 |
| 4 | 17 | 73827216 | rs140184929 | C | T | UNC13D | D | 9.13 | 2.26 | 104 | 92 | 1 | 2 | | 2 | | 0.006 |
| 4 | 17 | 73839609 | rs527842266 | C | G | UNC13D | . | 9.13 | 2.26 | 104 | 92 | 1 | 2 | | 2 | | 0.002 |
| 4 | 18 | 72179676 | rs201407255 | C | T | CNDP2 | . | 4.57 | 2.26 | 104 | 92 | 1 | 2 | | 2 | | 0.003 |
| 4 | 18 | 72185883 | rs201217537 | G | A | CNDP2 | . | 4.57 | 2.26 | 104 | 92 | 1 | 2 | | 2 | | 0.0069 |
| 4 | 19 | 38934191 | rs192495718 | C | G | RYR1 | . | 1.96 | . | 364 | 322 | 0 | 3 | | 3 | | 0.004 |
| 4 | 19 | 38948941 | rs573737900 | C | T | RYR1 | . | 1.96 | . | 364 | 322 | 0 | 3 | | 3 | | 0.001 |
| 4 | 19 | 38964364 | rs551509462 | G | C | RYR1 | D | 1.96 | . | 364 | 322 | 0 | 3 | | 3 | | 0.004 |
| 4 | 19 | 38981375 | rs78851466 | A | G | RYR1 | . | 1.96 | . | 364 | 322 | 0 | 3 | | 3 | | 0.0089 |
| 4 | 19 | 39014545 | rs200939091 | G | A | RYR1 | . | 1.96 | . | 364 | 322 | 0 | 3 | | 3 | | 0.002 |
| 4 | 19 | 39014556 | rs370630840 | C | T | RYR1 | D | 1.96 | . | 364 | 322 | 0 | 3 | | 3 | | 0.001 |
| 4 | 19 | 39018329 | rs538497899 | C | T | RYR1 | D | 1.96 | . | 364 | 322 | 0 | 3 | | 3 | | 0.001 |
| 4 | 19 | 8140232 | rs145316149 | G | A | FBN3 | D | 3.04 | 2.26 | 208 | 184 | 1 | 2 | | 2 | | 0.004 |
| 4 | 19 | 8150331 | rs142940013 | G | A | FBN3 | D | 3.04 | 2.26 | 208 | 184 | 1 | 2 | | 2 | | 0.004 |
| 4 | 19 | 8155130 | rs183278638 | G | A | FBN3 | D | 3.04 | 2.26 | 208 | 184 | 1 | 2 | | 2 | | 0.002 |
| 4 | 19 | 8188820 | rs145435433 | C | T | FBN3 | D | 3.04 | 2.26 | 208 | 184 | 1 | 2 | | 2 | | 0.006 |
| 4 | 19 | 8979212 | rs149481309 | C | T | MUC16 | . | 5.71 | 2.83 | 208 | 184 | 2 | 5 | | 5 | | 0.0089 |
| 4 | 19 | 9002496 | rs553074376 | C | T | MUC16 | . | 5.71 | 2.83 | 208 | 184 | 2 | 5 | | 5 | | 0.006 |
| 4 | 19 | 9043416 | rs17417801 | G | A | MUC16 | . | 5.71 | 2.83 | 208 | 184 | 2 | 5 | | 5 | | 0.0079 |
| 4 | 19 | 9056878 | rs200934751 | GAGA | G | MUC16 | . | 5.71 | 2.83 | 208 | 184 | 2 | 5 | | 5 | | 0.0069 |
| 4 | 20 | 21142998 | rs191064527 | G | A | KIZ | . | 1.96 | . | 156 | 138 | 0 | 3 | | 3 | | 0.004 |
| 4 | 20 | 21143067 | rs116937124 | T | C | KIZ | . | 1.96 | . | 156 | 138 | 0 | 3 | | 3 | | 0.006 |
| 4 | 20 | 39788407 | rs201733074 | T | C | PLCG1 | . | 3.04 | 4.52 | 156 | 138 | 1 | 4 | | 4 | | 0.0079 |

| group | chr | pos | id | ref | alt | gene | LR | **Gene burden ratio** | | **Total No. of alleles**  **in gene** | | **No. alt alleles**  **in genes** | | **No. of ≥3-Year**  **cases with**  **alt alleles** | **Variant allele frequency** |
| --- | --- | --- | --- | --- | --- | --- | --- | --- | --- | --- | --- | --- | --- | --- | --- |
|  |  |  |  |  |  |  |  | KG  East Asia | ≤1-Year | ≤1-Year | ≥3-Year | ≤1-Year | ≥3-Year |  | KG  East Asia |
| 4 | 20 | 39797820 | rs547025579 | GACCAGAACC | G | PLCG1 | . | 3.04 | 4.52 | 156 | 138 | 1 | 4 | 4 | 0.0069 |
| 4 | 20 | 39798092 | rs183538599 | C | T | PLCG1 | . | 3.04 | 4.52 | 156 | 138 | 1 | 4 | 4 | 0.0079 |
| 4 | 21 | 10908822 | rs546417233 | T | C | TPTE | . | 9.13 | 2.26 | 208 | 184 | 1 | 2 | 2 | 0.002 |
| 4 | 21 | 10908886 | rs532224827 | T | C | TPTE | . | 9.13 | 2.26 | 208 | 184 | 1 | 2 | 2 | 0.002 |
| 4 | 21 | 10920159 | rs557556075 | G | T | TPTE | . | 9.13 | 2.26 | 208 | 184 | 1 | 2 | 2 | 0.002 |
| 4 | 21 | 10970067 | rs547492558 | T | C | TPTE | . | 9.13 | 2.26 | 208 | 184 | 1 | 2 | 2 | 0.003 |
| 4 | 21 | 43621840 | rs564785493 | T | A | ABCG1 | . | 9.13 | 2.26 | 104 | 92 | 1 | 2 | 2 | 0.001 |
| 4 | 21 | 43636306 | rs149713099 | C | T | ABCG1 | . | 9.13 | 2.26 | 104 | 92 | 1 | 2 | 2 | 0.0079 |
| 4 | 2 | 170038100 | rs140572511 | G | A | LRP2 | D | 11.41 | . | 260 | 230 | 0 | 5 | 5 | 0.002 |
| 4 | 2 | 170038761 | rs3213760 | C | T | LRP2 | D | 11.41 | . | 260 | 230 | 0 | 5 | 5 | 0.004 |
| 4 | 2 | 170042008 | rs563916043 | C | T | LRP2 | . | 11.41 | . | 260 | 230 | 0 | 5 | 5 | 0.001 |
| 4 | 2 | 170058290 | rs138382534 | C | T | LRP2 | D | 11.41 | . | 260 | 230 | 0 | 5 | 5 | 0.003 |
| 4 | 2 | 170163815 | rs142594441 | C | T | LRP2 | D | 11.41 | . | 260 | 230 | 0 | 5 | 5 | 0.001 |
| 4 | 2 | 179404792 | rs556524594 | C | T | TTN | . | 4.57 | 1.7 | 416 | 368 | 2 | 3 | 3 | 0.001 |
| 4 | 2 | 179425208 | rs142478636 | G | T | TTN | D | 4.57 | 1.7 | 416 | 368 | 2 | 3 | 3 | 0.004 |
| 4 | 2 | 179430305 | rs185887755 | G | A | TTN | D | 4.57 | 1.7 | 416 | 368 | 2 | 3 | 3 | 0.003 |
| 4 | 2 | 179437342 | rs567446185 | C | T | TTN | D | 4.57 | 1.7 | 416 | 368 | 2 | 3 | 3 | 0.001 |
| 4 | 2 | 179481839 | rs144688960 | C | A | TTN | . | 4.57 | 1.7 | 416 | 368 | 2 | 3 | 3 | 0.001 |
| 4 | 2 | 179504772 | rs551963261 | C | T | TTN | . | 4.57 | 1.7 | 416 | 368 | 2 | 3 | 3 | 0.001 |
| 4 | 2 | 179577222 | rs186857044 | C | A | TTN | D | 4.57 | 1.7 | 416 | 368 | 2 | 3 | 3 | 0.001 |
| 4 | 2 | 179585717 | rs367826445 | C | T | TTN | D | 4.57 | 1.7 | 416 | 368 | 2 | 3 | 3 | 0.002 |
| 4 | 2 | 203058233 | rs13024221 | T | C | KIAA2012 | . | 9.13 | 2.26 | 104 | 92 | 1 | 2 | 2 | 0.003 |
| 4 | 2 | 203059076 | rs141298049 | G | A | KIAA2012 | . | 9.13 | 2.26 | 104 | 92 | 1 | 2 | 2 | 0.006 |
| 4 | 22 | 31522450 | rs150976596 | G | A | INPP5J | D | 4.57 | 3.39 | 104 | 92 | 1 | 3 | 3 | 0.0099 |
| 4 | 22 | 31522715 | rs370874308 | A | T | INPP5J | . | 4.57 | 3.39 | 104 | 92 | 1 | 3 | 3 | 0.003 |
| 4 | 22 | 32614713 | rs78144589 | C | T | SLC5A4 | . | 6.85 | 3.39 | 104 | 92 | 1 | 3 | 3 | 0.0099 |
| 4 | 22 | 32631002 | rs554791323 | T | C | SLC5A4 | D | 6.85 | 3.39 | 104 | 92 | 1 | 3 | 3 | 0.001 |
| 4 | 2 | 70031769 | rs193084283 | A | G | ANXA4 | . | 1.83 | 2.26 | 104 | 92 | 1 | 2 | 2 | 0.0079 |
| 4 | 2 | 70039849 | rs184226986 | G | A | ANXA4 | . | 1.83 | 2.26 | 104 | 92 | 1 | 2 | 2 | 0.0079 |
| 4 | 2 | 71801442 | rs147483765 | C | T | DYSF | D | 3.04 | 2.26 | 156 | 138 | 1 | 2 | 2 | 0.002 |

| group | chr | pos | id | ref | alt | gene | LR | **Gene burden ratio** | | **Total No. of alleles**  **in gene** | | | **No. alt alleles**  **in genes** | | | **No. of ≥3-Year**  **cases with**  **alt alleles** | | **Variant allele frequency** | |
| --- | --- | --- | --- | --- | --- | --- | --- | --- | --- | --- | --- | --- | --- | --- | --- | --- | --- | --- | --- |
|  |  |  |  |  |  |  |  | KG  East Asia | ≤1-Year | | ≤1-Year | ≥3-Year | | ≤1-Year | ≥3-Year | |  | | KG  East Asia |
| 4 | 2 | 71901318 | rs573892877 | C | G | DYSF | . | 3.04 | 2.26 | | 156 | 138 | | 1 | 2 | | 2 | | 0.001 |
| 4 | 2 | 71901432 | rs144355449 | C | T | DYSF | . | 3.04 | 2.26 | | 156 | 138 | | 1 | 2 | | 2 | | 0.003 |
| 4 | 3 | 130282510 | rs150427289 | T | C | COL6A6 | . | 2.28 | 1.51 | | 208 | 184 | | 3 | 4 | | 4 | | 0.0089 |
| 4 | 3 | 130286067 | rs145020873 | A | G | COL6A6 | D | 2.28 | 1.51 | | 208 | 184 | | 3 | 4 | | 4 | | 0.0099 |
| 4 | 3 | 130289976 | rs200963433 | C | T | COL6A6 | D | 2.28 | 1.51 | | 208 | 184 | | 3 | 4 | | 4 | | 0.004 |
| 4 | 3 | 130346196 | rs117951912 | G | A | COL6A6 | D | 2.28 | 1.51 | | 208 | 184 | | 3 | 4 | | 4 | | 0.0069 |
| 4 | 3 | 148904379 | rs555339346 | C | G | CP | D | 9.13 | 2.26 | | 156 | 138 | | 1 | 2 | | 2 | | 0.001 |
| 4 | 3 | 148917507 | rs17847018 | T | C | CP | D | 9.13 | 2.26 | | 156 | 138 | | 1 | 2 | | 2 | | 0.003 |
| 4 | 3 | 148930242 | rs563241895 | A | T | CP | D | 9.13 | 2.26 | | 156 | 138 | | 1 | 2 | | 2 | | 0.002 |
| 4 | 3 | 183822730 | rs560673114 | C | CATTCCTCT | HTR3E | . | 2.28 | 2.26 | | 156 | 138 | | 1 | 2 | | 2 | | 0.0089 |
| 4 | 3 | 183823729 | rs187832026 | G | T | HTR3E | D | 2.28 | 2.26 | | 156 | 138 | | 1 | 2 | | 2 | | 0.0069 |
| 4 | 3 | 183823919 | rs532417196 | T | C | HTR3E | D | 2.28 | 2.26 | | 156 | 138 | | 1 | 2 | | 2 | | 0.001 |
| 4 | 3 | 2928719 | rs184171731 | A | C | CNTN4 | . | 6.85 | 1.7 | | 156 | 138 | | 2 | 3 | | 3 | | 0.003 |
| 4 | 3 | 3080611 | rs10510251 | G | C | CNTN4 | . | 6.85 | 1.7 | | 156 | 138 | | 2 | 3 | | 3 | | 0.003 |
| 4 | 3 | 3081959 | rs339284 | T | C | CNTN4 | . | 6.85 | 1.7 | | 156 | 138 | | 2 | 3 | | 3 | | 0.002 |
| 4 | 3 | 62309627 | rs1881268 | G | C | C3orf14 | . | 4.57 | . | | 104 | 92 | | 0 | 3 | | 3 | | 0.001 |
| 4 | 3 | 62317022 | rs186089632 | C | A | C3orf14 | . | 4.57 | . | | 104 | 92 | | 0 | 3 | | 3 | | 0.003 |
| 4 | 4 | 106158550 | rs141975400 | G | T | TET2 | . | 4.57 | 2.26 | | 52 | 46 | | 1 | 2 | | 2 | | 0.005 |
| 4 | 4 | 6596385 | rs3216941 | AC | A | MAN2B2 | . | 9.13 | 2.26 | | 52 | 46 | | 1 | 2 | | 2 | | 0.006 |
| 4 | 4 | 983115 | rs143381873 | G | A | SLC26A1 | D | 4.57 | 2.26 | | 208 | 184 | | 1 | 2 | | 2 | | 0.005 |
| 4 | 4 | 983342 | rs201608921 | C | T | SLC26A1 | D | 4.57 | 2.26 | | 208 | 184 | | 1 | 2 | | 2 | | 0.002 |
| 4 | 4 | 983810 | rs563866785 | G | A | SLC26A1 | D | 4.57 | 2.26 | | 208 | 184 | | 1 | 2 | | 2 | | 0.001 |
| 4 | 4 | 984938 | rs139024319 | G | A | SLC26A1 | D | 4.57 | 2.26 | | 208 | 184 | | 1 | 2 | | 2 | | 0.002 |
| 4 | 5 | 1495038 | rs201521332 | G | A | LPCAT1 | . | 9.55 | 2.36 | | 52 | 44 | | 1 | 2 | | 2 | | 0.0069 |
| 4 | 5 | 180477285 | rs200884524 | C | T | BTNL9 | . | 4.57 | . | | 104 | 92 | | 0 | 3 | | 3 | | 0.001 |
| 4 | 5 | 180483533 | rs373494500 | T | C | BTNL9 | . | 4.57 | . | | 104 | 92 | | 0 | 3 | | 3 | | 0.0069 |
| 4 | 6 | 169622491 | rs138932100 | G | A | THBS2 | D | 2.28 | 2.26 | | 208 | 184 | | 1 | 2 | | 2 | | 0.006 |
| 4 | 6 | 169623562 | rs182173220 | G | A | THBS2 | D | 2.28 | 2.26 | | 208 | 184 | | 1 | 2 | | 2 | | 0.001 |
| 4 | 6 | 169628312 | rs368102843 | C | T | THBS2 | D | 2.28 | 2.26 | | 208 | 184 | | 1 | 2 | | 2 | | 0.001 |
| 4 | 6 | 169646282 | rs76393784 | A | T | THBS2 | . | 2.28 | 2.26 | | 208 | 184 | | 1 | 2 | | 2 | | 0.002 |

| group | chr | pos | id | ref | alt | gene | LR | **Gene burden ratio** | | **Total No. of alleles**  **in gene** | | **No. alt alleles**  **in genes** | | **No. of ≥3-Year**  **cases with**  **alt alleles** | | **Variant allele frequency** | |
| --- | --- | --- | --- | --- | --- | --- | --- | --- | --- | --- | --- | --- | --- | --- | --- | --- | --- |
|  |  |  |  |  |  |  |  | KG  East Asia | ≤1-Year | ≤1-Year | ≥3-Year | ≤1-Year | ≥3-Year | |  | | KG  East Asia |
| 4 | 6 | 35438350 | rs187631484 | C | T | MIR7111 | . | 4.57 | 2.26 | 52 | 46 | 1 | 2 | | 2 | | 0.0069 |
| 4 | 6 | 35438350 | rs187631484 | C | T | RPL10A | . | 4.57 | 2.26 | 52 | 46 | 1 | 2 | | 2 | | 0.0069 |
| 4 | 6 | 43160731 | rs568565110 | C | G | CUL9 | . | 9.13 | 2.26 | 156 | 138 | 1 | 2 | | 2 | | 0.001 |
| 4 | 6 | 43170522 | rs200509434 | G | T | CUL9 | D | 9.13 | 2.26 | 156 | 138 | 1 | 2 | | 2 | | 0.0069 |
| 4 | 6 | 43172581 | rs80345623 | G | A | CUL9 | D | 9.13 | 2.26 | 156 | 138 | 1 | 2 | | 2 | | 0.0099 |
| 4 | 6 | 49416648 | rs199555550 | G | A | MUT | . | 13.7 | . | 156 | 138 | 0 | 3 | | 3 | | 0.0079 |
| 4 | 6 | 49425591 | rs200908035 | T | C | MUT | D | 13.7 | . | 156 | 138 | 0 | 3 | | 3 | | 0.0079 |
| 4 | 6 | 49425720 | rs528689712 | T | C | MUT | D | 13.7 | . | 156 | 138 | 0 | 3 | | 3 | | 0.001 |
| 4 | 7 | 100357429 | rs374243234 | C | T | ZAN | . | 3.04 | 2.26 | 156 | 138 | 1 | 2 | | 2 | | 0.001 |
| 4 | 7 | 100363045 | rs184742914 | A | T | ZAN | . | 3.04 | 2.26 | 156 | 138 | 1 | 2 | | 2 | | 0.003 |
| 4 | 7 | 100389715 | rs369936309 | C | T | ZAN | D | 3.04 | 2.26 | 156 | 138 | 1 | 2 | | 2 | | 0.003 |
| 4 | 7 | 128483506 | rs200215903 | G | A | FLNC | D | 2.28 | 3.39 | 208 | 184 | 1 | 3 | | 3 | | 0.005 |
| 4 | 7 | 128485314 | rs199917473 | G | A | FLNC | . | 2.28 | 3.39 | 208 | 184 | 1 | 3 | | 3 | | 0.003 |
| 4 | 7 | 128490926 | rs140857707 | C | T | FLNC | D | 2.28 | 3.39 | 208 | 184 | 1 | 3 | | 3 | | 0.002 |
| 4 | 7 | 128497224 | rs180834558 | G | T | FLNC | D | 2.28 | 3.39 | 208 | 184 | 1 | 3 | | 3 | | 0.0099 |
| 4 | 7 | 149481919 | rs561989729 | C | G | SSPO | . | 2.79 | 2.71 | 1484 | 1312 | 5 | 12 | | 12 | | 0.002 |
| 4 | 7 | 149484595 | rs532285725 | A | G | SSPO | . | 2.79 | 2.71 | 1484 | 1312 | 5 | 12 | | 12 | | 0.004 |
| 4 | 7 | 149484976 | rs372638209 | G | A | SSPO | . | 2.79 | 2.71 | 1484 | 1312 | 5 | 12 | | 12 | | 0.002 |
| 4 | 7 | 149486719 | rs185269282 | C | G | SSPO | . | 2.79 | 2.71 | 1484 | 1312 | 5 | 12 | | 12 | | 0.003 |
| 4 | 7 | 149489049 | rs189781142 | G | T | SSPO | . | 2.79 | 2.71 | 1484 | 1312 | 5 | 12 | | 12 | | 0.002 |
| 4 | 7 | 149490676 | rs4725314 | C | T | SSPO | . | 2.79 | 2.71 | 1484 | 1312 | 5 | 12 | | 12 | | 0.001 |
| 4 | 7 | 149491991 | rs550645855 | G | A | SSPO | . | 2.79 | 2.71 | 1484 | 1312 | 5 | 12 | | 12 | | 0.003 |
| 4 | 7 | 149492720 | rs573097199 | G | A | SSPO | . | 2.79 | 2.71 | 1484 | 1312 | 5 | 12 | | 12 | | 0.001 |
| 4 | 7 | 149493767 | rs118118675 | G | A | SSPO | . | 2.79 | 2.71 | 1484 | 1312 | 5 | 12 | | 12 | | 0.004 |
| 4 | 7 | 149494380 | rs376898523 | C | T | SSPO | . | 2.79 | 2.71 | 1484 | 1312 | 5 | 12 | | 12 | | 0.002 |
| 4 | 7 | 149501078 | rs147663076 | C | A | SSPO | . | 2.79 | 2.71 | 1484 | 1312 | 5 | 12 | | 12 | | 0.005 |
| 4 | 7 | 149502637 | rs375487670 | C | T | SSPO | . | 2.79 | 2.71 | 1484 | 1312 | 5 | 12 | | 12 | | 0.0069 |
| 4 | 7 | 149503944 | rs191161538 | C | T | SSPO | . | 2.79 | 2.71 | 1484 | 1312 | 5 | 12 | | 12 | | 0.004 |
| 4 | 7 | 149506195 | rs73727627 | C | T | SSPO | . | 2.79 | 2.71 | 1484 | 1312 | 5 | 12 | | 12 | | 0.001 |

| group | chr | pos | id | ref | alt | gene | LR | **Gene burden ratio** | | **Total No. of alleles**  **in gene** | | **No. alt alleles**  **in genes** | | **No. of ≥3-Year**  **cases with**  **alt alleles** | | **Variant allele frequency** | |
| --- | --- | --- | --- | --- | --- | --- | --- | --- | --- | --- | --- | --- | --- | --- | --- | --- | --- |
|  |  |  |  |  |  |  |  | KG  East Asia | ≤1-Year | ≤1-Year | ≥3-Year | ≤1-Year | ≥3-Year | |  | | KG  East Asia |
| 4 | 7 | 149509035 | rs189816441 | A | G | SSPO | . | 2.79 | 2.71 | 1484 | 1312 | 5 | 12 | | 12 | | 0.006 |
| 4 | 7 | 149509064 | rs73727632 | T | C | SSPO | . | 2.79 | 2.71 | 1484 | 1312 | 5 | 12 | | 12 | | 0.004 |
| 4 | 7 | 149509079 | rs73727633 | T | C | SSPO | . | 2.79 | 2.71 | 1484 | 1312 | 5 | 12 | | 12 | | 0.004 |
| 4 | 7 | 149509381 | rs757724 | C | T | SSPO | . | 2.79 | 2.71 | 1484 | 1312 | 5 | 12 | | 12 | | 0.004 |
| 4 | 7 | 149509407 | rs146934333 | G | C | SSPO | . | 2.79 | 2.71 | 1484 | 1312 | 5 | 12 | | 12 | | 0.0089 |
| 4 | 7 | 149509691 | rs73727635 | G | A | SSPO | . | 2.79 | 2.71 | 1484 | 1312 | 5 | 12 | | 12 | | 0.004 |
| 4 | 7 | 149515870 | rs371607382 | G | A | SSPO | . | 2.79 | 2.71 | 1484 | 1312 | 5 | 12 | | 12 | | 0.001 |
| 4 | 7 | 149518144 | rs577743302 | A | C | SSPO | . | 2.79 | 2.71 | 1484 | 1312 | 5 | 12 | | 12 | | 0.001 |
| 4 | 7 | 149519649 | rs58369703 | G | C | SSPO | . | 2.79 | 2.71 | 1484 | 1312 | 5 | 12 | | 12 | | 0.003 |
| 4 | 7 | 149519705 | rs55857423 | G | A | SSPO | . | 2.79 | 2.71 | 1484 | 1312 | 5 | 12 | | 12 | | 0.003 |
| 4 | 7 | 149519711 | rs547007891 | G | T | SSPO | . | 2.79 | 2.71 | 1484 | 1312 | 5 | 12 | | 12 | | 0.005 |
| 4 | 7 | 149521545 | rs143632762 | G | A | SSPO | . | 2.79 | 2.71 | 1484 | 1312 | 5 | 12 | | 12 | | 0.001 |
| 4 | 17 | 67246623 | rs559974558 | G | A | ABCA5 | D | 2.9 | 1.5 | 652 | 580 | 6 | 8 | | 8 | | 0.001 |
| 4 | 17 | 67247973 | rs201343208 | G | A | ABCA5 | D | 2.9 | 1.5 | 652 | 580 | 6 | 8 | | 8 | | 0.001 |
| 4 | 17 | 67250466 | rs199641093 | C | T | ABCA5 | D | 2.9 | 1.5 | 652 | 580 | 6 | 8 | | 8 | | 0.004 |
| 4 | 17 | 67299017 | rs201944918 | A | G | ABCA5 | D | 2.9 | 1.5 | 652 | 580 | 6 | 8 | | 8 | | 0.0079 |
| 4 | 17 | 67305519 | rs199888749 | G | A | ABCA5 | D | 2.9 | 1.5 | 652 | 580 | 6 | 8 | | 8 | | 0.0079 |
| 4 | 17 | 73827216 | rs140184929 | C | T | UNC13D | D | 5.43 | . | 264 | 232 | 0 | 3 | | 3 | | 0.006 |
| 4 | 17 | 73839609 | rs527842266 | C | G | UNC13D | . | 5.43 | . | 264 | 232 | 0 | 3 | | 3 | | 0.002 |
| 4 | 17 | 7701543 | rs141742705 | G | A | DNAH2 | D | 1.81 | 2.28 | 396 | 348 | 1 | 2 | | 2 | | 0.001 |
| 4 | 17 | 7705344 | rs8073196 | G | C | DNAH2 | . | 1.81 | 2.28 | 396 | 348 | 1 | 2 | | 2 | | 0.001 |
| 4 | 17 | 7736250 | rs201527036 | G | A | DNAH2 | . | 1.81 | 2.28 | 396 | 348 | 1 | 2 | | 2 | | 0.001 |
| 4 | 17 | 79684531 | rs201577202 | C | T | SLC25A10 | . | 3.62 | 1.52 | 264 | 232 | 3 | 4 | | 4 | | 0.001 |
| 4 | 17 | 79684871 | rs77609145 | A | T | SLC25A10 | D | 3.62 | 1.52 | 264 | 232 | 3 | 4 | | 4 | | 0.006 |
| 4 | 18 | 2707800 | rs184984483 | C | T | SMCHD1 | . | 5.48 | 3.37 | 258 | 230 | 1 | 3 | | 3 | | 0.001 |
| 4 | 18 | 2777922 | rs527648000 | C | T | SMCHD1 | . | 5.48 | 3.37 | 258 | 230 | 1 | 3 | | 3 | | 0.005 |
| 4 | 18 | 28911778 | rs147775289 | T | C | DSG1 | D | 5.43 | 1.71 | 528 | 464 | 2 | 3 | | 3 | | 0.003 |
| 4 | 18 | 28934293 | rs149191001 | C | T | DSG1 | D | 5.43 | 1.71 | 528 | 464 | 2 | 3 | | 3 | | 0.001 |
| 4 | 18 | 28934674 | rs181411154 | G | A | DSG1 | D | 5.43 | 1.71 | 528 | 464 | 2 | 3 | | 3 | | 0.001 |
| 4 | 18 | 28934927 | rs148488583 | C | G | DSG1 | D | 5.43 | 1.71 | 528 | 464 | 2 | 3 | | 3 | | 0.004 |

| group | chr | pos | id | ref | alt | gene | LR | **Gene burden ratio** | | **Total No. of alleles**  **in gene** | | **No. alt alleles**  **in genes** | | **No. of ≥3-Year**  **cases with**  **alt alleles** | | **Variant allele frequency** | |
| --- | --- | --- | --- | --- | --- | --- | --- | --- | --- | --- | --- | --- | --- | --- | --- | --- | --- |
|  |  |  |  |  |  |  |  | KG  East Asia | ≤1-Year | ≤1-Year | ≥3-Year | ≤1-Year | ≥3-Year | |  | | KG  East Asia |
| 4 | 18 | 580853 | rs114933134 | G | A | CETN1 | D | 3.62 | . | 132 | 116 | 0 | 4 | | 4 | | 0.005 |
| 4 | 18 | 61160178 | rs370525785 | T | C | SERPINB5 | . | 3.62 | 2.28 | 264 | 232 | 1 | 2 | | 2 | | 0.001 |
| 4 | 18 | 61170818 | rs185364126 | G | A | SERPINB5 | D | 3.62 | 2.28 | 264 | 232 | 1 | 2 | | 2 | | 0.002 |
| 4 | 18 | 61305002 | rs201297323 | T | C | SERPINB4 | D | 1.81 | 3.41 | 264 | 232 | 1 | 3 | | 3 | | 0.0069 |
| 4 | 18 | 61305289 | rs188021365 | A | T | SERPINB4 | . | 1.81 | 3.41 | 264 | 232 | 1 | 3 | | 3 | | 0.005 |
| 4 | 1 | 87380851 | rs546745 | A | G | HS2ST1 | . | 3.62 | 2.28 | 264 | 232 | 1 | 2 | | 2 | | 0.006 |
| 4 | 1 | 87563514 | rs143260332 | G | A | HS2ST1 | . | 3.62 | 2.28 | 264 | 232 | 1 | 2 | | 2 | | 0.004 |
| 4 | 18 | 76886315 | rs200431802 | C | T | ATP9B | D | 3.62 | 2.28 | 264 | 232 | 1 | 2 | | 2 | | 0.004 |
| 4 | 18 | 77096664 | rs201172611 | G | A | ATP9B | D | 3.62 | 2.28 | 264 | 232 | 1 | 2 | | 2 | | 0.001 |
| 4 | 18 | 9549345 | rs199964908 | G | A | PPP4R1 | . | 2.72 | 1.71 | 132 | 116 | 2 | 3 | | 3 | | 0.0079 |
| 4 | 19 | 14071095 | rs140301367 | G | A | DCAF15 | . | 5.43 | 3.41 | 132 | 116 | 1 | 3 | | 3 | | 0.0079 |
| 4 | 7 | 149521647 | rs564348526 | C | T | SSPO | . | 2.79 | 2.71 | 1484 | 1312 | 5 | 12 | | 12 | | 0.0069 |
| 4 | 7 | 149521654 | rs578088844 | G | A | SSPO | . | 2.79 | 2.71 | 1484 | 1312 | 5 | 12 | | 12 | | 0.002 |
| 4 | 7 | 149522951 | rs200469643 | C | T | SSPO | . | 2.79 | 2.71 | 1484 | 1312 | 5 | 12 | | 12 | | 0.001 |
| 4 | 7 | 75192236 | . | C | A | HIP1 | . | 4.57 | 2.26 | 104 | 92 | 1 | 2 | | 2 | | 0.0089 |
| 4 | 7 | 75210547 | . | A | T | HIP1 | . | 4.57 | 2.26 | 104 | 92 | 1 | 2 | | 2 | | 0.001 |
| 4 | 8 | 110439252 | rs375463553 | C | A | PKHD1L1 | D | 4.57 | 2.26 | 208 | 184 | 1 | 2 | | 2 | | 0.005 |
| 4 | 8 | 110463357 | rs202241413 | C | T | PKHD1L1 | D | 4.57 | 2.26 | 208 | 184 | 1 | 2 | | 2 | | 0.003 |
| 4 | 8 | 110493660 | rs139600051 | A | G | PKHD1L1 | . | 4.57 | 2.26 | 208 | 184 | 1 | 2 | | 2 | | 0.005 |
| 4 | 8 | 110527435 | rs559437602 | C | T | PKHD1L1 | D | 4.57 | 2.26 | 208 | 184 | 1 | 2 | | 2 | | 0.002 |
| 4 | 8 | 145736896 | rs557256260 | C | T | RECQL4 | . | 2.28 | 1.7 | 260 | 230 | 2 | 3 | | 3 | | 0.001 |
| 4 | 8 | 145738985 | rs536831548 | G | C | RECQL4 | . | 2.28 | 1.7 | 260 | 230 | 2 | 3 | | 3 | | 0.001 |
| 4 | 8 | 145741388 | rs200097701 | C | G | RECQL4 | . | 2.28 | 1.7 | 260 | 230 | 2 | 3 | | 3 | | 0.005 |
| 4 | 8 | 145741602 | rs34633809 | C | T | RECQL4 | . | 2.28 | 1.7 | 260 | 230 | 2 | 3 | | 3 | | 0.0089 |
| 4 | 8 | 145742799 | rs34642881 | T | C | RECQL4 | . | 2.28 | 1.7 | 260 | 230 | 2 | 3 | | 3 | | 0.0079 |
| 4 | 8 | 17400906 | rs12680645 | G | A | SLC7A2 | D | 3.42 | 1.7 | 156 | 138 | 2 | 3 | | 3 | | 0.0079 |
| 4 | 8 | 17407821 | rs188973136 | C | G | SLC7A2 | D | 3.42 | 1.7 | 156 | 138 | 2 | 3 | | 3 | | 0.006 |
| 4 | 8 | 17417839 | rs201373242 | A | G | SLC7A2 | D | 3.42 | 1.7 | 156 | 138 | 2 | 3 | | 3 | | 0.001 |
| 4 | 9 | 439392 | rs117109271 | A | G | DOCK8 | . | 3.42 | 3.39 | 104 | 92 | 1 | 3 | | 3 | | 0.0099 |
| 4 | 9 | 441423 | rs188141951 | C | T | DOCK8 | . | 3.42 | 3.39 | 104 | 92 | 1 | 3 | | 3 | | 0.003 |

| group | chr | pos | id | ref | alt | gene | LR | **Gene burden ratio** | | **Total No. of alleles**  **in gene** | | **No. alt alleles**  **in genes** | | **No. of ≥3-Year**  **cases with**  **alt alleles** | **Variant allele frequency** |
| --- | --- | --- | --- | --- | --- | --- | --- | --- | --- | --- | --- | --- | --- | --- | --- |
|  |  |  |  |  |  |  |  | KG  East Asia | ≤1-Year | ≤1-Year | ≥3-Year | ≤1-Year | ≥3-Year |  | KG  East Asia |
| 4 | X | 1460714 | . | C | T | IL3RA | D | 9.33 | 2.31 | 104 | 90 | 1 | 2 | 2 | 0.001 |
| 4 | X | 1471130 | . | G | T | IL3RA | . | 9.33 | 2.31 | 104 | 90 | 1 | 2 | 2 | 0.004 |
| 5 | 2 | 113342071 | rs528909726 | G | A | CHCHD5 | . | . | . | 38 | 40 | 0 | 2 | 1 | 0.002 |
| 5 | 3 | 63898360 | rs576518931 | G | GGCAGCA | ATXN7 | . | . | 1.76 | 38 | 36 | 3 | 5 | 4 | 0.004 |
| 5 | 4 | 46994972 | rs34464680 | G | GAA | GABRA4 | . | 1.41 | 1.81 | 70 | 54 | 5 | 7 | 6 | 0.0089 |
| 6 | 10 | 122618148 | rs2241846 | G | C | WDR11 | . | 1.57 | 1.67 | 50 | 46 | 15 | 23 | 19 | 0.3472 |
| 6 | 10 | 35485028 | rs137918654 | G | T | CREM | . | 2.74 | 3.39 | 52 | 46 | 1 | 3 | 2 | 0.0347 |
| 6 | 10 | 44788826 | rs58189594 | C | T | C10orf142 | . | 1.87 | 3.39 | 52 | 46 | 3 | 9 | 8 | 0.1121 |
| 6 | 10 | 47087078 | rs2229967 | G | T | CH17-360D5.1 | . | 1.86 | 1.83 | 260 | 230 | 13 | 21 | 21 | . |
| 6 | 10 | 47087078 | rs2229967 | G | T | NPY4R | . | 1.86 | 1.83 | 260 | 230 | 13 | 21 | 21 | . |
| 6 | 10 | 47087403 | rs781881744 | T | C | CH17-360D5.1 | . | 1.86 | 1.83 | 260 | 230 | 13 | 21 | 21 | . |
| 6 | 10 | 47087403 | rs781881744 | T | C | NPY4R | . | 1.86 | 1.83 | 260 | 230 | 13 | 21 | 21 | . |
| 6 | 10 | 47087499 | rs114592738 | G | A | CH17-360D5.1 | . | 1.86 | 1.83 | 260 | 230 | 13 | 21 | 21 | . |
| 6 | 10 | 47087499 | rs114592738 | G | A | NPY4R | . | 1.86 | 1.83 | 260 | 230 | 13 | 21 | 21 | . |
| 6 | 10 | 47087520 | rs115443559 | G | A | CH17-360D5.1 | . | 1.86 | 1.83 | 260 | 230 | 13 | 21 | 21 | 0.0179 |
| 6 | 10 | 47087520 | rs115443559 | G | A | NPY4R | . | 1.86 | 1.83 | 260 | 230 | 13 | 21 | 21 | 0.0179 |
| 6 | 10 | 47087609 | rs79871698 | G | A | CH17-360D5.1 | . | 1.86 | 1.83 | 260 | 230 | 13 | 21 | 21 | 0.0923 |
| 6 | 10 | 47087609 | rs79871698 | G | A | NPY4R | . | 1.86 | 1.83 | 260 | 230 | 13 | 21 | 21 | 0.0923 |
| 6 | 10 | 7747155 | rs76983422 | G | C | ITIH2 | . | 2.74 | 1.7 | 52 | 46 | 2 | 3 | 3 | 0.0397 |
| 6 | 1 | 100617938 | rs78161968 | T | C | LRRC39 | . | 1.68 | 2.29 | 156 | 136 | 3 | 6 | 6 | 0.0446 |
| 6 | 1 | 100618085 | rs773979041 | A | G | LRRC39 | . | 1.68 | 2.29 | 156 | 136 | 3 | 6 | 6 | . |
| 6 | 1 | 100620728 | rs78962557 | G | A | LRRC39 | . | 1.68 | 2.29 | 156 | 136 | 3 | 6 | 6 | 0.0198 |
| 6 | 1 | 109456983 | rs141562079 | C | T | GPSM2 | D | 2.28 | 6.78 | 156 | 138 | 1 | 6 | 6 | 0.0258 |
| 6 | 1 | 109465156 | rs199964596 | TCAA | T | GPSM2 | . | 2.28 | 6.78 | 156 | 138 | 1 | 6 | 6 | 0.0129 |
| 6 | 1 | 109465165 | rs35029887 | ACTT | A | GPSM2 | . | 2.28 | 6.78 | 156 | 138 | 1 | 6 | 6 | . |
| 6 | 11 | 118886656 | rs7131534 | G | A | RPS25 | . | 1.52 | 1.7 | 52 | 46 | 2 | 3 | 3 | 0.0704 |
| 6 | 11 | 13410690 | rs375824034 | TGAAA | T | BTBD10 | . | 2.49 | 1.7 | 52 | 46 | 4 | 6 | 6 | 0.0675 |
| 6 | 11 | 16812551 | rs116885602 | C | T | PLEKHA7 | . | 1.61 | 1.85 | 100 | 90 | 6 | 10 | 9 | 0.0456 |
| 6 | 11 | 16863087 | rs452745 | A | G | PLEKHA7 | . | 1.61 | 1.85 | 100 | 90 | 6 | 10 | 9 | 0.1617 |
| 6 | 1 | 118530450 | rs184368389 | T | C | SPAG17 | . | 4.57 | 3.39 | 52 | 46 | 1 | 3 | 3 | 0.0179 |

| group | chr | pos | id | ref | alt | gene | LR | **Gene burden ratio** | | **Total No. of alleles**  **in gene** | | **No. alt alleles**  **in genes** | | | **No. of ≥3-Year**  **cases with**  **alt alleles** | | **Variant allele frequency** |
| --- | --- | --- | --- | --- | --- | --- | --- | --- | --- | --- | --- | --- | --- | --- | --- | --- | --- |
|  |  |  |  |  |  |  |  | KG  East Asia | ≤1-Year | ≤1-Year | ≥3-Year | ≤1-Year | ≥3-Year |  | | KG  East Asia | |
| 6 | 1 | 12082881 | rs11588779 | C | T | MIIP | . | 4.57 | 3.39 | 52 | 46 | 2 | 6 | 6 | | 0.0685 | |
| 6 | 11 | 22232870 | rs78987921 | G | A | ANO5 | . | 4.57 | 2.26 | 104 | 92 | 1 | 2 | 2 | | 0.0109 | |
| 6 | 11 | 22239801 | . | C | T | ANO5 | . | 4.57 | 2.26 | 104 | 92 | 1 | 2 | 2 | | . | |
| 6 | 11 | 32636495 | rs145888197 | T | C | CCDC73 | . | 1.52 | 2.26 | 52 | 46 | 1 | 2 | 2 | | 0.0377 | |
| 6 | 1 | 145414790 | . | G | C | HFE2 | . | 4.57 | 2.26 | 52 | 46 | 1 | 2 | 2 | | 0.0129 | |
| 6 | 1 | 145456731 | rs6694055 | G | C | POLR3GL | . | 1.83 | 2.26 | 52 | 46 | 1 | 2 | 2 | | 0.0119 | |
|  |  |  |  |  |  |  |  |  |  |  |  |  |  |  | |  | |
| 6 | 1 | 145527604 | rs2274620 | A | G | ITGA10 | . | 1.62 | 2.94 | 260 | 230 | 5 | 13 | 12 | | 0.0208 | |
| 6 | 1 | 145534221 | . | G | T | ITGA10 | . | 1.62 | 2.94 | 260 | 230 | 5 | 13 | 12 | | . | |
| 6 | 1 | 145536082 | rs2274616 | G | A | ITGA10 | . | 1.62 | 2.94 | 260 | 230 | 5 | 13 | 12 | | 0.0704 | |
| 6 | 1 | 145541806 | rs77912414 | T | C | ITGA10 | . | 1.62 | 2.94 | 260 | 230 | 5 | 13 | 12 | | 0.0119 | |
| 6 | 1 | 152484245 | rs2282298 | C | T | LCE5A | . | 2.28 | 3.39 | 52 | 46 | 1 | 3 | 3 | | 0.0228 | |
| 6 | 11 | 55136125 | rs117954374 | C | T | OR4A15 | . | 2.08 | 2.83 | 52 | 46 | 2 | 5 | 5 | | 0.0585 | |
| 6 | 11 | 551644 | rs113026126 | C | T | LRRC56 | . | 2.54 | 1.88 | 52 | 46 | 3 | 5 | 5 | | 0.0476 | |
| 6 | 11 | 5776484 | rs4910844 | A | T | OR52N4 | . | 1.56 | 2.64 | 52 | 46 | 6 | 14 | 10 | | 0.1974 | |
| 6 | 1 | 15834360 | rs2020902 | A | G | CASP9 | . | 3.04 | 2.26 | 52 | 46 | 1 | 2 | 2 | | 0.0317 | |
| 6 | 1 | 15860803 | rs11583306 | C | T | DNAJC16 | . | 2.74 | 1.7 | 52 | 46 | 2 | 3 | 3 | | 0.0466 | |
| 6 | 1 | 159799808 | rs10430458 | C | T | SLAMF8 | . | 3.42 | 1.7 | 52 | 46 | 2 | 3 | 3 | | 0.0317 | |
| 6 | 1 | 159858290 | rs3795334 | T | A | CFAP45 | . | 1.52 | 2.83 | 52 | 46 | 2 | 5 | 5 | | 0.0556 | |
| 6 | 11 | 61048196 | rs78505441 | G | C | VWCE | . | 1.83 | 2.26 | 52 | 46 | 1 | 2 | 2 | | 0.0268 | |
| 6 | 11 | 61071331 | rs28720346 | C | T | DDB1 | . | 1.83 | 2.26 | 52 | 46 | 1 | 2 | 2 | | 0.0258 | |
| 6 | 11 | 62365619 | rs11231155 | A | G | MTA2 | . | 1.83 | 2.26 | 52 | 46 | 2 | 4 | 4 | | 0.0526 | |
| 6 | 11 | 62369881 | rs35156678 | G | A | EML3 | . | 2.03 | 2.26 | 52 | 46 | 2 | 4 | 4 | | 0.0437 | |
| 6 | 1 | 16382911 | rs72474563 | A | G | CLCNKB | . | 9.13 | 2.26 | 52 | 46 | 2 | 4 | 4 | | 0.0357 | |
| 6 | 11 | 67771408 | rs188940236 | C | T | UNC93B1 | . | 1.62 | 2.2 | 44 | 40 | 2 | 4 | 4 | | 0.0308 | |
| 6 | 1 | 168105581 | rs200664972 | A | AG | GPR161 | . | 1.83 | 4.52 | 52 | 46 | 1 | 4 | 4 | | 0.0516 | |
| 6 | 11 | 71710425 | rs373342292 | CTCA | C | IL18BP | . | 3.65 | . | 52 | 46 | 0 | 4 | 4 | | 0.0198 | |
| 6 | 1 | 177247693 | rs138799872 | C | T | BRINP2 | . | 4.57 | 2.26 | 52 | 46 | 1 | 2 | 2 | | 0.0119 | |
| 6 | 11 | 797714 | . | T | A | PANO1 | . | 7.72 | 2.83 | 154 | 136 | 2 | 5 | 5 | | . | |
| 6 | 11 | 798082 | rs201547522 | C | CT | PANO1 | . | 7.72 | 2.83 | 154 | 136 | 2 | 5 | 5 | | 0.0248 | |
| 6 | 11 | 798222 | rs572464433 | T | TCGC | PANO1 | . | 7.72 | 2.83 | 154 | 136 | 2 | 5 | 5 | | . | |

| group | chr | pos | id | ref | alt | gene | LR | **Gene burden ratio** | | | **Total No. of alleles**  **in gene** | | | **No. alt alleles**  **in genes** | | | **No. of ≥3-Year**  **cases with**  **alt alleles** | | **Variant allele frequency** |
| --- | --- | --- | --- | --- | --- | --- | --- | --- | --- | --- | --- | --- | --- | --- | --- | --- | --- | --- | --- |
|  |  |  |  |  |  |  |  | KG  East Asia | ≤1-Year | ≤1-Year | | ≥3-Year | ≤1-Year | | ≥3-Year |  | | KG  East Asia | |
| 6 | 1 | 180240510 | rs2764449 | T | C | LHX4 | . | 2.28 | . | 156 | | 138 | 0 | | 3 | 3 | | 0.0139 | |
| 6 | 1 | 180243593 | rs200119009 | C | T | LHX4 | D | 2.28 | . | 156 | | 138 | 0 | | 3 | 3 | | . | |
| 6 | 1 | 180243601 | . | G | A | LHX4 | D | 2.28 | . | 156 | | 138 | 0 | | 3 | 3 | | . | |
| 6 | 1 | 201356001 | . | CCCA | * | LAD1 | . | 4.38 | 1.92 | 184 | | 160 | 12 | | 20 | 20 | | . | |
| 6 | 1 | 201356001 | rs398053706 | CCCA | C | LAD1 | . | 4.38 | 1.92 | 184 | | 160 | 12 | | 20 | 20 | | 0.0575 | |
| 6 | 1 | 201356004 | . | ACC | * | LAD1 | . | 4.38 | 1.92 | 184 | | 160 | 12 | | 20 | 20 | | . | |
| 6 | 1 | 201356004 | rs552300739 | ACC | A | LAD1 | . | 4.38 | 1.92 | 184 | | 160 | 12 | | 20 | 20 | | 0.0486 | |
| 6 | 1 | 210267893 | rs144713062 | TGAA | T | SYT14 | . | 3.2 | 3.96 | 52 | | 46 | 2 | | 7 | 6 | | 0.0655 | |
| 6 | 12 | 110923012 | rs142702785 | A | G | FAM216A | . | 2.74 | . | 52 | | 46 | 0 | | 3 | 3 | | 0.0149 | |
| 6 | 12 | 113592306 | rs200344876 | G | GC | CFAP73 | . | 3.42 | 3.39 | 52 | | 46 | 1 | | 3 | 3 | | 0.0169 | |
| 6 | 1 | 2117557 | rs547643542 | G | A | FAAP20 | . | 3.65 | 1.51 | 104 | | 92 | 6 | | 8 | 7 | | 0.0139 | |
| 6 | 12 | 122243731 | rs557334621 | C | T | SETD1B | . | 2.28 | 2.26 | 156 | | 138 | 1 | | 2 | 2 | | . | |
| 6 | 12 | 122255354 | rs541427059 | TGCG | T | SETD1B | . | 2.28 | 2.26 | 156 | | 138 | 1 | | 2 | 2 | | 0.0129 | |
| 6 | 12 | 122265770 | rs117774166 | G | A | SETD1B | . | 2.28 | 2.26 | 156 | | 138 | 1 | | 2 | 2 | | 0.0188 | |
| 6 | 12 | 12630665 | rs142947418 | TGCACGCTGG | T | DUSP16 | . | 1.55 | 1.64 | 178 | | 154 | 48 | | 68 | 57 | | 0.13 | |
| 6 | 12 | 12630675 | rs201941751 | GCACGC | G | DUSP16 | . | 1.55 | 1.64 | 178 | | 154 | 48 | | 68 | 57 | | 0.2698 | |
| 6 | 12 | 12630681 | rs200271649 | TGGGC | T | DUSP16 | . | 1.55 | 1.64 | 178 | | 154 | 48 | | 68 | 57 | | 0.2698 | |
| 6 | 12 | 12633287 | rs10845555 | A | G | DUSP16 | . | 1.55 | 1.64 | 178 | | 154 | 48 | | 68 | 57 | | 0.4097 | |
| 6 | 1 | 2130262 | rs144802031 | C | G | FAAP20 | . | 3.65 | 1.51 | 104 | | 92 | 6 | | 8 | 7 | | 0.0754 | |
| 6 | 12 | 14923935 | rs199781231 | T | TC | HIST4H4 | . | 1.52 | 2.26 | 52 | | 46 | 1 | | 2 | 2 | | 0.0198 | |
| 6 | 12 | 16377347 | rs117974895 | C | T | SLC15A5 | . | 2.28 | 1.7 | 52 | | 46 | 2 | | 3 | 3 | | 0.0337 | |
| 6 | 1 | 224008913 | rs3738370 | G | A | TP53BP2 | . | 1.89 | 1.51 | 52 | | 46 | 9 | | 12 | 11 | | 0.1925 | |
| 6 | 1 | 228289872 | rs373634959 | G | A | C1orf35 | . | 2.28 | 2.26 | 52 | | 46 | 1 | | 2 | 2 | | 0.0139 | |
| 6 | 1 | 22924364 | rs72651347 | G | A | EPHA8 | . | 2.28 | 1.7 | 104 | | 92 | 2 | | 3 | 3 | | 0.0179 | |
| 6 | 1 | 22927298 | rs569320402 | C | T | EPHA8 | D | 2.28 | 1.7 | 104 | | 92 | 2 | | 3 | 3 | | . | |
| 6 | 1 | 234509972 | rs73099933 | A | G | COA6 | . | 2.5 | 2.89 | 52 | | 42 | 3 | | 7 | 7 | | 0.0804 | |
| 6 | 1 | 247719769 | rs56043070 | G | A | GCSAML | . | 2.28 | 3.39 | 52 | | 46 | 1 | | 3 | 3 | | 0.0327 | |
| 6 | 1 | 248512939 | rs201185608 | AC | A | OR14C36 | . | 1.76 | 1.88 | 52 | | 46 | 3 | | 5 | 5 | | 0.0615 | |
| 6 | 12 | 49721122 | rs73309977 | C | T | TROAP | . | 1.96 | 3.39 | 52 | | 46 | 1 | | 3 | 3 | | 0.0397 | |

| group | chr | pos | id | ref | alt | gene | LR | **Gene burden ratio** | | | **Total No. of alleles**  **in gene** | | | **No. alt alleles**  **in genes** | | | **No. of ≥3-Year**  **cases with**  **alt alleles** | | **Variant allele frequency** |
| --- | --- | --- | --- | --- | --- | --- | --- | --- | --- | --- | --- | --- | --- | --- | --- | --- | --- | --- | --- |
|  |  |  |  |  |  |  |  | KG  East Asia | ≤1-Year | ≤1-Year | | ≥3-Year | ≤1-Year | | ≥3-Year |  | | KG  East Asia | |
| 6 | 12 | 51510213 | rs77417603 | T | A | TFCP2 | . | 2.28 | 2.26 | 52 | | 46 | 2 | | 4 | 4 | | 0.0228 | |
| 6 | 12 | 52156281 | rs187002252 | A | G | SCN8A | . | 1.83 | 2.26 | 52 | | 46 | 1 | | 2 | 2 | | 0.0179 | |
| 6 | 12 | 53427826 | rs140133257 | G | T | EIF4B | . | 2.74 | . | 52 | | 46 | 0 | | 3 | 3 | | 0.0228 | |
| 6 | 12 | 54756528 | rs77759698 | A | G | GPR84 | D | 1.76 | 5.65 | 52 | | 46 | 1 | | 5 | 5 | | 0.0347 | |
| 6 | 12 | 55523586 | rs398102299 | AT | A | OR9K2 | . | 1.66 | 3.01 | 52 | | 46 | 3 | | 8 | 7 | | 0.1141 | |
| 6 | 12 | 55641255 | rs4522268 | C | T | OR6C74 | . | 1.96 | 3.39 | 52 | | 46 | 3 | | 9 | 8 | | 0.1171 | |
| 6 | 12 | 55759191 | rs398102300 | AT | A | OR6C75 | . | 2.28 | 3.96 | 52 | | 46 | 2 | | 7 | 7 | | 0.0724 | |
| 6 | 12 | 58007149 | rs141337782 | G | C | ARHGEF25 | . | 1.52 | 2.83 | 52 | | 46 | 2 | | 5 | 4 | | 0.1151 | |
| 6 | 1 | 26524503 | rs199601379 | C | T | CATSPER4 | D | 1.71 | 3.39 | 104 | | 92 | 1 | | 3 | 2 | | . | |
| 6 | 1 | 26526554 | rs6700024 | G | A | CATSPER4 | . | 1.71 | 3.39 | 104 | | 92 | 1 | | 3 | 2 | | 0.0208 | |
| 6 | 12 | 70928745 | rs3752702 | G | A | PTPRB | . | 3.04 | 2.26 | 52 | | 46 | 1 | | 2 | 2 | | 0.0486 | |
| 6 | 12 | 95456367 | rs138805411 | T | C | NR2C1 | D | 4.57 | 4.52 | 52 | | 46 | 1 | | 4 | 4 | | 0.0208 | |
| 6 | 13 | 76457183 | rs9530477 | T | C | LMO7DN | . | 1.66 | 1.88 | 52 | | 46 | 12 | | 20 | 15 | | 0.3284 | |
| 6 | 14 | 35182348 | rs35515423 | GA | G | CFL2 | . | 1.55 | 1.7 | 46 | | 44 | 8 | | 13 | 11 | | 0.2034 | |
| 6 | 14 | 94750501 | rs77844573 | A | G | SERPINA10 | . | 1.6 | 1.98 | 104 | | 92 | 4 | | 7 | 7 | | 0.0258 | |
| 6 | 14 | 94756458 | rs2232699 | A | T | SERPINA10 | D | 1.6 | 1.98 | 104 | | 92 | 4 | | 7 | 7 | | 0.0298 | |
| 6 | 15 | 101013123 | rs1566775 | G | A | CERS3 | . | 4.57 | 2.26 | 52 | | 46 | 1 | | 2 | 2 | | 0.0258 | |
| 6 | 15 | 101601367 | rs148929418 | C | CACTT | LRRK1 | . | 3.26 | 2.26 | 52 | | 46 | 5 | | 10 | 10 | | 0.1409 | |
| 6 | 15 | 28947605 | rs3893142 | C | A | GOLGA8M | . | 1.75 | 1.53 | 46 | | 40 | 3 | | 4 | 4 | | 0.0675 | |
| 6 | 15 | 60803458 | rs779626945 | C | T | RORA | D | 2.28 | . | 104 | | 92 | 0 | | 3 | 3 | | . | |
| 6 | 15 | 60919432 | rs73424068 | C | T | RORA | . | 2.28 | . | 104 | | 92 | 0 | | 3 | 3 | | 0.0149 | |
| 6 | 15 | 78458485 | rs3816253 | T | C | IDH3A | . | 1.64 | 1.58 | 52 | | 46 | 10 | | 14 | 11 | | 0.1885 | |
| 6 | 16 | 1272750 | rs113856625 | G | A | TPSG1 | D | 2.13 | 3.96 | 104 | | 92 | 2 | | 7 | 7 | | . | |
| 6 | 16 | 1273444 | rs61587627 | G | T | TPSG1 | D | 2.13 | 3.96 | 104 | | 92 | 2 | | 7 | 7 | | 0.0972 | |
| 6 | 16 | 13297242 | rs13331224 | C | T | SHISA9 | . | 1.64 | 1.57 | 48 | | 46 | 6 | | 9 | 9 | | 0.1042 | |
| 6 | 16 | 1825689 | rs3826055 | C | T | EME2 | . | 6.85 | 1.7 | 104 | | 92 | 2 | | 3 | 3 | | 0.0129 | |
| 6 | 16 | 1825789 | rs746707908 | T | C | EME2 | . | 6.85 | 1.7 | 104 | | 92 | 2 | | 3 | 3 | | . | |
| 6 | 1 | 6266793 | rs72853039 | G | C | RNF207 | . | 1.71 | 3.39 | 52 | | 46 | 2 | | 6 | 6 | | 0.0972 | |
| 6 | 16 | 28998111 | rs4788115 | T | A | LAT | . | 1.74 | 2.26 | 52 | | 46 | 4 | | 8 | 8 | | 0.1359 | |

| group | chr | pos | id | ref | alt | gene | LR | **Gene burden ratio** | | **Total No. of alleles**  **in gene** | | **No. alt alleles**  **in genes** | | **No. of ≥3-Year**  **cases with**  **alt alleles** | **Variant allele frequency** |
| --- | --- | --- | --- | --- | --- | --- | --- | --- | --- | --- | --- | --- | --- | --- | --- |
|  |  |  |  |  |  |  |  | KG  East Asia | ≤1-Year | ≤1-Year | ≥3-Year | ≤1-Year | ≥3-Year |  | KG  East Asia |
| 6 | 16 | 30455945 | rs146596728 | A | C | SEPHS2 | . | 4.57 | 2.26 | 104 | 92 | 1 | 2 | 2 | 0.0248 |
| 6 | 16 | 30456188 | rs550048089 | G | A | SEPHS2 | . | 4.57 | 2.26 | 104 | 92 | 1 | 2 | 2 | . |
| 6 | 16 | 3075701 | rs2717664 | C | T | THOC6 | . | 1.72 | 2.75 | 104 | 92 | 14 | 34 | 28 | 0.1825 |
| 6 | 16 | 3075999 | rs2245000 | C | G | THOC6 | . | 1.72 | 2.75 | 104 | 92 | 14 | 34 | 28 | 0.3046 |
| 6 | 16 | 3554840 | rs80187466 | G | T | CLUAP1 | . | 1.96 | . | 156 | 138 | 0 | 9 | 9 | 0.0556 |
| 6 | 16 | 3558283 | rs59492947 | A | T | CLUAP1 | . | 1.96 | . | 156 | 138 | 0 | 9 | 9 | 0.0556 |
| 6 | 16 | 3580565 | rs79684678 | T | C | CLUAP1 | . | 1.96 | . | 156 | 138 | 0 | 9 | 9 | 0.0556 |
| 6 | 16 | 5077897 | rs112669475 | G | A | NAGPA | . | 2.03 | . | 52 | 46 | 0 | 4 | 4 | 0.0397 |
| 6 | 16 | 57736047 | rs72795521 | G | A | DRC7 | . | 1.71 | 1.7 | 156 | 138 | 14 | 21 | 17 | 0.2669 |
| 6 | 16 | 57756907 | rs113469607 | C | A | DRC7 | . | 1.71 | 1.7 | 156 | 138 | 14 | 21 | 17 | 0.0218 |
| 6 | 16 | 57757046 | rs139945134 | C | T | DRC7 | . | 1.71 | 1.7 | 156 | 138 | 14 | 21 | 17 | 0.0129 |
| 6 | 1 | 6647702 | rs183072854 | G | A | ZBTB48 | . | 3.04 | 2.26 | 52 | 46 | 1 | 2 | 2 | 0.0268 |
| 6 | 16 | 67180171 | rs7184692 | T | C | C16orf70 | . | 2.54 | 2.83 | 52 | 46 | 2 | 5 | 5 | 0.0595 |
| 6 | 16 | 83998662 | rs733728 | A | G | OSGIN1 | . | 1.68 | . | 52 | 46 | 0 | 7 | 7 | 0.0962 |
| 6 | 16 | 84801966 | rs189466547 | T | C | USP10 | . | 4.57 | 2.26 | 52 | 46 | 1 | 2 | 2 | 0.0109 |
| 6 | 17 | 27067480 | rs750108245 | G | A | NEK8 | . | 1.9 | 2.83 | 208 | 184 | 2 | 5 | 5 | . |
| 6 | 17 | 27067558 | rs565763400 | C | T | NEK8 | . | 1.9 | 2.83 | 208 | 184 | 2 | 5 | 5 | . |
| 6 | 17 | 27068005 | rs147832976 | TGAG | T | NEK8 | . | 1.9 | 2.83 | 208 | 184 | 2 | 5 | 5 | 0.0407 |
| 6 | 17 | 27068012 | rs757972103 | G | T | NEK8 | . | 1.9 | 2.83 | 208 | 184 | 2 | 5 | 5 | . |
| 6 | 17 | 34182099 | rs149317141 | G | GT | HEATR9 | . | 1.56 | 1.7 | 156 | 138 | 20 | 30 | 27 | 0.0198 |
| 6 | 17 | 34185535 | rs35283303 | AG | A | HEATR9 | . | 1.56 | 1.7 | 156 | 138 | 20 | 30 | 27 | 0.3224 |
| 6 | 17 | 34192406 | . | G | A | HEATR9 | . | 1.56 | 1.7 | 156 | 138 | 20 | 30 | 27 | 0.0952 |
| 6 | 17 | 36895514 | rs72819704 | A | G | PCGF2 | . | 1.63 | 1.88 | 104 | 92 | 6 | 10 | 9 | 0.0169 |
| 6 | 17 | 36896534 | rs2075057 | C | T | PCGF2 | . | 1.63 | 1.88 | 104 | 92 | 6 | 10 | 9 | 0.0962 |
| 6 | 17 | 37824838 | . | GCAA | G | PNMT | . | 2.28 | 4.52 | 104 | 92 | 1 | 4 | 4 | . |
| 6 | 17 | 37826201 | rs60871117 | C | T | PNMT | . | 2.28 | 4.52 | 104 | 92 | 1 | 4 | 4 | 0.0357 |
| 6 | 17 | 48753044 | rs371874263 | C | T | ABCC3 | D | 2.28 | 1.51 | 208 | 184 | 3 | 4 | 4 | . |
| 6 | 17 | 48755450 | rs11568583 | A | G | ABCC3 | . | 2.28 | 1.51 | 208 | 184 | 3 | 4 | 4 | 0.0258 |
| 6 | 17 | 48761020 | rs572541933 | G | A | ABCC3 | D | 2.28 | 1.51 | 208 | 184 | 3 | 4 | 4 | . |
| 6 | 17 | 48765100 | rs756871504 | C | T | ABCC3 | . | 2.28 | 1.51 | 208 | 184 | 3 | 4 | 4 | . |

| group | chr | pos | id | ref | alt | gene | LR | **Gene burden ratio** | | **Total No. of alleles**  **in gene** | | **No. alt alleles**  **in genes** | | **No. of ≥3-Year**  **cases with**  **alt alleles** | **Variant allele frequency** |
| --- | --- | --- | --- | --- | --- | --- | --- | --- | --- | --- | --- | --- | --- | --- | --- |
|  |  |  |  |  |  |  |  | KG  East Asia | ≤1-Year | ≤1-Year | ≥3-Year | ≤1-Year | ≥3-Year |  | KG  East Asia |
| 6 | 17 | 4906146 | rs10533622 | GC | G | KIF1C | . | 1.7 | 1.75 | 164 | 194 | 30 | 62 | 53 | . |
| 6 | 17 | 4906146 | rs146311497 | GCC | G | KIF1C | . | 1.7 | 1.75 | 164 | 194 | 30 | 62 | 53 | 0.3244 |
| 6 | 17 | 4906146 | rs763524690 | G | GC | KIF1C | . | 1.7 | 1.75 | 164 | 194 | 30 | 62 | 53 | . |
| 6 | 17 | 4907374 | rs766141834 | G | A | KIF1C | . | 1.7 | 1.75 | 164 | 194 | 30 | 62 | 53 | . |
| 6 | 17 | 4924097 | rs4790725 | C | G | KIF1C | . | 1.7 | 1.75 | 164 | 194 | 30 | 62 | 53 | 0.0446 |
| 6 | 17 | 59667953 | rs17610181 | G | A | NACA2 | . | 1.77 | 1.94 | 52 | 46 | 7 | 12 | 11 | 0.1667 |
| 6 | 17 | 64876769 | rs376464596 | C | T | CACNG5 | D | 2.09 | 1.55 | 156 | 138 | 8 | 11 | 9 | . |
| 6 | 17 | 64876770 | rs142916987 | G | A | CACNG5 | D | 2.09 | 1.55 | 156 | 138 | 8 | 11 | 9 | . |
| 6 | 17 | 64880788 | rs2286677 | G | A | CACNG5 | . | 2.09 | 1.55 | 156 | 138 | 8 | 11 | 9 | 0.126 |
| 6 | 17 | 76130947 | rs62079073 | G | T | TMC8 | . | 1.52 | 1.58 | 52 | 46 | 5 | 7 | 7 | 0.0804 |
| 6 | 17 | 8109965 | rs144397670 | G | A | AURKB | . | 1.71 | 3.36 | 206 | 184 | 1 | 3 | 3 | 0.0159 |
| 6 | 17 | 8110079 | rs139322514 | G | A | AURKB | . | 1.71 | 3.36 | 206 | 184 | 1 | 3 | 3 | . |
| 6 | 17 | 8113270 | rs766965552 | A | G | AURKB | . | 1.71 | 3.36 | 206 | 184 | 1 | 3 | 3 | . |
| 6 | 17 | 8113544 | . | C | G | AURKB | . | 1.71 | 3.36 | 206 | 184 | 1 | 3 | 3 | . |
| 6 | 18 | 33694444 | rs148550301 | A | G | SLC39A6 | . | 4.57 | . | 52 | 46 | 0 | 3 | 3 | 0.0298 |
| 6 | 1 | 89523927 | rs60070945 | C | T | GBP1 | . | 1.71 | 2.03 | 52 | 46 | 5 | 9 | 9 | 0.12 |
| 6 | 19 | 10426524 | rs79442975 | G | A | FDX1L | . | 1.83 | 2.26 | 52 | 46 | 1 | 2 | 2 | 0.0397 |
| 6 | 19 | 12875807 | rs115585485 | A | C | HOOK2 | . | 1.96 | 3.39 | 52 | 46 | 1 | 3 | 3 | 0.0238 |
| 6 | 19 | 1917687 | rs138069352 | C | G | SCAMP4 | . | 1.71 | 3.39 | 52 | 46 | 1 | 3 | 3 | 0.0437 |
| 6 | 1 | 92798945 | rs78196083 | C | T | RPAP2 | . | 1.52 | 2.26 | 52 | 46 | 1 | 2 | 2 | 0.0456 |
| 6 | 19 | 32083223 | rs11880125 | A | G | THEG5 | . | 4.57 | 1.7 | 104 | 92 | 4 | 6 | 6 | 0.0179 |
| 6 | 19 | 32083250 | rs79323410 | T | C | THEG5 | . | 4.57 | 1.7 | 104 | 92 | 4 | 6 | 6 | 0.0179 |
| 6 | 19 | 39905903 | rs3859551 | A | G | PLEKHG2 | . | 1.64 | 1.7 | 260 | 230 | 4 | 6 | 6 | 0.0387 |
| 6 | 19 | 39906985 | rs10401595 | T | C | PLEKHG2 | . | 1.64 | 1.7 | 260 | 230 | 4 | 6 | 6 | 0.0387 |
| 6 | 19 | 39907573 | rs763779951 | C | G | PLEKHG2 | D | 1.64 | 1.7 | 260 | 230 | 4 | 6 | 6 | . |
| 6 | 19 | 39915627 | rs200639701 | A | G | PLEKHG2 | D | 1.64 | 1.7 | 260 | 230 | 4 | 6 | 6 | . |
| 6 | 19 | 39915764 | . | A | G | PLEKHG2 | D | 1.64 | 1.7 | 260 | 230 | 4 | 6 | 6 | . |
| 6 | 19 | 39948307 | rs2304215 | C | T | SUPT5H | . | 1.52 | 2.26 | 52 | 46 | 1 | 2 | 2 | 0.0129 |
| 6 | 19 | 40327312 | rs3760924 | A | G | FBL | . | 2.28 | 2.54 | 52 | 46 | 4 | 9 | 8 | 0.0992 |
| 6 | 19 | 42603776 | rs1205817 | A | G | POU2F2 | . | 1.52 | 1.7 | 52 | 46 | 8 | 12 | 11 | 0.1865 |

| group | chr | pos | id | ref | alt | gene | LR | **Gene burden ratio** | | **Total No. of alleles**  **in gene** | | **No. alt alleles**  **in genes** | | **No. of ≥3-Year**  **cases with**  **alt alleles** | | **Variant allele frequency** | |
| --- | --- | --- | --- | --- | --- | --- | --- | --- | --- | --- | --- | --- | --- | --- | --- | --- | --- |
|  |  |  |  |  |  |  |  | KG  East Asia | ≤1-Year | ≤1-Year | ≥3-Year | ≤1-Year | ≥3-Year | |  | | KG  East Asia |
| 6 | 19 | 43268140 | rs11355507 | AG | A | PSG8 | . | 1.96 | 1.7 | 52 | 46 | 2 | 3 | | 3 | | 0.0188 |
| 6 | 19 | 45296846 | rs66944506 | A | AC | CBLC | . | 1.68 | 1.98 | 104 | 92 | 4 | 7 | | 7 | | 0.0962 |
| 6 | 19 | 45297454 | rs1903831 | A | C | CBLC | . | 1.68 | 1.98 | 104 | 92 | 4 | 7 | | 7 | | 0.0863 |
| 6 | 19 | 47290651 | rs3826793 | G | T | SLC1A5 | . | 1.56 | 1.63 | 52 | 46 | 9 | 13 | | 11 | | 0.2212 |
| 6 | 19 | 48735017 | rs140826611 | C | CTT | CARD8 | . | 2.28 | 1.51 | 52 | 46 | 3 | 4 | | 4 | | 0.0575 |
| 6 | 19 | 49956688 | rs78750735 | T | C | ALDH16A1 | . | 1.8 | 1.72 | 208 | 180 | 39 | 58 | | 52 | | . |
| 6 | 19 | 49965131 | rs76844851 | G | A | ALDH16A1 | . | 1.8 | 1.72 | 208 | 180 | 39 | 58 | | 52 | | 0.3313 |
| 6 | 19 | 49965131 | rs76844851 | G | C | ALDH16A1 | . | 1.8 | 1.72 | 208 | 180 | 39 | 58 | | 52 | | 0.0149 |
| 6 | 19 | 49965132 | rs79109084 | G | C | ALDH16A1 | . | 1.8 | 1.72 | 208 | 180 | 39 | 58 | | 52 | | 0.3313 |
| 6 | 19 | 51330423 | rs61752560 | C | G | KLK15 | . | 2.28 | 1.81 | 52 | 46 | 5 | 8 | | 7 | | 0.1032 |
| 6 | 19 | 51582802 | rs199715229 | C | T | KLK14 | D | 4.57 | 2.26 | 104 | 92 | 1 | 2 | | 2 | | 0.0119 |
| 6 | 19 | 51585822 | rs769468261 | G | A | KLK14 | . | 4.57 | 2.26 | 104 | 92 | 1 | 2 | | 2 | | . |
| 6 | 19 | 54578105 | . | C | T | TARM1 | . | 2.74 | . | 52 | 46 | 0 | 3 | | 3 | | 0.0268 |
| 6 | 19 | 58118371 | rs78803667 | G | A | ZNF530 | D | 6.85 | 3.39 | 52 | 46 | 1 | 3 | | 3 | | 0.0159 |
| 6 | 19 | 7688614 | rs2335521 | T | C | XAB2 | . | 2.85 | 5.65 | 52 | 46 | 1 | 5 | | 5 | | 0.0407 |
| 6 | 19 | 7935408 | . | A | ACACTGGGG  GTGAGGCA  GGGGGAGAG  AAAGGGGCCTG | PRR36 | . | 6.49 | 6.74 | 218 | 194 | 1 | 6 | | 6 | | . |
| 6 | 19 | 7935423 | rs759755075 | CAGGGGGAG  AGAAAGGGG  CCTGCACTGG  GGGTGAGGG | C | PRR36 | . | 6.49 | 6.74 | 218 | 194 | 1 | 6 | | 6 | | . |
| 6 | 19 | 7936105 | . | A | T | PRR36 | . | 6.49 | 6.74 | 218 | 194 | 1 | 6 | | 6 | | . |
| 6 | 19 | 7937299 | . | C | T | PRR36 | . | 6.49 | 6.74 | 218 | 194 | 1 | 6 | | 6 | | . |
| 6 | 19 | 829555 | rs144713752 | C | G | AZU1 | . | 2.74 | . | 52 | 46 | 0 | 3 | | 3 | | 0.0129 |
| 6 | 19 | 9236698 | rs111279560 | G | GATGGT | OR7G3 | . | 1.51 | 1.64 | 152 | 134 | 18 | 26 | | 22 | | 0.3651 |
| 6 | 19 | 9236916 | rs75266995 | AG | A | OR7G3 | . | 1.51 | 1.64 | 152 | 134 | 18 | 26 | | 22 | | 0.0317 |
| 6 | 19 | 9236969 | rs61751875 | G | A | OR7G3 | . | 1.51 | 1.64 | 152 | 134 | 18 | 26 | | 22 | | 0.0357 |
| 6 | 20 | 23584368 | rs118095359 | G | A | CST9 | . | 1.83 | 2.26 | 52 | 46 | 1 | 2 | | 2 | | 0.0129 |
| 6 | 20 | 31672812 | rs71349705 | C | T | BPIFB4 | . | 6.85 | . | 104 | 92 | 0 | 3 | | 3 | | . |

| group | chr | pos | id | ref | alt | gene | LR | **Gene burden ratio** | | **Total No. of alleles**  **in gene** | | **No. alt alleles**  **in genes** | | **No. of ≥3-Year**  **cases with**  **alt alleles** | | **Variant allele frequency** | |
| --- | --- | --- | --- | --- | --- | --- | --- | --- | --- | --- | --- | --- | --- | --- | --- | --- | --- |
|  |  |  |  |  |  |  |  | KG  East Asia | ≤1-Year | ≤1-Year | ≥3-Year | ≤1-Year | ≥3-Year | |  | | KG  East Asia |
| 6 | 20 | 31677295 | rs142982767 | C | T | BPIFB4 | . | 6.85 | . | 104 | 92 | 0 | 3 | | 3 | | 0.0179 |
| 6 | 20 | 32005736 | rs116972153 | G | A | SNTA1 | . | 1.52 | 1.7 | 52 | 46 | 2 | 3 | | 3 | | 0.0238 |
| 6 | 20 | 4228485 | rs3746669 | G | T | ADRA1D | . | 1.5 | 2.26 | 52 | 46 | 11 | 22 | | 17 | | 0.2887 |
| 6 | 20 | 44180813 | rs17348421 | G | A | WFDC8 | . | 2.28 | 2.83 | 52 | 46 | 2 | 5 | | 4 | | 0.0585 |
| 6 | 20 | 44511257 | rs35972756 | G | A | ZSWIM1 | . | 4.57 | 2.26 | 52 | 46 | 1 | 2 | | 2 | | 0.0159 |
| 6 | 20 | 44676727 | rs12481488 | T | A | SLC12A5 | . | 3.42 | 3.39 | 52 | 46 | 1 | 3 | | 3 | | 0.0238 |
| 6 | 20 | 50307365 | rs117858424 | A | G | ATP9A | . | 1.66 | 2.26 | 52 | 46 | 2 | 4 | | 4 | | 0.0476 |
| 6 | 2 | 121997127 | rs147546143 | G | GACGGT | TFCP2L1 | . | 2.03 | 2.26 | 52 | 46 | 2 | 4 | | 4 | | 0.0565 |
| 6 | 2 | 128466446 | . | G | A | WDR33 | D | 1.9 | . | 208 | 184 | 0 | 5 | | 5 | | . |
| 6 | 2 | 128477849 | rs774069217 | C | T | WDR33 | D | 1.9 | . | 208 | 184 | 0 | 5 | | 5 | | . |
| 6 | 2 | 128522203 | rs117753184 | A | T | WDR33 | . | 1.9 | . | 208 | 184 | 0 | 5 | | 5 | | 0.0258 |
| 6 | 2 | 128522852 | . | G | A | WDR33 | . | 1.9 | . | 208 | 184 | 0 | 5 | | 5 | | . |
| 6 | 21 | 34860749 | . | CAATTA | C | DNAJC28 | . | 1.9 | 2.83 | 52 | 46 | 4 | 10 | | 9 | | 0.1667 |
| 6 | 21 | 42615293 | rs2252576 | C | T | BACE2 | . | 1.52 | 2.83 | 52 | 46 | 2 | 5 | | 4 | | 0.0675 |
| 6 | 21 | 43412786 | rs200509586 | GTCA | G | ZBTB21 | . | 11.41 | 5.65 | 52 | 46 | 1 | 5 | | 5 | | 0.0109 |
| 6 | 2 | 153515710 | rs141445791 | A | C | PRPF40A | . | 1.56 | . | 104 | 90 | 0 | 4 | | 4 | | 0.0278 |
| 6 | 2 | 153515879 | rs767401165 | A | G | PRPF40A | . | 1.56 | . | 104 | 90 | 0 | 4 | | 4 | | . |
| 6 | 2 | 169791766 | . | G | A | ABCB11 | D | 4.57 | 3.39 | 156 | 138 | 1 | 3 | | 3 | | . |
| 6 | 2 | 169801131 | rs118109635 | G | A | ABCB11 | D | 4.57 | 3.39 | 156 | 138 | 1 | 3 | | 3 | | 0.0129 |
| 6 | 2 | 169853135 | . | A | G | ABCB11 | . | 4.57 | 3.39 | 156 | 138 | 1 | 3 | | 3 | | . |
| 6 | 2 | 175304621 | rs67227536 | C | G | GPR155 | . | 6.85 | 3.39 | 104 | 92 | 1 | 3 | | 3 | | 0.0139 |
| 6 | 2 | 175333632 | rs28588913 | G | A | GPR155 | . | 6.85 | 3.39 | 104 | 92 | 1 | 3 | | 3 | | 0.0248 |
| 6 | 2 | 202498027 | rs78297522 | T | C | TMEM237 | . | 1.96 | 1.7 | 52 | 46 | 2 | 3 | | 3 | | 0.0298 |
| 6 | 22 | 19420778 | rs3747064 | T | A | MRPL40 | . | 1.52 | 4.52 | 52 | 46 | 1 | 4 | | 4 | | 0.0714 |
| 6 | 22 | 24313530 | rs199896117 | GGA | G | DDTL | . | 2.28 | 2.26 | 52 | 46 | 1 | 2 | | 2 | | 0.0278 |
| 6 | 22 | 24919647 | rs118163237 | G | A | UPB1 | D | 1.83 | 2.26 | 52 | 46 | 1 | 2 | | 2 | | 0.0188 |
| 6 | 2 | 231077154 | rs41309096 | G | A | SP110 | . | 1.83 | 1.7 | 52 | 46 | 4 | 6 | | 6 | | 0.0883 |
| 6 | 2 | 30862980 | rs12466818 | C | T | LCLAT1 | . | 1.52 | 1.98 | 52 | 46 | 8 | 14 | | 11 | | 0.2222 |
| 6 | 2 | 31412347 | rs78099670 | G | A | CAPN14 | . | 7.3 | 1.51 | 260 | 230 | 3 | 4 | | 4 | | 0.0179 |

| group | chr | pos | id | ref | alt | gene | LR | **Gene burden ratio** | | **Total No. of alleles**  **in gene** | | | **No. alt alleles**  **in genes** | | **No. of ≥3-Year**  **cases with**  **alt alleles** | **Variant allele frequency** |
| --- | --- | --- | --- | --- | --- | --- | --- | --- | --- | --- | --- | --- | --- | --- | --- | --- |
|  |  |  |  |  |  |  |  | KG  East Asia | ≤1-Year | | ≤1-Year | ≥3-Year | ≤1-Year | ≥3-Year |  | KG  East Asia |
| 6 | 2 | 31414833 | . | G | T | CAPN14 | D | 7.3 | 1.51 | | 260 | 230 | 3 | 4 | 4 | . |
| 6 | 2 | 31414844 | rs147299374 | C | T | CAPN14 | D | 7.3 | 1.51 | | 260 | 230 | 3 | 4 | 4 | . |
| 6 | 2 | 31414959 | rs141014145 | A | G | CAPN14 | D | 7.3 | 1.51 | | 260 | 230 | 3 | 4 | 4 | 0.0149 |
| 6 | 2 | 31422395 | rs200657395 | TCTC | T | CAPN14 | . | 7.3 | 1.51 | | 260 | 230 | 3 | 4 | 4 | 0.0139 |
| 6 | 2 | 55491007 | rs369772725 | G | GA | MTIF2 | . | 2.75 | 5.18 | | 44 | 34 | 1 | 4 | 4 | 0.0486 |
| 6 | 2 | 98128073 | . | G | * | ANKRD36B | . | 1.86 | 2.71 | | 116 | 110 | 14 | 36 | 33 | . |
| 6 | 2 | 98128073 | rs373085949 | G | A | ANKRD36B | . | 1.86 | 2.71 | | 116 | 110 | 14 | 36 | 33 | . |
| 6 | 2 | 98164184 | rs13001728 | C | G | ANKRD36B | . | 1.86 | 2.71 | | 116 | 110 | 14 | 36 | 33 | 0.1796 |
| 6 | 3 | 100593675 | rs79152576 | T | C | ABI3BP | . | 2.28 | 2.26 | | 52 | 46 | 1 | 2 | 2 | 0.0139 |
| 6 | 3 | 107097080 | rs138204694 | CAAATG | C | CCDC54 | . | 2.61 | 4.52 | | 52 | 46 | 1 | 4 | 4 | 0.0317 |
| 6 | 3 | 111780629 | rs73853301 | C | T | TMPRSS7 | . | 3.04 | 4.52 | | 104 | 92 | 2 | 8 | 6 | 0.0258 |
| 6 | 3 | 111780630 | rs73853302 | C | T | TMPRSS7 | . | 3.04 | 4.52 | | 104 | 92 | 2 | 8 | 6 | 0.0258 |
| 6 | 3 | 120428621 | rs11720353 | T | C | RABL3 | . | 1.68 | 1.58 | | 52 | 46 | 5 | 7 | 7 | 0.127 |
| 6 | 3 | 122002576 | rs117375173 | A | G | CASR | D | 1.71 | 6.78 | | 104 | 92 | 1 | 6 | 6 | 0.0446 |
| 6 | 3 | 122002644 | rs768660050 | G | T | CASR | D | 1.71 | 6.78 | | 104 | 92 | 1 | 6 | 6 | . |
| 6 | 3 | 133331230 | rs71317417 | C | T | TOPBP1 | . | 2.28 | 2.26 | | 52 | 46 | 1 | 2 | 2 | 0.0437 |
| 6 | 3 | 182871464 | rs500288 | A | G | LAMP3 | . | 3.42 | 1.7 | | 52 | 46 | 2 | 3 | 3 | 0.0317 |
| 6 | 3 | 196296182 | rs79085393 | G | C | FBXO45 | . | 1.9 | 1.88 | | 52 | 46 | 3 | 5 | 5 | 0.0665 |
| 6 | 3 | 46714821 | rs11130104 | C | G | ALS2CL | . | 1.56 | 1.88 | | 52 | 46 | 18 | 30 | 20 | 0.3571 |
| 6 | 3 | 56682841 | rs71621834 | A | C | FAM208A | . | 3.18 | 2.36 | | 52 | 44 | 2 | 4 | 4 | 0.0327 |
| 6 | 3 | 58620105 | rs76752946 | G | C | FAM3D | . | 2.61 | 4.52 | | 52 | 46 | 1 | 4 | 4 | 0.0308 |
| 6 | 4 | 141458699 | rs149594258 | A | C | ELMOD2 | . | 1.96 | 3.39 | | 52 | 46 | 1 | 3 | 3 | 0.0208 |
| 6 | 4 | 184367558 | rs10533201 | TCTG | T | CDKN2AIP | . | 1.66 | 2.01 | | 52 | 46 | 9 | 16 | 13 | 0.244 |
| 6 | 4 | 20751278 | rs2322688 | A | G | KCNIP4 | . | 1.65 | 1.84 | | 52 | 46 | 8 | 13 | 11 | 0.1726 |
| 6 | 4 | 48517296 | rs757286932 | C | A | FRYL | . | 1.52 | 2.83 | | 260 | 230 | 2 | 5 | 5 | . |
| 6 | 4 | 48545814 | rs10517225 | A | T | FRYL | . | 1.52 | 2.83 | | 260 | 230 | 2 | 5 | 5 | 0.0238 |
| 6 | 4 | 48546796 | rs78799039 | A | G | FRYL | . | 1.52 | 2.83 | | 260 | 230 | 2 | 5 | 5 | . |
| 6 | 4 | 48549674 | rs776615697 | GAGA | G | FRYL | . | 1.52 | 2.83 | | 260 | 230 | 2 | 5 | 5 | . |
| 6 | 4 | 48559138 | rs779161058 | AG | A | FRYL | . | 1.52 | 2.83 | | 260 | 230 | 2 | 5 | 5 | . |
| 6 | 4 | 48993993 | rs749533750 | CTTG | C | CWH43 | . | 1.71 | . | | 104 | 92 | 0 | 3 | 3 | . |

| group | chr | pos | id | ref | alt | gene | LR | **Gene burden ratio** | | **Total No. of alleles**  **in gene** | | | **No. alt alleles**  **in genes** | | | **No. of ≥3-Year**  **cases with**  **alt alleles** | **Variant allele frequency** | |
| --- | --- | --- | --- | --- | --- | --- | --- | --- | --- | --- | --- | --- | --- | --- | --- | --- | --- | --- |
|  |  |  |  |  |  |  |  | KG  East Asia | ≤1-Year | | ≤1-Year | ≥3-Year | | ≤1-Year | ≥3-Year |  | | KG  East Asia |
| 6 | 4 | 49034669 | rs147750792 | CA | C | CWH43 | . | 1.71 | . | | 104 | 92 | | 0 | 3 | 3 | | 0.0149 |
| 6 | 4 | 69094459 | rs75647314 | C | A | TMPRSS11B | . | 3.04 | 2.26 | | 104 | 92 | | 2 | 4 | 4 | | 0.0278 |
| 6 | 4 | 69096987 | rs575638339 | C | T | TMPRSS11B | D | 3.04 | 2.26 | | 104 | 92 | | 2 | 4 | 4 | | . |
| 6 | 4 | 70078281 | rs62298955 | G | C | UGT2B11 | . | 1.96 | 3.39 | | 52 | 46 | | 1 | 3 | 3 | | 0.0476 |
| 6 | 4 | 77940418 | rs28541859 | T | A | SEPT11 | . | 1.52 | 2.26 | | 52 | 46 | | 4 | 8 | 8 | | 0.1012 |
| 6 | 5 | 151784206 | rs145273801 | G | A | NMUR2 | . | 1.71 | 1.7 | | 104 | 92 | | 2 | 3 | 3 | | 0.0248 |
| 6 | 5 | 151784490 | rs762380505 | C | A | NMUR2 | D | 1.71 | 1.7 | | 104 | 92 | | 2 | 3 | 3 | | . |
| 6 | 5 | 170221307 | rs117380156 | G | A | GABRP | . | 3.42 | 3.39 | | 104 | 92 | | 1 | 3 | 3 | | 0.0159 |
| 6 | 5 | 170236578 | rs558177227 | C | T | GABRP | D | 3.42 | 3.39 | | 104 | 92 | | 1 | 3 | 3 | | . |
| 6 | 5 | 74998426 | rs17649248 | G | A | POC5 | . | 1.62 | 1.98 | | 104 | 92 | | 12 | 21 | 19 | | 0.119 |
| 6 | 5 | 75008193 | rs2047059 | T | C | POC5 | . | 1.62 | 1.98 | | 104 | 92 | | 12 | 21 | 19 | | 0.2153 |
| 6 | 6 | 106978193 | rs17495742 | A | G | AIM1 | . | 1.76 | 2.79 | | 154 | 138 | | 2 | 5 | 4 | | . |
| 6 | 6 | 106991361 | rs61741114 | T | C | AIM1 | D | 1.76 | 2.79 | | 154 | 138 | | 2 | 5 | 4 | | . |
| 6 | 6 | 107016343 | rs3747789 | T | G | AIM1 | . | 1.76 | 2.79 | | 154 | 138 | | 2 | 5 | 4 | | 0.0694 |
| 6 | 6 | 10927469 | rs770638323 | A | G | SYCP2L | . | 3.42 | 3.39 | | 104 | 92 | | 1 | 3 | 2 | | . |
| 6 | 6 | 10935424 | rs181416897 | C | T | SYCP2L | . | 3.42 | 3.39 | | 104 | 92 | | 1 | 3 | 2 | | 0.0109 |
| 6 | 6 | 142487469 | rs225656 | C | A | VTA1 | . | 1.76 | 1.91 | | 104 | 92 | | 16 | 27 | 25 | | 0.1845 |
| 6 | 6 | 142510676 | rs3830800 | GTATT | G | VTA1 | . | 1.76 | 1.91 | | 104 | 92 | | 16 | 27 | 25 | | 0.1468 |
| 6 | 6 | 26410148 | rs77721150 | T | C | BTN3A1 | . | 2.71 | 4.52 | | 156 | 138 | | 2 | 8 | 8 | | 0.0248 |
| 6 | 6 | 26410227 | rs7770214 | G | A | BTN3A1 | . | 2.71 | 4.52 | | 156 | 138 | | 2 | 8 | 8 | | 0.0308 |
| 6 | 6 | 26410266 | . | T | C | BTN3A1 | . | 2.71 | 4.52 | | 156 | 138 | | 2 | 8 | 8 | | . |
| 6 | 6 | 28268497 | rs2281043 | C | T | PGBD1 | . | 2.85 | 2.83 | | 52 | 46 | | 4 | 10 | 9 | | 0.1448 |
| 6 | 6 | 30574428 | . | A | G | PPP1R10 | . | 3.42 | . | | 52 | 46 | | 0 | 3 | 3 | | 0.0218 |
| 6 | 6 | 30618867 | . | T | C | C6orf136 | . | 1.76 | 2.83 | | 52 | 46 | | 2 | 5 | 4 | | 0.0595 |
| 6 | 6 | 30670948 | rs536243116 | C | T | MDC1 | D | 2.11 | 3.39 | | 156 | 138 | | 2 | 6 | 5 | | . |
| 6 | 6 | 30679289 | rs147822906 | G | C | MDC1 | . | 2.11 | 3.39 | | 156 | 138 | | 2 | 6 | 5 | | 0.0198 |
| 6 | 6 | 30679510 | rs17189329 | G | A | MDC1 | . | 2.11 | 3.39 | | 156 | 138 | | 2 | 6 | 5 | | 0.0595 |
| 6 | 6 | 31733650 | rs707936 | G | A | VWA7 | . | 2.08 | . | | 52 | 46 | | 0 | 5 | 4 | | 0.0615 |
| 6 | 6 | 31948421 | . | TCTC | T | STK19 | . | 2.15 | 1.51 | | 52 | 46 | | 6 | 8 | 8 | | 0.0972 |
| 6 | 6 | 36929653 | rs144897670 | C | T | PI16 | . | 3.04 | 2.26 | | 52 | 46 | | 1 | 2 | 2 | | 0.0129 |

| group | chr | pos | id | ref | alt | gene | LR | **Gene burden ratio** | | **Total No. of alleles**  **in gene** | | | **No. alt alleles**  **in genes** | | | **No. of ≥3-Year**  **cases with**  **alt alleles** | | **Variant allele frequency** |
| --- | --- | --- | --- | --- | --- | --- | --- | --- | --- | --- | --- | --- | --- | --- | --- | --- | --- | --- |
|  |  |  |  |  |  |  |  | KG  East Asia | ≤1-Year | ≤1-Year | ≥3-Year | ≤1-Year | | ≥3-Year |  | | KG  East Asia | |
| 6 | 6 | 397261 | rs34318727 | G | A | IRF4 | . | 2.08 | 5.65 | 52 | 46 | 1 | | 5 | 5 | | 0.0496 | |
| 6 | 6 | 46133282 | rs16874326 | T | C | ENPP5 | D | 1.52 | 3.39 | 52 | 46 | 1 | | 3 | 3 | | 0.0407 | |
| 6 | 6 | 72011086 | rs16880821 | C | T | OGFRL1 | . | 4.57 | . | 52 | 46 | 0 | | 3 | 3 | | 0.0149 | |
| 6 | 6 | 87994504 | rs35259282 | C | T | GJB7 | D | 2.28 | 2.26 | 104 | 92 | 1 | | 2 | 2 | | 0.0188 | |
| 6 | 6 | 87994537 | rs112552839 | G | A | GJB7 | D | 2.28 | 2.26 | 104 | 92 | 1 | | 2 | 2 | | . | |
| 6 | 7 | 100230618 | rs41303468 | A | T | TFR2 | . | 2.28 | 2.26 | 52 | 46 | 1 | | 2 | 2 | | 0.0139 | |
| 6 | 7 | 140125701 | rs760033770 | G | A | RAB19 | . | 2.54 | 5.65 | 156 | 138 | 1 | | 5 | 5 | | . | |
| 6 | 7 | 140125753 | rs771901851 | G | A | RAB19 | D | 2.54 | 5.65 | 156 | 138 | 1 | | 5 | 5 | | . | |
| 6 | 7 | 140174292 | rs10709936 | CA | C | MKRN1 | . | 1.95 | 1.66 | 42 | 38 | 8 | | 12 | 10 | | 0.1766 | |
| 6 | 7 | 142562051 | rs143667567 | C | CCCTCCT | EPHB6 | . | 2.74 | 1.61 | 148 | 138 | 4 | | 6 | 6 | | 0.0238 | |
| 6 | 7 | 142562051 | rs143667567 | C | CCCT | EPHB6 | . | 2.74 | 1.61 | 148 | 138 | 4 | | 6 | 6 | | 0.0139 | |
| 6 | 7 | 142565743 | rs8177158 | G | A | EPHB6 | . | 2.74 | 1.61 | 148 | 138 | 4 | | 6 | 6 | | . | |
| 6 | 7 | 156468559 | rs3823617 | T | C | RNF32 | . | 1.52 | 1.7 | 52 | 46 | 4 | | 6 | 6 | | 0.1111 | |
| 6 | 7 | 28534518 | rs77306029 | C | T | CREB5 | . | 2.28 | 2.26 | 52 | 46 | 2 | | 4 | 4 | | 0.0397 | |
| 6 | 7 | 72984917 | . | CGTT | C | TBL2 | . | 4.57 | . | 52 | 46 | 0 | | 5 | 5 | | 0.0159 | |
| 6 | 8 | 17104886 | rs145945235 | G | A | VPS37A | . | 1.79 | 2.44 | 44 | 36 | 2 | | 4 | 4 | | 0.0526 | |
| 6 | 8 | 37791988 | rs201462725 | GT | G | GOT1L1 | . | 2.74 | 1.7 | 52 | 46 | 2 | | 3 | 3 | | 0.0337 | |
| 6 | 8 | 87163770 | rs150698519 | G | T | ATP6V0D2 | . | 1.83 | 2.26 | 52 | 46 | 1 | | 2 | 2 | | 0.0149 | |
| 6 | 8 | 95188916 | rs67774240 | G | A | CDH17 | . | 1.83 | 3.39 | 156 | 138 | 1 | | 3 | 3 | | 0.0278 | |
| 6 | 8 | 95189955 | rs138007982 | G | T | CDH17 | . | 1.83 | 3.39 | 156 | 138 | 1 | | 3 | 3 | | 0.0139 | |
| 6 | 8 | 95201518 | rs749070399 | TAAAAA | T | CDH17 | . | 1.83 | 3.39 | 156 | 138 | 1 | | 3 | 3 | | . | |
| 6 | 9 | 100693386 | rs201990544 | CACT | C | HEMGN | . | 6.85 | 3.39 | 52 | 46 | 1 | | 3 | 3 | | 0.0129 | |
| 6 | 9 | 101984010 | rs201959100 | G | A | ALG2 | D | 6.85 | . | 52 | 46 | 0 | | 3 | 3 | | 0.0188 | |
| 6 | 9 | 127074783 | rs139169292 | TC | T | NEK6 | . | 1.59 | 2.26 | 104 | 92 | 8 | | 16 | 16 | | 0.0923 | |
| 6 | 9 | 127076271 | rs56045213 | A | G | NEK6 | . | 1.59 | 2.26 | 104 | 92 | 8 | | 16 | 16 | | 0.0923 | |
| 6 | 9 | 5892552 | rs148372841 | G | C | MLANA | . | 9.13 | 4.52 | 52 | 46 | 1 | | 4 | 4 | | 0.0139 | |
| 6 | 9 | 95411725 | rs72756427 | G | A | IPPK | . | 2.74 | 1.7 | 52 | 46 | 2 | | 3 | 3 | | 0.0476 | |
| 6 | X | 31089629 | rs7057057 | C | A | FTHL17 | . | 1.73 | 2.26 | 52 | 46 | 3 | | 6 | 5 | | 0.1453 | |
| 6 | X | 49114808 | . | C | A | FOXP3 | D | 2.88 | 1.88 | 52 | 46 | 3 | | 5 | 4 | | 0.0393 | |
