# Supplementary material for: Identification of potential genetic causal variants for rheumatoid arthritis by whole-exome sequencing
Source: Oncotarget. 2017 Nov 22;8(67):111119–29. doi: 10.18632/oncotarget.22630 (PMC5762310; doi:10.18632/oncotarget.22630)
Supplement: Supplementary file 5 [file oncotarget-08-111119-s005.docx]

**Supplementary Table 6: High priority candidate gene list in Rheumatoid Arthritis disease.**

|  | Gene | SNP | | | | | | p-Value | | | | | | | Odd ratio | | | | | | | | | References | | | | |  | |  | | |  | |
| --- | --- | --- | --- | --- | --- | --- | --- | --- | --- | --- | --- | --- | --- | --- | --- | --- | --- | --- | --- | --- | --- | --- | --- | --- | --- | --- | --- | --- | --- | --- | --- | --- | --- | --- | --- |
|  | ABHD6 | rs73081554 | | | | | | 5.00E-08 | | | | | | | 1.18 | | | | | | | | | Okada Y,PMID: 24390342 | | | | |  | |  | | |  | |
|  | ACOXL | rs6732565 | | | | | | 3.00E-08 | | | | | | | 1.07 | | | | | | | | | Okada Y,PMID: 24390342 | | | | |  | |  | | |  | |
|  | AFF3 | rs9653442\|rs11676922\|rs10865035 | | | | | | 1.00E-14\|1.00E-14\|2.00E-08\|2.00E-06 | | | | | | | 1.12\|1.12\|1.12 | | | | | | | | | Okada Y,PMID: 24390342\|Stahl EA,PMID: 20453842\|Jiang L,PMID: 24782177\|Stahl EA,PMID: 20453842 | | | | |  | |  | | |  | |
|  | AHNAK2 | rs2582532 | | | | | | 3.00E-07 | | | | | | | 1.17 | | | | | | | | | Okada Y,PMID: 24390342 | | | | |  | |  | | |  | |
|  | AIRE | rs2075876\|rs760426 | | | | | | 4.00E-09\|4.40E-08 | | | | | | | 1.18\|1.16 | | | | | | | | | Terao C,PMID: 21505073\|Terao C,PMID: 21505073 | | | | |  | |  | | |  | |
|  | ANAPC4 | rs3816587 | | | | | | 9.00E-06 | | | | | | | 1.09 | | | | | | | | | WTCCC,PMID: 17554300 | | | | |  | |  | | |  | |
|  | ANKRD55 | rs77331626\|rs7731626\|rs6859219 | | | | | | 7.00E-24\|8.00E-23\|1.00E-11 | | | | | | | 1.21\|1.21\|1.28 | | | | | | | | | Okada Y,PMID: 24390342\|Okada Y,PMID: 24390342\|Stahl EA,PMID: 20453842 | | | | |  | |  | | |  | |
|  | ANXA3 | rs2867461 | | | | | | 1.00E-12 | | | | | | | 1.13 | | | | | | | | | Okada Y,PMID: 22446963 | | | | |  | |  | | |  | |
|  | APOM | rs805297 | | | | | | 3.00E-10 | | | | | | | 1.56 | | | | | | | | | Hu HJ,PMID: 21844665 | | | | |  | |  | | |  | |
|  | ARAP1 | rs3781913 | | | | | | 6.00E-10 | | | | | | | 1.12 | | | | | | | | | Okada Y,PMID: 22446963 | | | | |  | |  | | |  | |
|  | ARHGEF3 | rs2062583 | | | | | | 2.16E-06 | | | | | | | 0.63 | | | | | | | | | Freudenberg J,PMID: 21452313 | | | | |  | |  | | |  | |
|  | ARID5B | rs71508903\|rs71508903\|rs10821944 | | | | | | 1.00E-08\|1.00E-08\|6.00E-18 | | | | | | | 1.18\|1.18\|1.16 | | | | | | | | | Okada Y,PMID: 24390342\|Okada Y,PMID: 24390342\|Okada Y,PMID: 22446963 | | | | |  | |  | | |  | |
|  | ARL15 | rs255758 | | | | | | 7.00E-06 | | | | | | | 1.42 | | | | | | | | | Negi S,PMID: 23918589 | | | | |  | |  | | |  | |
|  | ATG5 | rs9372120 | | | | | | 4.00E-08 | | | | | | | 1.10 | | | | | | | | | Okada Y,PMID: 24390342 | | | | |  | |  | | |  | |
|  | ATM | chr11:107967350 | | | | | | 1.00E-08 | | | | | | | 1.21 | | | | | | | | | Okada Y,PMID: 24390342 | | | | |  | |  | | |  | |
|  | B3GNT2 | rs13385025\|rs11900673 | | | | | | 9.00E-07\|1.00E-08 | | | | | | | 1.11\|1.11 | | | | | | | | | Okada Y,PMID: 24390342\|Okada Y,PMID: 22446963 | | | | |  | |  | | |  | |
|  | BATF | rs7155603 | | | | | | 1.00E-07 | | | | | | | 1.16 | | | | | | | | | Stahl EA,PMID: 20453842 | | | | |  | |  | | |  | |
|  | Gene | SNP | | | | | | p-Value | | | | | | | Odd ratio | | | | | | | | | References | | | | |  | |  | | |  | |
|  | BLK | rs2736337\|rs1600249\|rs2736340 | | | | | | 2.00E-07\|5.00E-06\|1.22E-05\|6.00E-09 | | | | | | | 1.15\|0.77\|1.29\|1.19 | | | | | | | | | Okada Y,PMID: 24390342\|Freudenberg J,PMID: 21452313\|Freudenberg J,PMID: 21452313\|Gregersen PK,PMID: 19503088 | | | | |  | |  | | |  | |
|  | BTNL2 | rs3763309 | | | | | | 2.00E-124 | | | | | | | 2.30 | | | | | | | | | Orozco G,PMID: 24449572 | | | | |  | |  | | |  | |
|  | C1QBP | rs72634030 | | | | | | 2.00E-09 | | | | | | | 1.12 | | | | | | | | | Okada Y,PMID: 24390342 | | | | |  | |  | | |  | |
|  | C4orf52 | rs11933540 | | | | | | 1.00E-16 | | | | | | | 1.15 | | | | | | | | | Okada Y,PMID: 24390342 | | | | |  | |  | | |  | |
|  | C5 | rs10985070\|rs3761847\|rs881375 | | | | | | 4.00E-09\|2.00E-07\|4.00E-08 | | | | | | | 1.09\|1.13\|NR | | | | | | | | | Okada Y,PMID: 24390342\|Stahl EA,PMID: 20453842\|Gregersen PK,PMID: 19503088 | | | | |  | |  | | |  | |
|  | C5orf30 | rs2561477\|rs26232 | | | | | | 1.00E-10\|4.00E-08 | | | | | | | 1.09\|1.14 | | | | | | | | | Okada Y,PMID: 24390342\|Stahl EA,PMID: 20453842 | | | | |  | |  | | |  | |
|  | C6orf10 | rs9275406 | | | | | | 3.00E-12 | | | | | | | 2.10 | | | | | | | | | Negi S,PMID: 23918589 | | | | |  | |  | | |  | |
|  | CASP8 | rs6715284 | | | | | | 2.00E-09 | | | | | | | 1.15 | | | | | | | | | Okada Y,PMID: 24390342 | | | | |  | |  | | |  | |
|  | CCL19 | rs11574914 | | | | | | 2.00E-15 | | | | | | | 1.13 | | | | | | | | | Okada Y,PMID: 24390342 | | | | |  | |  | | |  | |
|  | CCL21 | rs951005\|rs2812378\|rs11574914 | | | | | | 4.00E-10\|3.00E-08\|2.00E-15 | | | | | | | 1.19\|1.12\|1.13 | | | | | | | | | Stahl EA,PMID: 20453842\|Raychaudhuri S,PMID: 18794853\|Okada Y,PMID: 24390342 | | | | |  | |  | | |  | |
|  | CCR6 | rs1571878\|rs3093023\|rs1854853\|rs3093024 | | | | | | 1.00E-22\|2.00E-11\|4.00E-09\|2.00E-10\|8.00E-19 | | | | | | | 1.28\|1.13\|NR\|NR | | | | | | | | | Okada Y,PMID: 24390342\|Stahl EA,PMID: 20453842\|Jiang L,PMID: 24782177\|Jiang L,PMID: 24782177\|Kochi Y,PMID: 20453841 | | | | |  | |  | | |  | |
|  | CD2 | rs624988 | | | | | | 8.00E-10 | | | | | | | 1.09 | | | | | | | | | Okada Y,PMID: 24390342 | | | | |  | |  | | |  | |
|  | CD226 | rs2469434 | | | | | | 1.00E-08 | | | | | | | NR | | | | | | | | | Okada Y,PMID: 24390342 | | | | |  | |  | | |  | |
|  | CD244 | rs11265493\|rs3753389\|rs3766379\|rs1319651\|rs6682654 | | | | | | 4.10E-07\|8.00E-08\|3.00E-08\|6.40E-07\|7.00E-08 | | | | | | | 1.28\|1.3\|1.31\|1.28\|1.31 | | | | | | | | | Suzuki A,PMID: 18794858\|Suzuki A,PMID: 18794858\|Suzuki A,PMID: 18794858\|Suzuki A,PMID: 18794858\|Suzuki A,PMID: 18794858 | | | | |  | |  | | |  | |
|  | Gene | | SNP | | | | | | p-Value | | | | | | | | | Odd ratio | | | | | References | | |  | |  | |  | |  | | |  |
|  | CD247 | | rs840016 | | | | | | 2.00E-06 | | | | | | | | | 1.11 | | | | | Stahl EA,PMID: 20453842 | | |  | |  | |  | |  | | |  |
|  | CD40 | | rs4239702\|rs4810485 | | | | | | 1.00E-16\|3.00E-09\|8.00E-09 | | | | | | | | | 1.14\|0.85\|1.15 | | | | | Okada Y,PMID: 24390342\|Stahl EA,PMID: 20453842\|Raychaudhuri S,PMID: 18794853 | | |  | |  | |  | |  | | |  |
|  | CD5 | | rs508970 | | | | | | 3.00E-06 | | | | | | | | | 1.07 | | | | | Okada Y,PMID: 24390342 | | |  | |  | |  | |  | | |  |
|  | CD83 | | chr6:14103212\|rs12529514 | | | | | | 3.00E-06\|2.00E-08 | | | | | | | | | 1.16\|1.14 | | | | | Okada Y,PMID: 24390342\|Okada Y,PMID: 22446963 | | |  | |  | |  | |  | | |  |
|  | CDK2 | | rs773125 | | | | | | 1.00E-10 | | | | | | | | | 1.09 | | | | | Okada Y,PMID: 24390342 | | |  | |  | |  | |  | | |  |
|  | CDK4 | | rs1633360 | | | | | | 1.00E-07 | | | | | | | | | 1.07 | | | | | Okada Y,PMID: 24390342 | | |  | |  | |  | |  | | |  |
|  | CDK5RAP2 | | rs12379034 | | | | | | 1.00E-12 | | | | | | | | | 1.34 | | | | | Jiang L,PMID: 24782177 | | |  | |  | |  | |  | | |  |
|  | CDK6 | | rs4272\|rs42041 | | | | | | 1.00E-08\|4.00E-06 | | | | | | | | | 1.10\|1.11 | | | | | Okada Y,PMID: 24390342\|Raychaudhuri S,PMID: 18794853 | | |  | |  | |  | |  | | |  |
|  | CEP57 | | rs4409785 | | | | | | 1.00E-11 | | | | | | | | | 1.12 | | | | | Okada Y,PMID: 24390342 | | |  | |  | |  | |  | | |  |
|  | CFLAR | | rs6715284 | | | | | | 2.00E-09 | | | | | | | | | 1.15 | | | | | Okada Y,PMID: 24390342 | | |  | |  | |  | |  | | |  |
|  | CLNK | | rs13142500 | | | | | | 2.00E-06 | | | | | | | | | 1.10 | | | | | Okada Y,PMID: 24390342 | | |  | |  | |  | |  | | |  |
|  | CLYBL | | rs9557321 | | | | | | 6.00E-08 | | | | | | | | | 1.73 | | | | | Bossini-Castillo L,PMID: 24532677 | | |  | |  | |  | |  | | |  |
|  | COG6 | | rs9603616 | | | | | | 2.00E-12 | | | | | | | | | 1.10 | | | | | Okada Y,PMID: 24390342 | | |  | |  | |  | |  | | |  |
|  | CSF2 | | rs657075\|rs657075 | | | | | | 6.00E-06\|3.00E-10 | | | | | | | | | 1.12\|1.12 | | | | | Okada Y,PMID: 24390342\|Okada Y,PMID: 22446963 | | |  | |  | |  | |  | | |  |
|  | CSF3 | | chr17:38031857 | | | | | | 2.00E-12 | | | | | | | | | 1.09 | | | | | Okada Y,PMID: 24390342 | | |  | |  | |  | |  | | |  |
|  | CTLA4 | | rs3087243\|rs3087243\|rs231775\|rs231735 | | | | | | 3.00E-25\|1.00E-08\|6.30E-07\|6.00E-09 | | | | | | | | | 1.14\|1.15\|1.09\|NR | | | | | Okada Y,PMID: 24390342\|Stahl EA,PMID: 20453842\|Doroth??e Diogo,PMID:23261300\|Gregersen PK,PMID: 19503088 | | |  | |  | |  | |  | | |  |
|  | CXCR5 | | rs10790268 | | | | | | 1.00E-15 | | | | | | | | | 1.14 | | | | | Okada Y,PMID: 24390342 | | |  | |  | |  | |  | | |  |
|  | Gene | | | | SNP | | | | | | | | p-Value | | | | | | Odd ratio | | | | | | References | | | |  | |  | | |  | |
|  | DNASE1L3 | | | | rs73081554 | | | | | | | | 5.00E-08 | | | | | | 1.18 | | | | | | Okada Y,PMID: 24390342 | | | |  | |  | | |  | |
|  | DPP4 | | | | rs12617656 | | | | | | | | 1.00E-08 | | | | | | 1.24 | | | | | | Jiang L,PMID: 24782177 | | | |  | |  | | |  | |
|  | EOMES | | | | rs3806624 | | | | | | | | 3.00E-08 | | | | | | 1.08 | | | | | | Okada Y,PMID: 24390342 | | | |  | |  | | |  | |
|  | ETS1 | | | | rs73013527\|rs4937362 | | | | | | | | 1.00E-06\|8.00E-07 | | | | | | 1.08\|1.09 | | | | | | Okada Y,PMID: 24390342\|Okada Y,PMID: 22446963 | | | |  | |  | | |  | |
|  | ETV7 | | | | rs2234067 | | | | | | | | 1.60E-09 | | | | | | 1.15 | | | | | | Okada Y,PMID: 24390342 | | | |  | |  | | |  | |
|  | FADS1 | | | | rs968567 | | | | | | | | 2.00E-08 | | | | | | 1.12 | | | | | | Okada Y,PMID: 24390342 | | | |  | |  | | |  | |
|  | FADS2 | | | | rs968567 | | | | | | | | 2.00E-08 | | | | | | 1.12 | | | | | | Okada Y,PMID: 24390342 | | | |  | |  | | |  | |
|  | FADS3 | | | | rs968567 | | | | | | | | 2.00E-08 | | | | | | 1.12 | | | | | | Okada Y,PMID: 24390342 | | | |  | |  | | |  | |
|  | FAM124A | | | | rs3790022 | | | | | | | | 1.00E-06 | | | | | | 1.49 | | | | | | Bossini-Castillo L,PMID: 24532677 | | | |  | |  | | |  | |
|  | FCGR2A | | | | rs72717009\|rs1801274\|rs11810143 | | | | | | | | 1.00E-07\|2.40E-07\|1.80E-07 | | | | | | 1.13\|1.10\|1.14 | | | | | | Okada Y,PMID: 24390342\|Doroth??e Diogo,PMID:23261300\|Doroth??e Diogo,PMID:23261300 | | | |  | |  | | |  | |
|  | FCRL3 | | | | rs2317230 | | | | | | | | 2.00E-07 | | | | | | 1.07 | | | | | | Okada Y,PMID: 24390342 | | | |  | |  | | |  | |
|  | FLI1 | | | | rs4937362 | | | | | | | | 8.00E-07 | | | | | | 1.09 | | | | | | Okada Y,PMID: 22446963 | | | |  | |  | | |  | |
|  | GATA3 | | | | rs3824660 | | | | | | | | 2.00E-08 | | | | | | 1.08 | | | | | | Okada Y,PMID: 24390342 | | | |  | |  | | |  | |
|  | GATSL3 | | | | rs1043099 | | | | | | | | 7.00E-09 | | | | | | 1.19 | | | | | | Orozco G,PMID: 24449572 | | | |  | |  | | |  | |
|  | GCH1 | | | | rs3783637 | | | | | | | | 2.00E-06 | | | | | | 1.10 | | | | | | Okada Y,PMID: 22446963 | | | |  | |  | | |  | |
|  | GMCL1L | | | | rs2961663 | | | | | | | | 4.00E-06 | | | | | | NR | | | | | | Padyukov L,PMID: 21156761 | | | |  | |  | | |  | |
|  | GPR125 | | | | rs6448119 | | | | | | | | 7.00E-06 | | | | | | NR | | | | | | Padyukov L,PMID: 21156761 | | | |  | |  | | |  | |
|  | GRHL2 | | | | rs678347 | | | | | | | | 2.00E-08 | | | | | | 1.08 | | | | | | Okada Y,PMID: 24390342 | | | |  | |  | | |  | |
|  | GRM5 | | | | rs518167 | | | | | | | | 2.00E-06 | | | | | | 2.24 | | | | | | Bossini-Castillo L,PMID: 24532677 | | | |  | |  | | |  | |
|  | HLA | | | | rs12194148\|rs2157337 | | | | | | | | 5.00E-58\|9.00E-52 | | | | | | NR\|NR | | | | | | Padyukov L,PMID: 21156761\|Padyukov L,PMID: 21156761 | | | |  | |  | | |  | |
|  | Gene | | | SNP | | | | | | p-Value | | | | Odd ratio | | | | | | References | | | | | | |  | |  | |  | | |  | |
|  | HLA-B | | | rs2596565 | | | | | | 9.00E-09 | | | | 1.40 | | | | | | Bossini-Castillo L,PMID: 24532677 | | | | | | |  | |  | |  | | |  | |
|  | HLA-DQA1 | | | rs9271348\|rs6457617\|rs9275406 | | | | | | 5.00E-07\|1.00E-09\|3.00E-12 | | | | 1.28\|NR\|2.10 | | | | | | Bossini-Castillo L,PMID: 24532677\|Julia A,PMID: 18668548\|Negi S,PMID: 23918589 | | | | | | |  | |  | |  | | |  | |
|  | HLA-DQA2 | | | rs12525220\|rs6457617\|rs9275406 | | | | | | 2.00E-13\|1.00E-09\|3.00E-12 | | | | 2.87\|NR\|2.10 | | | | | | Jiang L,PMID: 24782177\|Julia A,PMID: 18668548\|Negi S,PMID: 23918589 | | | | | | |  | |  | |  | | |  | |
|  | HLA-DQB1 | | | rs12525220\|rs9275406 | | | | | | 2.00E-13\|3.00E-12 | | | | 2.87\|2.10 | | | | | | Jiang L,PMID: 24782177\|Negi S,PMID: 23918589 | | | | | | |  | |  | |  | | |  | |
|  | IKZF3 | | | chr17:38031857\|rs2872507 | | | | | | 2.00E-12\|9.00E-07 | | | | 1.09\|1.10 | | | | | | Okada Y,PMID: 24390342\|Stahl EA,PMID: 20453842 | | | | | | |  | |  | |  | | |  | |
|  | IL2 | | | rs45475795\|rs13119723 | | | | | | 4.00E-06\|7.00E-07 | | | | 1.14\|1.12 | | | | | | Okada Y,PMID: 24390342\|Stahl EA,PMID: 20453842 | | | | | | |  | |  | |  | | |  | |
|  | IL20RB | | | rs9826828 | | | | | | 9.00E-10 | | | | 1.44 | | | | | | Okada Y,PMID: 24390342 | | | | | | |  | |  | |  | | |  | |
|  | IL21 | | | rs45475795\|rs13119723 | | | | | | 4.00E-06\|7.00E-07 | | | | 1.14\|1.12 | | | | | | Okada Y,PMID: 24390342\|Stahl EA,PMID: 20453842 | | | | | | |  | |  | |  | | |  | |
|  | IL2RA | | | rs706778\|rs706778\|rs2228150\|rs2104286 | | | | | | 5.00E-14\|1.00E-11\|6.60E-06\|1.00E-06 | | | | 1.10\|1.14\|1.25\|1.19 | | | | | | Okada Y,PMID: 24390342\|Stahl EA,PMID: 20453842\|Doroth??e Diogo,PMID:23261300\|Orozco G,PMID: 24449572 | | | | | | |  | |  | |  | | |  | |
|  | IL2RB | | | rs3218251 | | | | | | 6.00E-06 | | | | 1.08 | | | | | | Okada Y,PMID: 24390342 | | | | | | |  | |  | |  | | |  | |
|  | IL3 | | | rs657075 | | | | | | 6.00E-06 | | | | 1.12 | | | | | | Okada Y,PMID: 24390342 | | | | | | |  | |  | |  | | |  | |
|  | IL6R | | | rs2228145 | | | | | | 4.00E-09 | | | | 1.08 | | | | | | Okada Y,PMID: 24390342 | | | | | | |  | |  | |  | | |  | |
|  | IL6ST | | | rs6859219 | | | | | | 1.00E-11 | | | | 1.28 | | | | | | Stahl EA,PMID: 20453842 | | | | | | |  | |  | |  | | |  | |
|  | INPP5B | | | rs28411352 | | | | | | 3.00E-12 | | | | 1.11 | | | | | | Okada Y,PMID: 24390342 | | | | | | |  | |  | |  | | |  | |
|  | intergenic | | | rs12413578 | | | | | | 5.00E-08 | | | | NR | | | | | | Okada Y,PMID: 24390342 | | | | | | |  | |  | |  | | |  | |
|  | IRAK1 | | | rs5987194 | | | | | | 3.00E-12 | | | | 1.16 | | | | | | Okada Y,PMID: 24390342 | | | | | | |  | |  | |  | | |  | |
|  | IRF4 | | | rs9378815 | | | | | | 1.00E-07 | | | | 1.09 | | | | | | Okada Y,PMID: 24390342 | | | | | | |  | |  | |  | | |  | |
|  | IRF5 | | | chr7:128580042\|rs10488631\|rs3807306 | | | | | | 1.00E-14\|4.00E-11\|3.00E-07 | | | | 1.12\|1.19\|1.44 | | | | | | Okada Y,PMID: 24390342\|Stahl EA,PMID: 20453842\|Padyukov L,PMID: 21156761 | | | | | | |  | |  | |  | | |  | |
|  | IRF8 | | | rs13330176\|rs2280381 | | | | | | 1.00E-12\|2.00E-06 | | | | 1.12\|1.12 | | | | | | Okada Y,PMID: 24390342\|Okada Y,PMID: 22446963 | | | | | | |  | |  | |  | | |  | |
|  | JAZF1 | | | rs67250450 | | | | | | 3.00E-09 | | | | 1.11 | | | | | | Okada Y,PMID: 24390342 | | | | | | |  | |  | |  | | |  | |
|  | Gene | | | | | | SNP | | | | | p-Value | | | | | Odd ratio | | | | | References | | | | | | | | | | |  | | |
|  | KCNIP4 | | | | | | rs6448119 | | | | | 7.00E-06 | | | | | NR | | | | | Padyukov L,PMID: 21156761 | | | | | | | | | | |  | | |
|  | KIF3 | | | | | | rs17374222 | | | | | 2.00E-06 | | | | | 1.13 | | | | | Stahl EA,PMID: 20453842 | | | | | | | | | | |  | | |
|  | KIF5A | | | | | | rs1678542\|rs1678542 | | | | | 1.00E-07\|9.00E-08 | | | | | 1.20\|1.12 | | | | | Orozco G,PMID: 24449572\|Raychaudhuri S,PMID: 18794853 | | | | | | | | | | |  | | |
|  | LBH | | | | | | rs10175798 | | | | | 1.00E-09 | | | | | 1.08 | | | | | Okada Y,PMID: 24390342 | | | | | | | | | | |  | | |
|  | LOC339442 | | | | | | rs12140275 | | | | | 2.00E-09 | | | | | 1.11 | | | | | Okada Y,PMID: 24390342 | | | | | | | | | | |  | | |
|  | MED1 | | | | | | rs1877030 | | | | | 2.00E-08 | | | | | 1.09 | | | | | Okada Y,PMID: 24390342 | | | | | | | | | | |  | | |
|  | MHC | | | | | | rs7748270\|rs6457617\|rs12525220 | | | | | 1.00E-16\|5.00E-75 \|2.00E-13 | | | | | 2.01\|2.36\|2.87 | | | | | Jiang L,PMID: 24782177\|WTCCC,PMID: 17554300\|Jiang L,PMID: 24782177 | | | | | | | | | | |  | | |
|  | MICA | | | | | | rs2596565 | | | | | 9.00E-09 | | | | | 1.40 | | | | | Bossini-Castillo L,PMID: 24532677 | | | | | | | | | | |  | | |
|  | MMEL1 | | | | | | chr1:2523811\|rs3890745 | | | | | 5.00E-09\|1.00E-07 | | | | | 1.10\|1.12 | | | | | Okada Y,PMID: 24390342\|Raychaudhuri S,PMID: 18794853 | | | | | | | | | | |  | | |
|  | MTF1 | | | | | | rs28411352 | | | | | 3.00E-12 | | | | | 1.11 | | | | | Okada Y,PMID: 24390342 | | | | | | | | | | |  | | |
|  | NFKBIE | | | | | | rs2233424\|rs2233434 | | | | | 1.00E-19\|6.00E-19 \|1.00E-15 | | | | | 1.26\|1.19 | | | | | Okada Y,PMID: 24390342\|Okada Y,PMID: 22446963\|Myouzen K,PMID: 23028356 | | | | | | | | | | |  | | |
|  | OLIG3 | | | | | | rs2230926\|rs6920220\|rs10499194 | | | | | 2.00E-06\|1.00E-07 \|1.00E-09 | | | | | 1.31\|1.22\|1.33 | | | | | Kochi Y,PMID: 20453841\|Plenge RM,PMID: 17982456\|Plenge RM,PMID: 17982456 | | | | | | | | | | |  | | |
|  | P2RY10 | | | | | | chrX:78464616 | | | | | 4.00E-08 | | | | | 1.11 | | | | | Okada Y,PMID: 24390342 | | | | | | | | | | |  | | |
|  | PADI4 | | | | | | rs2301888\|rs2240335 | | | | | 1.00E-18\|2.00E-08 | | | | | 1.13\|1.50 | | | | | Okada Y,PMID: 24390342\|Freudenberg J,PMID: 21452313 | | | | | | | | | | |  | | |
|  | PDE2A | | | | | | rs3781913 | | | | | 6.00E-10 | | | | | 1.12 | | | | | Okada Y,PMID: 22446963 | | | | | | | | | | |  | | |
|  | PIP4K2C | | | | | | rs1678542 | | | | | 9.00E-08 | | | | | 1.12 | | | | | Raychaudhuri S,PMID: 18794853 | | | | | | | | | | |  | | |
|  | PLCL2 | | | | | | rs4452313 | | | | | 2.00E-10 | | | | | NR | | | | | Okada Y,PMID: 24390342 | | | | | | | | | | |  | | |
|  | PLD4 | | | | | | rs2582532\|rs2841277 | | | | | 3.00E-07\|2.00E-14 | | | | | 1.17\|1.15 | | | | | Okada Y,PMID: 24390342\|Okada Y,PMID: 22446963 | | | | | | | | | | |  | | |
|  | POU3F1 | | | | | | rs12131057 | | | | | 4.00E-07 | | | | | 1.16 | | | | | Stahl EA,PMID: 20453842 | | | | | | | | | | |  | | |
|  | PPIL4 | | | | | | rs9373594 | | | | | 3.00E-09 | | | | | 1.09 | | | | | Okada Y,PMID: 24390342 | | | | | | | | | | |  | | |
|  | PRKCB1 | | | | | | rs7404928 | | | | | 4.00E-06 | | | | | 1.08 | | | | | Okada Y,PMID: 22446963 | | | | | | | | | | |  | | |
|  | PRKCH | | | | | | rs3783782\|rs1957895 | | | | | 2.00E-09\|4.00E-07 | | | | | 1.14\|1.09 | | | | | Okada Y,PMID: 24390342\|Okada Y,PMID: 22446963 | | | | | | | | | | |  | | |
|  | PRKCQ | | | | | | rs947474\|rs4750316 | | | | | 3.00E-10\|2.00E-06\|4.00E-06 | | | | | 1.12\|1.15\|1.14 | | | | | Okada Y,PMID: 24390342\|Stahl EA,PMID: 20453842\|Raychaudhuri S,PMID: 18794853 | | | | | | | | | | |  | | |
| Gene | | | | | | SNP | | | | | | p-Value | | | | | Odd ratio | | | | | References | | | | | | | | | | | | | |
| PTPN11 | | | | | | rs10774624 | | | | | | 7.00E-09 | | | | | 1.09 | | | | | Okada Y,PMID: 24390342 | | | | | | | | | | | | | |
| PTPN2 | | | | | | rs8083786\|rs2847297 | | | | | | 2.00E-11\|2.00E-08 | | | | | 1.18\|1.10 | | | | | Okada Y,PMID: 24390342\|Okada Y,PMID: 22446963 | | | | | | | | | | | | | |
| PTPN22 | | | | | | rs2476601\|rs2476601\|rs6679677 | | | | | | 9.00E-170\|9.00E-74\|0\|1.00E-08\|6.00E-42\|6.00E-25 | | | | | 1.80\|1.94\|1.82\|1.79\|1.98 | | | | | Okada Y,PMID: 24390342\|Stahl EA,PMID: 20453842\|Doroth??e Diogo,PMID:23261300\|Padyukov L,PMID: 21156761\|Raychaudhuri S,PMID: 18794853\|WTCCC,PMID: 17554300 | | | | | | | | | | | | | |
| RAD51B | | | | | | rs1950897 | | | | | | 5.00E-08 | | | | | 1.09 | | | | | Okada Y,PMID: 24390342 | | | | | | | | | | | | | |
| RAG1 | | | | | | rs331463 | | | | | | 1.00E-07 | | | | | 1.12 | | | | | Okada Y,PMID: 24390342 | | | | | | | | | | | | | |
| RAG2 | | | | | | rs331463 | | | | | | 1.00E-07 | | | | | 1.12 | | | | | Okada Y,PMID: 24390342 | | | | | | | | | | | | | |
| RASGRP1 | | | | | | rs8032939 | | | | | | 2.00E-18 | | | | | 1.13 | | | | | Okada Y,PMID: 24390342 | | | | | | | | | | | | | |
| RBPJ | | | | | | rs874040\|rs6448432 | | | | | | 1.00E-16\|4.00E-07 | | | | | 1.14\|1.19 | | | | | Stahl EA,PMID: 20453842\|Orozco G,PMID: 24449572 | | | | | | | | | | | | | |
| RCAN1 | | | | | | chr21:35928240 | | | | | | 3.00E-07 | | | | | 1.11 | | | | | Okada Y,PMID: 24390342 | | | | | | | | | | | | | |
| REL | | | | | | rs34695944\|rs13031237\|rs13017599 | | | | | | 2.00E-15\|8.00E-07\|2.00E-12 | | | | | 1.12\|1.13\|NR | | | | | Okada Y,PMID: 24390342\|Stahl EA,PMID: 20453842\|Gregersen PK,PMID: 19503088 | | | | | | | | | | | | | |
| RNASEH2B | | | | | | rs3790022 | | | | | | 1.00E-06 | | | | | 1.4925 | | | | | Bossini-Castillo L,PMID: 24532677 | | | | | | | | | | | | | |
| RPS12P4 | | | | | | rs4305317 | | | | | | 2.00E-06 | | | | | 1.45 | | | | | Padyukov L,PMID: 21156761 | | | | | | | | | | | | | |
| RTKN2 | | | | | | rs6479800\|rs3125734 | | | | | | 4.00E-06\|5.00E-09 | | | | | 1.19\|NR | | | | | Okada Y,PMID: 24390342\|Myouzen K,PMID: 23028356 | | | | | | | | | | | | | |
| RUNX1 | | | | | | rs8133843 | | | | | | 2.00E-08 | | | | | 1.09 | | | | | Okada Y,PMID: 24390342 | | | | | | | | | | | | | |
| SALL3 | | | | | | rs2002842 | | | | | | 6.00E-06 | | | | | 1.61 | | | | | Julia A,PMID: 18668548 | | | | | | | | | | | | | |
| SFTPD | | | | | | rs726288 | | | | | | 9.00E-09 | | | | | 1.22 | | | | | Okada Y,PMID: 24390342 | | | | | | | | | | | | | |
| SH2B3 | | | | | | rs10774624\|rs3184504 | | | | | | 7.00E-09\|6.00E-06 | | | | | 1.09\|1.08 | | | | | Okada Y,PMID: 24390342\|Stahl EA,PMID: 20453842 | | | | | | | | | | | | | |
| SMIM21 | | | | | | rs1943199 | | | | | | 2.00E-08 | | | | | 1.94 | | | | | Bossini-Castillo L,PMID: 24532677 | | | | | | | | | | | | | |
| SPRED2 | | | | | | rs1858037\|rs934734 | | | | | | 1.00E-08\|5.00E-10\|2.00E-08 | | | | | 1.19\|1.13 | | | | | Okada Y,PMID: 24390342\|Stahl EA,PMID: 20453842\|Jiang L,PMID: 24782177 | | | | | | | | | | | | | |
| STAT4 | | | | | | rs11889341\|rs7574865 | | | | | | 1.00E-12\|3.00E-07\|2.00E-06 | | | | | 1.12\|1.16 | | | | | Okada Y,PMID: 24390342\|Stahl EA,PMID: 20453842\|Kochi Y,PMID: 20453841 | | | | | | | | | | | | | |
| SYNGR1 | | | | | | rs909685 | | | | | | 1.00E-16 | | | | | 1.13 | | | | | Okada Y,PMID: 24390342 | | | | | | | | | | | | | |
| TAGAP | | | | | | rs2451258 | | | | | | 2.00E-10 | | | | | 1.10 | | | | | Okada Y,PMID: 24390342 | | | | | | | | | | | | | |
| Gene | | | | | | SNP | | | | | p-Value | | | | | Odd ratio | | | | | References | | | | | | | | | |  | | |  | |
| TEC | | | | | | rs2664035 | | | | | 1.00E-07 | | | | | 1.07 | | | | | Okada Y,PMID: 24390342 | | | | | | | | | |  | | |  | |
| TNFAIP3 | | | | | | rs7752903\|rs6920220\|rs2230926\|rs10499194 | | | | | 2.00E-20\|9.00E-13\|1.00E-07\|6.80E-14\|2.00E-06\|1.00E-09 | | | | | 1.41\|1.22\|1.38\|1.33 | | | | | Okada Y,PMID: 24390342\|Stahl EA,PMID: 20453842\|Plenge RM,PMID: 17982456\|Doroth??e Diogo,PMID:23261300\|Kochi Y,PMID: 20453841\|Plenge RM,PMID: 17982456 | | | | | | | | | |  | | |  | |
| TNFRSF14 | | | | | | chr1:2523811\|rs3890745 | | | | | 5.00E-09\|4.00E-06\|1.00E-06\|1.00E-07 | | | | | 1.10\|1.12\|NR\|  1.12 | | | | | Okada Y,PMID: 24390342\|Stahl EA,PMID: 20453842\|Orozco G,PMID: 24449572\|Raychaudhuri S,PMID: 18794853 | | | | | | | | | |  | | |  | |
| TNFRSF9 | | | | | | rs227163 | | | | | 3.00E-09 | | | | | 1.11 | | | | | Okada Y,PMID: 24390342 | | | | | | | | | |  | | |  | |
| TRAF6 | | | | | | rs331463 | | | | | 1.00E-07 | | | | | 1.12 | | | | | Okada Y,PMID: 24390342 | | | | | | | | | |  | | |  | |
| TRHDE | | | | | | rs12831974 | | | | | 6.00E-06 | | | | | 1.27 | | | | | Freudenberg J,PMID: 21452313 | | | | | | | | | |  | | |  | |
| TXNDC11 | | | | | | rs4780401 | | | | | 4.00E-08 | | | | | 1.07 | | | | | Okada Y,PMID: 24390342 | | | | | | | | | |  | | |  | |
| TYK2 | | | | | | rs34536443 | | | | | 5.00E-16 | | | | | 1.46 | | | | | Okada Y,PMID: 24390342 | | | | | | | | | |  | | |  | |
| UBASH3A | | | | | | rs1893592\|rs11203203 | | | | | 7.00E-12\|4.00E-06 | | | | | 1.11\|1.11 | | | | | Okada Y,PMID: 24390342\|Stahl EA,PMID: 20453842 | | | | | | | | | |  | | |  | |
| UBE2L3 | | | | | | rs11089637 | | | | | 2.00E-07 | | | | | 1.10 | | | | | Okada Y,PMID: 24390342 | | | | | | | | | |  | | |  | |
| WDFY4 | | | | | | rs2671692 | | | | | 3.00E-09 | | | | | 1.07 | | | | | Okada Y,PMID: 24390342 | | | | | | | | | |  | | |  | |
| YDJC | | | | | | rs11089637 | | | | | 2.00E-07 | | | | | 1.10 | | | | | Okada Y,PMID: 24390342 | | | | | | | | | |  | | |  | |
| ZNF438 | | | | | | rs793108 | | | | | 1.00E-09 | | | | | 1.08 | | | | | Okada Y,PMID: 24390342 | | | | | | | | | |  | | |  | |
| ZNF774 | | | | | | rs6496667 | | | | | 1.00E-06 | | | | | 1.09 | | | | | Okada Y,PMID: 22446963 | | | | | | | | | |  | | |  | |
