# Supplementary material for: Identification of potential genetic causal variants for rheumatoid arthritis by whole-exome sequencing
Source: Oncotarget. 2017 Nov 22;8(67):111119–29. doi: 10.18632/oncotarget.22630 (PMC5762310; doi:10.18632/oncotarget.22630)
Supplement: Supplementary file 6 [file oncotarget-08-111119-s006.docx]

**Supplementary Table 7:** C**omparison of X chromosome associated novel variants distribution between female and male in RA patients.**


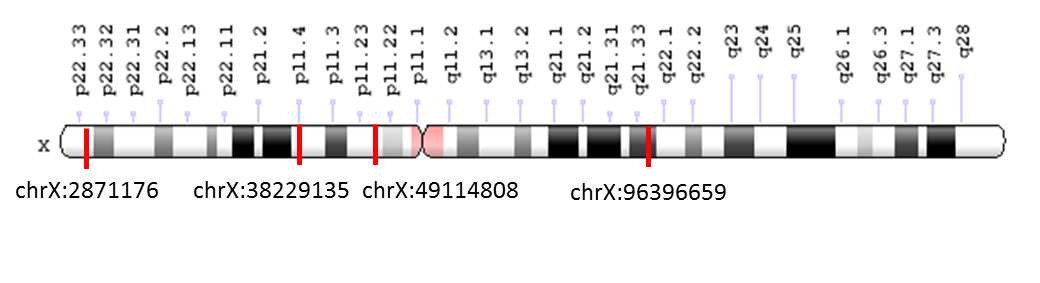


| group | variant | ref | alt | gene | gender | No. of cases with alt alleles |
| --- | --- | --- | --- | --- | --- | --- |
| 2 | chrX:38229135  (rs72554348) | G | C | OCT | female | 4 |
|  |  |  |  |  | male | 1 |
| 5 | chX:96396659  (rs363755) | C | T | DIAPH2 | female | 2 |
|  |  |  |  |  | male | 1 |
| 6 | chX:2871176  (rs56393981) | G | A | ARSE | female | 4 |
|  |  |  |  |  | male | 1 |
| 6 | chX:49114808 | C | A | FOXP3 | female | 7 |
|  |  |  |  |  | male | 1 |
